# Network Pharmacology-Based Strategy to Identify the Pharmacological Mechanisms of *Pulsatilla* Decoction against Crohn’s Disease

**DOI:** 10.3389/fphar.2022.844685

**Published:** 2022-04-05

**Authors:** Jinguo Liu, Lu Zhang, Zhaojun Wang, Shanshan Chen, Shuyan Feng, Yujin He, Shuo Zhang

**Affiliations:** ^1^ The First Affiliated Hospital, Zhejiang Chinese Medical University, Hangzhou, China; ^2^ The First Affiliated Hospital, Zhejiang Chinese Medical University, Hangzhou, China; ^3^ Department of Gastroenterology, Edong Healthcare City Hospital of Traditional Chinese Medicine, Hubei Chinese Medical University, Wuhan, China; ^4^ Department of Gastroenterology, The Second Affiliated Hospital, Zhejiang Chinese Medical University, Hangzhou, China

**Keywords:** Crohn’s disease, *Pulsatilla* decoction, network pharmacology, pharmacological mechanisms, fibrosis

## Abstract

**Purpose:** To explore pharmacological mechanisms of *Pulsatilla* decoction (PD) against Crohn’s disease (CD) *via* network pharmacology analysis followed by experimental validation.

**Methods:** Public databases were searched to identify bioactive compounds and related targets of PD as well as related genes in patients with CD. Analyses using the drug–compound–target–disease network, the protein–protein interaction (PPI) network, and Gene Ontology (GO) and Kyoto Encyclopedia of Genes and Genomes (KEGG) enrichment analyses were performed to predict the core targets and pathways of PD against CD. Colon tissue resected from patients with CD and tissue samples from a mouse model of CD fibrosis treated with PD were assessed to verify the major targets of PD in CD predicted by network pharmacologic analysis.

**Results:** A search of the targets of bioactive compounds in PD and targets in CD identified 134 intersection targets. The target HSP90AA1, which was common to the drug–compound–target–disease and PPI networks, was used to simulate molecular docking with the corresponding bioactive compound. GO and KEGG enrichment analyses showed that multiple targets in the antifibrotic pathway were enriched and could be experimentally validated in CD patients and in a mouse model of CD fibrosis. Assays of colon tissues from CD patients showed that intestinal fibrosis was greater in stenoses than in nonstenoses, with upregulation of p-AKT, AKT, p-mTOR, mTOR, p-ERK1/2, ERK1/2, p-PKC, and PKC targets. Treatment of CD fibrosis mice with PD reduced the degree of fibrosis, with downregulation of the p-AKT, AKT, p-mTOR, mTOR, p-ERK1/2, ERK1/2, and PKC targets.

**Conclusion:** Network pharmacology analysis was able to predict bioactive compounds in PD and their potential targets in CD. Several of these targets were validated experimentally, providing insight into the pharmacological mechanisms underlying the biological activities of PD in patients with CD.

## Introduction

Crohn’s disease (CD) is a chronic inflammatory disease of the gastrointestinal tract with a progressive and destructive course and an increasing incidence worldwide (Roda et al., 2020). In China, the pooled incidence rate of CD between 2011 and 2013 was 0.34 [95% confidence interval (CI) 0.16–0.75; *p* < 0.001] per 100,000 person-years (Li et al., 2019). Although this rate has tended to increase over the last 10 years (Ng et al., 2017; Li and Shi, 2018), up-to-date epidemiological studies are lacking. The most frequent clinical manifestations of CD include gastroenterological symptoms, such as chronic abdominal pain, diarrhea, and obstruction and perianal lesions, and systematic symptoms, such as weight loss and fatigue. CD may be the final result of complex interactions involving genetic susceptibility, alterations in gut microbiota, and environmental and immune factors (Torres et al., 2017; Roda et al., 2020), but its etiology remains unclear. CD can be treated with biological agents, with or without immunosuppressants and corticosteroids. The focus of treatment has altered from maintaining remission to mucosal healing. Up to 70% of CD patients have been reported to undergo surgery for fibrotic strictures due to lack of specific and valid antifibrotic drugs (Yoo et al., 2020). In addition, it is difficult to balance the benefits and risks of these drugs, with certain patients developing reduced sensitivity to secondary treatments.

Combinations of traditional Chinese herbs and Western medicines have recently been used to treat patients with CD. The inclusion of traditional Chinese herbs has been found to reduce the dosage and frequency of modern drugs as well as alleviate their side effects (Wang et al., 2019a). The treatment of complex disorders with single-target drugs has resulted in poor therapeutic responses and high rates of toxic side effects. Traditional Chinese medicine (TCM), which consists of mixtures of components, has certain advantages, including synergies of these components in addressing various pathways and targets (Zhang et al., 2016). Nevertheless, the use of TCM to treat various diseases is limited due to lack of evidence in animal models and high-quality clinical trials (Teschke et al., 2015). *Pulsatilla* decoction (PD), comprising *Pulsatilla chinensis, Phellodendron chinense, Coptis chinensis*, and *Cortex fraxini*, is a TCM used to treat CD-like diseases (Liu et al., 2015). PD has been shown to alleviate acute colitis in mice by suppressing inflammation and epithelial barrier disruption (Wang et al., 2016; Wang et al., 2018). Few studies have assessed the activity of PD in chronic colitis, especially its antifibrotic effect. *C. chinensis*, one of the herbs in PD, has been widely studied in CD. Berberine, an isoquinoline alkaloid extracted from *C. chinensis*, has been shown to reduce intestinal barrier leakage of various luminal substances, thus reducing morbidity in inflammatory bowel disease (IBD), including CD (DiGuilio et al., 2016). Berberine has also been found to ameliorate IBD by suppressing Th1/Th17 differentiation and inflammatory responses (Li et al., 2015a). However, the key mechanisms underlying the effects of PD in CD remain unknown.

The pharmacological relationships between drugs and targets can be assessed by network pharmacology, which links systems biology with pharmacodynamic and pharmacokinetic characteristics (Cui et al., 2018). The effects of drugs on complex diseases can be systematically analyzed using drug–target–gene–disease interplay networks (Jing et al., 2019). Moreover, network pharmacology can target multiple nodes in interacting molecular systems, resulting in increased efficacy and fewer adverse reactions (Hopkins, 2007; Hopkins, 2008). This approach is in accordance with the holistic concept of TCM, which diagnoses and treats diseases from a systemic perspective (Chen et al., 2016). Network pharmacology analyses of the relationships among the four components of PD and targets of CD may identify the underlying signal pathways of PD against CD, with key targets verified experimentally.

## Materials and Methods

### Screening of Bioactive Compounds and Disease Targets

The bioactive compounds of the four components of PD were screened from the Traditional Chinese Medicine Database and Analysis Platform (TCMSP) database (https://www.tcmsp-e.com/) (Ru et al., 2014). Screening criteria included oral bioavailability (OB) ≥30% and drug likeness ≥0.18 (Eder et al., 2019), based on the absorption, distribution, metabolism, and excretion (ADME) model. Targets associated with these compounds were also collected from the TCMSP database. These compound-related targets were converted to gene symbols using the annotated UniProt database (https://www.uniprot.org/), filtered by previously identified human genes.

The CD-related genes were collected from GeneCards (https://www.genecards.org/), OMIM (https://omim.org/), TTD (http://db.idrblab.net/ttd/), PharmGkb (https://www.pharmgkb.org/), and DrugBank (https://www.drugbank.com/) databases. These genes were compiled and duplicates were removed. Genes related to both CD and the compounds in PD were subsequently identified.

### Network Construction

Cytoscape 3.8.0 was used to construct the drug–compound–target–disease network from the prepared form, in which the nodes indicated the potential bioactive compounds and target genes and the edges indicated their interrelationships. A degree-sorted circle layout was applied to determine the degree of the nodes of target genes, with the circle layout subsequently transformed into a grid layout.

Drug–disease intersecting genes were uploaded on the Online STRING 11.5 database (https://string-db.org/) to create the protein–protein interaction (PPI) network. The organism was limited to *Homo sapiens*, the minimum required interaction score was set to 0.9, and disconnected nodes in the network were hidden.

### Screening the Core of the PPI Network

The tabular text of the PPI network was imported in Cytoscape, and CytoNCA was used to filter the target genes. The first filtration conditions consisted of Betweenness ≥ 47.50701087, Closeness ≥ 0.2071495785, Degree ≥ 10, Eigenvector ≥ 0.0415849315, LAC ≥ 3.2, and Network ≥ 4.266666667. The duplicate filtration conditions consisted of Betweenness ≥ 10.07131665, Closeness ≥ 0.524590164, Degree ≥ 16, Eigenvector ≥ 0.120196819, LAC ≥ 8.8, and Network ≥ 9.890909091. The filtered genes were utilized to construct the subnetwork, and the final network was subjected to duplicate filtering.

### Molecular Docking of Protein Receptors and Small Molecule Ligands

The 3D conformers of the PPI core were selected as protein receptors for molecular docking from the PDB database (https://www.rcsb.org/) with a UniProt molecule name. PyMOL 2.4 was chosen to remove water molecules and small-molecule ligands of 3D conformers. The bioactive compounds of the PPI core were considered small-molecule ligands. Their 2D structures were obtained from PubChem (https://pubchem.ncbi.nlm.nih.gov/), and their homologous 3D conformers were drawn by Chem3D of ChemBioOffice 2014 with minimum free energy optimization. Finally, the 3D conformers of protein receptors and small-molecule ligands were used for molecular docking in Vina, and the docking position of the minimum free energy appeared in PyMOL.

### Bioinformatic Annotation and Pathway Drawing

The biological functions of target genes were annotated, and their pathways were drawn in R software using the Bioconductor package in Gene Ontology (GO) (http://www.geneontology.org/) and Kyoto Encyclopedia of Genes and Genomes (KEGG) (https://www.genome.jp/kegg/) databases. The GO enrichment analysis annotated gene functions, and the KEGG enrichment analysis showed gene-related pathways.

### Experimental Design

Surgically resected colon segments from stenotic and nonstenotic areas as close as possible to the resection margins were collected from eight patients with documented CD who underwent colectomy in the First Affiliated Hospital of Zhejiang Chinese Medical University (Hangzhou, China). All patients provided written and oral informed consent, and the study protocol was approved by the Institutional Ethics Committee of Zhejiang Chinese Medical University (2021-KL-209-01).

A total of 25 male C57BL/6 mice, aged 6–8 weeks and weighing 16–20 g, were obtained from Shanghai Slake Experimental Animal Co., Ltd (Shanghai, China) and fed standard laboratory water and food freely. The mice were randomly divided into three groups: mice in the PD group (*n* = 10) were orally administered with PD solution once daily for six continuous weeks, and mice in the model (*n* = 10) and normal (*n* = 5) groups were orally administered an equivalent volume of normal saline once daily for 6 weeks. After 1 week, mice in the PD and model groups were administered 2,4,6-trinitro-benzene sulfonic acid (TNBS; Sigma-Aldrich, United States), as described (Wirtz et al., 2017). Briefly, the mice were pre-sensitized by spraying 150 µl of 1% (wt/vol) TNBS solution on shaved skin area on the back for 1 week. Mice in the PD and model groups were starved for 24 h, followed by once weekly enteral administration of 100 µl of 0.5, 1.5, 2.5, 2.5, 2.5, or 2.5% TNBS in 50% ethanol using a 1-ml syringe and a 3.5-F catheter for continuous 6 weeks. During this time, mice in the PD and model groups were administered once daily with PD solution and normal saline, respectively. Finally, the mice were killed and their colons were collected for subsequent experiments. The animal study was approved by the Institutional Animal Care and Use Committee of Zhejiang Chinese Medical University (IACUC-20211018-06).

### Western Blotting Assay

Proteins were extracted from the colon tissues of patients and mice using the radioimmunoprecipitation assay lysis buffer supplemented with phenylmethanesulfonylfluoride (PMSF) and a protease and phosphatase inhibitor cocktail (Solarbio, China). Protein concentrations were quantified using BCA protein assay kits (Beyotime, China), and equal aliquots were mixed with the sodium dodecyl sulfate–polyacrylamide gel electrophoresis (SDS-PAGE) sample loading buffer (Beyotime). The protein samples were separated by SDS-PAGE (Beyotime), transferred to Hybond polyvinylidene fluoride (PVDF) membranes (GE Healthcare, United States), blocked with 5% nonfat milk for 1 h, and incubated overnight at 4°C with the following primary antibodies: mouse anti-AKT (1:1,000, ProteinTech, United States), mouse anti-AKT (phospho Ser473, 1:1,000, ImmunoWay, United States), mouse anti-mTOR (1:1,000, ProteinTech), mouse anti-mTOR (phospho Ser2448, 1:1,000, ImmunoWay), rabbit anti-PKCs (1:1,000, ImmunoWay), rabbit anti-PKCs (phospho Thr2647, 1:1,000, Affinity, China), rabbit anti-ERK1/2 (1:1,000, ImmunoWay), rabbit anti-ERK1/2 (phospho Tyr204, 1:1,000, ImmunoWay), rabbit anti-α-SMA (1:1,000, Abcam, United Kingdom), rabbit anti-vimentin (1:1,000, Abcam), and mouse anti-β-actin (1:5,000, ProteinTech), which were used as an internal control. The membranes were washed three times with Tris-buffered saline containing Tween 20 (TBST) and incubated with the appropriate horseradish peroxidase (HRP)–labeled goat secondary antibodies (1:5,000, Thermo Fisher Scientific, United States) at room temperature for 1 h. The membranes were incubated with enhanced chemiluminescence (ECL) solution (Applygen, China), and protein expression was visualized using a chemiluminescence imager (Clinx Science Instruments, China).

### Real-Time Quantitative PCR Assay

Total RNA was extracted from colon tissues using a tissue RNA purification kit (ESscience Biotech, China), and cDNA was synthesized using the HiScript^®^ II Q RT SuperMix for qPCR (+gDNA wiper) (Vazyme, China), according to the manufacturer’s instructions. The OD 260/OD 280 ratio of the extracted RNA samples ranged from 1.8 to 2.0. Real-time quantitative PCR was performed with Taq Pro Universal SYBR qPCR Master Mix (Vazyme), and the primer sequences are shown in Tables 1, 2. The reaction conditions were as follows: initial denaturation at 95°C for 10 min, followed by 40 cycles of denaturation at 95°C for 15 s, and annealing and extension at 60°C for 30 s; a dissolution curve of 95°C for 15 s, 60°C for 60 s, and 95°C for 15 s was obtained. ACTB was used as the internal reference gene, and relative expression of target genes was assessed using the 2^−∆∆Ct^ method (Livak and Schmittgen, 2001).

**TABLE 1:** Human PCR primers.

Gene	GenBank Accession	Primer Sequences (5′–3′)	Product Length
AKT	NM_001382431.1	F: TGG​ACT​ACC​TGC​ACT​CGG​AGA​A	154 bp
		R: GTG​CCG​CAA​AAG​GTC​TTC​ATG​G	
mTOR	NM_001386500.1	F: AGC​ATC​GGA​TGC​TTA​GGA​GTG​G	146 bp
		R: CAG​CCA​GTC​ATC​TTT​GGA​GAC​C	
PKCs	NM_006904.7	F: GCG​CCA​TAT​CTG​TCA​TCT​GCT​G	127 bp
		R: TTA​TAG​CGG​CGC​TTC​AGG​TCG​A	
ERK1	NM_001040056.3	F: TGG​CAA​GCA​CTA​CCT​GGA​TCA​G	116 bp
		R: GCA​GAG​ACT​GTA​GGT​AGT​TTC​GG	
ERK2	NM_002745.5	F: ACA​CCA​ACC​TCT​CGT​ACA​TCG​G	124 bp
		R: TGG​CAG​TAG​GTC​TGG​TGC​TCA​A	
Vimentin	NM_003380.5	F: AGG​CAA​AGC​AGG​AGT​CCA​CTG​A	100 bp
		R: ATC​TGG​CGT​TCC​AGG​GAC​TCA​T	
α-SMA	NM_001613.4	F: ACT​GCC​TTG​GTG​TGT​GAC​AA	224 bp
		R: TCC​CAG​TTG​GTG​ATG​ATG​CC	
ACTB	NM_001101.5	F: CTCGCCTTTGCCGATCC	298 bp
		R: TCT​CCA​TGT​CGT​CCC​AGT​TG	

**TABLE 2:** Mouse PCR primers.

Gene	GenBank Accession	Primer Sequences (5′–3′)	Product Length
AKT	NM_001382431.1	F: GGA​CTA​CTT​GCA​CTC​CGA​GAA​G	136 bp
		R: CAT​AGT​GGC​ACC​GTC​CTT​GAT​C	
mTOR	NM_001386500.1	F: AGA​AGG​GTC​TCC​AAG​GAC​GAC​T	159 bp
		R: GCA​GGA​CAC​AAA​GGC​AGC​ATT​G	
PKCs	NM_011159.2	F: ACT​TAC​CGT​GTC​GTG​CCA​AT	186 bp
		R: ATC​GCT​TTT​CCC​CGA​CAC​TT	
ERK1	NM_001109891.2	F: GGC​TTT​CTG​ACG​GAG​TAT​GTG​G	129 bp
		R: GTT​GGA​GAG​CAT​CTC​AGC​CAG​A	
ERK2	NM_001357115.1	F: CCC​CCA​GTT​CTT​TAC​CCT​GG	253 bp
		R: ATT​CAG​AAC​AGG​GAG​GAA​CCA​C	
Vimentin	NM_003380.5	F: CGG​AAA​GTG​GAA​TCC​TTG​CAG​G	138 bp
		R: AGC​AGT​GAG​GTC​AGG​CTT​GGA​A	
α-SMA	NM_001613.4	F: TGC​TGA​CAG​AGG​CAC​CAC​TGA​A	138 bp
		R: CAG​TTG​TAC​GTC​CAG​AGG​CAT​AG	
ACTB	NM_007393.5	F: GTT​GGA​GCA​AAC​ATC​CCC​CA	189 bp
		R: CGC​GAC​CAT​CCT​CCT​CTT​AG	

### Sirius Red Staining and Masson Staining

Mouse and human colon tissues were fixed using 10% formalin in phosphate-buffered saline (PBS), embedded in paraffin, cut into 4-μm-thick slices, and stained with Sirius red (G-CLONE, China) and Masson’s trichrome (Solarbio), according to standard methods. After sealing with a neutral gum, the stained slices were inspected using a light microscope (Olympus, Japan) to histologically assess the fibrosis of colon tissues.

### Immunohistochemistry

Paraffin-embedded human colon tissue slices (4 μm) were deparaffinized in xylene and rehydrated through a series of gradient ethanol and tap water. The rehydrated tissue slices were immersed in sodium citrate buffer (10 mm sodium citrate, pH 6.0) (Servicebio, China), microwaved for 5 min for antigen retrieval, and maintained in this solution for 15 min. After cooling with tap water, the tissue sections were soaked in 3% hydrogen peroxide (H_2_O_2_) (Dawen Biotec, China) for 15 min, blocked with 5% bovine serum albumin (BSA) at 37°C for 30 min, and incubated overnight at 4°C with rabbit anti-PKC (1:300, Immunoway) and rabbit anti-ERK1/2 (1:300, Immunoway) antibodies. After washing three times with PBS, the tissue sections were incubated with HRP-labeled anti-rabbit antibodies (1:500, Thermo Fisher Scientific) at 37°C for 1 h and reacted with DAB chromogenic solution (Biosharp, China). Finally, the slices were counterstained with hematoxylin, sealed with neutral balsam, and inspected under a light microscope.

### Immunofluorescence Assay

Paraffin-embedded mouse tissue samples were prepared as described for immunohistochemistry. Then, 4-μm tissue slices were incubated overnight at 4°C with rabbit anti-PKCs (1:300, Immunoway, United States) and rabbit anti-ERK1/2 (1:300, Immunoway) antibodies. After three washes with PBS, the tissue sections were incubated at room temperature for 1 h with Alexa Fluor® 488 F(ab')2-Goat IgG (1:300, Thermo Fisher Scientific, United States), stained with DAPI solution (Dawen Biotec) for 10 min, and sealed with an antifluorescence quenching sealing agent (Solarbio). The stained sections were viewed under a fluorescence microscope (LEICA, Germany).

### Statistical Analysis

The results were expressed as mean ± SEM. Two-group comparisons were performed using two-tailed t-tests, and multigroup comparisons were performed using one-way analysis of variance (ANOVA), with pairwise comparisons between multiple groups assessed by the LSD test. All statistical analyses were performed using the Statistical Package for the Social Sciences (SPSS) 21.0 (IBM Corp, Armonk, NY, United States). All *p* values were two-sided, with *p* <0.05 considered statistically significant. Digital images were quantified by ImageJ software (Bethesda, MD, United States).

## Results

### Intersection Genes of *Pulsatilla* Decoction and Crohn’s Disease

**FIGURE 1:**
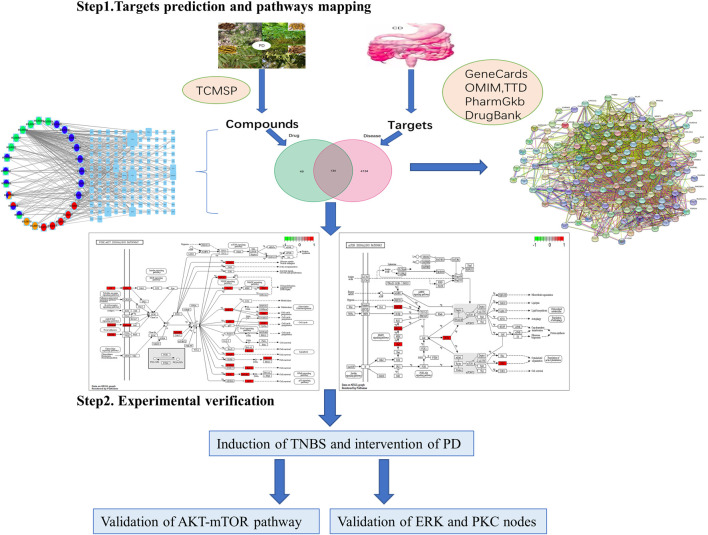
Network pharmacology framework of *Pulsatilla* decoction for treatment of Crohn’s disease.

**FIGURE 2:**
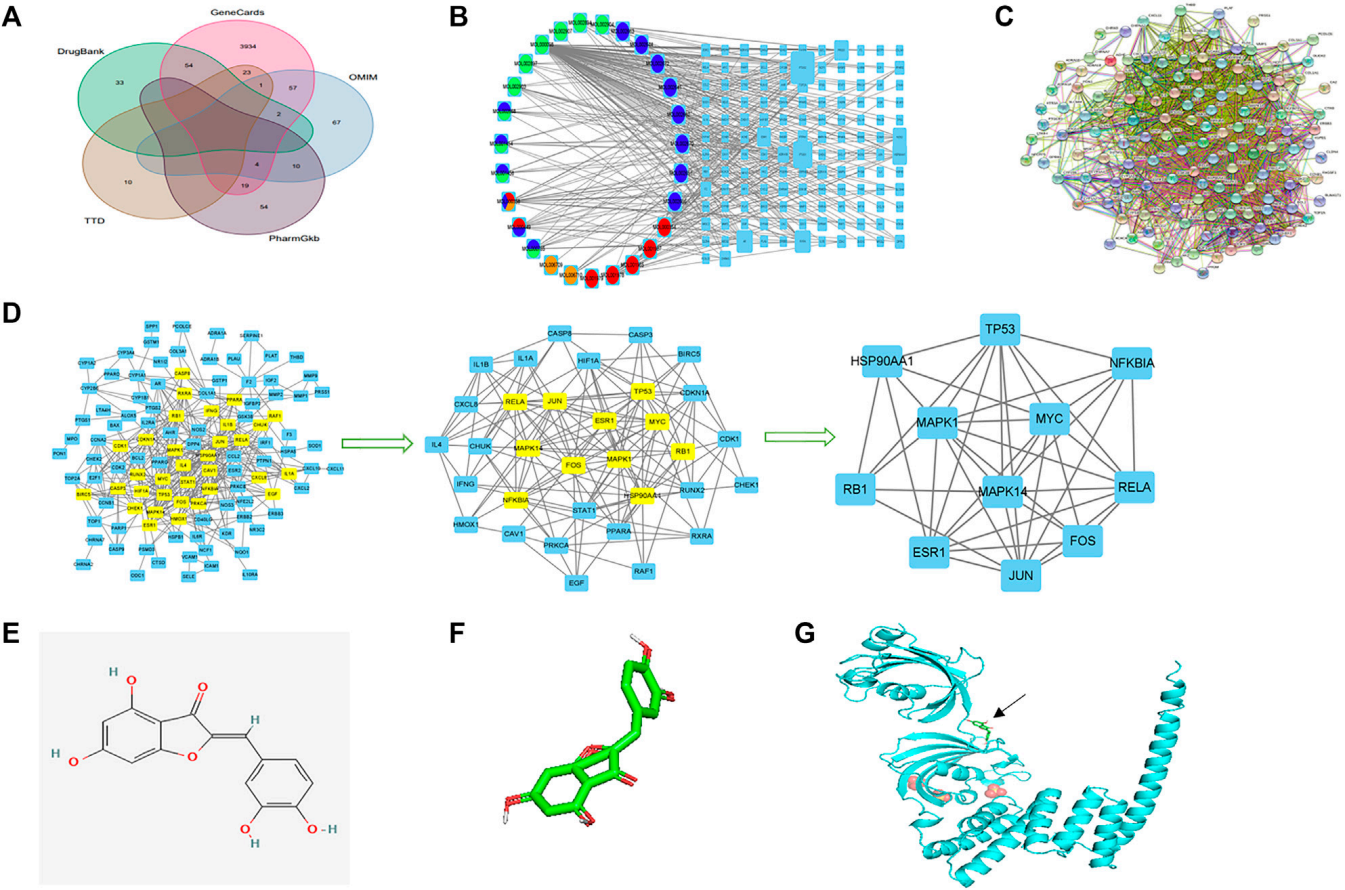
Network construction, PPI network core screening, and molecular docking simulation. **(A)** Venn diagram of the five databases surveyed. **(B)** Drug–compound–target–disease network. Red: *Pulsatilla chinensis*; blue: *Phellodendron chinense*; green: *Coptis chinensis*; yellow: *Cortex fraxini*. **(C)** PPI network. **(D)** Screening of the PPI network core. **(E)** 2D structure of aureusidin. **(F)** 3D conformer of aureusidin. **(G)** 3D conformer of HSP90AA1 and molecular docking. The black arrow indicates the position of molecular docking.

Based on the screening conditions, 53 compounds and 2,520 protein targets were identified in the four components of PD. These compounds were synthesized and their protein targets were converted to gene symbols (Supplementary Tables S1, S2). Concurrently, 4,268 CD-related genes were identified in the databases, following the removal of duplicate genes (Figure 2A; Supplementary Table S3). Finally, 134 intersection genes were identified (Figure 1; Supplementary Table S4).

### Drug–Compound–Target–Disease Network and PPI Network

The form prepared for assessing the drug–compound–target–disease network is shown in Supplementary Table S5. The network was exported from Cytoscape (Figure 2B), with the left circle indicating drugs and the right square indicating target genes. The size of gene nodes and number of edges indicated the degrees of correlation. PTGS1, PTGS2, PRSS1, RXRA, AR, HSP90AA1, ESR1, and NOS2 genes were identified as the key genes of the drug–compound–target–disease network.

The PPI network was drawn by the Online STRING database using the drug–disease intersection (Figure 2C). Proteins were represented by network nodes, with each node representing all the proteins produced by a single, protein-coding gene locus. Edges represented protein–protein associations but did not necessarily mean that these proteins were physically bound to each other. Moreover, a corresponding tabular text was downloaded to screen the PPI network core.

### PPI Network Core Screening and Molecular Docking Simulation

The first filtration identified 33 network nodes (Supplementary Table S6; Figure 2D). After duplicate filtering, 11 network nodes were obtained (Supplementary Tables S7, S8; Figure 2D). Because the scores of CytoNCA were in accordance with the overall nodes, each score of an individual node differed from the previous score. The FOS, MAPK1, RB1, HSP90AA1, ESR1, TP53, MAPK14, JUN, RELA, NFKBIA, and MYC were identified as the core of the PPI network. A comparison of the key genes of the drug–compound–target–disease and PPI networks showed that the HSP90AA1 gene was common to both and used to simulate molecular docking.

The 3D conformer of HSP90AA1 was used as the protein receptor for molecular docking, with aureusidin considered the small-molecule ligand for molecular docking (Figures 2E,F). The molecular docking of HSP90AA1 and aureusidin is shown in Figure 2G.

### GO and KEGG Enrichment Analyses

**FIGURE 3:**
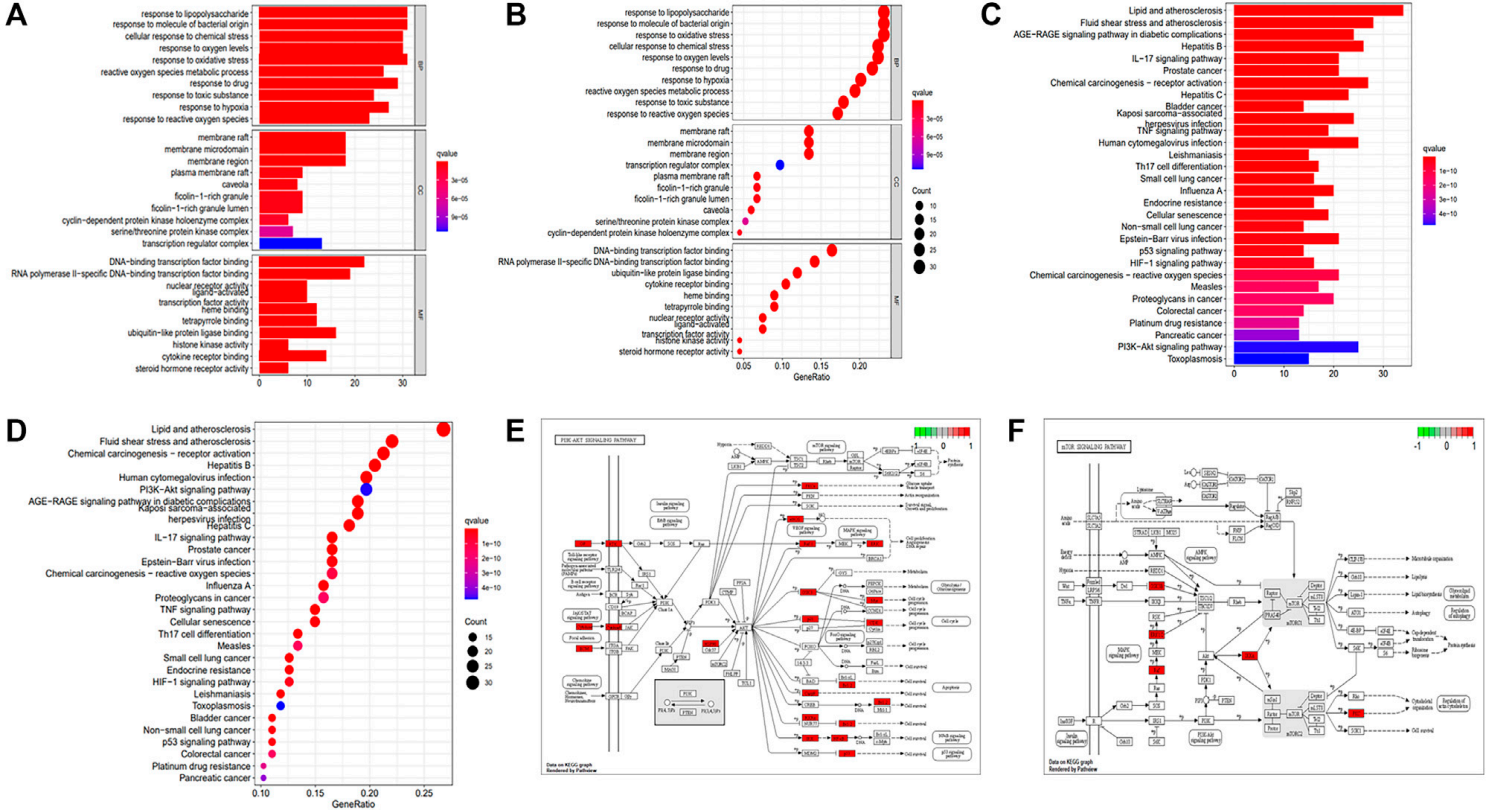
GO and KEGG enrichment analyses. **(A)** Barplot of GO annotation. **(B)** Bubble diagram of GO annotation. **(C)** Barplot of KEGG enrichment analysis. **(D)** Bubble diagram of KEGG enrichment analysis. **(E)** PI3K-AKT signaling pathway. **(F)** mTOR signaling pathway.

The GO enrichment analysis (Figures 3A,B) was performed to annotate gene functions, including biological processes (BPs), cellular components (CCs), and molecular functions (MFs). BP mainly included responses to lipopolysaccharide, a molecule of bacterial origin, and oxidative stress, both of which correlated significantly with CD fibrosis (Sartor, 1995; Hofmann et al., 2011; Alzoghaibi, 2013). CC was mainly associated with membrane rafts, microdomains, and regions, whereas MF was strongly associated with binding to the DNA-binding transcription factor, RNA polymerase II–specific DNA-binding transcription factor, and ubiquitin-like protein ligase.

KEGG enrichment analysis revealed that the key signaling pathways were mainly involved in lipid and atherosclerosis, fluid shear stress and atherosclerosis, chemical carcinogenesis receptor activation, hepatitis B, human cytomegalovirus infection, and the PI3K-AKT signaling pathway (Supplementary Table S9; Figures 3C,D), with the latter significantly associated with fibrotic diseases (Zhao et al., 2015; Tsoyi et al., 2018; Wei et al., 2018; Liu et al., 2021a). KEGG enrichment analysis also showed involvement of the mTOR signaling pathway, which acts downstream of the PI3K-AKT pathway (Zhao et al., 2015; Wei et al., 2018; Liu et al., 2021a). PKCs and ERK1/2 were nodes common to the P13K-AKT and mTOR signaling pathways (Figures 3E,F). These findings indicated that the key mechanism of PD in the treatment of CD was strongly associated with the AKT-mTOR pathway, suggesting that PD might have an antifibrotic effect in patients with CD.

### Imaging and Pathological Changes in Patients With CD

**FIGURE 4:**
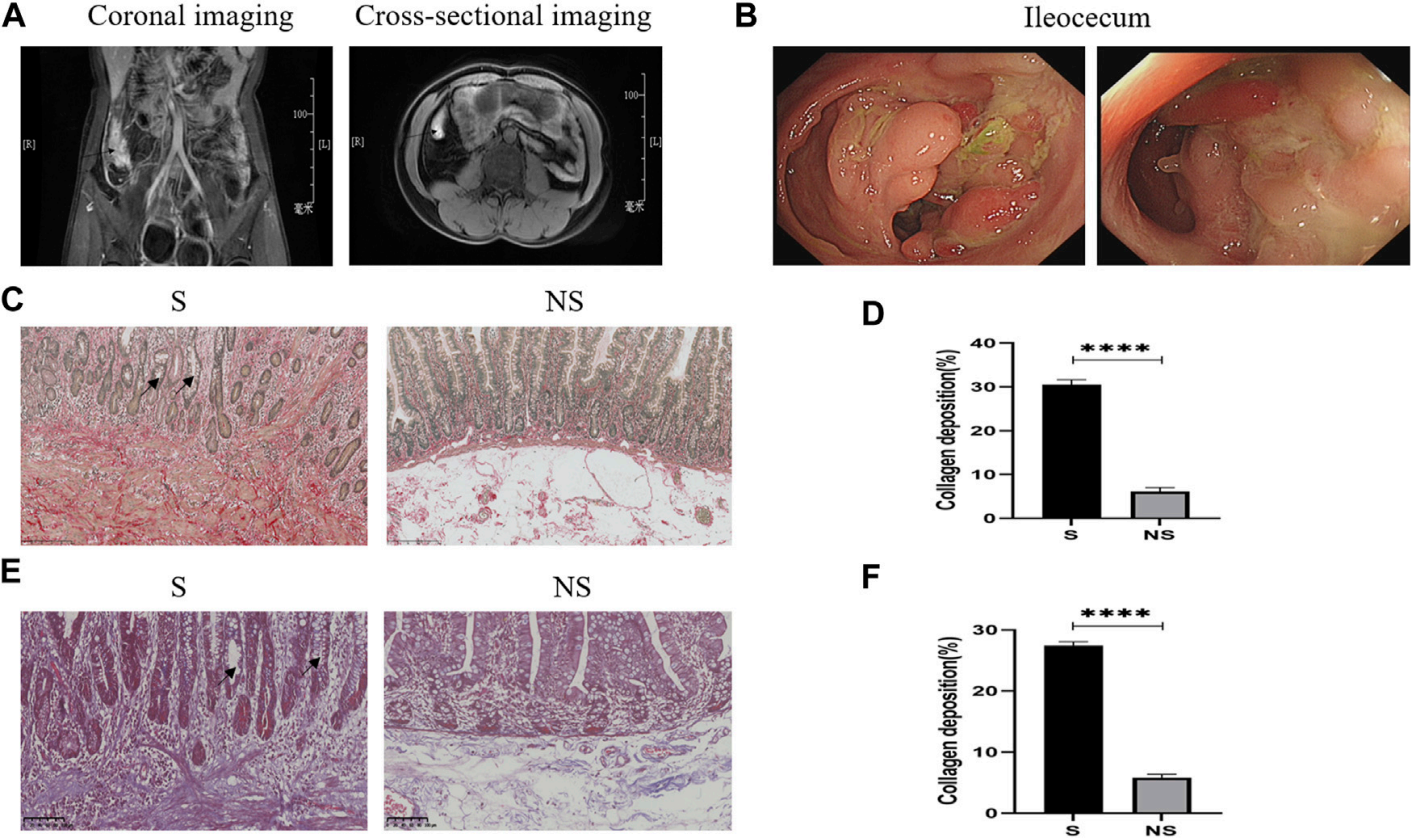
Clinical data of patients with CD. **(A)** Abdominal enhanced CT. Arrows: ileocecal high signal focus (coronal and cross-sectional imaging). **(B)** Colonoscopy: ileocecal stenoses. **(C)** Sirius red staining. Red represents collagen fibers and yellow indicates muscle fibers as determined by light microscopy. S, stenoses; NS, non-stenoses. Scale bar: 250 μm, magnification: ×200. Arrows: disordered arrangement of intestinal glands and deformation and reduction of goblet cells. **(D)** Sirius red staining of collagen deposition. **(E)** Masson’s staining. Blue indicates collagen fibers and red represents muscle fibers as determined by light microscopy. Scale bar: 100 μm, magnification: ×200. Arrows: disordered arrangement of intestinal glands and deformation and reduction of goblet cells. **(F)** Masson’s staining of collagen deposition. *p < 0.05, **p < 0.01, ***p < 0.001, and ****p < 0.0001.

Patients with CD underwent abdominal computed tomography (CT) or magnetic resonance imaging (MRI) and colonoscopic evaluation before surgery. Intestinal stenoses showed high signal enhancement on abdominal-enhanced CT (Figure 4A), with colonoscopy showing macroscopic stenoses caused by cobblestone hyperplasia (Figure 4B). Pathologic evaluation of postoperative colon specimens by Sirius red (Figures 4C,D) and Masson’s (Figures 4E,F) staining showed that collagen deposition was greater in areas of stenosis than nonstenosis. In addition, crypt structures were destroyed as shown by the disordered arrangement of intestinal glands and deformation and reduction of goblet cells.

### Expression of AKT/mTOR and PKCs/ERK1/2 in Areas of Stenosis and Nonstenosis in Patients With CD

**FIGURE 5:**
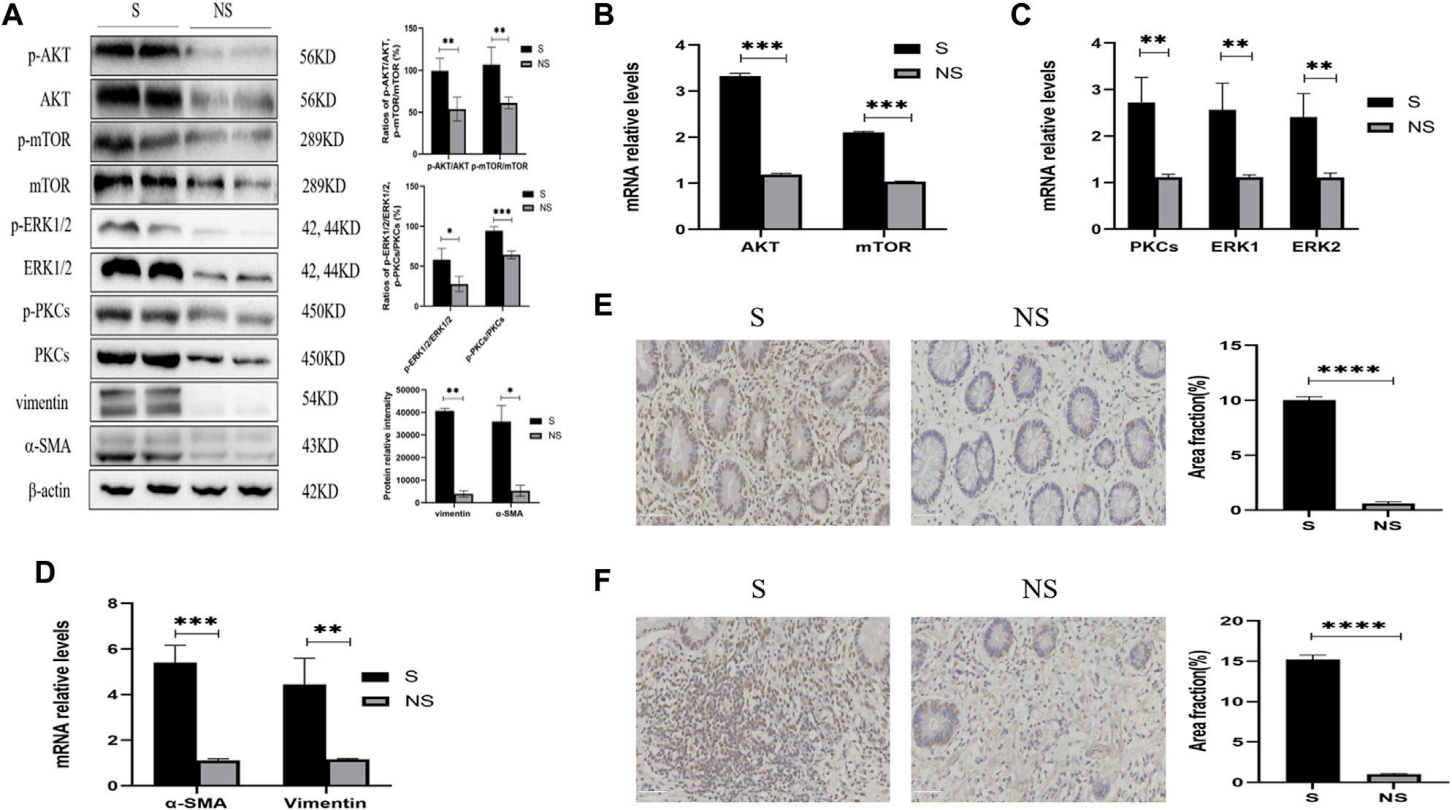
Expression of AKT/mTOR and PKCs/ERK1/2 in stenoses and nonstenoses of CD patients. **(A)** Western blotting showing the relative protein intensities of p-AKT, AKT, p-mTOR, mTOR, p-ERK1/2, ERK1/2, p-PKCs, PKCs, vimentin, and α-SMA. **(B–D)** Real-time quantitative qPCR showing the relative levels of **(B)** AKT and mTOR mRNAs, **(C)** PKCs and ERK1/2 mRNAs, and **(D)** vimentin and α-SMA mRNAs. **(E,F)** Immunohistochemistry of **(E)** PKCs and **(F)** ERK1/2. Scale bars: 50 μm, magnification: ×400. S, stenoses; NS, non-stenoses. *p < 0.05, **p < 0.01, ***p < 0.001, and ****p < 0.0001.

Western blotting of resected colonic specimens showed that the levels of p-AKT, AKT, p-mTOR, mTOR, p-PKC, PKC, p-ERK1/2, and ERK1/2 proteins were higher in areas of stenosis than in non-stenosis close to the surgical margins (Figure 5A). In addition, the ratios of phosphorylated to unphosphorylated proteins (p-AKT/AKT, p-mTOR/mTOR, p-PKCs/PKCs, and p-ERK1/2/ERK1/2) revealed higher values in strictures than nonstrictures (Figure 5A). These findings were consistent with the differences in expression of the fibrosis markers vimentin and α-SMA (Figure 5A), but interestingly, there were no significant differences between stenotic areas and surrounding tissue with similar degree of fibrosis (data not shown). Similarly, the relative levels of mRNAs encoded by these genes were higher in strictures than in nonstrictures (Figures 5B–D). Moreover, immunohistochemistry showed that the levels of PKCs and ERK1/2 were higher in strictures (Figures 5E,F). Taken together, these findings indicated that fibrosis in patients with CD was closely related with the expression of AKT, mTOR, PKCs, and ERK1/2.

### Weight and Colon Pathological Changes in Mice

**FIGURE 6:**
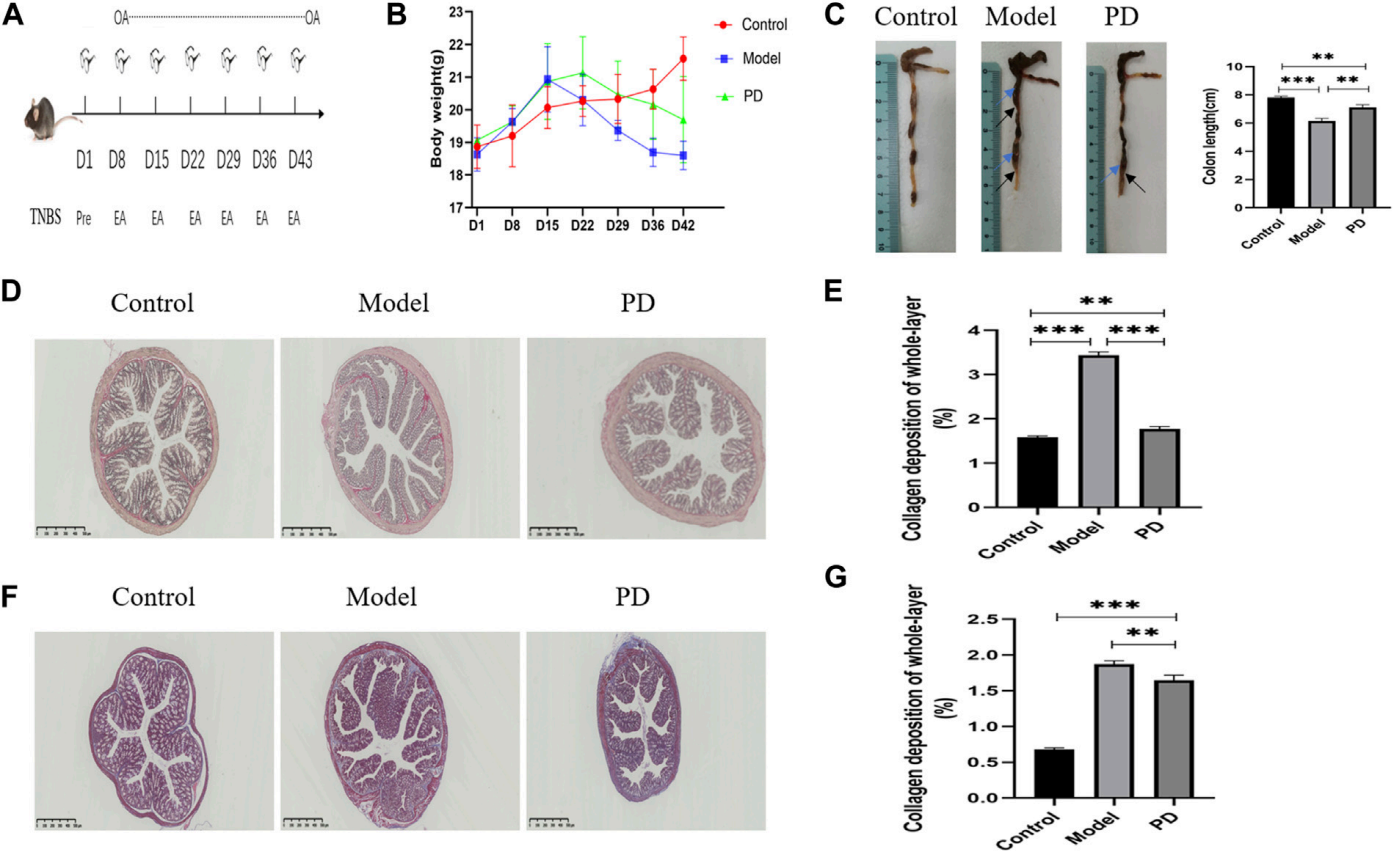
Assessment of the mouse colonic fibrosis model. **(A)** Schematic overview of the model. OA: oral administration; Pre: TNBS presensitization; EA: TNBS enteral administration. **(B)** Mouse weight change over time. **(C)** Mouse colon length over time. Blue arrows: local expansion and deformation of the intestinal cavity; black arrows: thickening of the intestinal walls and intestinal stenoses. **(D)** Sirius red staining of collagen fibers. PD: administration of *Pulsatilla* decoction. Scale bar: 500 μm, magnification: ×40. **(E)** Sirius red staining showing whole-layer collagen deposition. **(F)** Masson’s staining. Blue indicates collagen fibers. Scale bar: 500 μm, magnification: ×40. **(G)** Masson staining showing whole-layer collagen deposition. *p < 0.05, **p < 0.01, ***p < 0.001, and ****p < 0.0001.

Mice were killed after six cycles of TNBS and continuous oral administration of PD or saline (Figure 6A). Weight loss was lower (Figure 6B) and colons were longer (Figure 6C) in mice administered with PD. The mice in the model and PD groups showed local expansion and deformation of the intestinal cavity and thickening of the intestinal walls and intestinal stenoses (Figure 6C). Sirius red staining showed that collagen deposition was greater in mice treated with TNBS alone than in mice treated with TNBS and PD. Sirius staining in the control group resulted from the staining of vascular elastic fibers confined to the submucosa (Figure 6D). Quantification of collagen deposition showed that PD mitigated collagen deposition throughout the colon (Figure 6E). Moreover, Masson’s staining reduced the area of false-positive fibrosis (Figures 6F,G).

### PD Inhibits the Expression of AKT/mTOR and PKCs/ERK1/2 in a Mouse Model of Fibrosis

**FIGURE 7:**
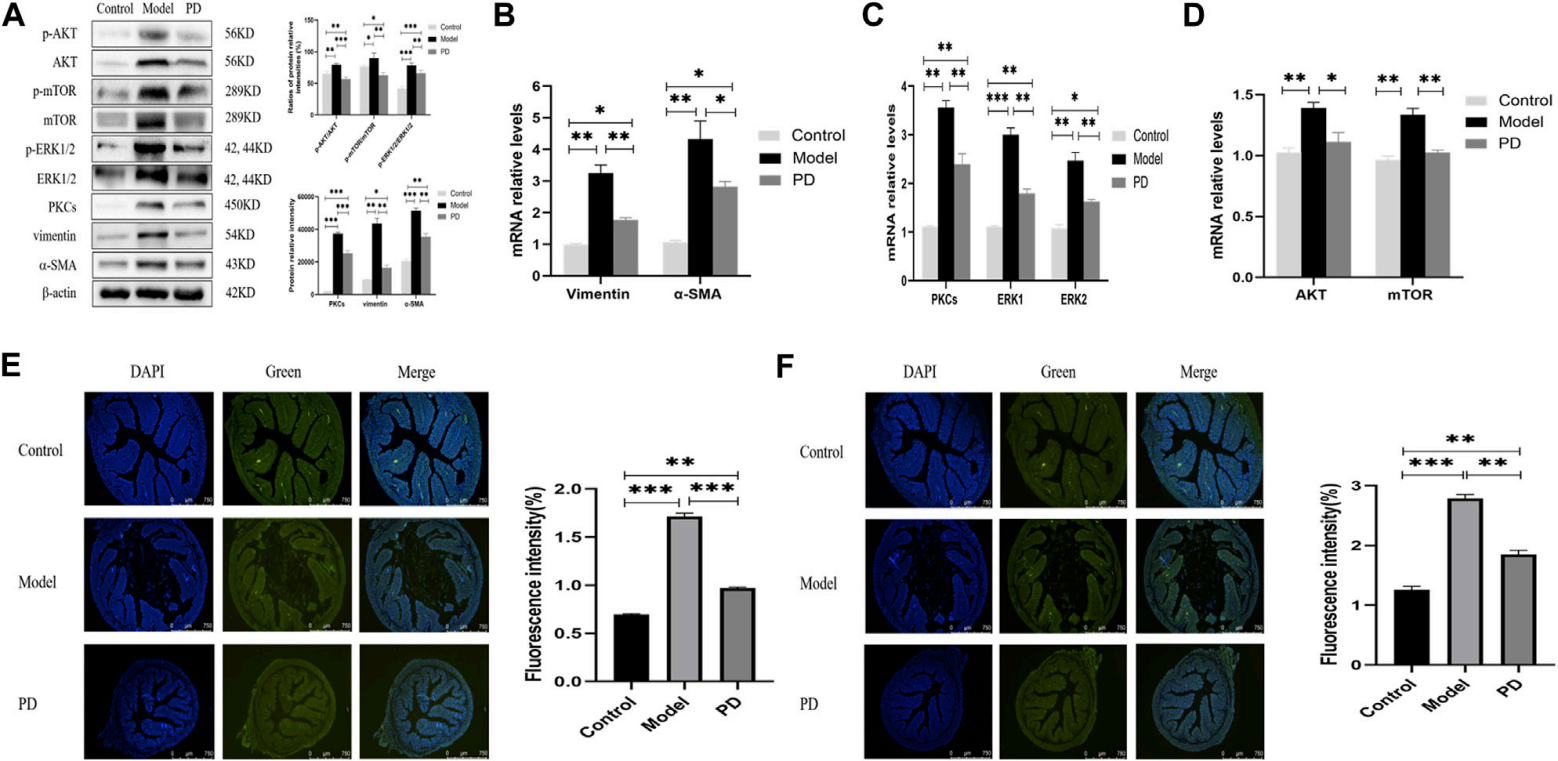
Expression of AKT/mTOR and PKCs/ERK1/2 in the mouse model of colonic fibrosis. **(A)** Western blotting showing the relative protein intensities of p-AKT, AKT, p-mTOR, mTOR, p-ERK1/2, ERK1/2, PKCs, vimentin, and α-SMA. **(B–D)** Real-time quantitative qPCR, showing the relative levels of **(B)** AKT and mTOR mRNAs, **(C)** PKCs and ERK1/2 mRNAs, and **(D)** vimentin and α-SMA mRNAs. **(E,F)** Immunofluorescence of **(E)** PKCs and **(F)** ERK1/2. Scale bars: 750 μm, magnification: ×50. PD: *Pulsatilla* decoction. *p < 0.05, **p < 0.01, ***p < 0.001, and ****p < 0.0001.

Western blotting showed that PD administration downregulated the levels of expression of p-AKT, AKT, p-mTOR, mTOR, PKCs, p-ERK1/2, and ERK1/2 in mice treated with TNBS and downregulated the levels of vimentin and α-SMA (Figure 7A). Moreover, the ratios of phosphorylated to unphosphorylated proteins (p-AKT/AKT, p-mTOR/mTOR, and p-ERK1/2/ERK1/2) revealed higher values in the model group and lower values in the PD group than those in the control group (Figure 7A). Similarly, PD administration downregulated the expression of AKT, mTOR, PKC, ERK1/2, vimentin, and α-SMA mRNAs in the colon tissue (Figures 7B–D). Immunofluorescence assays also found that the fluorescence intensities of PKCs and ERK1/2 were lower in mice treated with TNBS plus PD than in mice treated with TNBS plus saline but were higher in the control group (Figures 7E,F). These findings indicated that PD could inhibit intestinal fibrosis by reducing the levels of expression of AKT/mTOR and PKCs/ERK1/2.

## Discussion

Drug discovery usually involves the designing of maximally selective ligands that act on individual targets (Hopkins, 2008). Many complex diseases, however, involve multiple targets, making this the main reason for the failure of new drugs in clinical trials. Network pharmacology, which involves drug targets, disease targets, and their pharmacological relationships, can be used to systematically study the effects of drugs on complex diseases. For example, the drug–compound–target–disease network, which can evaluate the effects of drugs on macroscopic regulation, may be a new approach to study the mechanisms of action of TCMs and their compounds (Chen et al., 2014). In the present study, construction of the drug–compound–target–disease and PPI networks showed that the HSP90AA1 gene was the key intersecting gene between both. This gene was found to be associated with the glucocorticoid response and severity of fatigue in patients with CD (Grimstad et al., 2020; Skrzypczak-Zielinska et al., 2021). Molecular docking showed that the protein encoded by the HSP90AA1 gene bound to aureusidin, the bioactive compound of *P. chinensis*, which had various anti-inflammatory effects on inflammation-related diseases (Ren et al., 2020; Yang et al., 2020; Yang et al., 2021). Moreover, anemoside B4, a triterpenoid saponin extracted from *P. chinensis*, had also been found to ameliorate inflammation by regulating intestinal flora (Liu et al., 2021b) and pathways associated with NF-κB and MAPK (Ma et al., 2020; Zhang et al., 2021). In addition, dihydroberberine from *P. chinense*, as well as *C. chinensis*, was shown to alleviate ulcerative colitis by regulating the gut barrier function and immune-inflammatory response (Li et al., 2021).

GO and KEGG enrichment analyses indicated that PD may be effective against intestinal fibrosis in CD through the AKT-mTOR pathway, with PKCs and ERK1/2 being the common nodes. Furthermore, both human and animal experiments revealed that the key mechanism of PD against CD was strongly associated with its antifibrotic effect through the regulation of the AKT-mTOR pathway, especially PKCs and ERK1/2. Previous studies found that PD might play an important role in the treatment of colitis by inhibiting the expression of pro-inflammatory cytokines (Yu et al., 2011) and activating the NLRP3 inflammasome (Wang et al., 2012a). Esculin extracted from *C. fraxini* was found to alleviate inflammation *via* the pathways of PPARγ/NF-кB and AKT/GSK3β/NF-κB (Tian et al., 2019; Xu et al., 2021), with coptisine from *C. chinensis* regulating the PI3K/AKT pathway (Wu et al., 2019). Moreover, PD was recently shown to alleviate colitis by enhancing autophagy and regulating the PI3K-AKT-mTORC1 pathway (Wang et al., 2022). However, most of these studies focused on the anti-inflammatory effects of PD, suggesting the need to further study its antifibrotic mechanism.

Intestinal fibrosis characterized by lumen stenoses is a common complication of CD, with 30–50% of patients with CD developing intestinal fibrosis within 10 years after diagnosis (Rieder et al., 2013). Persistent chronic inflammation in CD results in progressive fibrosis, with excessive deposition of the extracellular matrix (ECM), a process mainly driven by mesenchymal cells, such as fibroblasts, myofibroblasts, and smooth muscle cells. Treatment with tryptase was found to activate intestinal fibroblasts through the PAR2-AKT-mTOR pathway and induce their differentiation into myofibroblasts (Liu et al., 2021a). The increase in mesenchymal cells and excessive secretion of ECM are considered key features of intestinal fibrotic strictures (Li et al., 2019). These alterations result from epithelial–mesenchymal transition (EMT), in which epithelial cells lose typical epithelial cell markers and acquire mesenchymal cell markers, along with the upregulation of collagens, α-SMA, vimentin, and fibronectin (Wang et al., 2021). A similar process in endothelial cells is called the endothelial–mesenchymal transition (EndoMT), during which endothelial cells lose typical endothelial markers and express fibroblast-like markers (Dejana et al., 2017). Suppression of the PI3K-AKT-mTOR signaling pathway was shown to prevent hepatic stellate cells from synthesizing a large amount of ECM (Wang et al., 2019b), and attenuation of AKT-mTOR-Yap pathway activation was found to inhibit fibroblast activation and ECM deposition (Bai et al., 2020). In the present study, we found that the expression of the AKT-mTOR pathway and PKCs and ERK1/2 nodes was significantly higher in areas with intestinal stenoses than those without intestinal stenoses in patients with CD, and this increased expression was inhibited by PD in mice treated with TNBS. Transforming growth factor (TGF)-beta was found to promote profibrotic behavior by serosal fibroblasts *via* PKC and ERK1/2 mitogen-activated protein kinase (MAPK) cell signaling (Mulsow et al., 2005). Moreover, advanced oxidation proteins induced EMT of intestinal epithelial cells *via* a PKC-mediated signaling pathway (Xu et al., 2017). Suppression of ERK1/2 MAPK reduced collagen expression (Monteleone et al., 2016), whereas activated ERK1/2 stimulated collagen proliferation and inhibited collagen degradation (Theiss et al., 2005). In addition, berberine from *C. chinensis* was shown to alleviate fibrosis by suppressing HIF-1 α activation (Hu et al., 2018). Furthermore, epiberberine extracted from *C. chinensis* and the water extract of *C. fraxini* were shown to regulate AKT and ERK pathways (Li et al., 2015b; Liu et al., 2020), with phellodendronoside A from *P. chinense* inhibiting the activation of ERK and p38MAPK (Si et al., 2021). Thus, targeting the AKT-mTOR signaling pathway, especially PKCs and ERK1/2, might be a potential therapeutic approach in the treatment of CD fibrosis.

KEGG enrichment analysis also showed involvement of signaling pathways associated with atherosclerosis. CD has been associated with an increased risk of atherosclerotic cardiovascular disease (ASCVD) (Kristensen et al., 2014a; Kristensen et al., 2014b; Singh et al., 2014; Bigeh et al., 2020), with the expression of homocysteine, which increases the risk of atherosclerosis and induces oxidative stress, being significantly higher in patients with CD than in controls (Aniwan et al., 2017). GO enrichment analysis also showed that oxidative stress was a critical biological process in CD. Oxidative stress might be a major factor contributing to the tissue injury and fibrosis of CD (Alzoghaibi, 2013; Pereira et al., 2015). In addition, berberine was found to inhibit fibrosis by reducing the peroxidative stress in the liver (Wang et al., 2012b). These findings indicate the need to monitor CD patients for the potential risk of ASCVD and suggest that PD treatment may alleviate atherosclerosis and oxidative stress in both CD and ASCVD.

This study had several limitations. For example, none of the colon specimens was obtained from patients treated preoperatively with PD. Prospective and retrospective studies that include larger numbers of samples are needed, as studies in patients treated with PD and the application of agonists or inhibitors will help further identify the specific key targets of PD against fibrosis at animal and cellular levels. Moreover, because PD is a mixture of many compounds, the proportions of these compounds should be analyzed by high-performance liquid chromatography (HPLC). In addition, atherosclerosis is a multifocal disease associated with lipid deposition (Falk, 2006), whereas CD is characterized by the accumulation of hypertrophic mesenteric adipose tissue around the fibrotic intestinal segment (Mao et al., 2019), with the latter playing an important role in intestinal fibrosis (Eder et al., 2019; Mao et al., 2019). Additional studies are needed to determine whether PD affects CD fibrosis by acting on the accumulation of adipose tissue.

## Conclusion

Network pharmacology analysis and *in vivo* experimental validation were performed to assess the pharmacological mechanisms of PD against CD. This network pharmacological analysis predicted that PD exerted its therapeutic effects in CD by regulating multiple ingredients, targets, and pathways. Evaluation of colon tissues from CD patients showed that intestinal fibrosis was greater in stenotic than nonstenotic regions and was accompanied by the upregulation of p-AKT, AKT, p-mTOR, mTOR, p-PKCs, PKCs, p-ERK1/2, and ERK1/2. Moreover, PD treatment of mice with TNBS-induced colonic fibrosis reduced the level of fibrosis and downregulated the expression of p-AKT, AKT, p-mTOR, mTOR, PKCs, p-ERK1/2, and ERK1/2. Taken together, these findings indicate that the method described in this study provides an optimized approach to elucidate the pharmacological mechanisms of PD and identify new targets for the treatment of CD fibrosis.

## Data Availability

The datasets presented in this study can be found in online repositories. The names of the repository/repositories and accession number(s) can be found in the article/Supplementary Material.
