# Supplementary material for: Network Pharmacology-Based Strategy to Identify the Pharmacological Mechanisms of Pulsatilla Decoction against Crohn’s Disease
Source: Front Pharmacol. 2022 Apr 5;13:844685. doi: 10.3389/fphar.2022.844685 (PMC9016333; doi:10.3389/fphar.2022.844685)
Supplement: Supplementary file 1 [file DataSheet1.zip › Table (1).DOCX]

| **Supplemental Table 1. Drug-compound-protein targets from TCMSP database** | | | |
| --- | --- | --- | --- |
| Drug | MolId | MolName | Target |
| Pulsatilla chinensis | MOL001973 | Sitosteryl acetate | Progesterone receptor |
| Pulsatilla chinensis | MOL001978 | Aureusidin | Nitric oxide synthase, inducible |
| Pulsatilla chinensis | MOL001978 | Aureusidin | Prostaglandin G/H synthase 1 |
| Pulsatilla chinensis | MOL001978 | Aureusidin | Estrogen receptor |
| Pulsatilla chinensis | MOL001978 | Aureusidin | Androgen receptor |
| Pulsatilla chinensis | MOL001978 | Aureusidin | Peroxisome proliferator-activated receptor gamma |
| Pulsatilla chinensis | MOL001978 | Aureusidin | Prostaglandin G/H synthase 2 |
| Pulsatilla chinensis | MOL001978 | Aureusidin | Carbonic anhydrase 2 |
| Pulsatilla chinensis | MOL001978 | Aureusidin | Estrogen receptor beta |
| Pulsatilla chinensis | MOL001978 | Aureusidin | Mitogen-activated protein kinase 14 |
| Pulsatilla chinensis | MOL001978 | Aureusidin | Glycogen synthase kinase-3 beta |
| Pulsatilla chinensis | MOL001978 | Aureusidin | Heat shock protein HSP 90-alpha |
| Pulsatilla chinensis | MOL001978 | Aureusidin | Cell division protein kinase 2 |
| Pulsatilla chinensis | MOL001978 | Aureusidin | Phosphatidylinositol-4,5-bisphosphate 3-kinase catalytic subunit gamma isoform |
| Pulsatilla chinensis | MOL001978 | Aureusidin | cAMP-dependent protein kinase catalytic subunit alpha |
| Pulsatilla chinensis | MOL001978 | Aureusidin | Trypsin-1 |
| Pulsatilla chinensis | MOL001978 | Aureusidin | Proto-oncogene serine/threonine-protein kinase Pim-1 |
| Pulsatilla chinensis | MOL001978 | Aureusidin | Cyclin-A2 |
| Pulsatilla chinensis | MOL001979 | LAN | Progesterone receptor |
| Pulsatilla chinensis | MOL001979 | LAN | Mineralocorticoid receptor |
| Pulsatilla chinensis | MOL001979 | LAN | Nuclear receptor coactivator 2 |
| Pulsatilla chinensis | MOL001985 | ZINC01615307 | Prostaglandin G/H synthase 1 |
| Pulsatilla chinensis | MOL001985 | ZINC01615307 | Potassium voltage-gated channel subfamily H member 2 |
| Pulsatilla chinensis | MOL001985 | ZINC01615307 | Coagulation factor X |
| Pulsatilla chinensis | MOL001985 | ZINC01615307 | Prostaglandin G/H synthase 2 |
| Pulsatilla chinensis | MOL001985 | ZINC01615307 | Vascular endothelial growth factor receptor 2 |
| Pulsatilla chinensis | MOL001985 | ZINC01615307 | Tyrosine-protein phosphatase non-receptor type 1 |
| Pulsatilla chinensis | MOL001985 | ZINC01615307 | DNA topoisomerase 2-alpha |
| Pulsatilla chinensis | MOL001985 | ZINC01615307 | Heat shock protein HSP 90-alpha |
| Pulsatilla chinensis | MOL001985 | ZINC01615307 | Nuclear receptor coactivator 2 |
| Pulsatilla chinensis | MOL001985 | ZINC01615307 | Calcium-activated potassium channel subunit alpha 1 |
| Pulsatilla chinensis | MOL001985 | ZINC01615307 | Calmodulin |
| Pulsatilla chinensis | MOL001987 | β-sitosterol | DNA topoisomerase 2-alpha |
| Pulsatilla chinensis | MOL000211 | Mairin | Progesterone receptor |
| Pulsatilla chinensis | MOL000354 | isorhamnetin | Nitric oxide synthase, inducible |
| Pulsatilla chinensis | MOL000354 | isorhamnetin | Prostaglandin G/H synthase 1 |
| Pulsatilla chinensis | MOL000354 | isorhamnetin | Estrogen receptor |
| Pulsatilla chinensis | MOL000354 | isorhamnetin | Androgen receptor |
| Pulsatilla chinensis | MOL000354 | isorhamnetin | Peroxisome proliferator-activated receptor gamma |
| Pulsatilla chinensis | MOL000354 | isorhamnetin | Prostaglandin G/H synthase 2 |
| Pulsatilla chinensis | MOL000354 | isorhamnetin | Tyrosine-protein phosphatase non-receptor type 1 |
| Pulsatilla chinensis | MOL000354 | isorhamnetin | Estrogen receptor beta |
| Pulsatilla chinensis | MOL000354 | isorhamnetin | Dipeptidyl peptidase 4 |
| Pulsatilla chinensis | MOL000354 | isorhamnetin | Mitogen-activated protein kinase 14 |
| Pulsatilla chinensis | MOL000354 | isorhamnetin | Glycogen synthase kinase-3 beta |
| Pulsatilla chinensis | MOL000354 | isorhamnetin | Heat shock protein HSP 90-alpha |
| Pulsatilla chinensis | MOL000354 | isorhamnetin | Cell division protein kinase 2 |
| Pulsatilla chinensis | MOL000354 | isorhamnetin | Phosphatidylinositol-4,5-bisphosphate 3-kinase catalytic subunit gamma isoform |
| Pulsatilla chinensis | MOL000354 | isorhamnetin | cAMP-dependent protein kinase catalytic subunit alpha |
| Pulsatilla chinensis | MOL000354 | isorhamnetin | Trypsin-1 |
| Pulsatilla chinensis | MOL000354 | isorhamnetin | Proto-oncogene serine/threonine-protein kinase Pim-1 |
| Pulsatilla chinensis | MOL000354 | isorhamnetin | Cyclin-A2 |
| Pulsatilla chinensis | MOL000354 | isorhamnetin | Nuclear receptor coactivator 2 |
| Pulsatilla chinensis | MOL000354 | isorhamnetin | Calmodulin |
| Pulsatilla chinensis | MOL000354 | isorhamnetin | Glycogen phosphorylase, muscle form |
| Pulsatilla chinensis | MOL000354 | isorhamnetin | Peroxisome proliferator-activated receptor delta |
| Pulsatilla chinensis | MOL000354 | isorhamnetin | Serine/threonine-protein kinase Chk1 |
| Pulsatilla chinensis | MOL000354 | isorhamnetin | Aldose reductase |
| Pulsatilla chinensis | MOL000354 | isorhamnetin | Nuclear receptor coactivator 1 |
| Pulsatilla chinensis | MOL000354 | isorhamnetin | Coagulation factor VII |
| Pulsatilla chinensis | MOL000354 | isorhamnetin | Prothrombin |
| Pulsatilla chinensis | MOL000354 | isorhamnetin | Nitric-oxide synthase, endothelial |
| Pulsatilla chinensis | MOL000354 | isorhamnetin | Acetylcholinesterase |
| Pulsatilla chinensis | MOL000354 | isorhamnetin | Gamma-aminobutyric-acid receptor subunit alpha-1 |
| Pulsatilla chinensis | MOL000354 | isorhamnetin | Amine oxidase [flavin-containing] B |
| Pulsatilla chinensis | MOL000354 | isorhamnetin | Glutamate receptor 2 |
| Pulsatilla chinensis | MOL000354 | isorhamnetin | Cytochrome P450-cam |
| Pulsatilla chinensis | MOL000354 | isorhamnetin | Transcription factor p65 |
| Pulsatilla chinensis | MOL000354 | isorhamnetin | Xanthine dehydrogenase/oxidase |
| Pulsatilla chinensis | MOL000354 | isorhamnetin | Neutrophil cytosol factor 1 |
| Pulsatilla chinensis | MOL000354 | isorhamnetin | Oxidized low-density lipoprotein receptor 1 |
| Pulsatilla chinensis | MOL000358 | beta-sitosterol | Progesterone receptor |
| Pulsatilla chinensis | MOL000358 | beta-sitosterol | Nuclear receptor coactivator 2 |
| Pulsatilla chinensis | MOL000358 | beta-sitosterol | Prostaglandin G/H synthase 1 |
| Pulsatilla chinensis | MOL000358 | beta-sitosterol | Prostaglandin G/H synthase 2 |
| Pulsatilla chinensis | MOL000358 | beta-sitosterol | Heat shock protein HSP 90-alpha |
| Pulsatilla chinensis | MOL000358 | beta-sitosterol | Phosphatidylinositol-4,5-bisphosphate 3-kinase catalytic subunit gamma isoform |
| Pulsatilla chinensis | MOL000358 | beta-sitosterol | Potassium voltage-gated channel subfamily H member 2 |
| Pulsatilla chinensis | MOL000358 | beta-sitosterol | cAMP-dependent protein kinase catalytic subunit alpha |
| Pulsatilla chinensis | MOL000358 | beta-sitosterol | D(1A) dopamine receptor |
| Pulsatilla chinensis | MOL000358 | beta-sitosterol | Muscarinic acetylcholine receptor M3 |
| Pulsatilla chinensis | MOL000358 | beta-sitosterol | Muscarinic acetylcholine receptor M1 |
| Pulsatilla chinensis | MOL000358 | beta-sitosterol | Sodium channel protein type 5 subunit alpha |
| Pulsatilla chinensis | MOL000358 | beta-sitosterol | Gamma-aminobutyric-acid receptor subunit alpha-2 |
| Pulsatilla chinensis | MOL000358 | beta-sitosterol | Muscarinic acetylcholine receptor M4 |
| Pulsatilla chinensis | MOL000358 | beta-sitosterol | cGMP-inhibited 3',5'-cyclic phosphodiesterase A |
| Pulsatilla chinensis | MOL000358 | beta-sitosterol | 5-hydroxytryptamine 2A receptor |
| Pulsatilla chinensis | MOL000358 | beta-sitosterol | Gamma-aminobutyric-acid receptor subunit alpha-5 |
| Pulsatilla chinensis | MOL000358 | beta-sitosterol | Alpha-1A adrenergic receptor |
| Pulsatilla chinensis | MOL000358 | beta-sitosterol | Gamma-aminobutyric-acid receptor subunit alpha-3 |
| Pulsatilla chinensis | MOL000358 | beta-sitosterol | Muscarinic acetylcholine receptor M2 |
| Pulsatilla chinensis | MOL000358 | beta-sitosterol | Alpha-1B adrenergic receptor |
| Pulsatilla chinensis | MOL000358 | beta-sitosterol | Beta-2 adrenergic receptor |
| Pulsatilla chinensis | MOL000358 | beta-sitosterol | Neuronal acetylcholine receptor subunit alpha-2 |
| Pulsatilla chinensis | MOL000358 | beta-sitosterol | Sodium-dependent serotonin transporter |
| Pulsatilla chinensis | MOL000358 | beta-sitosterol | Mu-type opioid receptor |
| Pulsatilla chinensis | MOL000358 | beta-sitosterol | Gamma-aminobutyric-acid receptor subunit alpha-1 |
| Pulsatilla chinensis | MOL000358 | beta-sitosterol | Neuronal acetylcholine receptor subunit alpha-7 |
| Pulsatilla chinensis | MOL000358 | beta-sitosterol | Cytochrome P450-cam |
| Pulsatilla chinensis | MOL000358 | beta-sitosterol | Apoptosis regulator Bcl-2 |
| Pulsatilla chinensis | MOL000358 | beta-sitosterol | Apoptosis regulator BAX |
| Pulsatilla chinensis | MOL000358 | beta-sitosterol | Caspase-9 |
| Pulsatilla chinensis | MOL000358 | beta-sitosterol | Transcription factor AP-1 |
| Pulsatilla chinensis | MOL000358 | beta-sitosterol | Caspase-3 |
| Pulsatilla chinensis | MOL000358 | beta-sitosterol | Caspase-8 |
| Pulsatilla chinensis | MOL000358 | beta-sitosterol | Protein kinase C alpha type |
| Pulsatilla chinensis | MOL000358 | beta-sitosterol | Transforming growth factor beta-1 |
| Pulsatilla chinensis | MOL000358 | beta-sitosterol | Serum paraoxonase/arylesterase 1 |
| Pulsatilla chinensis | MOL000358 | beta-sitosterol | Microtubule-associated protein 2 |
| Pulsatilla chinensis | MOL000449 | Stigmasterol | Progesterone receptor |
| Pulsatilla chinensis | MOL000449 | Stigmasterol | Mineralocorticoid receptor |
| Pulsatilla chinensis | MOL000449 | Stigmasterol | Nuclear receptor coactivator 2 |
| Pulsatilla chinensis | MOL000449 | Stigmasterol | null |
| Pulsatilla chinensis | MOL000449 | Stigmasterol | Ig gamma-1 chain C region |
| Pulsatilla chinensis | MOL000449 | Stigmasterol | Retinoic acid receptor RXR-alpha |
| Pulsatilla chinensis | MOL000449 | Stigmasterol | Nuclear receptor coactivator 1 |
| Pulsatilla chinensis | MOL000449 | Stigmasterol | Prostaglandin G/H synthase 1 |
| Pulsatilla chinensis | MOL000449 | Stigmasterol | Prostaglandin G/H synthase 2 |
| Pulsatilla chinensis | MOL000449 | Stigmasterol | Alpha-2A adrenergic receptor |
| Pulsatilla chinensis | MOL000449 | Stigmasterol | Sodium-dependent noradrenaline transporter |
| Pulsatilla chinensis | MOL000449 | Stigmasterol | Sodium-dependent dopamine transporter |
| Pulsatilla chinensis | MOL000449 | Stigmasterol | Beta-2 adrenergic receptor |
| Pulsatilla chinensis | MOL000449 | Stigmasterol | Aldose reductase |
| Pulsatilla chinensis | MOL000449 | Stigmasterol | Urokinase-type plasminogen activator |
| Pulsatilla chinensis | MOL000449 | Stigmasterol | Leukotriene A-4 hydrolase |
| Pulsatilla chinensis | MOL000449 | Stigmasterol | Amine oxidase [flavin-containing] B |
| Pulsatilla chinensis | MOL000449 | Stigmasterol | Amine oxidase [flavin-containing] A |
| Pulsatilla chinensis | MOL000449 | Stigmasterol | cAMP-dependent protein kinase catalytic subunit alpha |
| Pulsatilla chinensis | MOL000449 | Stigmasterol | Chymotrypsinogen B |
| Pulsatilla chinensis | MOL000449 | Stigmasterol | Muscarinic acetylcholine receptor M3 |
| Pulsatilla chinensis | MOL000449 | Stigmasterol | Muscarinic acetylcholine receptor M1 |
| Pulsatilla chinensis | MOL000449 | Stigmasterol | Beta-1 adrenergic receptor |
| Pulsatilla chinensis | MOL000449 | Stigmasterol | Sodium channel protein type 5 subunit alpha |
| Pulsatilla chinensis | MOL000449 | Stigmasterol | 5-hydroxytryptamine 2A receptor |
| Pulsatilla chinensis | MOL000449 | Stigmasterol | Alpha-1A adrenergic receptor |
| Pulsatilla chinensis | MOL000449 | Stigmasterol | Gamma-aminobutyric-acid receptor subunit alpha-3 |
| Pulsatilla chinensis | MOL000449 | Stigmasterol | Muscarinic acetylcholine receptor M2 |
| Pulsatilla chinensis | MOL000449 | Stigmasterol | Alpha-1B adrenergic receptor |
| Pulsatilla chinensis | MOL000449 | Stigmasterol | Gamma-aminobutyric-acid receptor subunit alpha-1 |
| Pulsatilla chinensis | MOL000449 | Stigmasterol | Neuronal acetylcholine receptor subunit alpha-7 |
| Phellodendron chinense | MOL001454 | berberine | Nitric oxide synthase, inducible |
| Phellodendron chinense | MOL001454 | berberine | Prostaglandin G/H synthase 1 |
| Phellodendron chinense | MOL001454 | berberine | Potassium voltage-gated channel subfamily H member 2 |
| Phellodendron chinense | MOL001454 | berberine | Estrogen receptor |
| Phellodendron chinense | MOL001454 | berberine | Androgen receptor |
| Phellodendron chinense | MOL001454 | berberine | Sodium channel protein type 5 subunit alpha |
| Phellodendron chinense | MOL001454 | berberine | Coagulation factor X |
| Phellodendron chinense | MOL001454 | berberine | Prostaglandin G/H synthase 2 |
| Phellodendron chinense | MOL001454 | berberine | Nitric-oxide synthase, endothelial |
| Phellodendron chinense | MOL001454 | berberine | Retinoic acid receptor RXR-alpha |
| Phellodendron chinense | MOL001454 | berberine | Beta-2 adrenergic receptor |
| Phellodendron chinense | MOL001454 | berberine | Heat shock protein HSP 90-alpha |
| Phellodendron chinense | MOL001454 | berberine | cAMP-dependent protein kinase catalytic subunit alpha |
| Phellodendron chinense | MOL001454 | berberine | Trypsin-1 |
| Phellodendron chinense | MOL001454 | berberine | Nuclear receptor coactivator 2 |
| Phellodendron chinense | MOL001454 | berberine | cAMP and cAMP-inhibited cGMP 3',5'-cyclic phosphodiesterase 10A |
| Phellodendron chinense | MOL001454 | berberine | Calmodulin |
| Phellodendron chinense | MOL001458 | coptisine | Nitric oxide synthase, inducible |
| Phellodendron chinense | MOL001458 | coptisine | Prostaglandin G/H synthase 1 |
| Phellodendron chinense | MOL001458 | coptisine | Potassium voltage-gated channel subfamily H member 2 |
| Phellodendron chinense | MOL001458 | coptisine | Estrogen receptor |
| Phellodendron chinense | MOL001458 | coptisine | Androgen receptor |
| Phellodendron chinense | MOL001458 | coptisine | Sodium channel protein type 5 subunit alpha |
| Phellodendron chinense | MOL001458 | coptisine | Prostaglandin G/H synthase 2 |
| Phellodendron chinense | MOL001458 | coptisine | Nitric-oxide synthase, endothelial |
| Phellodendron chinense | MOL001458 | coptisine | Trypsin-1 |
| Phellodendron chinense | MOL002641 | Phellavin_qt | Prostaglandin G/H synthase 2 |
| Phellodendron chinense | MOL002641 | Phellavin_qt | Coagulation factor VII |
| Phellodendron chinense | MOL002641 | Phellavin_qt | Heat shock protein HSP 90-alpha |
| Phellodendron chinense | MOL002643 | delta 7-stigmastenol | Progesterone receptor |
| Phellodendron chinense | MOL002644 | Phellopterin | Prothrombin |
| Phellodendron chinense | MOL002644 | Phellopterin | Muscarinic acetylcholine receptor M1 |
| Phellodendron chinense | MOL002644 | Phellopterin | Sodium channel protein type 5 subunit alpha |
| Phellodendron chinense | MOL002644 | Phellopterin | Prostaglandin G/H synthase 2 |
| Phellodendron chinense | MOL002644 | Phellopterin | Retinoic acid receptor RXR-alpha |
| Phellodendron chinense | MOL002644 | Phellopterin | Alpha-1B adrenergic receptor |
| Phellodendron chinense | MOL002644 | Phellopterin | Tyrosine-protein phosphatase non-receptor type 1 |
| Phellodendron chinense | MOL002644 | Phellopterin | Beta-2 adrenergic receptor |
| Phellodendron chinense | MOL002644 | Phellopterin | Gamma-aminobutyric-acid receptor subunit alpha-1 |
| Phellodendron chinense | MOL002644 | Phellopterin | Dipeptidyl peptidase 4 |
| Phellodendron chinense | MOL002644 | Phellopterin | Heat shock protein HSP 90-alpha |
| Phellodendron chinense | MOL002644 | Phellopterin | Neuronal acetylcholine receptor subunit alpha-7 |
| Phellodendron chinense | MOL002651 | Dehydrotanshinone II A | D(1A) dopamine receptor |
| Phellodendron chinense | MOL002651 | Dehydrotanshinone II A | Muscarinic acetylcholine receptor M3 |
| Phellodendron chinense | MOL002651 | Dehydrotanshinone II A | Prothrombin |
| Phellodendron chinense | MOL002651 | Dehydrotanshinone II A | Muscarinic acetylcholine receptor M1 |
| Phellodendron chinense | MOL002651 | Dehydrotanshinone II A | Estrogen receptor |
| Phellodendron chinense | MOL002651 | Dehydrotanshinone II A | Androgen receptor |
| Phellodendron chinense | MOL002651 | Dehydrotanshinone II A | Sodium channel protein type 5 subunit alpha |
| Phellodendron chinense | MOL002651 | Dehydrotanshinone II A | Peroxisome proliferator-activated receptor gamma |
| Phellodendron chinense | MOL002651 | Dehydrotanshinone II A | Muscarinic acetylcholine receptor M5 |
| Phellodendron chinense | MOL002651 | Dehydrotanshinone II A | Prostaglandin G/H synthase 2 |
| Phellodendron chinense | MOL002651 | Dehydrotanshinone II A | Muscarinic acetylcholine receptor M4 |
| Phellodendron chinense | MOL002651 | Dehydrotanshinone II A | Delta-type opioid receptor |
| Phellodendron chinense | MOL002651 | Dehydrotanshinone II A | Acetylcholinesterase |
| Phellodendron chinense | MOL002651 | Dehydrotanshinone II A | 5-hydroxytryptamine 2A receptor |
| Phellodendron chinense | MOL002651 | Dehydrotanshinone II A | Alpha-1A adrenergic receptor |
| Phellodendron chinense | MOL002651 | Dehydrotanshinone II A | Beta-2 adrenergic receptor |
| Phellodendron chinense | MOL002651 | Dehydrotanshinone II A | Mu-type opioid receptor |
| Phellodendron chinense | MOL002651 | Dehydrotanshinone II A | Gamma-aminobutyric-acid receptor subunit alpha-1 |
| Phellodendron chinense | MOL002651 | Dehydrotanshinone II A | Dipeptidyl peptidase 4 |
| Phellodendron chinense | MOL002651 | Dehydrotanshinone II A | Neuronal acetylcholine receptor subunit alpha-7 |
| Phellodendron chinense | MOL002651 | Dehydrotanshinone II A | Nuclear receptor coactivator 1 |
| Phellodendron chinense | MOL002662 | rutaecarpine | Prostaglandin G/H synthase 1 |
| Phellodendron chinense | MOL002662 | rutaecarpine | Androgen receptor |
| Phellodendron chinense | MOL002662 | rutaecarpine | Sodium channel protein type 5 subunit alpha |
| Phellodendron chinense | MOL002662 | rutaecarpine | Coagulation factor X |
| Phellodendron chinense | MOL002662 | rutaecarpine | Prostaglandin G/H synthase 2 |
| Phellodendron chinense | MOL002662 | rutaecarpine | Nitric-oxide synthase, endothelial |
| Phellodendron chinense | MOL002662 | rutaecarpine | 5-hydroxytryptamine 3 receptor |
| Phellodendron chinense | MOL002662 | rutaecarpine | Retinoic acid receptor RXR-alpha |
| Phellodendron chinense | MOL002662 | rutaecarpine | Phosphatidylinositol-4,5-bisphosphate 3-kinase catalytic subunit gamma isoform |
| Phellodendron chinense | MOL002662 | rutaecarpine | Serine/threonine-protein kinase Chk1 |
| Phellodendron chinense | MOL002662 | rutaecarpine | cAMP-dependent protein kinase catalytic subunit alpha |
| Phellodendron chinense | MOL002662 | rutaecarpine | 72 kDa type IV collagenase |
| Phellodendron chinense | MOL002662 | rutaecarpine | Matrix metalloproteinase-9 |
| Phellodendron chinense | MOL002662 | rutaecarpine | Tumor necrosis factor |
| Phellodendron chinense | MOL002662 | rutaecarpine | Cytochrome P450 3A4 |
| Phellodendron chinense | MOL002662 | rutaecarpine | null |
| Phellodendron chinense | MOL002662 | rutaecarpine | Interleukin-4 |
| Phellodendron chinense | MOL002662 | rutaecarpine | Cytochrome P450 2B6 |
| Phellodendron chinense | MOL002663 | Skimmianin | Retinoic acid receptor RXR-alpha |
| Phellodendron chinense | MOL002663 | Skimmianin | Gamma-aminobutyric-acid receptor subunit alpha-1 |
| Phellodendron chinense | MOL002663 | Skimmianin | Heat shock protein HSP 90-alpha |
| Phellodendron chinense | MOL002663 | Skimmianin | cAMP-dependent protein kinase catalytic subunit alpha |
| Phellodendron chinense | MOL002663 | Skimmianin | Phosphatidylinositol-4,5-bisphosphate 3-kinase catalytic subunit gamma isoform |
| Phellodendron chinense | MOL002666 | Chelerythrine | Prostaglandin G/H synthase 1 |
| Phellodendron chinense | MOL002666 | Chelerythrine | Potassium voltage-gated channel subfamily H member 2 |
| Phellodendron chinense | MOL002666 | Chelerythrine | Prostaglandin G/H synthase 2 |
| Phellodendron chinense | MOL002666 | Chelerythrine | Retinoic acid receptor RXR-alpha |
| Phellodendron chinense | MOL002666 | Chelerythrine | cAMP-dependent protein kinase catalytic subunit alpha |
| Phellodendron chinense | MOL002666 | Chelerythrine | Nuclear receptor coactivator 2 |
| Phellodendron chinense | MOL000449 | Stigmasterol | Progesterone receptor |
| Phellodendron chinense | MOL000449 | Stigmasterol | Mineralocorticoid receptor |
| Phellodendron chinense | MOL000449 | Stigmasterol | Nuclear receptor coactivator 2 |
| Phellodendron chinense | MOL000449 | Stigmasterol | null |
| Phellodendron chinense | MOL000449 | Stigmasterol | Ig gamma-1 chain C region |
| Phellodendron chinense | MOL000449 | Stigmasterol | Retinoic acid receptor RXR-alpha |
| Phellodendron chinense | MOL000449 | Stigmasterol | Nuclear receptor coactivator 1 |
| Phellodendron chinense | MOL000449 | Stigmasterol | Prostaglandin G/H synthase 1 |
| Phellodendron chinense | MOL000449 | Stigmasterol | Prostaglandin G/H synthase 2 |
| Phellodendron chinense | MOL000449 | Stigmasterol | Alpha-2A adrenergic receptor |
| Phellodendron chinense | MOL000449 | Stigmasterol | Sodium-dependent noradrenaline transporter |
| Phellodendron chinense | MOL000449 | Stigmasterol | Sodium-dependent dopamine transporter |
| Phellodendron chinense | MOL000449 | Stigmasterol | Beta-2 adrenergic receptor |
| Phellodendron chinense | MOL000449 | Stigmasterol | Aldose reductase |
| Phellodendron chinense | MOL000449 | Stigmasterol | Urokinase-type plasminogen activator |
| Phellodendron chinense | MOL000449 | Stigmasterol | Leukotriene A-4 hydrolase |
| Phellodendron chinense | MOL000449 | Stigmasterol | Amine oxidase [flavin-containing] B |
| Phellodendron chinense | MOL000449 | Stigmasterol | Amine oxidase [flavin-containing] A |
| Phellodendron chinense | MOL000449 | Stigmasterol | cAMP-dependent protein kinase catalytic subunit alpha |
| Phellodendron chinense | MOL000449 | Stigmasterol | Chymotrypsinogen B |
| Phellodendron chinense | MOL000449 | Stigmasterol | Muscarinic acetylcholine receptor M3 |
| Phellodendron chinense | MOL000449 | Stigmasterol | Muscarinic acetylcholine receptor M1 |
| Phellodendron chinense | MOL000449 | Stigmasterol | Beta-1 adrenergic receptor |
| Phellodendron chinense | MOL000449 | Stigmasterol | Sodium channel protein type 5 subunit alpha |
| Phellodendron chinense | MOL000449 | Stigmasterol | 5-hydroxytryptamine 2A receptor |
| Phellodendron chinense | MOL000449 | Stigmasterol | Alpha-1A adrenergic receptor |
| Phellodendron chinense | MOL000449 | Stigmasterol | Gamma-aminobutyric-acid receptor subunit alpha-3 |
| Phellodendron chinense | MOL000449 | Stigmasterol | Muscarinic acetylcholine receptor M2 |
| Phellodendron chinense | MOL000449 | Stigmasterol | Alpha-1B adrenergic receptor |
| Phellodendron chinense | MOL000449 | Stigmasterol | Gamma-aminobutyric-acid receptor subunit alpha-1 |
| Phellodendron chinense | MOL000449 | Stigmasterol | Neuronal acetylcholine receptor subunit alpha-7 |
| Phellodendron chinense | MOL002668 | Worenine | Nitric oxide synthase, inducible |
| Phellodendron chinense | MOL002668 | Worenine | Prostaglandin G/H synthase 1 |
| Phellodendron chinense | MOL002668 | Worenine | Estrogen receptor |
| Phellodendron chinense | MOL002668 | Worenine | Androgen receptor |
| Phellodendron chinense | MOL002668 | Worenine | Prostaglandin G/H synthase 2 |
| Phellodendron chinense | MOL002668 | Worenine | Serine/threonine-protein kinase Chk1 |
| Phellodendron chinense | MOL002668 | Worenine | Proto-oncogene serine/threonine-protein kinase Pim-1 |
| Phellodendron chinense | MOL002670 | Cavidine | Prostaglandin G/H synthase 1 |
| Phellodendron chinense | MOL002670 | Cavidine | Muscarinic acetylcholine receptor M3 |
| Phellodendron chinense | MOL002670 | Cavidine | Potassium voltage-gated channel subfamily H member 2 |
| Phellodendron chinense | MOL002670 | Cavidine | Muscarinic acetylcholine receptor M1 |
| Phellodendron chinense | MOL002670 | Cavidine | Beta-1 adrenergic receptor |
| Phellodendron chinense | MOL002670 | Cavidine | Sodium channel protein type 5 subunit alpha |
| Phellodendron chinense | MOL002670 | Cavidine | Coagulation factor X |
| Phellodendron chinense | MOL002670 | Cavidine | Muscarinic acetylcholine receptor M5 |
| Phellodendron chinense | MOL002670 | Cavidine | Prostaglandin G/H synthase 2 |
| Phellodendron chinense | MOL002670 | Cavidine | 5-hydroxytryptamine 3 receptor |
| Phellodendron chinense | MOL002670 | Cavidine | Alpha-2C adrenergic receptor |
| Phellodendron chinense | MOL002670 | Cavidine | Muscarinic acetylcholine receptor M4 |
| Phellodendron chinense | MOL002670 | Cavidine | Retinoic acid receptor RXR-alpha |
| Phellodendron chinense | MOL002670 | Cavidine | Delta-type opioid receptor |
| Phellodendron chinense | MOL002670 | Cavidine | 5-hydroxytryptamine 2A receptor |
| Phellodendron chinense | MOL002670 | Cavidine | 5-hydroxytryptamine 2C receptor |
| Phellodendron chinense | MOL002670 | Cavidine | Alpha-1B adrenergic receptor |
| Phellodendron chinense | MOL002670 | Cavidine | Beta-2 adrenergic receptor |
| Phellodendron chinense | MOL002670 | Cavidine | Alpha-1D adrenergic receptor |
| Phellodendron chinense | MOL002670 | Cavidine | DNA topoisomerase 2-alpha |
| Phellodendron chinense | MOL002670 | Cavidine | Mu-type opioid receptor |
| Phellodendron chinense | MOL002670 | Cavidine | Heat shock protein HSP 90-alpha |
| Phellodendron chinense | MOL002670 | Cavidine | null |
| Phellodendron chinense | MOL002670 | Cavidine | Calmodulin |
| Phellodendron chinense | MOL002670 | Cavidine | D(1A) dopamine receptor |
| Phellodendron chinense | MOL002670 | Cavidine | Sodium-dependent serotonin transporter |
| Phellodendron chinense | MOL002670 | Cavidine | Coagulation factor VII |
| Phellodendron chinense | MOL002670 | Cavidine | cAMP and cAMP-inhibited cGMP 3',5'-cyclic phosphodiesterase 10A |
| Phellodendron chinense | MOL002672 | Hericenone H | Prothrombin |
| Phellodendron chinense | MOL000358 | beta-sitosterol | Progesterone receptor |
| Phellodendron chinense | MOL000358 | beta-sitosterol | Nuclear receptor coactivator 2 |
| Phellodendron chinense | MOL000358 | beta-sitosterol | Prostaglandin G/H synthase 1 |
| Phellodendron chinense | MOL000358 | beta-sitosterol | Prostaglandin G/H synthase 2 |
| Phellodendron chinense | MOL000358 | beta-sitosterol | Heat shock protein HSP 90-alpha |
| Phellodendron chinense | MOL000358 | beta-sitosterol | Phosphatidylinositol-4,5-bisphosphate 3-kinase catalytic subunit gamma isoform |
| Phellodendron chinense | MOL000358 | beta-sitosterol | Potassium voltage-gated channel subfamily H member 2 |
| Phellodendron chinense | MOL000358 | beta-sitosterol | cAMP-dependent protein kinase catalytic subunit alpha |
| Phellodendron chinense | MOL000358 | beta-sitosterol | D(1A) dopamine receptor |
| Phellodendron chinense | MOL000358 | beta-sitosterol | Muscarinic acetylcholine receptor M3 |
| Phellodendron chinense | MOL000358 | beta-sitosterol | Muscarinic acetylcholine receptor M1 |
| Phellodendron chinense | MOL000358 | beta-sitosterol | Sodium channel protein type 5 subunit alpha |
| Phellodendron chinense | MOL000358 | beta-sitosterol | Gamma-aminobutyric-acid receptor subunit alpha-2 |
| Phellodendron chinense | MOL000358 | beta-sitosterol | Muscarinic acetylcholine receptor M4 |
| Phellodendron chinense | MOL000358 | beta-sitosterol | cGMP-inhibited 3',5'-cyclic phosphodiesterase A |
| Phellodendron chinense | MOL000358 | beta-sitosterol | 5-hydroxytryptamine 2A receptor |
| Phellodendron chinense | MOL000358 | beta-sitosterol | Gamma-aminobutyric-acid receptor subunit alpha-5 |
| Phellodendron chinense | MOL000358 | beta-sitosterol | Alpha-1A adrenergic receptor |
| Phellodendron chinense | MOL000358 | beta-sitosterol | Gamma-aminobutyric-acid receptor subunit alpha-3 |
| Phellodendron chinense | MOL000358 | beta-sitosterol | Muscarinic acetylcholine receptor M2 |
| Phellodendron chinense | MOL000358 | beta-sitosterol | Alpha-1B adrenergic receptor |
| Phellodendron chinense | MOL000358 | beta-sitosterol | Beta-2 adrenergic receptor |
| Phellodendron chinense | MOL000358 | beta-sitosterol | Neuronal acetylcholine receptor subunit alpha-2 |
| Phellodendron chinense | MOL000358 | beta-sitosterol | Sodium-dependent serotonin transporter |
| Phellodendron chinense | MOL000358 | beta-sitosterol | Mu-type opioid receptor |
| Phellodendron chinense | MOL000358 | beta-sitosterol | Gamma-aminobutyric-acid receptor subunit alpha-1 |
| Phellodendron chinense | MOL000358 | beta-sitosterol | Neuronal acetylcholine receptor subunit alpha-7 |
| Phellodendron chinense | MOL000358 | beta-sitosterol | Cytochrome P450-cam |
| Phellodendron chinense | MOL000358 | beta-sitosterol | Apoptosis regulator Bcl-2 |
| Phellodendron chinense | MOL000358 | beta-sitosterol | Apoptosis regulator BAX |
| Phellodendron chinense | MOL000358 | beta-sitosterol | Caspase-9 |
| Phellodendron chinense | MOL000358 | beta-sitosterol | Transcription factor AP-1 |
| Phellodendron chinense | MOL000358 | beta-sitosterol | Caspase-3 |
| Phellodendron chinense | MOL000358 | beta-sitosterol | Caspase-8 |
| Phellodendron chinense | MOL000358 | beta-sitosterol | Protein kinase C alpha type |
| Phellodendron chinense | MOL000358 | beta-sitosterol | Transforming growth factor beta-1 |
| Phellodendron chinense | MOL000358 | beta-sitosterol | Serum paraoxonase/arylesterase 1 |
| Phellodendron chinense | MOL000358 | beta-sitosterol | Microtubule-associated protein 2 |
| Phellodendron chinense | MOL000622 | Magnograndiolide | Gamma-aminobutyric-acid receptor subunit alpha-2 |
| Phellodendron chinense | MOL000622 | Magnograndiolide | Gamma-aminobutyric-acid receptor subunit alpha-1 |
| Phellodendron chinense | MOL000622 | Magnograndiolide | Glutamate receptor 2 |
| Phellodendron chinense | MOL000622 | Magnograndiolide | Gamma-aminobutyric-acid receptor subunit alpha-6 |
| Phellodendron chinense | MOL000785 | palmatine | Nitric oxide synthase, inducible |
| Phellodendron chinense | MOL000785 | palmatine | Prostaglandin G/H synthase 1 |
| Phellodendron chinense | MOL000785 | palmatine | Potassium voltage-gated channel subfamily H member 2 |
| Phellodendron chinense | MOL000785 | palmatine | Estrogen receptor |
| Phellodendron chinense | MOL000785 | palmatine | Androgen receptor |
| Phellodendron chinense | MOL000785 | palmatine | Sodium channel protein type 5 subunit alpha |
| Phellodendron chinense | MOL000785 | palmatine | Prostaglandin G/H synthase 2 |
| Phellodendron chinense | MOL000785 | palmatine | Nitric-oxide synthase, endothelial |
| Phellodendron chinense | MOL000785 | palmatine | Retinoic acid receptor RXR-alpha |
| Phellodendron chinense | MOL000785 | palmatine | Beta-2 adrenergic receptor |
| Phellodendron chinense | MOL000785 | palmatine | Estrogen receptor beta |
| Phellodendron chinense | MOL000785 | palmatine | Heat shock protein HSP 90-alpha |
| Phellodendron chinense | MOL000785 | palmatine | Trypsin-1 |
| Phellodendron chinense | MOL000785 | palmatine | Proto-oncogene serine/threonine-protein kinase Pim-1 |
| Phellodendron chinense | MOL000785 | palmatine | Nuclear receptor coactivator 2 |
| Phellodendron chinense | MOL000785 | palmatine | Calmodulin |
| Phellodendron chinense | MOL000785 | palmatine | cAMP-dependent protein kinase catalytic subunit alpha |
| Phellodendron chinense | MOL000785 | palmatine | Cell division protein kinase 2 |
| Phellodendron chinense | MOL000785 | palmatine | Coagulation factor VII |
| Coptis chinensis | MOL001454 | berberine | Nitric oxide synthase, inducible |
| Coptis chinensis | MOL001454 | berberine | Prostaglandin G/H synthase 1 |
| Coptis chinensis | MOL001454 | berberine | Potassium voltage-gated channel subfamily H member 2 |
| Coptis chinensis | MOL001454 | berberine | Estrogen receptor |
| Coptis chinensis | MOL001454 | berberine | Androgen receptor |
| Coptis chinensis | MOL001454 | berberine | Sodium channel protein type 5 subunit alpha |
| Coptis chinensis | MOL001454 | berberine | Coagulation factor Xa |
| Coptis chinensis | MOL001454 | berberine | Prostaglandin G/H synthase 2 |
| Coptis chinensis | MOL001454 | berberine | Nitric-oxide synthase, endothelial |
| Coptis chinensis | MOL001454 | berberine | Retinoic acid receptor RXR-alpha |
| Coptis chinensis | MOL001454 | berberine | Beta-2 adrenergic receptor |
| Coptis chinensis | MOL001454 | berberine | Heat shock protein HSP 90 |
| Coptis chinensis | MOL001454 | berberine | mRNA of PKA Catalytic Subunit C-alpha |
| Coptis chinensis | MOL001454 | berberine | Trypsin-1 |
| Coptis chinensis | MOL001454 | berberine | Nuclear receptor coactivator 2 |
| Coptis chinensis | MOL001454 | berberine | cAMP and cAMP-inhibited cGMP 3',5'-cyclic phosphodiesterase 10A |
| Coptis chinensis | MOL001454 | berberine | Calmodulin |
| Coptis chinensis | MOL002894 | berberrubine | Nitric oxide synthase, inducible |
| Coptis chinensis | MOL002894 | berberrubine | Prostaglandin G/H synthase 1 |
| Coptis chinensis | MOL002894 | berberrubine | Potassium voltage-gated channel subfamily H member 2 |
| Coptis chinensis | MOL002894 | berberrubine | Estrogen receptor |
| Coptis chinensis | MOL002894 | berberrubine | Androgen receptor |
| Coptis chinensis | MOL002894 | berberrubine | Sodium channel protein type 5 subunit alpha |
| Coptis chinensis | MOL002894 | berberrubine | Prostaglandin G/H synthase 2 |
| Coptis chinensis | MOL002894 | berberrubine | Nitric-oxide synthase, endothelial |
| Coptis chinensis | MOL002894 | berberrubine | Retinoic acid receptor RXR-alpha |
| Coptis chinensis | MOL002894 | berberrubine | mRNA of PKA Catalytic Subunit C-alpha |
| Coptis chinensis | MOL002894 | berberrubine | Trypsin-1 |
| Coptis chinensis | MOL002894 | berberrubine | Nuclear receptor coactivator 2 |
| Coptis chinensis | MOL002894 | berberrubine | Calmodulin |
| Coptis chinensis | MOL002897 | epiberberine | Nitric oxide synthase, inducible |
| Coptis chinensis | MOL002897 | epiberberine | Potassium voltage-gated channel subfamily H member 2 |
| Coptis chinensis | MOL002897 | epiberberine | Estrogen receptor |
| Coptis chinensis | MOL002897 | epiberberine | Androgen receptor |
| Coptis chinensis | MOL002897 | epiberberine | Prostaglandin G/H synthase 2 |
| Coptis chinensis | MOL002897 | epiberberine | Nitric-oxide synthase, endothelial |
| Coptis chinensis | MOL002897 | epiberberine | Retinoic acid receptor RXR-alpha |
| Coptis chinensis | MOL002897 | epiberberine | mRNA of PKA Catalytic Subunit C-alpha |
| Coptis chinensis | MOL002897 | epiberberine | Trypsin-1 |
| Coptis chinensis | MOL002897 | epiberberine | Nuclear receptor coactivator 2 |
| Coptis chinensis | MOL002897 | epiberberine | cAMP and cAMP-inhibited cGMP 3',5'-cyclic phosphodiesterase 10A |
| Coptis chinensis | MOL002903 | (R)-Canadine | Prostaglandin G/H synthase 1 |
| Coptis chinensis | MOL002903 | (R)-Canadine | Muscarinic acetylcholine receptor M3 |
| Coptis chinensis | MOL002903 | (R)-Canadine | Potassium voltage-gated channel subfamily H member 2 |
| Coptis chinensis | MOL002903 | (R)-Canadine | Muscarinic acetylcholine receptor M1 |
| Coptis chinensis | MOL002903 | (R)-Canadine | Sodium channel protein type 5 subunit alpha |
| Coptis chinensis | MOL002903 | (R)-Canadine | Coagulation factor Xa |
| Coptis chinensis | MOL002903 | (R)-Canadine | Muscarinic acetylcholine receptor M5 |
| Coptis chinensis | MOL002903 | (R)-Canadine | Prostaglandin G/H synthase 2 |
| Coptis chinensis | MOL002903 | (R)-Canadine | 5-hydroxytryptamine receptor 3A |
| Coptis chinensis | MOL002903 | (R)-Canadine | Alpha-2C adrenergic receptor |
| Coptis chinensis | MOL002903 | (R)-Canadine | Muscarinic acetylcholine receptor M4 |
| Coptis chinensis | MOL002903 | (R)-Canadine | Delta-type opioid receptor |
| Coptis chinensis | MOL002903 | (R)-Canadine | 5-hydroxytryptamine 2A receptor |
| Coptis chinensis | MOL002903 | (R)-Canadine | 5-hydroxytryptamine 2C receptor |
| Coptis chinensis | MOL002903 | (R)-Canadine | Alpha-1B adrenergic receptor |
| Coptis chinensis | MOL002903 | (R)-Canadine | Sodium-dependent dopamine transporter |
| Coptis chinensis | MOL002903 | (R)-Canadine | Beta-2 adrenergic receptor |
| Coptis chinensis | MOL002903 | (R)-Canadine | Alpha-1D adrenergic receptor |
| Coptis chinensis | MOL002903 | (R)-Canadine | Sodium-dependent serotonin transporter |
| Coptis chinensis | MOL002903 | (R)-Canadine | Mu-type opioid receptor |
| Coptis chinensis | MOL002903 | (R)-Canadine | Heat shock protein HSP 90 |
| Coptis chinensis | MOL002903 | (R)-Canadine | mRNA of PKA Catalytic Subunit C-alpha |
| Coptis chinensis | MOL002903 | (R)-Canadine | cAMP and cAMP-inhibited cGMP 3',5'-cyclic phosphodiesterase 10A |
| Coptis chinensis | MOL002903 | (R)-Canadine | Calmodulin |
| Coptis chinensis | MOL002903 | (R)-Canadine | Dopamine D1 receptor |
| Coptis chinensis | MOL002903 | (R)-Canadine | D(1B) dopamine receptor |
| Coptis chinensis | MOL002903 | (R)-Canadine | Retinoic acid receptor RXR-alpha |
| Coptis chinensis | MOL002903 | (R)-Canadine | Sodium-dependent noradrenaline transporter |
| Coptis chinensis | MOL002903 | (R)-Canadine | Alpha-1A adrenergic receptor |
| Coptis chinensis | MOL002903 | (R)-Canadine | Muscarinic acetylcholine receptor M2 |
| Coptis chinensis | MOL002903 | (R)-Canadine | Calcium-activated potassium channel subunit alpha 1 |
| Coptis chinensis | MOL002904 | Berlambine | Nitric oxide synthase, inducible |
| Coptis chinensis | MOL002904 | Berlambine | Prostaglandin G/H synthase 1 |
| Coptis chinensis | MOL002904 | Berlambine | Muscarinic acetylcholine receptor M3 |
| Coptis chinensis | MOL002904 | Berlambine | Potassium voltage-gated channel subfamily H member 2 |
| Coptis chinensis | MOL002904 | Berlambine | Androgen receptor |
| Coptis chinensis | MOL002904 | Berlambine | Sodium channel protein type 5 subunit alpha |
| Coptis chinensis | MOL002904 | Berlambine | Coagulation factor Xa |
| Coptis chinensis | MOL002904 | Berlambine | Prostaglandin G/H synthase 2 |
| Coptis chinensis | MOL002904 | Berlambine | Nitric-oxide synthase, endothelial |
| Coptis chinensis | MOL002904 | Berlambine | Coagulation factor VII |
| Coptis chinensis | MOL002904 | Berlambine | Retinoic acid receptor RXR-alpha |
| Coptis chinensis | MOL002904 | Berlambine | Alpha-1B adrenergic receptor |
| Coptis chinensis | MOL002904 | Berlambine | Beta-2 adrenergic receptor |
| Coptis chinensis | MOL002904 | Berlambine | Alpha-1D adrenergic receptor |
| Coptis chinensis | MOL002904 | Berlambine | Heat shock protein HSP 90 |
| Coptis chinensis | MOL002904 | Berlambine | mRNA of PKA Catalytic Subunit C-alpha |
| Coptis chinensis | MOL002904 | Berlambine | Trypsin-1 |
| Coptis chinensis | MOL002904 | Berlambine | Nuclear receptor coactivator 2 |
| Coptis chinensis | MOL002904 | Berlambine | Calcium-activated potassium channel subunit alpha 1 |
| Coptis chinensis | MOL002904 | Berlambine | Calmodulin |
| Coptis chinensis | MOL002907 | Corchoroside A_qt | Mineralocorticoid receptor |
| Coptis chinensis | MOL002907 | Corchoroside A_qt | Nuclear receptor coactivator 2 |
| Coptis chinensis | MOL000622 | Magnograndiolide | Gamma-aminobutyric-acid receptor alpha-2 subunit |
| Coptis chinensis | MOL000622 | Magnograndiolide | Gamma-aminobutyric acid receptor subunit alpha-1 |
| Coptis chinensis | MOL000622 | Magnograndiolide | Glutamate receptor 2 |
| Coptis chinensis | MOL000622 | Magnograndiolide | Gamma-aminobutyric-acid receptor subunit alpha-6 |
| Coptis chinensis | MOL000785 | palmatine | Nitric oxide synthase, inducible |
| Coptis chinensis | MOL000785 | palmatine | Prostaglandin G/H synthase 1 |
| Coptis chinensis | MOL000785 | palmatine | Potassium voltage-gated channel subfamily H member 2 |
| Coptis chinensis | MOL000785 | palmatine | Estrogen receptor |
| Coptis chinensis | MOL000785 | palmatine | Androgen receptor |
| Coptis chinensis | MOL000785 | palmatine | Sodium channel protein type 5 subunit alpha |
| Coptis chinensis | MOL000785 | palmatine | Prostaglandin G/H synthase 2 |
| Coptis chinensis | MOL000785 | palmatine | Nitric-oxide synthase, endothelial |
| Coptis chinensis | MOL000785 | palmatine | Retinoic acid receptor RXR-alpha |
| Coptis chinensis | MOL000785 | palmatine | Beta-2 adrenergic receptor |
| Coptis chinensis | MOL000785 | palmatine | Estrogen receptor beta |
| Coptis chinensis | MOL000785 | palmatine | Heat shock protein HSP 90 |
| Coptis chinensis | MOL000785 | palmatine | Trypsin-1 |
| Coptis chinensis | MOL000785 | palmatine | Proto-oncogene serine/threonine-protein kinase Pim-1 |
| Coptis chinensis | MOL000785 | palmatine | Nuclear receptor coactivator 2 |
| Coptis chinensis | MOL000785 | palmatine | Calmodulin |
| Coptis chinensis | MOL000785 | palmatine | mRNA of PKA Catalytic Subunit C-alpha |
| Coptis chinensis | MOL000785 | palmatine | Cell division protein kinase 2 |
| Coptis chinensis | MOL000785 | palmatine | Coagulation factor VII |
| Coptis chinensis | MOL000098 | quercetin | Prostaglandin G/H synthase 1 |
| Coptis chinensis | MOL000098 | quercetin | Androgen receptor |
| Coptis chinensis | MOL000098 | quercetin | Peroxisome proliferator activated receptor gamma |
| Coptis chinensis | MOL000098 | quercetin | Prostaglandin G/H synthase 2 |
| Coptis chinensis | MOL000098 | quercetin | Heat shock protein HSP 90 |
| Coptis chinensis | MOL000098 | quercetin | Phosphatidylinositol-4,5-bisphosphate 3-kinase catalytic subunit, gamma isoform |
| Coptis chinensis | MOL000098 | quercetin | Nuclear receptor coactivator 2 |
| Coptis chinensis | MOL000098 | quercetin | Dipeptidyl peptidase IV |
| Coptis chinensis | MOL000098 | quercetin | Aldose reductase |
| Coptis chinensis | MOL000098 | quercetin | Trypsin-1 |
| Coptis chinensis | MOL000098 | quercetin | DNA topoisomerase II |
| Coptis chinensis | MOL000098 | quercetin | Thrombin |
| Coptis chinensis | MOL000098 | quercetin | Potassium voltage-gated channel subfamily H member 2 |
| Coptis chinensis | MOL000098 | quercetin | Sodium channel protein type 5 subunit alpha |
| Coptis chinensis | MOL000098 | quercetin | Coagulation factor Xa |
| Coptis chinensis | MOL000098 | quercetin | Beta-2 adrenergic receptor |
| Coptis chinensis | MOL000098 | quercetin | Androgen receptor |
| Coptis chinensis | MOL000098 | quercetin | Peroxisome proliferator activated receptor gamma |
| Coptis chinensis | MOL000098 | quercetin | Prostaglandin G/H synthase 2 |
| Coptis chinensis | MOL000098 | quercetin | Heat shock protein HSP 90 |
| Coptis chinensis | MOL000098 | quercetin | Phosphatidylinositol-4,5-bisphosphate 3-kinase catalytic subunit, gamma isoform |
| Coptis chinensis | MOL000098 | quercetin | Nuclear receptor coactivator 2 |
| Coptis chinensis | MOL000098 | quercetin | Dipeptidyl peptidase IV |
| Coptis chinensis | MOL000098 | quercetin | Aldose reductase |
| Coptis chinensis | MOL000098 | quercetin | Trypsin-1 |
| Coptis chinensis | MOL000098 | quercetin | DNA topoisomerase II |
| Coptis chinensis | MOL000098 | quercetin | Thrombin |
| Coptis chinensis | MOL000098 | quercetin | Potassium voltage-gated channel subfamily H member 2 |
| Coptis chinensis | MOL000098 | quercetin | Sodium channel protein type 5 subunit alpha |
| Coptis chinensis | MOL000098 | quercetin | Coagulation factor Xa |
| Coptis chinensis | MOL000098 | quercetin | Beta-2 adrenergic receptor |
| Coptis chinensis | MOL000098 | quercetin | Proto-oncogene c-Fos |
| Coptis chinensis | MOL000098 | quercetin | Cyclin-dependent kinase inhibitor 1 |
| Coptis chinensis | MOL000098 | quercetin | Eukaryotic translation initiation factor 6 |
| Coptis chinensis | MOL000098 | quercetin | Apoptosis regulator BAX |
| Coptis chinensis | MOL000098 | quercetin | Caspase-9 |
| Coptis chinensis | MOL000098 | quercetin | Urokinase-type plasminogen activator |
| Coptis chinensis | MOL000098 | quercetin | 72 kDa type IV collagenase |
| Coptis chinensis | MOL000098 | quercetin | Matrix metalloproteinase-9 |
| Coptis chinensis | MOL000098 | quercetin | Mitogen-activated protein kinase 1 |
| Coptis chinensis | MOL000098 | quercetin | Interleukin-10 |
| Coptis chinensis | MOL000098 | quercetin | Pro-epidermal growth factor |
| Coptis chinensis | MOL000098 | quercetin | Retinoblastoma-associated protein |
| Coptis chinensis | MOL000098 | quercetin | Tumor necrosis factor |
| Coptis chinensis | MOL000098 | quercetin | Transcription factor AP-1 |
| Coptis chinensis | MOL000098 | quercetin | Interleukin-6 |
| Coptis chinensis | MOL000098 | quercetin | Cyclin-dependent kinase inhibitor 2A, isoforms 1/2/3 |
| Coptis chinensis | MOL000098 | quercetin | Activator of 90 kDa heat shock protein ATPase homolog 1 |
| Coptis chinensis | MOL000098 | quercetin | Caspase-3 |
| Coptis chinensis | MOL000098 | quercetin | Cellular tumor antigen p53 |
| Coptis chinensis | MOL000098 | quercetin | ETS domain-containing protein Elk-1 |
| Coptis chinensis | MOL000098 | quercetin | NF-kappa-B inhibitor alpha |
| Coptis chinensis | MOL000098 | quercetin | NADPH--cytochrome P450 reductase |
| Coptis chinensis | MOL000098 | quercetin | Ornithine decarboxylase |
| Coptis chinensis | MOL000098 | quercetin | Xanthine dehydrogenase/oxidase |
| Coptis chinensis | MOL000098 | quercetin | Caspase-8 |
| Coptis chinensis | MOL000098 | quercetin | DNA topoisomerase 1 |
| Coptis chinensis | MOL000098 | quercetin | RAF proto-oncogene serine/threonine-protein kinase |
| Coptis chinensis | MOL000098 | quercetin | Superoxide dismutase [Cu-Zn] |
| Coptis chinensis | MOL000098 | quercetin | Protein kinase C alpha type |
| Coptis chinensis | MOL000098 | quercetin | Interstitial collagenase |
| Coptis chinensis | MOL000098 | quercetin | Hypoxia-inducible factor 1-alpha |
| Coptis chinensis | MOL000098 | quercetin | Signal transducer and activator of transcription 1-alpha/beta |
| Coptis chinensis | MOL000098 | quercetin | Protein CBFA2T1 |
| Coptis chinensis | MOL000098 | quercetin | Probable E3 ubiquitin-protein ligase HERC5 |
| Coptis chinensis | MOL000098 | quercetin | Cell division control protein 2 homolog |
| Coptis chinensis | MOL000098 | quercetin | 78 kDa glucose-regulated protein |
| Coptis chinensis | MOL000098 | quercetin | Receptor tyrosine-protein kinase erbB-2 |
| Coptis chinensis | MOL000098 | quercetin | Peroxisome proliferator-activated receptor gamma |
| Coptis chinensis | MOL000098 | quercetin | Acetyl-CoA carboxylase 1 |
| Coptis chinensis | MOL000098 | quercetin | Heme oxygenase 1 |
| Coptis chinensis | MOL000098 | quercetin | Cytochrome P450 3A4 |
| Coptis chinensis | MOL000098 | quercetin | Cytochrome P450 1A2 |
| Coptis chinensis | MOL000098 | quercetin | Caveolin-1 |
| Coptis chinensis | MOL000098 | quercetin | Myc proto-oncogene protein |
| Coptis chinensis | MOL000098 | quercetin | Tissue factor |
| Coptis chinensis | MOL000098 | quercetin | Gap junction alpha-1 protein |
| Coptis chinensis | MOL000098 | quercetin | Cytochrome P450 1A1 |
| Coptis chinensis | MOL000098 | quercetin | Intercellular adhesion molecule 1 |
| Coptis chinensis | MOL000098 | quercetin | Interleukin-1 beta |
| Coptis chinensis | MOL000098 | quercetin | C-C motif chemokine 2 |
| Coptis chinensis | MOL000098 | quercetin | E-selectin |
| Coptis chinensis | MOL000098 | quercetin | Vascular cell adhesion protein 1 |
| Coptis chinensis | MOL000098 | quercetin | Prostaglandin E2 receptor EP3 subtype |
| Coptis chinensis | MOL000098 | quercetin | Interleukin-8 |
| Coptis chinensis | MOL000098 | quercetin | Protein kinase C beta type |
| Coptis chinensis | MOL000098 | quercetin | Baculoviral IAP repeat-containing protein 5 |
| Coptis chinensis | MOL000098 | quercetin | Dual oxidase 2 |
| Coptis chinensis | MOL000098 | quercetin | Nitric oxide synthase, endothelial |
| Coptis chinensis | MOL000098 | quercetin | Heat shock protein beta-1 |
| Coptis chinensis | MOL000098 | quercetin | Transforming growth factor beta-1 |
| Coptis chinensis | MOL000098 | quercetin | Estrogen sulfotransferase |
| Coptis chinensis | MOL000098 | quercetin | Maltase-glucoamylase, intestinal |
| Coptis chinensis | MOL000098 | quercetin | Interleukin-2 |
| Coptis chinensis | MOL000098 | quercetin | Nuclear receptor subfamily 1 group I member 2 |
| Coptis chinensis | MOL000098 | quercetin | Cytochrome P450 1B1 |
| Coptis chinensis | MOL000098 | quercetin | G2/mitotic-specific cyclin-B1 |
| Coptis chinensis | MOL000098 | quercetin | Tissue-type plasminogen activator |
| Coptis chinensis | MOL000098 | quercetin | Thrombomodulin |
| Coptis chinensis | MOL000098 | quercetin | Plasminogen activator inhibitor 1 |
| Coptis chinensis | MOL000098 | quercetin | Collagen alpha-1(I) chain |
| Coptis chinensis | MOL000098 | quercetin | Interferon gamma |
| Coptis chinensis | MOL000098 | quercetin | Arachidonate 5-lipoxygenase |
| Coptis chinensis | MOL000098 | quercetin | Phosphatidylinositol-3,4,5-trisphosphate 3-phosphatase and dual-specificity protein phosphatase PTEN |
| Coptis chinensis | MOL000098 | quercetin | Interleukin-1 alpha |
| Coptis chinensis | MOL000098 | quercetin | Myeloperoxidase |
| Coptis chinensis | MOL000098 | quercetin | DNA topoisomerase 2-alpha |
| Coptis chinensis | MOL000098 | quercetin | Neutrophil cytosol factor 1 |
| Coptis chinensis | MOL000098 | quercetin | ATP-binding cassette sub-family G member 2 |
| Coptis chinensis | MOL000098 | quercetin | Hyaluronan synthase 2 |
| Coptis chinensis | MOL000098 | quercetin | Glutathione S-transferase P |
| Coptis chinensis | MOL000098 | quercetin | Nuclear factor erythroid 2-related factor 2 |
| Coptis chinensis | MOL000098 | quercetin | NAD(P)H dehydrogenase [quinone] 1 |
| Coptis chinensis | MOL000098 | quercetin | Poly [ADP-ribose] polymerase 1 |
| Coptis chinensis | MOL000098 | quercetin | Aryl hydrocarbon receptor |
| Coptis chinensis | MOL000098 | quercetin | 26S proteasome non-ATPase regulatory subunit 3 |
| Coptis chinensis | MOL000098 | quercetin | Solute carrier family 2, facilitated glucose transporter member 4 |
| Coptis chinensis | MOL000098 | quercetin | Collagen alpha-1(III) chain |
| Coptis chinensis | MOL000098 | quercetin | DNA gyrase subunit B |
| Coptis chinensis | MOL000098 | quercetin | C-X-C motif chemokine 11 |
| Coptis chinensis | MOL000098 | quercetin | C-X-C motif chemokine 2 |
| Coptis chinensis | MOL000098 | quercetin | DDB1- and CUL4-associated factor 5 |
| Coptis chinensis | MOL000098 | quercetin | Nuclear receptor subfamily 1 group I member 3 |
| Coptis chinensis | MOL000098 | quercetin | Serine/threonine-protein kinase Chk2 |
| Coptis chinensis | MOL000098 | quercetin | Insulin receptor |
| Coptis chinensis | MOL000098 | quercetin | Claudin-4 |
| Coptis chinensis | MOL000098 | quercetin | Peroxisome proliferator-activated receptor alpha |
| Coptis chinensis | MOL000098 | quercetin | Peroxisome proliferator-activated receptor delta |
| Coptis chinensis | MOL000098 | quercetin | Heat shock factor protein 1 |
| Coptis chinensis | MOL000098 | quercetin | C-reactive protein |
| Coptis chinensis | MOL000098 | quercetin | C-X-C motif chemokine 10 |
| Coptis chinensis | MOL000098 | quercetin | Inhibitor of nuclear factor kappa-B kinase subunit alpha |
| Coptis chinensis | MOL000098 | quercetin | Osteopontin |
| Coptis chinensis | MOL000098 | quercetin | Runt-related transcription factor 2 |
| Coptis chinensis | MOL000098 | quercetin | Ras association domain-containing protein 1 |
| Coptis chinensis | MOL000098 | quercetin | Transcription factor E2F1 |
| Coptis chinensis | MOL000098 | quercetin | Transcription factor E2F2 |
| Coptis chinensis | MOL000098 | quercetin | Prostatic acid phosphatase |
| Coptis chinensis | MOL000098 | quercetin | Cathepsin D |
| Coptis chinensis | MOL000098 | quercetin | Insulin-like growth factor-binding protein 3 |
| Coptis chinensis | MOL000098 | quercetin | Insulin-like growth factor II |
| Coptis chinensis | MOL000098 | quercetin | CD40 ligand |
| Coptis chinensis | MOL000098 | quercetin | Interferon regulatory factor 1 |
| Coptis chinensis | MOL000098 | quercetin | Receptor tyrosine-protein kinase erbB-3 |
| Coptis chinensis | MOL000098 | quercetin | Serum paraoxonase/arylesterase 1 |
| Coptis chinensis | MOL000098 | quercetin | Type I iodothyronine deiodinase |
| Coptis chinensis | MOL000098 | quercetin | Procollagen C-endopeptidase enhancer 1 |
| Coptis chinensis | MOL000098 | quercetin | Puromycin-sensitive aminopeptidase |
| Coptis chinensis | MOL000098 | quercetin | Hexokinase-2 |
| Coptis chinensis | MOL000098 | quercetin | Homeobox protein Nkx-3.1 |
| Coptis chinensis | MOL000098 | quercetin | Ras GTPase-activating protein 1 |
| Coptis chinensis | MOL000098 | quercetin | Peroxidase C1A |
| Coptis chinensis | MOL000098 | quercetin | Glutathione S-transferase Mu 1 |
| Coptis chinensis | MOL000098 | quercetin | Glutathione S-transferase Mu 2 |
| Coptis chinensis | MOL001458 | coptisine | Nitric oxide synthase, inducible |
| Coptis chinensis | MOL001458 | coptisine | Prostaglandin G/H synthase 1 |
| Coptis chinensis | MOL001458 | coptisine | Potassium voltage-gated channel subfamily H member 2 |
| Coptis chinensis | MOL001458 | coptisine | Estrogen receptor |
| Coptis chinensis | MOL001458 | coptisine | Androgen receptor |
| Coptis chinensis | MOL001458 | coptisine | Sodium channel protein type 5 subunit alpha |
| Coptis chinensis | MOL001458 | coptisine | Prostaglandin G/H synthase 2 |
| Coptis chinensis | MOL001458 | coptisine | Nitric-oxide synthase, endothelial |
| Coptis chinensis | MOL001458 | coptisine | Trypsin-1 |
| Coptis chinensis | MOL002668 | Worenine | Nitric oxide synthase, inducible |
| Coptis chinensis | MOL002668 | Worenine | Prostaglandin G/H synthase 1 |
| Coptis chinensis | MOL002668 | Worenine | Estrogen receptor |
| Coptis chinensis | MOL002668 | Worenine | Androgen receptor |
| Coptis chinensis | MOL002668 | Worenine | Prostaglandin G/H synthase 2 |
| Coptis chinensis | MOL002668 | Worenine | Serine/threonine-protein kinase Chk1 |
| Coptis chinensis | MOL002668 | Worenine | Proto-oncogene serine/threonine-protein kinase Pim-1 |
| Cortex fraxini | MOL000358 | beta-sitosterol | Progesterone receptor |
| Cortex fraxini | MOL000358 | beta-sitosterol | Nuclear receptor coactivator 2 |
| Cortex fraxini | MOL000358 | beta-sitosterol | Prostaglandin G/H synthase 1 |
| Cortex fraxini | MOL000358 | beta-sitosterol | Prostaglandin G/H synthase 2 |
| Cortex fraxini | MOL000358 | beta-sitosterol | Heat shock protein HSP 90-alpha |
| Cortex fraxini | MOL000358 | beta-sitosterol | Phosphatidylinositol-4,5-bisphosphate 3-kinase catalytic subunit gamma isoform |
| Cortex fraxini | MOL000358 | beta-sitosterol | Potassium voltage-gated channel subfamily H member 2 |
| Cortex fraxini | MOL000358 | beta-sitosterol | cAMP-dependent protein kinase catalytic subunit alpha |
| Cortex fraxini | MOL000358 | beta-sitosterol | D(1A) dopamine receptor |
| Cortex fraxini | MOL000358 | beta-sitosterol | Muscarinic acetylcholine receptor M3 |
| Cortex fraxini | MOL000358 | beta-sitosterol | Muscarinic acetylcholine receptor M1 |
| Cortex fraxini | MOL000358 | beta-sitosterol | Sodium channel protein type 5 subunit alpha |
| Cortex fraxini | MOL000358 | beta-sitosterol | Gamma-aminobutyric-acid receptor subunit alpha-2 |
| Cortex fraxini | MOL000358 | beta-sitosterol | Muscarinic acetylcholine receptor M4 |
| Cortex fraxini | MOL000358 | beta-sitosterol | cGMP-inhibited 3',5'-cyclic phosphodiesterase A |
| Cortex fraxini | MOL000358 | beta-sitosterol | 5-hydroxytryptamine 2A receptor |
| Cortex fraxini | MOL000358 | beta-sitosterol | Alpha-1A adrenergic receptor |
| Cortex fraxini | MOL000358 | beta-sitosterol | Gamma-aminobutyric-acid receptor subunit alpha-3 |
| Cortex fraxini | MOL000358 | beta-sitosterol | Muscarinic acetylcholine receptor M2 |
| Cortex fraxini | MOL000358 | beta-sitosterol | Alpha-1B adrenergic receptor |
| Cortex fraxini | MOL000358 | beta-sitosterol | Beta-2 adrenergic receptor |
| Cortex fraxini | MOL000358 | beta-sitosterol | Neuronal acetylcholine receptor subunit alpha-2 |
| Cortex fraxini | MOL000358 | beta-sitosterol | Sodium-dependent serotonin transporter |
| Cortex fraxini | MOL000358 | beta-sitosterol | Mu-type opioid receptor |
| Cortex fraxini | MOL000358 | beta-sitosterol | Gamma-aminobutyric-acid receptor subunit alpha-1 |
| Cortex fraxini | MOL000358 | beta-sitosterol | Neuronal acetylcholine receptor subunit alpha-7 |
| Cortex fraxini | MOL000358 | beta-sitosterol | Cytochrome P450-cam |
| Cortex fraxini | MOL000358 | beta-sitosterol | Apoptosis regulator Bcl-2 |
| Cortex fraxini | MOL000358 | beta-sitosterol | Apoptosis regulator BAX |
| Cortex fraxini | MOL000358 | beta-sitosterol | Caspase-9 |
| Cortex fraxini | MOL000358 | beta-sitosterol | Transcription factor AP-1 |
| Cortex fraxini | MOL000358 | beta-sitosterol | Caspase-3 |
| Cortex fraxini | MOL000358 | beta-sitosterol | Caspase-8 |
| Cortex fraxini | MOL000358 | beta-sitosterol | Protein kinase C alpha type |
| Cortex fraxini | MOL000358 | beta-sitosterol | Transforming growth factor beta-1 |
| Cortex fraxini | MOL000358 | beta-sitosterol | Serum paraoxonase/arylesterase 1 |
| Cortex fraxini | MOL000358 | beta-sitosterol | Microtubule-associated protein 2 |
| Cortex fraxini | MOL006709 | AIDS214634 | Prostaglandin G/H synthase 2 |
| Cortex fraxini | MOL006709 | AIDS214634 | Nuclear receptor coactivator 2 |
| Cortex fraxini | MOL006709 | AIDS214634 | Calmodulin |
| Cortex fraxini | MOL006709 | AIDS214634 | Potassium voltage-gated channel subfamily H member 2 |
| Cortex fraxini | MOL006709 | AIDS214634 | Coagulation factor X |
| Cortex fraxini | MOL006709 | AIDS214634 | Coagulation factor VII |
| Cortex fraxini | MOL006710 | 8-(beta-D-Glucopyranosyloxy)-7-hydroxy-6-methoxy-2H-1-benzopyran-2-one | Prothrombin |
| Cortex fraxini | MOL006710 | 8-(beta-D-Glucopyranosyloxy)-7-hydroxy-6-methoxy-2H-1-benzopyran-2-one | Prostaglandin G/H synthase 2 |
| Cortex fraxini | MOL006710 | 8-(beta-D-Glucopyranosyloxy)-7-hydroxy-6-methoxy-2H-1-benzopyran-2-one | Tyrosine-protein phosphatase non-receptor type 1 |
| Cortex fraxini | MOL006710 | 8-(beta-D-Glucopyranosyloxy)-7-hydroxy-6-methoxy-2H-1-benzopyran-2-one | DNA topoisomerase 2-alpha |
| Cortex fraxini | MOL006710 | 8-(beta-D-Glucopyranosyloxy)-7-hydroxy-6-methoxy-2H-1-benzopyran-2-one | Trypsin-1 |
