# Supplementary material for: Network Pharmacology-Based Strategy to Identify the Pharmacological Mechanisms of Pulsatilla Decoction against Crohn’s Disease
Source: Front Pharmacol. 2022 Apr 5;13:844685. doi: 10.3389/fphar.2022.844685 (PMC9016333; doi:10.3389/fphar.2022.844685)
Supplement: Supplementary file 1 [file DataSheet1.zip › Table (2).DOCX]

| **Supplemental Table 2. Drug-compound-gene symbols after annotation** | | | |
| --- | --- | --- | --- |
| Drug | MolId | MolName | Symbol |
| Pulsatilla chinensis | MOL001973 | Sitosteryl acetate | PGR |
| Pulsatilla chinensis | MOL001978 | Aureusidin | NOS2 |
| Pulsatilla chinensis | MOL001978 | Aureusidin | PTGS1 |
| Pulsatilla chinensis | MOL001978 | Aureusidin | ESR1 |
| Pulsatilla chinensis | MOL001978 | Aureusidin | AR |
| Pulsatilla chinensis | MOL001978 | Aureusidin | PPARG |
| Pulsatilla chinensis | MOL001978 | Aureusidin | PTGS2 |
| Pulsatilla chinensis | MOL001978 | Aureusidin | CA2 |
| Pulsatilla chinensis | MOL001978 | Aureusidin | ESR2 |
| Pulsatilla chinensis | MOL001978 | Aureusidin | MAPK14 |
| Pulsatilla chinensis | MOL001978 | Aureusidin | GSK3B |
| Pulsatilla chinensis | MOL001978 | Aureusidin | HSP90AA1 |
| Pulsatilla chinensis | MOL001978 | Aureusidin | CDK2 |
| Pulsatilla chinensis | MOL001978 | Aureusidin | PRKACA |
| Pulsatilla chinensis | MOL001978 | Aureusidin | PRSS1 |
| Pulsatilla chinensis | MOL001978 | Aureusidin | CCNA2 |
| Pulsatilla chinensis | MOL001979 | LAN | PGR |
| Pulsatilla chinensis | MOL001979 | LAN | NR3C2 |
| Pulsatilla chinensis | MOL001979 | LAN | NCOA2 |
| Pulsatilla chinensis | MOL001985 | ZINC01615307 | PTGS1 |
| Pulsatilla chinensis | MOL001985 | ZINC01615307 | KCNH2 |
| Pulsatilla chinensis | MOL001985 | ZINC01615307 | F10 |
| Pulsatilla chinensis | MOL001985 | ZINC01615307 | PTGS2 |
| Pulsatilla chinensis | MOL001985 | ZINC01615307 | KDR |
| Pulsatilla chinensis | MOL001985 | ZINC01615307 | PTPN1 |
| Pulsatilla chinensis | MOL001985 | ZINC01615307 | TOP2A |
| Pulsatilla chinensis | MOL001985 | ZINC01615307 | HSP90AA1 |
| Pulsatilla chinensis | MOL001985 | ZINC01615307 | NCOA2 |
| Pulsatilla chinensis | MOL001985 | ZINC01615307 | CAMKMT |
| Pulsatilla chinensis | MOL001987 | β-sitosterol | TOP2A |
| Pulsatilla chinensis | MOL000211 | Mairin | PGR |
| Pulsatilla chinensis | MOL000354 | isorhamnetin | NOS2 |
| Pulsatilla chinensis | MOL000354 | isorhamnetin | PTGS1 |
| Pulsatilla chinensis | MOL000354 | isorhamnetin | ESR1 |
| Pulsatilla chinensis | MOL000354 | isorhamnetin | AR |
| Pulsatilla chinensis | MOL000354 | isorhamnetin | PPARG |
| Pulsatilla chinensis | MOL000354 | isorhamnetin | PTGS2 |
| Pulsatilla chinensis | MOL000354 | isorhamnetin | PTPN1 |
| Pulsatilla chinensis | MOL000354 | isorhamnetin | ESR2 |
| Pulsatilla chinensis | MOL000354 | isorhamnetin | DPP4 |
| Pulsatilla chinensis | MOL000354 | isorhamnetin | MAPK14 |
| Pulsatilla chinensis | MOL000354 | isorhamnetin | GSK3B |
| Pulsatilla chinensis | MOL000354 | isorhamnetin | HSP90AA1 |
| Pulsatilla chinensis | MOL000354 | isorhamnetin | CDK2 |
| Pulsatilla chinensis | MOL000354 | isorhamnetin | PRKACA |
| Pulsatilla chinensis | MOL000354 | isorhamnetin | PRSS1 |
| Pulsatilla chinensis | MOL000354 | isorhamnetin | CCNA2 |
| Pulsatilla chinensis | MOL000354 | isorhamnetin | NCOA2 |
| Pulsatilla chinensis | MOL000354 | isorhamnetin | CAMKMT |
| Pulsatilla chinensis | MOL000354 | isorhamnetin | PYGM |
| Pulsatilla chinensis | MOL000354 | isorhamnetin | PPARD |
| Pulsatilla chinensis | MOL000354 | isorhamnetin | CHEK1 |
| Pulsatilla chinensis | MOL000354 | isorhamnetin | AKR1B1 |
| Pulsatilla chinensis | MOL000354 | isorhamnetin | NCOA1 |
| Pulsatilla chinensis | MOL000354 | isorhamnetin | F7 |
| Pulsatilla chinensis | MOL000354 | isorhamnetin | F2 |
| Pulsatilla chinensis | MOL000354 | isorhamnetin | ACHE |
| Pulsatilla chinensis | MOL000354 | isorhamnetin | MAOB |
| Pulsatilla chinensis | MOL000354 | isorhamnetin | GRIA2 |
| Pulsatilla chinensis | MOL000354 | isorhamnetin | RELA |
| Pulsatilla chinensis | MOL000354 | isorhamnetin | NCF1 |
| Pulsatilla chinensis | MOL000354 | isorhamnetin | OLR1 |
| Pulsatilla chinensis | MOL000358 | beta-sitosterol | PGR |
| Pulsatilla chinensis | MOL000358 | beta-sitosterol | NCOA2 |
| Pulsatilla chinensis | MOL000358 | beta-sitosterol | PTGS1 |
| Pulsatilla chinensis | MOL000358 | beta-sitosterol | PTGS2 |
| Pulsatilla chinensis | MOL000358 | beta-sitosterol | HSP90AA1 |
| Pulsatilla chinensis | MOL000358 | beta-sitosterol | KCNH2 |
| Pulsatilla chinensis | MOL000358 | beta-sitosterol | PRKACA |
| Pulsatilla chinensis | MOL000358 | beta-sitosterol | DRD1 |
| Pulsatilla chinensis | MOL000358 | beta-sitosterol | CHRM3 |
| Pulsatilla chinensis | MOL000358 | beta-sitosterol | CHRM1 |
| Pulsatilla chinensis | MOL000358 | beta-sitosterol | SCN5A |
| Pulsatilla chinensis | MOL000358 | beta-sitosterol | CHRM4 |
| Pulsatilla chinensis | MOL000358 | beta-sitosterol | PDE3A |
| Pulsatilla chinensis | MOL000358 | beta-sitosterol | ADRA1A |
| Pulsatilla chinensis | MOL000358 | beta-sitosterol | CHRM2 |
| Pulsatilla chinensis | MOL000358 | beta-sitosterol | ADRA1B |
| Pulsatilla chinensis | MOL000358 | beta-sitosterol | ADRB2 |
| Pulsatilla chinensis | MOL000358 | beta-sitosterol | CHRNA2 |
| Pulsatilla chinensis | MOL000358 | beta-sitosterol | SLC6A4 |
| Pulsatilla chinensis | MOL000358 | beta-sitosterol | OPRM1 |
| Pulsatilla chinensis | MOL000358 | beta-sitosterol | CHRNA7 |
| Pulsatilla chinensis | MOL000358 | beta-sitosterol | BCL2 |
| Pulsatilla chinensis | MOL000358 | beta-sitosterol | BAX |
| Pulsatilla chinensis | MOL000358 | beta-sitosterol | CASP9 |
| Pulsatilla chinensis | MOL000358 | beta-sitosterol | JUN |
| Pulsatilla chinensis | MOL000358 | beta-sitosterol | CASP3 |
| Pulsatilla chinensis | MOL000358 | beta-sitosterol | CASP8 |
| Pulsatilla chinensis | MOL000358 | beta-sitosterol | PRKCA |
| Pulsatilla chinensis | MOL000358 | beta-sitosterol | PON1 |
| Pulsatilla chinensis | MOL000358 | beta-sitosterol | MAP2 |
| Pulsatilla chinensis | MOL000449 | Stigmasterol | PGR |
| Pulsatilla chinensis | MOL000449 | Stigmasterol | NR3C2 |
| Pulsatilla chinensis | MOL000449 | Stigmasterol | NCOA2 |
| Pulsatilla chinensis | MOL000449 | Stigmasterol | IGHG1 |
| Pulsatilla chinensis | MOL000449 | Stigmasterol | RXRA |
| Pulsatilla chinensis | MOL000449 | Stigmasterol | NCOA1 |
| Pulsatilla chinensis | MOL000449 | Stigmasterol | PTGS1 |
| Pulsatilla chinensis | MOL000449 | Stigmasterol | PTGS2 |
| Pulsatilla chinensis | MOL000449 | Stigmasterol | ADRA2A |
| Pulsatilla chinensis | MOL000449 | Stigmasterol | SLC6A2 |
| Pulsatilla chinensis | MOL000449 | Stigmasterol | SLC6A3 |
| Pulsatilla chinensis | MOL000449 | Stigmasterol | ADRB2 |
| Pulsatilla chinensis | MOL000449 | Stigmasterol | AKR1B1 |
| Pulsatilla chinensis | MOL000449 | Stigmasterol | PLAU |
| Pulsatilla chinensis | MOL000449 | Stigmasterol | LTA4H |
| Pulsatilla chinensis | MOL000449 | Stigmasterol | MAOB |
| Pulsatilla chinensis | MOL000449 | Stigmasterol | MAOA |
| Pulsatilla chinensis | MOL000449 | Stigmasterol | PRKACA |
| Pulsatilla chinensis | MOL000449 | Stigmasterol | CTRB1 |
| Pulsatilla chinensis | MOL000449 | Stigmasterol | CHRM3 |
| Pulsatilla chinensis | MOL000449 | Stigmasterol | CHRM1 |
| Pulsatilla chinensis | MOL000449 | Stigmasterol | ADRB1 |
| Pulsatilla chinensis | MOL000449 | Stigmasterol | SCN5A |
| Pulsatilla chinensis | MOL000449 | Stigmasterol | ADRA1A |
| Pulsatilla chinensis | MOL000449 | Stigmasterol | CHRM2 |
| Pulsatilla chinensis | MOL000449 | Stigmasterol | ADRA1B |
| Pulsatilla chinensis | MOL000449 | Stigmasterol | CHRNA7 |
| Phellodendron chinense | MOL001454 | berberine | NOS2 |
| Phellodendron chinense | MOL001454 | berberine | PTGS1 |
| Phellodendron chinense | MOL001454 | berberine | KCNH2 |
| Phellodendron chinense | MOL001454 | berberine | ESR1 |
| Phellodendron chinense | MOL001454 | berberine | AR |
| Phellodendron chinense | MOL001454 | berberine | SCN5A |
| Phellodendron chinense | MOL001454 | berberine | F10 |
| Phellodendron chinense | MOL001454 | berberine | PTGS2 |
| Phellodendron chinense | MOL001454 | berberine | RXRA |
| Phellodendron chinense | MOL001454 | berberine | ADRB2 |
| Phellodendron chinense | MOL001454 | berberine | HSP90AA1 |
| Phellodendron chinense | MOL001454 | berberine | PRKACA |
| Phellodendron chinense | MOL001454 | berberine | PRSS1 |
| Phellodendron chinense | MOL001454 | berberine | NCOA2 |
| Phellodendron chinense | MOL001454 | berberine | PDE10A |
| Phellodendron chinense | MOL001454 | berberine | CAMKMT |
| Phellodendron chinense | MOL001458 | coptisine | NOS2 |
| Phellodendron chinense | MOL001458 | coptisine | PTGS1 |
| Phellodendron chinense | MOL001458 | coptisine | KCNH2 |
| Phellodendron chinense | MOL001458 | coptisine | ESR1 |
| Phellodendron chinense | MOL001458 | coptisine | AR |
| Phellodendron chinense | MOL001458 | coptisine | SCN5A |
| Phellodendron chinense | MOL001458 | coptisine | PTGS2 |
| Phellodendron chinense | MOL001458 | coptisine | PRSS1 |
| Phellodendron chinense | MOL002641 | Phellavin_qt | PTGS2 |
| Phellodendron chinense | MOL002641 | Phellavin_qt | F7 |
| Phellodendron chinense | MOL002641 | Phellavin_qt | HSP90AA1 |
| Phellodendron chinense | MOL002643 | delta 7-stigmastenol | PGR |
| Phellodendron chinense | MOL002644 | Phellopterin | F2 |
| Phellodendron chinense | MOL002644 | Phellopterin | CHRM1 |
| Phellodendron chinense | MOL002644 | Phellopterin | SCN5A |
| Phellodendron chinense | MOL002644 | Phellopterin | PTGS2 |
| Phellodendron chinense | MOL002644 | Phellopterin | RXRA |
| Phellodendron chinense | MOL002644 | Phellopterin | ADRA1B |
| Phellodendron chinense | MOL002644 | Phellopterin | PTPN1 |
| Phellodendron chinense | MOL002644 | Phellopterin | ADRB2 |
| Phellodendron chinense | MOL002644 | Phellopterin | DPP4 |
| Phellodendron chinense | MOL002644 | Phellopterin | HSP90AA1 |
| Phellodendron chinense | MOL002644 | Phellopterin | CHRNA7 |
| Phellodendron chinense | MOL002651 | Dehydrotanshinone II A | DRD1 |
| Phellodendron chinense | MOL002651 | Dehydrotanshinone II A | CHRM3 |
| Phellodendron chinense | MOL002651 | Dehydrotanshinone II A | F2 |
| Phellodendron chinense | MOL002651 | Dehydrotanshinone II A | CHRM1 |
| Phellodendron chinense | MOL002651 | Dehydrotanshinone II A | ESR1 |
| Phellodendron chinense | MOL002651 | Dehydrotanshinone II A | AR |
| Phellodendron chinense | MOL002651 | Dehydrotanshinone II A | SCN5A |
| Phellodendron chinense | MOL002651 | Dehydrotanshinone II A | PPARG |
| Phellodendron chinense | MOL002651 | Dehydrotanshinone II A | CHRM5 |
| Phellodendron chinense | MOL002651 | Dehydrotanshinone II A | PTGS2 |
| Phellodendron chinense | MOL002651 | Dehydrotanshinone II A | CHRM4 |
| Phellodendron chinense | MOL002651 | Dehydrotanshinone II A | OPRD1 |
| Phellodendron chinense | MOL002651 | Dehydrotanshinone II A | ACHE |
| Phellodendron chinense | MOL002651 | Dehydrotanshinone II A | ADRA1A |
| Phellodendron chinense | MOL002651 | Dehydrotanshinone II A | ADRB2 |
| Phellodendron chinense | MOL002651 | Dehydrotanshinone II A | OPRM1 |
| Phellodendron chinense | MOL002651 | Dehydrotanshinone II A | DPP4 |
| Phellodendron chinense | MOL002651 | Dehydrotanshinone II A | CHRNA7 |
| Phellodendron chinense | MOL002651 | Dehydrotanshinone II A | NCOA1 |
| Phellodendron chinense | MOL002662 | rutaecarpine | PTGS1 |
| Phellodendron chinense | MOL002662 | rutaecarpine | AR |
| Phellodendron chinense | MOL002662 | rutaecarpine | SCN5A |
| Phellodendron chinense | MOL002662 | rutaecarpine | F10 |
| Phellodendron chinense | MOL002662 | rutaecarpine | PTGS2 |
| Phellodendron chinense | MOL002662 | rutaecarpine | RXRA |
| Phellodendron chinense | MOL002662 | rutaecarpine | CHEK1 |
| Phellodendron chinense | MOL002662 | rutaecarpine | PRKACA |
| Phellodendron chinense | MOL002662 | rutaecarpine | MMP2 |
| Phellodendron chinense | MOL002662 | rutaecarpine | MMP9 |
| Phellodendron chinense | MOL002662 | rutaecarpine | TNFAIP6 |
| Phellodendron chinense | MOL002662 | rutaecarpine | CYP3A4 |
| Phellodendron chinense | MOL002662 | rutaecarpine | IL4 |
| Phellodendron chinense | MOL002662 | rutaecarpine | CYP2B6 |
| Phellodendron chinense | MOL002663 | Skimmianin | RXRA |
| Phellodendron chinense | MOL002663 | Skimmianin | HSP90AA1 |
| Phellodendron chinense | MOL002663 | Skimmianin | PRKACA |
| Phellodendron chinense | MOL002666 | Chelerythrine | PTGS1 |
| Phellodendron chinense | MOL002666 | Chelerythrine | KCNH2 |
| Phellodendron chinense | MOL002666 | Chelerythrine | PTGS2 |
| Phellodendron chinense | MOL002666 | Chelerythrine | RXRA |
| Phellodendron chinense | MOL002666 | Chelerythrine | PRKACA |
| Phellodendron chinense | MOL002666 | Chelerythrine | NCOA2 |
| Phellodendron chinense | MOL000449 | Stigmasterol | PGR |
| Phellodendron chinense | MOL000449 | Stigmasterol | NR3C2 |
| Phellodendron chinense | MOL000449 | Stigmasterol | NCOA2 |
| Phellodendron chinense | MOL000449 | Stigmasterol | IGHG1 |
| Phellodendron chinense | MOL000449 | Stigmasterol | RXRA |
| Phellodendron chinense | MOL000449 | Stigmasterol | NCOA1 |
| Phellodendron chinense | MOL000449 | Stigmasterol | PTGS1 |
| Phellodendron chinense | MOL000449 | Stigmasterol | PTGS2 |
| Phellodendron chinense | MOL000449 | Stigmasterol | ADRA2A |
| Phellodendron chinense | MOL000449 | Stigmasterol | SLC6A2 |
| Phellodendron chinense | MOL000449 | Stigmasterol | SLC6A3 |
| Phellodendron chinense | MOL000449 | Stigmasterol | ADRB2 |
| Phellodendron chinense | MOL000449 | Stigmasterol | AKR1B1 |
| Phellodendron chinense | MOL000449 | Stigmasterol | PLAU |
| Phellodendron chinense | MOL000449 | Stigmasterol | LTA4H |
| Phellodendron chinense | MOL000449 | Stigmasterol | MAOB |
| Phellodendron chinense | MOL000449 | Stigmasterol | MAOA |
| Phellodendron chinense | MOL000449 | Stigmasterol | PRKACA |
| Phellodendron chinense | MOL000449 | Stigmasterol | CTRB1 |
| Phellodendron chinense | MOL000449 | Stigmasterol | CHRM3 |
| Phellodendron chinense | MOL000449 | Stigmasterol | CHRM1 |
| Phellodendron chinense | MOL000449 | Stigmasterol | ADRB1 |
| Phellodendron chinense | MOL000449 | Stigmasterol | SCN5A |
| Phellodendron chinense | MOL000449 | Stigmasterol | ADRA1A |
| Phellodendron chinense | MOL000449 | Stigmasterol | CHRM2 |
| Phellodendron chinense | MOL000449 | Stigmasterol | ADRA1B |
| Phellodendron chinense | MOL000449 | Stigmasterol | CHRNA7 |
| Phellodendron chinense | MOL002668 | Worenine | NOS2 |
| Phellodendron chinense | MOL002668 | Worenine | PTGS1 |
| Phellodendron chinense | MOL002668 | Worenine | ESR1 |
| Phellodendron chinense | MOL002668 | Worenine | AR |
| Phellodendron chinense | MOL002668 | Worenine | PTGS2 |
| Phellodendron chinense | MOL002668 | Worenine | CHEK1 |
| Phellodendron chinense | MOL002670 | Cavidine | PTGS1 |
| Phellodendron chinense | MOL002670 | Cavidine | CHRM3 |
| Phellodendron chinense | MOL002670 | Cavidine | KCNH2 |
| Phellodendron chinense | MOL002670 | Cavidine | CHRM1 |
| Phellodendron chinense | MOL002670 | Cavidine | ADRB1 |
| Phellodendron chinense | MOL002670 | Cavidine | SCN5A |
| Phellodendron chinense | MOL002670 | Cavidine | F10 |
| Phellodendron chinense | MOL002670 | Cavidine | CHRM5 |
| Phellodendron chinense | MOL002670 | Cavidine | PTGS2 |
| Phellodendron chinense | MOL002670 | Cavidine | ADRA2C |
| Phellodendron chinense | MOL002670 | Cavidine | CHRM4 |
| Phellodendron chinense | MOL002670 | Cavidine | RXRA |
| Phellodendron chinense | MOL002670 | Cavidine | OPRD1 |
| Phellodendron chinense | MOL002670 | Cavidine | ADRA1B |
| Phellodendron chinense | MOL002670 | Cavidine | ADRB2 |
| Phellodendron chinense | MOL002670 | Cavidine | ADRA1D |
| Phellodendron chinense | MOL002670 | Cavidine | TOP2A |
| Phellodendron chinense | MOL002670 | Cavidine | OPRM1 |
| Phellodendron chinense | MOL002670 | Cavidine | HSP90AA1 |
| Phellodendron chinense | MOL002670 | Cavidine | CAMKMT |
| Phellodendron chinense | MOL002670 | Cavidine | DRD1 |
| Phellodendron chinense | MOL002670 | Cavidine | SLC6A4 |
| Phellodendron chinense | MOL002670 | Cavidine | F7 |
| Phellodendron chinense | MOL002670 | Cavidine | PDE10A |
| Phellodendron chinense | MOL002672 | Hericenone H | F2 |
| Phellodendron chinense | MOL000358 | beta-sitosterol | PGR |
| Phellodendron chinense | MOL000358 | beta-sitosterol | NCOA2 |
| Phellodendron chinense | MOL000358 | beta-sitosterol | PTGS1 |
| Phellodendron chinense | MOL000358 | beta-sitosterol | PTGS2 |
| Phellodendron chinense | MOL000358 | beta-sitosterol | HSP90AA1 |
| Phellodendron chinense | MOL000358 | beta-sitosterol | KCNH2 |
| Phellodendron chinense | MOL000358 | beta-sitosterol | PRKACA |
| Phellodendron chinense | MOL000358 | beta-sitosterol | DRD1 |
| Phellodendron chinense | MOL000358 | beta-sitosterol | CHRM3 |
| Phellodendron chinense | MOL000358 | beta-sitosterol | CHRM1 |
| Phellodendron chinense | MOL000358 | beta-sitosterol | SCN5A |
| Phellodendron chinense | MOL000358 | beta-sitosterol | CHRM4 |
| Phellodendron chinense | MOL000358 | beta-sitosterol | PDE3A |
| Phellodendron chinense | MOL000358 | beta-sitosterol | ADRA1A |
| Phellodendron chinense | MOL000358 | beta-sitosterol | CHRM2 |
| Phellodendron chinense | MOL000358 | beta-sitosterol | ADRA1B |
| Phellodendron chinense | MOL000358 | beta-sitosterol | ADRB2 |
| Phellodendron chinense | MOL000358 | beta-sitosterol | CHRNA2 |
| Phellodendron chinense | MOL000358 | beta-sitosterol | SLC6A4 |
| Phellodendron chinense | MOL000358 | beta-sitosterol | OPRM1 |
| Phellodendron chinense | MOL000358 | beta-sitosterol | CHRNA7 |
| Phellodendron chinense | MOL000358 | beta-sitosterol | BCL2 |
| Phellodendron chinense | MOL000358 | beta-sitosterol | BAX |
| Phellodendron chinense | MOL000358 | beta-sitosterol | CASP9 |
| Phellodendron chinense | MOL000358 | beta-sitosterol | JUN |
| Phellodendron chinense | MOL000358 | beta-sitosterol | CASP3 |
| Phellodendron chinense | MOL000358 | beta-sitosterol | CASP8 |
| Phellodendron chinense | MOL000358 | beta-sitosterol | PRKCA |
| Phellodendron chinense | MOL000358 | beta-sitosterol | PON1 |
| Phellodendron chinense | MOL000358 | beta-sitosterol | MAP2 |
| Phellodendron chinense | MOL000622 | Magnograndiolide | GRIA2 |
| Phellodendron chinense | MOL000785 | palmatine | NOS2 |
| Phellodendron chinense | MOL000785 | palmatine | PTGS1 |
| Phellodendron chinense | MOL000785 | palmatine | KCNH2 |
| Phellodendron chinense | MOL000785 | palmatine | ESR1 |
| Phellodendron chinense | MOL000785 | palmatine | AR |
| Phellodendron chinense | MOL000785 | palmatine | SCN5A |
| Phellodendron chinense | MOL000785 | palmatine | PTGS2 |
| Phellodendron chinense | MOL000785 | palmatine | RXRA |
| Phellodendron chinense | MOL000785 | palmatine | ADRB2 |
| Phellodendron chinense | MOL000785 | palmatine | ESR2 |
| Phellodendron chinense | MOL000785 | palmatine | HSP90AA1 |
| Phellodendron chinense | MOL000785 | palmatine | PRSS1 |
| Phellodendron chinense | MOL000785 | palmatine | NCOA2 |
| Phellodendron chinense | MOL000785 | palmatine | CAMKMT |
| Phellodendron chinense | MOL000785 | palmatine | PRKACA |
| Phellodendron chinense | MOL000785 | palmatine | CDK2 |
| Phellodendron chinense | MOL000785 | palmatine | F7 |
| Coptis chinensis | MOL001454 | berberine | NOS2 |
| Coptis chinensis | MOL001454 | berberine | PTGS1 |
| Coptis chinensis | MOL001454 | berberine | KCNH2 |
| Coptis chinensis | MOL001454 | berberine | ESR1 |
| Coptis chinensis | MOL001454 | berberine | AR |
| Coptis chinensis | MOL001454 | berberine | SCN5A |
| Coptis chinensis | MOL001454 | berberine | PTGS2 |
| Coptis chinensis | MOL001454 | berberine | RXRA |
| Coptis chinensis | MOL001454 | berberine | ADRB2 |
| Coptis chinensis | MOL001454 | berberine | HSP90AB1 |
| Coptis chinensis | MOL001454 | berberine | PRSS1 |
| Coptis chinensis | MOL001454 | berberine | NCOA2 |
| Coptis chinensis | MOL001454 | berberine | PDE10A |
| Coptis chinensis | MOL001454 | berberine | CAMKMT |
| Coptis chinensis | MOL002894 | berberrubine | NOS2 |
| Coptis chinensis | MOL002894 | berberrubine | PTGS1 |
| Coptis chinensis | MOL002894 | berberrubine | KCNH2 |
| Coptis chinensis | MOL002894 | berberrubine | ESR1 |
| Coptis chinensis | MOL002894 | berberrubine | AR |
| Coptis chinensis | MOL002894 | berberrubine | SCN5A |
| Coptis chinensis | MOL002894 | berberrubine | PTGS2 |
| Coptis chinensis | MOL002894 | berberrubine | RXRA |
| Coptis chinensis | MOL002894 | berberrubine | PRSS1 |
| Coptis chinensis | MOL002894 | berberrubine | NCOA2 |
| Coptis chinensis | MOL002894 | berberrubine | CAMKMT |
| Coptis chinensis | MOL002897 | epiberberine | NOS2 |
| Coptis chinensis | MOL002897 | epiberberine | KCNH2 |
| Coptis chinensis | MOL002897 | epiberberine | ESR1 |
| Coptis chinensis | MOL002897 | epiberberine | AR |
| Coptis chinensis | MOL002897 | epiberberine | PTGS2 |
| Coptis chinensis | MOL002897 | epiberberine | RXRA |
| Coptis chinensis | MOL002897 | epiberberine | PRSS1 |
| Coptis chinensis | MOL002897 | epiberberine | NCOA2 |
| Coptis chinensis | MOL002897 | epiberberine | PDE10A |
| Coptis chinensis | MOL002903 | (R)-Canadine | PTGS1 |
| Coptis chinensis | MOL002903 | (R)-Canadine | CHRM3 |
| Coptis chinensis | MOL002903 | (R)-Canadine | KCNH2 |
| Coptis chinensis | MOL002903 | (R)-Canadine | CHRM1 |
| Coptis chinensis | MOL002903 | (R)-Canadine | SCN5A |
| Coptis chinensis | MOL002903 | (R)-Canadine | CHRM5 |
| Coptis chinensis | MOL002903 | (R)-Canadine | PTGS2 |
| Coptis chinensis | MOL002903 | (R)-Canadine | HTR3A |
| Coptis chinensis | MOL002903 | (R)-Canadine | ADRA2C |
| Coptis chinensis | MOL002903 | (R)-Canadine | CHRM4 |
| Coptis chinensis | MOL002903 | (R)-Canadine | OPRD1 |
| Coptis chinensis | MOL002903 | (R)-Canadine | ADRA1B |
| Coptis chinensis | MOL002903 | (R)-Canadine | SLC6A3 |
| Coptis chinensis | MOL002903 | (R)-Canadine | ADRB2 |
| Coptis chinensis | MOL002903 | (R)-Canadine | ADRA1D |
| Coptis chinensis | MOL002903 | (R)-Canadine | SLC6A4 |
| Coptis chinensis | MOL002903 | (R)-Canadine | OPRM1 |
| Coptis chinensis | MOL002903 | (R)-Canadine | HSP90AB1 |
| Coptis chinensis | MOL002903 | (R)-Canadine | PDE10A |
| Coptis chinensis | MOL002903 | (R)-Canadine | CAMKMT |
| Coptis chinensis | MOL002903 | (R)-Canadine | DRD1 |
| Coptis chinensis | MOL002903 | (R)-Canadine | DRD5 |
| Coptis chinensis | MOL002903 | (R)-Canadine | RXRA |
| Coptis chinensis | MOL002903 | (R)-Canadine | SLC6A2 |
| Coptis chinensis | MOL002903 | (R)-Canadine | ADRA1A |
| Coptis chinensis | MOL002903 | (R)-Canadine | CHRM2 |
| Coptis chinensis | MOL002904 | Berlambine | NOS2 |
| Coptis chinensis | MOL002904 | Berlambine | PTGS1 |
| Coptis chinensis | MOL002904 | Berlambine | CHRM3 |
| Coptis chinensis | MOL002904 | Berlambine | KCNH2 |
| Coptis chinensis | MOL002904 | Berlambine | AR |
| Coptis chinensis | MOL002904 | Berlambine | SCN5A |
| Coptis chinensis | MOL002904 | Berlambine | PTGS2 |
| Coptis chinensis | MOL002904 | Berlambine | F7 |
| Coptis chinensis | MOL002904 | Berlambine | RXRA |
| Coptis chinensis | MOL002904 | Berlambine | ADRA1B |
| Coptis chinensis | MOL002904 | Berlambine | ADRB2 |
| Coptis chinensis | MOL002904 | Berlambine | ADRA1D |
| Coptis chinensis | MOL002904 | Berlambine | HSP90AB1 |
| Coptis chinensis | MOL002904 | Berlambine | PRSS1 |
| Coptis chinensis | MOL002904 | Berlambine | NCOA2 |
| Coptis chinensis | MOL002904 | Berlambine | CAMKMT |
| Coptis chinensis | MOL002907 | Corchoroside A_qt | NR3C2 |
| Coptis chinensis | MOL002907 | Corchoroside A_qt | NCOA2 |
| Coptis chinensis | MOL000622 | Magnograndiolide | GABRA1 |
| Coptis chinensis | MOL000622 | Magnograndiolide | GRIA2 |
| Coptis chinensis | MOL000785 | palmatine | NOS2 |
| Coptis chinensis | MOL000785 | palmatine | PTGS1 |
| Coptis chinensis | MOL000785 | palmatine | KCNH2 |
| Coptis chinensis | MOL000785 | palmatine | ESR1 |
| Coptis chinensis | MOL000785 | palmatine | AR |
| Coptis chinensis | MOL000785 | palmatine | SCN5A |
| Coptis chinensis | MOL000785 | palmatine | PTGS2 |
| Coptis chinensis | MOL000785 | palmatine | RXRA |
| Coptis chinensis | MOL000785 | palmatine | ADRB2 |
| Coptis chinensis | MOL000785 | palmatine | ESR2 |
| Coptis chinensis | MOL000785 | palmatine | HSP90AB1 |
| Coptis chinensis | MOL000785 | palmatine | PRSS1 |
| Coptis chinensis | MOL000785 | palmatine | NCOA2 |
| Coptis chinensis | MOL000785 | palmatine | CAMKMT |
| Coptis chinensis | MOL000785 | palmatine | CDK2 |
| Coptis chinensis | MOL000785 | palmatine | F7 |
| Coptis chinensis | MOL000098 | quercetin | PTGS1 |
| Coptis chinensis | MOL000098 | quercetin | AR |
| Coptis chinensis | MOL000098 | quercetin | PPARG |
| Coptis chinensis | MOL000098 | quercetin | PTGS2 |
| Coptis chinensis | MOL000098 | quercetin | HSP90AB1 |
| Coptis chinensis | MOL000098 | quercetin | NCOA2 |
| Coptis chinensis | MOL000098 | quercetin | DPP4 |
| Coptis chinensis | MOL000098 | quercetin | AKR1B1 |
| Coptis chinensis | MOL000098 | quercetin | PRSS1 |
| Coptis chinensis | MOL000098 | quercetin | KCNH2 |
| Coptis chinensis | MOL000098 | quercetin | SCN5A |
| Coptis chinensis | MOL000098 | quercetin | ADRB2 |
| Coptis chinensis | MOL000098 | quercetin | AR |
| Coptis chinensis | MOL000098 | quercetin | PPARG |
| Coptis chinensis | MOL000098 | quercetin | PTGS2 |
| Coptis chinensis | MOL000098 | quercetin | HSP90AB1 |
| Coptis chinensis | MOL000098 | quercetin | NCOA2 |
| Coptis chinensis | MOL000098 | quercetin | DPP4 |
| Coptis chinensis | MOL000098 | quercetin | AKR1B1 |
| Coptis chinensis | MOL000098 | quercetin | PRSS1 |
| Coptis chinensis | MOL000098 | quercetin | KCNH2 |
| Coptis chinensis | MOL000098 | quercetin | SCN5A |
| Coptis chinensis | MOL000098 | quercetin | ADRB2 |
| Coptis chinensis | MOL000098 | quercetin | FOS |
| Coptis chinensis | MOL000098 | quercetin | CDKN1A |
| Coptis chinensis | MOL000098 | quercetin | EIF6 |
| Coptis chinensis | MOL000098 | quercetin | BAX |
| Coptis chinensis | MOL000098 | quercetin | CASP9 |
| Coptis chinensis | MOL000098 | quercetin | PLAU |
| Coptis chinensis | MOL000098 | quercetin | MMP2 |
| Coptis chinensis | MOL000098 | quercetin | MMP9 |
| Coptis chinensis | MOL000098 | quercetin | MAPK1 |
| Coptis chinensis | MOL000098 | quercetin | IL10RA |
| Coptis chinensis | MOL000098 | quercetin | EGF |
| Coptis chinensis | MOL000098 | quercetin | RB1 |
| Coptis chinensis | MOL000098 | quercetin | TNFAIP6 |
| Coptis chinensis | MOL000098 | quercetin | JUN |
| Coptis chinensis | MOL000098 | quercetin | IL6R |
| Coptis chinensis | MOL000098 | quercetin | AHSA1 |
| Coptis chinensis | MOL000098 | quercetin | CASP3 |
| Coptis chinensis | MOL000098 | quercetin | TP53 |
| Coptis chinensis | MOL000098 | quercetin | ELK1 |
| Coptis chinensis | MOL000098 | quercetin | NFKBIA |
| Coptis chinensis | MOL000098 | quercetin | POR |
| Coptis chinensis | MOL000098 | quercetin | ODC1 |
| Coptis chinensis | MOL000098 | quercetin | CASP8 |
| Coptis chinensis | MOL000098 | quercetin | TOP1 |
| Coptis chinensis | MOL000098 | quercetin | RAF1 |
| Coptis chinensis | MOL000098 | quercetin | SOD1 |
| Coptis chinensis | MOL000098 | quercetin | PRKCA |
| Coptis chinensis | MOL000098 | quercetin | MMP1 |
| Coptis chinensis | MOL000098 | quercetin | HIF1A |
| Coptis chinensis | MOL000098 | quercetin | STAT1 |
| Coptis chinensis | MOL000098 | quercetin | RUNX1T1 |
| Coptis chinensis | MOL000098 | quercetin | CDK1 |
| Coptis chinensis | MOL000098 | quercetin | HSPA5 |
| Coptis chinensis | MOL000098 | quercetin | ERBB2 |
| Coptis chinensis | MOL000098 | quercetin | PPARG |
| Coptis chinensis | MOL000098 | quercetin | ACACA |
| Coptis chinensis | MOL000098 | quercetin | HMOX1 |
| Coptis chinensis | MOL000098 | quercetin | CYP3A4 |
| Coptis chinensis | MOL000098 | quercetin | CYP1A2 |
| Coptis chinensis | MOL000098 | quercetin | CAV1 |
| Coptis chinensis | MOL000098 | quercetin | MYC |
| Coptis chinensis | MOL000098 | quercetin | F3 |
| Coptis chinensis | MOL000098 | quercetin | GJA1 |
| Coptis chinensis | MOL000098 | quercetin | CYP1A1 |
| Coptis chinensis | MOL000098 | quercetin | ICAM1 |
| Coptis chinensis | MOL000098 | quercetin | IL1B |
| Coptis chinensis | MOL000098 | quercetin | CCL2 |
| Coptis chinensis | MOL000098 | quercetin | SELE |
| Coptis chinensis | MOL000098 | quercetin | VCAM1 |
| Coptis chinensis | MOL000098 | quercetin | PTGER3 |
| Coptis chinensis | MOL000098 | quercetin | CXCL8 |
| Coptis chinensis | MOL000098 | quercetin | PRKCB |
| Coptis chinensis | MOL000098 | quercetin | BIRC5 |
| Coptis chinensis | MOL000098 | quercetin | DUOX2 |
| Coptis chinensis | MOL000098 | quercetin | NOS3 |
| Coptis chinensis | MOL000098 | quercetin | HSPB1 |
| Coptis chinensis | MOL000098 | quercetin | SULT1E1 |
| Coptis chinensis | MOL000098 | quercetin | IL2RA |
| Coptis chinensis | MOL000098 | quercetin | NR1I2 |
| Coptis chinensis | MOL000098 | quercetin | CYP1B1 |
| Coptis chinensis | MOL000098 | quercetin | CCNB1 |
| Coptis chinensis | MOL000098 | quercetin | PLAT |
| Coptis chinensis | MOL000098 | quercetin | THBD |
| Coptis chinensis | MOL000098 | quercetin | SERPINE1 |
| Coptis chinensis | MOL000098 | quercetin | COL1A1 |
| Coptis chinensis | MOL000098 | quercetin | IFNG |
| Coptis chinensis | MOL000098 | quercetin | ALOX5 |
| Coptis chinensis | MOL000098 | quercetin | IL1A |
| Coptis chinensis | MOL000098 | quercetin | MPO |
| Coptis chinensis | MOL000098 | quercetin | TOP2A |
| Coptis chinensis | MOL000098 | quercetin | NCF1 |
| Coptis chinensis | MOL000098 | quercetin | ABCG2 |
| Coptis chinensis | MOL000098 | quercetin | HAS2 |
| Coptis chinensis | MOL000098 | quercetin | GSTP1 |
| Coptis chinensis | MOL000098 | quercetin | NFE2L2 |
| Coptis chinensis | MOL000098 | quercetin | NQO1 |
| Coptis chinensis | MOL000098 | quercetin | PARP1 |
| Coptis chinensis | MOL000098 | quercetin | AHR |
| Coptis chinensis | MOL000098 | quercetin | PSMD3 |
| Coptis chinensis | MOL000098 | quercetin | SLC2A4 |
| Coptis chinensis | MOL000098 | quercetin | COL3A1 |
| Coptis chinensis | MOL000098 | quercetin | CXCL11 |
| Coptis chinensis | MOL000098 | quercetin | CXCL2 |
| Coptis chinensis | MOL000098 | quercetin | DCAF5 |
| Coptis chinensis | MOL000098 | quercetin | NR1I3 |
| Coptis chinensis | MOL000098 | quercetin | CHEK2 |
| Coptis chinensis | MOL000098 | quercetin | INSRR |
| Coptis chinensis | MOL000098 | quercetin | CLDN4 |
| Coptis chinensis | MOL000098 | quercetin | PPARA |
| Coptis chinensis | MOL000098 | quercetin | PPARD |
| Coptis chinensis | MOL000098 | quercetin | HSF1 |
| Coptis chinensis | MOL000098 | quercetin | CXCL10 |
| Coptis chinensis | MOL000098 | quercetin | CHUK |
| Coptis chinensis | MOL000098 | quercetin | SPP1 |
| Coptis chinensis | MOL000098 | quercetin | RUNX2 |
| Coptis chinensis | MOL000098 | quercetin | RASSF1 |
| Coptis chinensis | MOL000098 | quercetin | E2F1 |
| Coptis chinensis | MOL000098 | quercetin | E2F2 |
| Coptis chinensis | MOL000098 | quercetin | ACP3 |
| Coptis chinensis | MOL000098 | quercetin | CTSD |
| Coptis chinensis | MOL000098 | quercetin | IGFBP3 |
| Coptis chinensis | MOL000098 | quercetin | IGF2 |
| Coptis chinensis | MOL000098 | quercetin | CD40LG |
| Coptis chinensis | MOL000098 | quercetin | IRF1 |
| Coptis chinensis | MOL000098 | quercetin | ERBB3 |
| Coptis chinensis | MOL000098 | quercetin | PON1 |
| Coptis chinensis | MOL000098 | quercetin | DIO1 |
| Coptis chinensis | MOL000098 | quercetin | PCOLCE |
| Coptis chinensis | MOL000098 | quercetin | NPEPPS |
| Coptis chinensis | MOL000098 | quercetin | HK2 |
| Coptis chinensis | MOL000098 | quercetin | RASA1 |
| Coptis chinensis | MOL000098 | quercetin | GSTM1 |
| Coptis chinensis | MOL000098 | quercetin | GSTM2 |
| Coptis chinensis | MOL001458 | coptisine | NOS2 |
| Coptis chinensis | MOL001458 | coptisine | PTGS1 |
| Coptis chinensis | MOL001458 | coptisine | KCNH2 |
| Coptis chinensis | MOL001458 | coptisine | ESR1 |
| Coptis chinensis | MOL001458 | coptisine | AR |
| Coptis chinensis | MOL001458 | coptisine | SCN5A |
| Coptis chinensis | MOL001458 | coptisine | PTGS2 |
| Coptis chinensis | MOL001458 | coptisine | PRSS1 |
| Coptis chinensis | MOL002668 | Worenine | NOS2 |
| Coptis chinensis | MOL002668 | Worenine | PTGS1 |
| Coptis chinensis | MOL002668 | Worenine | ESR1 |
| Coptis chinensis | MOL002668 | Worenine | AR |
| Coptis chinensis | MOL002668 | Worenine | PTGS2 |
| Coptis chinensis | MOL002668 | Worenine | CHEK1 |
| Cortex fraxini | MOL000358 | beta-sitosterol | PGR |
| Cortex fraxini | MOL000358 | beta-sitosterol | NCOA2 |
| Cortex fraxini | MOL000358 | beta-sitosterol | PTGS1 |
| Cortex fraxini | MOL000358 | beta-sitosterol | PTGS2 |
| Cortex fraxini | MOL000358 | beta-sitosterol | HSP90AA1 |
| Cortex fraxini | MOL000358 | beta-sitosterol | KCNH2 |
| Cortex fraxini | MOL000358 | beta-sitosterol | PRKACA |
| Cortex fraxini | MOL000358 | beta-sitosterol | DRD1 |
| Cortex fraxini | MOL000358 | beta-sitosterol | CHRM3 |
| Cortex fraxini | MOL000358 | beta-sitosterol | CHRM1 |
| Cortex fraxini | MOL000358 | beta-sitosterol | SCN5A |
| Cortex fraxini | MOL000358 | beta-sitosterol | CHRM4 |
| Cortex fraxini | MOL000358 | beta-sitosterol | PDE3A |
| Cortex fraxini | MOL000358 | beta-sitosterol | ADRA1A |
| Cortex fraxini | MOL000358 | beta-sitosterol | CHRM2 |
| Cortex fraxini | MOL000358 | beta-sitosterol | ADRA1B |
| Cortex fraxini | MOL000358 | beta-sitosterol | ADRB2 |
| Cortex fraxini | MOL000358 | beta-sitosterol | CHRNA2 |
| Cortex fraxini | MOL000358 | beta-sitosterol | SLC6A4 |
| Cortex fraxini | MOL000358 | beta-sitosterol | OPRM1 |
| Cortex fraxini | MOL000358 | beta-sitosterol | CHRNA7 |
| Cortex fraxini | MOL000358 | beta-sitosterol | BCL2 |
| Cortex fraxini | MOL000358 | beta-sitosterol | BAX |
| Cortex fraxini | MOL000358 | beta-sitosterol | CASP9 |
| Cortex fraxini | MOL000358 | beta-sitosterol | JUN |
| Cortex fraxini | MOL000358 | beta-sitosterol | CASP3 |
| Cortex fraxini | MOL000358 | beta-sitosterol | CASP8 |
| Cortex fraxini | MOL000358 | beta-sitosterol | PRKCA |
| Cortex fraxini | MOL000358 | beta-sitosterol | PON1 |
| Cortex fraxini | MOL000358 | beta-sitosterol | MAP2 |
| Cortex fraxini | MOL006709 | AIDS214634 | PTGS2 |
| Cortex fraxini | MOL006709 | AIDS214634 | NCOA2 |
| Cortex fraxini | MOL006709 | AIDS214634 | CAMKMT |
| Cortex fraxini | MOL006709 | AIDS214634 | KCNH2 |
| Cortex fraxini | MOL006709 | AIDS214634 | F10 |
| Cortex fraxini | MOL006709 | AIDS214634 | F7 |
| Cortex fraxini | MOL006710 | 8-(beta-D-Glucopyranosyloxy)-7-hydroxy-6-methoxy-2H-1-benzopyran-2-one | F2 |
| Cortex fraxini | MOL006710 | 8-(beta-D-Glucopyranosyloxy)-7-hydroxy-6-methoxy-2H-1-benzopyran-2-one | PTGS2 |
| Cortex fraxini | MOL006710 | 8-(beta-D-Glucopyranosyloxy)-7-hydroxy-6-methoxy-2H-1-benzopyran-2-one | PTPN1 |
| Cortex fraxini | MOL006710 | 8-(beta-D-Glucopyranosyloxy)-7-hydroxy-6-methoxy-2H-1-benzopyran-2-one | TOP2A |
| Cortex fraxini | MOL006710 | 8-(beta-D-Glucopyranosyloxy)-7-hydroxy-6-methoxy-2H-1-benzopyran-2-one | PRSS1 |
