# Supplementary material for: Network Pharmacology-Based Strategy to Identify the Pharmacological Mechanisms of Pulsatilla Decoction against Crohn’s Disease
Source: Front Pharmacol. 2022 Apr 5;13:844685. doi: 10.3389/fphar.2022.844685 (PMC9016333; doi:10.3389/fphar.2022.844685)
Supplement: Supplementary file 1 [file DataSheet1.zip › Table (3).DOCX]

**Supplemental Table 3. Disease-related genes extracted from five databases**

| GeneCards | OMIM | TTD | PharmGkb | DrugBank |
| --- | --- | --- | --- | --- |
| NOD2 | ABCA1 | PDF | CTD | TPMT |
| IL6 | ABCA4 | INSR | SH2D1A | XDH |
| TNF | ABCB1 | CNR2 | GAA | GSTA1 |
| IL10 | ABCB4 | JAK3 | OED | GSTA2 |
| PKHD1 | ABCG8 | S1PR1 | MYMY1 | GSTM1 |
| GBA | ACTA2 | ITGB7 | AD5 | RAC1 |
| APOE | ADAM17 | IL12A | MYMY3 | CYP1A2 |
| LRRK2 | ADH1C | ICAM1 | PDB4 | HPRT1 |
| LMNA | AGL | PDE4A | PDB1 | NUDT15 |
| CTLA4 | APC | ITGA4 | PDB5 | SLC29A2 |
| APP | ARPC1B | IL6 | PDB6 | SLC28A3 |
| PRKCQ | ATG16L1 | JAK1 | IBD3 | ABCB1 |
| TGFB1 | ATP1A1 | PDE4D | IBD5 | SLC43A3 |
| HLA-DRB1 | ATP2C1 | TNF | IBD2 | ALB |
| TP53 | BCKDHB | GUCY2C | IBD4 | IMPDH1 |
| PRKN | BMPR2 | ICAM1 | IBD6 | IMPDH2 |
| RET | CALHM1 | IL23A | IBD7 | GMPS |
| IL23R | CAV3 | IL12B | IBD9 | ITPA |
| IL1B | CD36 | CELA1 | IBD8 | NR3C1 |
| VWF | CHCHD2 | ITGA4-ITGB7 | RMMD1 | CYP3A4 |
| TLR4 | COPA | MMP9 | CELIAC2 | CYP3A5 |
| IL10RA | CRB2 | ELANE | PARK10 | ABCB11 |
| MAPT | CTLA4 | CTSS | PKD3 | ANXA1 |
| IFNG | CX3CR1 | PDE4B | KWE | CYP2A6 |
| VCP | DBT | CD40 | MYP1 | CYP1B1 |
| CRP | DHTKD1 | CCR9 | PARK3 | CYP2B6 |
| PARK7 | DHX16 | ITGA4-ITGB1 | CCLA1 | CYP2C8 |
| STAT3 | DNAJB11 | MADCAM1 | PKD1P6 | CYP2C9 |
| ATG16L1 | DNAJC6 | CXCL10 | PKD1P5 | CYP2C19 |
| HLA-B | DZIP1L | IL21R | PKD1P4 | SERPINA6 |
| PTPN22 | ECE1 | SMAD7 | PKD1P3 | SLCO1A2 |
| CYBB | ECM1 | RORC | PKD1P2 | SLC22A8 |
| MPO | EGFR | KLRK1 | SLC2A4RG | SHBG |
| MEFV | EGR2 | Bact FimH | JCAD | CYP3A7 |
| ACE | EIF4G1 | TNF | SLC25A16 | CYP11B1 |
| HLA-DQB1 | ELOVL4 |  | GATD1 | CYP11B2 |
| CCL2 | ENPP1 |  | GBE1 | AKR1D1 |
| ALB | FARSB |  | CCDC180 | SRD5A2 |
| CXCL8 | FIG4 |  | PKHD1L1 | HSD11B2 |
| ABCA1 | FOXD3 |  | PKD1P1 | HSD11B1 |
| TLR2 | GARS1 |  | PYGL | ABCC4 |
| JAK2 | GATA4 |  | PKD1L1 | ABCC5 |
| CFTR | GBA |  | NAGLU | SLC28A2 |
| DNM2 | GBE1 |  | G6PC | SLC29A1 |
| CCR6 | GDAP1 |  | PKDREJ | AOX1 |
| SLC17A5 | GDNF |  | PKD2L2 | PPAT |
| NAGLU | GIGYF2 |  | PKD1L2 | PTGS1 |
| NOS3 | GLE1 |  | GNS | PTGS2 |
| TNFRSF1A | GNB4 |  | ASPA | PPARG |
| IL4 | GYG1 |  | F9 | CHUK |
| MIR21 | HARS1 |  | SLC6A19 | IKBKB |
| GDNF | HEXB |  | GALC | ALOX5 |
| MT-ND1 | HFE |  | PKD1L3 | MPO |
| EDNRB | HLA-DPB1 |  | PKD2L1 | nat |
| EGR2 | HLA-DQA1 |  | PEX7 | NAT1 |
| IRGM | HLA-DQB1 |  | ABCG8 | DHFR |
| IL2 | HNRNPA2B1 |  | PSPA | MTHFR |
| GFAP | HOXD10 |  | NPC2 | ABCC3 |
| PTEN | HSPB1 |  | BEST1 | ABCC1 |
| SERPINA1 | HTRA2 |  | MRE11 | SLC22A6 |
| SOD1 | HTT |  | CLCN1 | ABCC10 |
| MTHFR | IL10 |  | NDP | ABCC2 |
| NDRG1 | IL23R |  | PKHD1 | SLC16A1 |
| CTNNB1 | IL37 |  | EPM2A | ABCC11 |
| IL17A | IL6 |  | ABCA1 | SLCO1B3 |
| ICAM1 | INAVA |  | ATP7B | SLC22A11 |
| HSPB1 | IRF5 |  | PLP1 | SLCO1C1 |
| TTR | IRGM |  | PHYH | SLCO3A1 |
| NCF2 | IRS1 |  | LIPA | ABCG2 |
| LAMP2 | JPH1 |  | ABCA4 | SLC22A7 |
| UMOD | KIF1B |  | NCF1 | SLCO1B1 |
| MMP1 | LIPA |  | NCF2 | PGD |
| CLCN5 | LMNA |  | BCKDHB | SLC46A1 |
| LRP5 | LPA |  | PYGM | FPGS |
| NLRP3 | LRSAM1 |  | NF1 | GGH |
| LITAF | MFN2 |  | NPC1 | TYMS |
| CLN3 | MME |  | BCKDHA | ATIC |
| PPARG | MPV17 |  | HEXA | SLCO4C1 |
| IL18 | MPZ |  | AGL | SLC19A1 |
| FAS | MTHFR |  | CLCN5 | FOLR1 |
| MUC1 | MUC1 |  | PKD1 | FOLR2 |
| HLA-DQA1 | NCF1 |  | PRNP | SLC15A1 |
| IL21 | NCF2 |  | PKD2 | SLC36A1 |
| F9 | NDRG1 |  | GLRA1 | ACAT1 |
| APC | NEFL |  | AR | SLC7A11 |
| KRAS | NOS3 |  | PSEN2 | TBXAS1 |
| TNFRSF11A | NOTCH1 |  | TGFB1 | PLA2G1B |
| VEGFA | NOTCH2NLC |  |  | SLCO2B1 |
| APOA1 | PARK7 |  |  | BCHE |
| SLC22A4 | PDCD1 |  |  |  |
| SLC37A4 | PDE11A |  |  |  |
| EGFR | PDE8B |  |  |  |
| ABCB1 | PDZD7 |  |  |  |
| INS | PGAM2 |  |  |  |
| IL2RA | PHOX2B |  |  |  |
| MIR146A | PHYH |  |  |  |
| TNFRSF11B | PINK1 |  |  |  |
| F2 | PKD2 |  |  |  |
| AR | PKHD1 |  |  |  |
| BRAF | PLAU |  |  |  |
| IL1A | PLEKHG5 |  |  |  |
| SLC22A5 | PMP2 |  |  |  |
| ADAM17 | POGLUT1 |  |  |  |
| GALC | PON1 |  |  |  |
| IL1RN | PON2 |  |  |  |
| PIK3CA | PPM1K |  |  |  |
| IL13 | PRKAG2 |  |  |  |
| MME | PRKN |  |  |  |
| PON1 | PROM1 |  |  |  |
| CD4 | PSAP |  |  |  |
| NOTCH1 | PSEN2 |  |  |  |
| UCHL1 | RAB7A |  |  |  |
| AKT1 | REN |  |  |  |
| BDNF | RET |  |  |  |
| TNFRSF1B | RHCE |  |  |  |
| TLR5 | RHD |  |  |  |
| BRCA2 | SAG |  |  |  |
| MIR34A | SAR1B |  |  |  |
| SMAD4 | SEC61A1 |  |  |  |
| STAT4 | SEC63 |  |  |  |
| PROM1 | SFRP4 |  |  |  |
| GATA3 | SH3TC2 |  |  |  |
| NCF4 | SLC17A5 |  |  |  |
| IBD5 | SLC25A16 |  |  |  |
| CDH1 | SLURP1 |  |  |  |
| MMP9 | SNCA |  |  |  |
| SLC11A1 | SP110 |  |  |  |
| JUP | SQSTM1 |  |  |  |
| FOXP3 | SURF1 |  |  |  |
| MIR17 | TBP |  |  |  |
| ICOSLG | TG |  |  |  |
| CFH | TLR5 |  |  |  |
| IRF5 | TNFRSF11B |  |  |  |
| ATXN2 | TNNI3K |  |  |  |
| ACTA2 | TRIM2 |  |  |  |
| COL1A1 | UCHL1 |  |  |  |
| HP | VCP |  |  |  |
| SP140 | YARS1 |  |  |  |
| INAVA | ZAP70 |  |  |  |
| TNFSF15 | ZFAT |  |  |  |
| SAG | ZNF687 |  |  |  |
| JAG1 |  |  |  |  |
| IL10RB |  |  |  |  |
| NF1 |  |  |  |  |
| SERPINA3 |  |  |  |  |
| DSP |  |  |  |  |
| PFKM |  |  |  |  |
| ATM |  |  |  |  |
| CD40 |  |  |  |  |
| TTN |  |  |  |  |
| PTPN11 |  |  |  |  |
| IL12B |  |  |  |  |
| APOB |  |  |  |  |
| MIR145 |  |  |  |  |
| OCRL |  |  |  |  |
| MT-CYB |  |  |  |  |
| MIR155 |  |  |  |  |
| STAT1 |  |  |  |  |
| MMP3 |  |  |  |  |
| PLAU |  |  |  |  |
| F5 |  |  |  |  |
| TPMT |  |  |  |  |
| SMAD3 |  |  |  |  |
| ERBB2 |  |  |  |  |
| CCR5 |  |  |  |  |
| MIR29A |  |  |  |  |
| FCGR2A |  |  |  |  |
| GRN |  |  |  |  |
| HLA-A |  |  |  |  |
| KIT |  |  |  |  |
| PSTPIP1 |  |  |  |  |
| VDR |  |  |  |  |
| BRCA1 |  |  |  |  |
| NFKBIA |  |  |  |  |
| FGFR3 |  |  |  |  |
| NOS2 |  |  |  |  |
| IGF1 |  |  |  |  |
| HMOX1 |  |  |  |  |
| REN |  |  |  |  |
| MAPK1 |  |  |  |  |
| CAV3 |  |  |  |  |
| SERPINE1 |  |  |  |  |
| GM2A |  |  |  |  |
| CD36 |  |  |  |  |
| FN1 |  |  |  |  |
| MT-CO1 |  |  |  |  |
| CCL5 |  |  |  |  |
| MIR143 |  |  |  |  |
| BEST1 |  |  |  |  |
| MIR126 |  |  |  |  |
| CCND1 |  |  |  |  |
| PLA2G6 |  |  |  |  |
| NFKB1 |  |  |  |  |
| CSF2 |  |  |  |  |
| CYP27A1 |  |  |  |  |
| OPTN |  |  |  |  |
| CTSD |  |  |  |  |
| COL17A1 |  |  |  |  |
| IBD21 |  |  |  |  |
| COL7A1 |  |  |  |  |
| ITGA4 |  |  |  |  |
| CASR |  |  |  |  |
| HNRNPA1 |  |  |  |  |
| CD40LG |  |  |  |  |
| HSPD1 |  |  |  |  |
| ESR1 |  |  |  |  |
| LEP |  |  |  |  |
| CARD8 |  |  |  |  |
| TGFBR2 |  |  |  |  |
| TLR9 |  |  |  |  |
| S100A9 |  |  |  |  |
| FCGR3A |  |  |  |  |
| RIPK1 |  |  |  |  |
| MIR15A |  |  |  |  |
| DLD |  |  |  |  |
| AGT |  |  |  |  |
| GIGYF2 |  |  |  |  |
| SURF1 |  |  |  |  |
| MIF |  |  |  |  |
| MIR29B1 |  |  |  |  |
| MMP2 |  |  |  |  |
| HRAS |  |  |  |  |
| FLNA |  |  |  |  |
| GNAS |  |  |  |  |
| LACC1 |  |  |  |  |
| NR4A2 |  |  |  |  |
| COMT |  |  |  |  |
| DCTN1 |  |  |  |  |
| TBK1 |  |  |  |  |
| FGFR2 |  |  |  |  |
| CHAT |  |  |  |  |
| FASLG |  |  |  |  |
| B2M |  |  |  |  |
| INPP5E |  |  |  |  |
| IBD3 |  |  |  |  |
| IBD2 |  |  |  |  |
| TH |  |  |  |  |
| GATA6 |  |  |  |  |
| PTPN2 |  |  |  |  |
| HLA-DPB1 |  |  |  |  |
| HLA-C |  |  |  |  |
| SP110 |  |  |  |  |
| COPA |  |  |  |  |
| VPS13C |  |  |  |  |
| CP |  |  |  |  |
| IBD7 |  |  |  |  |
| CRB1 |  |  |  |  |
| CDKN2A |  |  |  |  |
| RHO |  |  |  |  |
| EPO |  |  |  |  |
| ELANE |  |  |  |  |
| CASP1 |  |  |  |  |
| MBL2 |  |  |  |  |
| ENPP1 |  |  |  |  |
| MIR106B |  |  |  |  |
| ENG |  |  |  |  |
| LCAT |  |  |  |  |
| DMD |  |  |  |  |
| A2M |  |  |  |  |
| EP300 |  |  |  |  |
| MIR140 |  |  |  |  |
| CASP3 |  |  |  |  |
| LTA |  |  |  |  |
| CARD9 |  |  |  |  |
| CYBC1 |  |  |  |  |
| TERT |  |  |  |  |
| NRAS |  |  |  |  |
| CX3CR1 |  |  |  |  |
| NLRP1 |  |  |  |  |
| HARS1 |  |  |  |  |
| IBD15 |  |  |  |  |
| PRTN3 |  |  |  |  |
| IBD18 |  |  |  |  |
| PTGS2 |  |  |  |  |
| IL5 |  |  |  |  |
| IBD6 |  |  |  |  |
| C4A |  |  |  |  |
| COL1A2 |  |  |  |  |
| SLC34A1 |  |  |  |  |
| MIR132 |  |  |  |  |
| IBD4 |  |  |  |  |
| ADIPOQ |  |  |  |  |
| MTOR |  |  |  |  |
| SPP1 |  |  |  |  |
| ITCH |  |  |  |  |
| CCL11 |  |  |  |  |
| CST3 |  |  |  |  |
| FLG |  |  |  |  |
| IL17F |  |  |  |  |
| CASP8 |  |  |  |  |
| IL23A |  |  |  |  |
| CAT |  |  |  |  |
| CEACAM6 |  |  |  |  |
| BBS1 |  |  |  |  |
| DLG5 |  |  |  |  |
| SBF1 |  |  |  |  |
| PDCD1 |  |  |  |  |
| PHOX2B |  |  |  |  |
| IL12RB1 |  |  |  |  |
| NOD1 |  |  |  |  |
| ACP5 |  |  |  |  |
| BGLAP |  |  |  |  |
| FGFR1 |  |  |  |  |
| NTRK1 |  |  |  |  |
| GPT |  |  |  |  |
| CREBBP |  |  |  |  |
| TGFB2 |  |  |  |  |
| ADAM10 |  |  |  |  |
| POMC |  |  |  |  |
| IFT172 |  |  |  |  |
| S100A8 |  |  |  |  |
| IBD8 |  |  |  |  |
| SERPINC1 |  |  |  |  |
| EDN1 |  |  |  |  |
| MLH1 |  |  |  |  |
| AIRE |  |  |  |  |
| IBD9 |  |  |  |  |
| LTF |  |  |  |  |
| FGF23 |  |  |  |  |
| ALMS1 |  |  |  |  |
| AIFM1 |  |  |  |  |
| RYR2 |  |  |  |  |
| MSH2 |  |  |  |  |
| C3 |  |  |  |  |
| PON2 |  |  |  |  |
| MIR210 |  |  |  |  |
| BTNL2 |  |  |  |  |
| CD28 |  |  |  |  |
| ABL1 |  |  |  |  |
| IBD16 |  |  |  |  |
| ZEB2 |  |  |  |  |
| SGSH |  |  |  |  |
| MYC |  |  |  |  |
| IL1R1 |  |  |  |  |
| ERCC6 |  |  |  |  |
| MTM1 |  |  |  |  |
| MIR122 |  |  |  |  |
| THBD |  |  |  |  |
| CACNA1A |  |  |  |  |
| IBD11 |  |  |  |  |
| IBD12 |  |  |  |  |
| IBD20 |  |  |  |  |
| IBD22 |  |  |  |  |
| IBD23 |  |  |  |  |
| IBD24 |  |  |  |  |
| IBD26 |  |  |  |  |
| IBD27 |  |  |  |  |
| HNF4A |  |  |  |  |
| ADA |  |  |  |  |
| MIR223 |  |  |  |  |
| NKX2-3 |  |  |  |  |
| IL22 |  |  |  |  |
| IBD25 |  |  |  |  |
| ACTB |  |  |  |  |
| MAP2K1 |  |  |  |  |
| ITGB3 |  |  |  |  |
| IGF2 |  |  |  |  |
| RUNX1 |  |  |  |  |
| SOS1 |  |  |  |  |
| WFS1 |  |  |  |  |
| IL18RAP |  |  |  |  |
| MSH6 |  |  |  |  |
| TGFBR1 |  |  |  |  |
| ADAMTS13 |  |  |  |  |
| FUT2 |  |  |  |  |
| GUCY2D |  |  |  |  |
| IL12A |  |  |  |  |
| TCF4 |  |  |  |  |
| DEFB4A |  |  |  |  |
| GCG |  |  |  |  |
| USH2A |  |  |  |  |
| IFNGR1 |  |  |  |  |
| SELE |  |  |  |  |
| TNFSF11 |  |  |  |  |
| TNFAIP3 |  |  |  |  |
| NR1H4 |  |  |  |  |
| EDNRA |  |  |  |  |
| SLC2A1 |  |  |  |  |
| ITGAM |  |  |  |  |
| IL6R |  |  |  |  |
| HIF1A |  |  |  |  |
| PMS2 |  |  |  |  |
| LRP6 |  |  |  |  |
| FGF2 |  |  |  |  |
| CAV1 |  |  |  |  |
| EGF |  |  |  |  |
| COL3A1 |  |  |  |  |
| BACH2 |  |  |  |  |
| BAX |  |  |  |  |
| CXCR4 |  |  |  |  |
| IFNA1 |  |  |  |  |
| F13A1 |  |  |  |  |
| IL12RB2 |  |  |  |  |
| MT-CO2 |  |  |  |  |
| MT-ND4L |  |  |  |  |
| PPARGC1A |  |  |  |  |
| TF |  |  |  |  |
| NCF4-AS1 |  |  |  |  |
| H2AC18 |  |  |  |  |
| CD79A |  |  |  |  |
| G6PD |  |  |  |  |
| CD14 |  |  |  |  |
| TIMP1 |  |  |  |  |
| GUCY2C |  |  |  |  |
| ZAP70 |  |  |  |  |
| KMT2A |  |  |  |  |
| BBS12 |  |  |  |  |
| ERBB4 |  |  |  |  |
| GSTM1 |  |  |  |  |
| PDGFRB |  |  |  |  |
| VCAM1 |  |  |  |  |
| S100A12 |  |  |  |  |
| CHEK2 |  |  |  |  |
| PIK3R1 |  |  |  |  |
| SELP |  |  |  |  |
| PTCH1 |  |  |  |  |
| INSR |  |  |  |  |
| MYO9B |  |  |  |  |
| MIR125A |  |  |  |  |
| PTH |  |  |  |  |
| TYK2 |  |  |  |  |
| DYRK1A |  |  |  |  |
| F3 |  |  |  |  |
| GUSB |  |  |  |  |
| ABCG8 |  |  |  |  |
| RAF1 |  |  |  |  |
| PTPRC |  |  |  |  |
| CCL3 |  |  |  |  |
| AGER |  |  |  |  |
| CXCL12 |  |  |  |  |
| CD8A |  |  |  |  |
| SOD2 |  |  |  |  |
| BTK |  |  |  |  |
| LDHA |  |  |  |  |
| FZD4 |  |  |  |  |
| CFI |  |  |  |  |
| XIAP |  |  |  |  |
| HLA-G |  |  |  |  |
| ERAP1 |  |  |  |  |
| CSF3 |  |  |  |  |
| TEK |  |  |  |  |
| FGA |  |  |  |  |
| RBP4 |  |  |  |  |
| IKBKG |  |  |  |  |
| SERPINH1 |  |  |  |  |
| RIPK2 |  |  |  |  |
| RETN |  |  |  |  |
| EIF4G1 |  |  |  |  |
| PTGER4 |  |  |  |  |
| MIR29C |  |  |  |  |
| SOX9 |  |  |  |  |
| SDHA |  |  |  |  |
| CXCL10 |  |  |  |  |
| CD19 |  |  |  |  |
| SHH |  |  |  |  |
| MIR107 |  |  |  |  |
| PNPLA3 |  |  |  |  |
| GAPDH |  |  |  |  |
| STAT5B |  |  |  |  |
| MTR |  |  |  |  |
| SATB2 |  |  |  |  |
| NR3C1 |  |  |  |  |
| NOTCH2 |  |  |  |  |
| F12 |  |  |  |  |
| INPP5D |  |  |  |  |
| HAMP |  |  |  |  |
| MIR144 |  |  |  |  |
| IL21-AS1 |  |  |  |  |
| IL2RB |  |  |  |  |
| NGF |  |  |  |  |
| MST1 |  |  |  |  |
| CEP43 |  |  |  |  |
| NSD1 |  |  |  |  |
| IFIH1 |  |  |  |  |
| IL18BP |  |  |  |  |
| PLA2G2A |  |  |  |  |
| CCN2 |  |  |  |  |
| DRD2 |  |  |  |  |
| SLC6A4 |  |  |  |  |
| DES |  |  |  |  |
| HTRA1 |  |  |  |  |
| GSTP1 |  |  |  |  |
| KIF5A |  |  |  |  |
| TLR1 |  |  |  |  |
| IL7R |  |  |  |  |
| GHRL |  |  |  |  |
| TLR3 |  |  |  |  |
| CLU |  |  |  |  |
| CCR1 |  |  |  |  |
| ACTC1 |  |  |  |  |
| IGF1R |  |  |  |  |
| XDH |  |  |  |  |
| MYD88 |  |  |  |  |
| CACNA1C |  |  |  |  |
| POFUT1 |  |  |  |  |
| SRC |  |  |  |  |
| GSK3B |  |  |  |  |
| CALR |  |  |  |  |
| MIR328 |  |  |  |  |
| TFRC |  |  |  |  |
| FCGR3B |  |  |  |  |
| BMP2 |  |  |  |  |
| ACHE |  |  |  |  |
| SYK |  |  |  |  |
| MIR203A |  |  |  |  |
| ERCC2 |  |  |  |  |
| UGT1A1 |  |  |  |  |
| TOR1A |  |  |  |  |
| CFHR2 |  |  |  |  |
| ITGAX |  |  |  |  |
| IRS1 |  |  |  |  |
| MUTYH |  |  |  |  |
| MIR192 |  |  |  |  |
| SST |  |  |  |  |
| IFNGR2 |  |  |  |  |
| CYP2D6 |  |  |  |  |
| ALK |  |  |  |  |
| LRP1 |  |  |  |  |
| CACNA1S |  |  |  |  |
| HPRT1 |  |  |  |  |
| ACTA2-AS1 |  |  |  |  |
| HSPA2 |  |  |  |  |
| CD209 |  |  |  |  |
| IRF1 |  |  |  |  |
| TGFB3 |  |  |  |  |
| CALCA |  |  |  |  |
| LRBA |  |  |  |  |
| TMEM106B |  |  |  |  |
| ITGB2 |  |  |  |  |
| MIR141 |  |  |  |  |
| GMPPB |  |  |  |  |
| LOX |  |  |  |  |
| MIR20A |  |  |  |  |
| IL15 |  |  |  |  |
| UBE2L3 |  |  |  |  |
| MMP13 |  |  |  |  |
| FCGR2B |  |  |  |  |
| PKD2L1 |  |  |  |  |
| IL4R |  |  |  |  |
| PECAM1 |  |  |  |  |
| MYLK |  |  |  |  |
| ALPL |  |  |  |  |
| RUNX2 |  |  |  |  |
| IDH1 |  |  |  |  |
| GPX1 |  |  |  |  |
| CIITA |  |  |  |  |
| DUOX2 |  |  |  |  |
| EPCAM |  |  |  |  |
| PRODH |  |  |  |  |
| SLC9A3 |  |  |  |  |
| S100B |  |  |  |  |
| IL26 |  |  |  |  |
| SH2B3 |  |  |  |  |
| MIR93 |  |  |  |  |
| ABCC6 |  |  |  |  |
| BCL10 |  |  |  |  |
| FLT1 |  |  |  |  |
| JUN |  |  |  |  |
| DEFA5 |  |  |  |  |
| MIR182 |  |  |  |  |
| KL |  |  |  |  |
| PALB2 |  |  |  |  |
| SELL |  |  |  |  |
| IFNB1 |  |  |  |  |
| CXCR3 |  |  |  |  |
| MIR342 |  |  |  |  |
| FOS |  |  |  |  |
| BMP6 |  |  |  |  |
| HMGCR |  |  |  |  |
| RELA |  |  |  |  |
| FLNC |  |  |  |  |
| HTR2A |  |  |  |  |
| CYCS |  |  |  |  |
| APOH |  |  |  |  |
| HSPA4 |  |  |  |  |
| MIR221 |  |  |  |  |
| MIR150 |  |  |  |  |
| MIR34C |  |  |  |  |
| FLT4 |  |  |  |  |
| GGT1 |  |  |  |  |
| SIAE |  |  |  |  |
| CTSB |  |  |  |  |
| IL3 |  |  |  |  |
| DAG1 |  |  |  |  |
| LCN2 |  |  |  |  |
| ALOX5 |  |  |  |  |
| ERCC1 |  |  |  |  |
| PPARA |  |  |  |  |
| PLG |  |  |  |  |
| MIR31 |  |  |  |  |
| ZNF687 |  |  |  |  |
| SETD2 |  |  |  |  |
| LBR |  |  |  |  |
| CCR9 |  |  |  |  |
| PSMB9 |  |  |  |  |
| CYP19A1 |  |  |  |  |
| DNMT3A |  |  |  |  |
| KCNN4 |  |  |  |  |
| MIR142 |  |  |  |  |
| PLAT |  |  |  |  |
| COL5A1 |  |  |  |  |
| BCL2 |  |  |  |  |
| MET |  |  |  |  |
| IL37 |  |  |  |  |
| GNB3 |  |  |  |  |
| ANG |  |  |  |  |
| CD27 |  |  |  |  |
| ETV6 |  |  |  |  |
| IMPG2 |  |  |  |  |
| SLC40A1 |  |  |  |  |
| PDGFB |  |  |  |  |
| CDKN2B |  |  |  |  |
| IDH2 |  |  |  |  |
| GLI3 |  |  |  |  |
| PLA2G7 |  |  |  |  |
| PEX5 |  |  |  |  |
| MAPK14 |  |  |  |  |
| RTEL1 |  |  |  |  |
| KDR |  |  |  |  |
| MBP |  |  |  |  |
| NBN |  |  |  |  |
| CASP9 |  |  |  |  |
| NOS1 |  |  |  |  |
| MIR30A |  |  |  |  |
| SAA1 |  |  |  |  |
| SYP |  |  |  |  |
| MYO5B |  |  |  |  |
| NEK9 |  |  |  |  |
| MVK |  |  |  |  |
| IKZF1 |  |  |  |  |
| PRKG1 |  |  |  |  |
| CCK |  |  |  |  |
| CSF1 |  |  |  |  |
| DKK1 |  |  |  |  |
| VIM |  |  |  |  |
| MTRR |  |  |  |  |
| PSMB8 |  |  |  |  |
| TYMP |  |  |  |  |
| CD80 |  |  |  |  |
| SYNE1 |  |  |  |  |
| SPINK1 |  |  |  |  |
| U2AF1 |  |  |  |  |
| SMAD2 |  |  |  |  |
| NR3C2 |  |  |  |  |
| MIR133B |  |  |  |  |
| MIR27A |  |  |  |  |
| PON3 |  |  |  |  |
| IGFBP3 |  |  |  |  |
| KRT8 |  |  |  |  |
| UBE4A |  |  |  |  |
| KRT18 |  |  |  |  |
| RB1 |  |  |  |  |
| MIR19A |  |  |  |  |
| ENO2 |  |  |  |  |
| BMP4 |  |  |  |  |
| MIR483 |  |  |  |  |
| NFE2L2 |  |  |  |  |
| CDKN2B-AS1 |  |  |  |  |
| DDC |  |  |  |  |
| CSF1R |  |  |  |  |
| TGM2 |  |  |  |  |
| CFB |  |  |  |  |
| PMPCA |  |  |  |  |
| FLCN |  |  |  |  |
| PRKCD |  |  |  |  |
| MIR127 |  |  |  |  |
| MDM2 |  |  |  |  |
| IL7 |  |  |  |  |
| FMNL2 |  |  |  |  |
| CDKN1A |  |  |  |  |
| CHGA |  |  |  |  |
| MIR200B |  |  |  |  |
| LEPR |  |  |  |  |
| PEX13 |  |  |  |  |
| MIR199A1 |  |  |  |  |
| SEMA3C |  |  |  |  |
| MIR10B |  |  |  |  |
| DPYD |  |  |  |  |
| NCSTN |  |  |  |  |
| SYNGAP1 |  |  |  |  |
| DNMT1 |  |  |  |  |
| HSP90AA1 |  |  |  |  |
| ATN1 |  |  |  |  |
| CYP1A1 |  |  |  |  |
| WNT1 |  |  |  |  |
| CHUK |  |  |  |  |
| SOX2 |  |  |  |  |
| MIR200A |  |  |  |  |
| KCNQ1OT1 |  |  |  |  |
| MIR320A |  |  |  |  |
| NAT2 |  |  |  |  |
| KNG1 |  |  |  |  |
| SLC19A1 |  |  |  |  |
| C5 |  |  |  |  |
| NEU1 |  |  |  |  |
| EYS |  |  |  |  |
| JAK1 |  |  |  |  |
| CPS1 |  |  |  |  |
| KITLG |  |  |  |  |
| MMP14 |  |  |  |  |
| CTDP1 |  |  |  |  |
| CFTR-AS1 |  |  |  |  |
| WAS |  |  |  |  |
| HGF |  |  |  |  |
| TNFSF13B |  |  |  |  |
| BRIP1 |  |  |  |  |
| PDGFRA |  |  |  |  |
| MIR222 |  |  |  |  |
| IFNA2 |  |  |  |  |
| SLC16A2 |  |  |  |  |
| DGKE |  |  |  |  |
| MIR22 |  |  |  |  |
| TREX1 |  |  |  |  |
| FOXP1 |  |  |  |  |
| MADCAM1 |  |  |  |  |
| HMGB1 |  |  |  |  |
| CTSK |  |  |  |  |
| PTH1R |  |  |  |  |
| CD86 |  |  |  |  |
| GSTT1 |  |  |  |  |
| MUC5AC |  |  |  |  |
| BLM |  |  |  |  |
| CYP1B1 |  |  |  |  |
| CYP3A4 |  |  |  |  |
| HJV |  |  |  |  |
| CREB1 |  |  |  |  |
| ANKH |  |  |  |  |
| CARD14 |  |  |  |  |
| SOCS1 |  |  |  |  |
| NDUFA13 |  |  |  |  |
| ESR2 |  |  |  |  |
| NQO1 |  |  |  |  |
| SPARC |  |  |  |  |
| HERC2 |  |  |  |  |
| AFP |  |  |  |  |
| CD34 |  |  |  |  |
| H19 |  |  |  |  |
| HLA-DPA1 |  |  |  |  |
| RNASE3 |  |  |  |  |
| NKX2-1 |  |  |  |  |
| CLEC7A |  |  |  |  |
| RPS27A |  |  |  |  |
| JAK3 |  |  |  |  |
| C4B |  |  |  |  |
| GHR |  |  |  |  |
| BCL2L1 |  |  |  |  |
| NPY |  |  |  |  |
| CHI3L1 |  |  |  |  |
| STK11 |  |  |  |  |
| SCNN1A |  |  |  |  |
| ISG15 |  |  |  |  |
| PIK3CD |  |  |  |  |
| MMP12 |  |  |  |  |
| RAC1 |  |  |  |  |
| FLI1 |  |  |  |  |
| TRPC6 |  |  |  |  |
| RBP3 |  |  |  |  |
| STAT6 |  |  |  |  |
| TAB2 |  |  |  |  |
| MIR214 |  |  |  |  |
| VEGFC |  |  |  |  |
| NCAM1 |  |  |  |  |
| ANXA11 |  |  |  |  |
| KAT6B |  |  |  |  |
| ANXA5 |  |  |  |  |
| DPP4 |  |  |  |  |
| ERBB3 |  |  |  |  |
| CD55 |  |  |  |  |
| DAO |  |  |  |  |
| GH1 |  |  |  |  |
| GSN |  |  |  |  |
| DSG2 |  |  |  |  |
| IL33 |  |  |  |  |
| CD274 |  |  |  |  |
| PHEX |  |  |  |  |
| IKBKB |  |  |  |  |
| MIR9-1 |  |  |  |  |
| SLC10A2 |  |  |  |  |
| IL19 |  |  |  |  |
| SLC6A8 |  |  |  |  |
| CDKN1B |  |  |  |  |
| PRSS1 |  |  |  |  |
| IGFBP1 |  |  |  |  |
| KRT1 |  |  |  |  |
| PTGS1 |  |  |  |  |
| CNTNAP2 |  |  |  |  |
| SLPI |  |  |  |  |
| RAD51 |  |  |  |  |
| AKT2 |  |  |  |  |
| AMACR |  |  |  |  |
| GZMB |  |  |  |  |
| FGG |  |  |  |  |
| ITGAL |  |  |  |  |
| TCF7L2 |  |  |  |  |
| CYP21A2 |  |  |  |  |
| MAPK3 |  |  |  |  |
| DSG1 |  |  |  |  |
| IGHM |  |  |  |  |
| TNFRSF8 |  |  |  |  |
| CDC42 |  |  |  |  |
| LGALS3 |  |  |  |  |
| PIK3C2A |  |  |  |  |
| VRK1 |  |  |  |  |
| CCR3 |  |  |  |  |
| CD46 |  |  |  |  |
| DNMT3B |  |  |  |  |
| AXIN2 |  |  |  |  |
| KRT7 |  |  |  |  |
| HLA-DRA |  |  |  |  |
| YY1AP1 |  |  |  |  |
| GALT |  |  |  |  |
| IRS2 |  |  |  |  |
| SFTPD |  |  |  |  |
| MIRLET7A1 |  |  |  |  |
| IL18R1 |  |  |  |  |
| TNFSF4 |  |  |  |  |
| IDO1 |  |  |  |  |
| DEFA6 |  |  |  |  |
| SI |  |  |  |  |
| MIR181A1 |  |  |  |  |
| IL16 |  |  |  |  |
| CCL20 |  |  |  |  |
| FGB |  |  |  |  |
| TGIF1 |  |  |  |  |
| CCR2 |  |  |  |  |
| CYP27B1 |  |  |  |  |
| WARS1 |  |  |  |  |
| HSPA5 |  |  |  |  |
| MRE11 |  |  |  |  |
| MICA |  |  |  |  |
| IRAK4 |  |  |  |  |
| ADAMTS4 |  |  |  |  |
| WWOX |  |  |  |  |
| SON |  |  |  |  |
| MIR200C |  |  |  |  |
| TAC1 |  |  |  |  |
| IRAK1 |  |  |  |  |
| USP8 |  |  |  |  |
| MAP3K7 |  |  |  |  |
| CRH |  |  |  |  |
| MYCN |  |  |  |  |
| VCL |  |  |  |  |
| MAGI2 |  |  |  |  |
| LZTR1 |  |  |  |  |
| MIR10A |  |  |  |  |
| IL11 |  |  |  |  |
| COMP |  |  |  |  |
| MAPK8 |  |  |  |  |
| CD163 |  |  |  |  |
| TIMP2 |  |  |  |  |
| IRF8 |  |  |  |  |
| CUX1 |  |  |  |  |
| ATP2A1 |  |  |  |  |
| CYP2E1 |  |  |  |  |
| DNASE1 |  |  |  |  |
| CYP2C9 |  |  |  |  |
| CDH3 |  |  |  |  |
| C1S |  |  |  |  |
| CCL4 |  |  |  |  |
| PDX1 |  |  |  |  |
| MOG |  |  |  |  |
| MIR25 |  |  |  |  |
| SHBG |  |  |  |  |
| CXCR1 |  |  |  |  |
| KIAA1109 |  |  |  |  |
| LIFR |  |  |  |  |
| HSPA8 |  |  |  |  |
| PIK3R2 |  |  |  |  |
| SLC12A4 |  |  |  |  |
| ANGPT2 |  |  |  |  |
| GREM1 |  |  |  |  |
| EIF2AK2 |  |  |  |  |
| EWSR1 |  |  |  |  |
| VIP |  |  |  |  |
| MIR34B |  |  |  |  |
| UBQLN4 |  |  |  |  |
| MIR148A |  |  |  |  |
| IL9 |  |  |  |  |
| ATF6 |  |  |  |  |
| TIMP3 |  |  |  |  |
| TBX21 |  |  |  |  |
| SLC34A3 |  |  |  |  |
| MIR18A |  |  |  |  |
| SIRT1 |  |  |  |  |
| TLR7 |  |  |  |  |
| LAMB1 |  |  |  |  |
| TPM3 |  |  |  |  |
| PARP1 |  |  |  |  |
| MIR486-1 |  |  |  |  |
| NOX1 |  |  |  |  |
| MYOC |  |  |  |  |
| IL1RAPL2 |  |  |  |  |
| WASHC5 |  |  |  |  |
| HSPA1A |  |  |  |  |
| NDUFAF3 |  |  |  |  |
| TYROBP |  |  |  |  |
| AQP4 |  |  |  |  |
| ARID1A |  |  |  |  |
| SHANK3 |  |  |  |  |
| XRCC1 |  |  |  |  |
| CRYAB |  |  |  |  |
| ECM1 |  |  |  |  |
| DDIT3 |  |  |  |  |
| EZH2 |  |  |  |  |
| KLK3 |  |  |  |  |
| C12orf60 |  |  |  |  |
| LAMB2 |  |  |  |  |
| ZNF365 |  |  |  |  |
| CNTF |  |  |  |  |
| LAMP1 |  |  |  |  |
| DOCK3 |  |  |  |  |
| IL6ST |  |  |  |  |
| CEACAM3 |  |  |  |  |
| ABCC2 |  |  |  |  |
| MAF |  |  |  |  |
| XK |  |  |  |  |
| ALDH2 |  |  |  |  |
| MMP7 |  |  |  |  |
| HSPA1L |  |  |  |  |
| YAP1 |  |  |  |  |
| DCC |  |  |  |  |
| ATP4A |  |  |  |  |
| CHD2 |  |  |  |  |
| CD44 |  |  |  |  |
| MIR204 |  |  |  |  |
| CTSG |  |  |  |  |
| NR1I2 |  |  |  |  |
| TAP1 |  |  |  |  |
| MIR23A |  |  |  |  |
| MIR196A1 |  |  |  |  |
| EPHX1 |  |  |  |  |
| NR1H3 |  |  |  |  |
| DEFB1 |  |  |  |  |
| ATP12A |  |  |  |  |
| MKI67 |  |  |  |  |
| KLRK1 |  |  |  |  |
| PF4 |  |  |  |  |
| ACE2 |  |  |  |  |
| AHSG |  |  |  |  |
| FCRL3 |  |  |  |  |
| CXCL9 |  |  |  |  |
| SMAD7 |  |  |  |  |
| IHH |  |  |  |  |
| C2 |  |  |  |  |
| DVL1 |  |  |  |  |
| SMARCB1 |  |  |  |  |
| ZEB1 |  |  |  |  |
| CXCR2 |  |  |  |  |
| HAVCR1 |  |  |  |  |
| ITPR3 |  |  |  |  |
| CD244 |  |  |  |  |
| HNRNPK |  |  |  |  |
| CDK4 |  |  |  |  |
| MIR24-1 |  |  |  |  |
| POU5F1 |  |  |  |  |
| RHOA |  |  |  |  |
| DCTN4 |  |  |  |  |
| PABPN1 |  |  |  |  |
| AHR |  |  |  |  |
| SCUBE3 |  |  |  |  |
| MIR205 |  |  |  |  |
| DCAF8 |  |  |  |  |
| BECN1 |  |  |  |  |
| SNAI2 |  |  |  |  |
| MAPK10 |  |  |  |  |
| CTCF |  |  |  |  |
| FAT4 |  |  |  |  |
| ITGA2 |  |  |  |  |
| GPR35 |  |  |  |  |
| COL6A3 |  |  |  |  |
| ITLN1 |  |  |  |  |
| CDH2 |  |  |  |  |
| ZFAT |  |  |  |  |
| MIR195 |  |  |  |  |
| FZD6 |  |  |  |  |
| TLR6 |  |  |  |  |
| MMEL1 |  |  |  |  |
| CCDC40 |  |  |  |  |
| KIR3DL1 |  |  |  |  |
| NTF3 |  |  |  |  |
| GAST |  |  |  |  |
| SLC2A9 |  |  |  |  |
| CCL17 |  |  |  |  |
| ACKR1 |  |  |  |  |
| RARB |  |  |  |  |
| UBAC2 |  |  |  |  |
| SUOX |  |  |  |  |
| TPM1 |  |  |  |  |
| KCNJ13 |  |  |  |  |
| EPX |  |  |  |  |
| SLC26A3 |  |  |  |  |
| GDF15 |  |  |  |  |
| TNFSF10 |  |  |  |  |
| RAB27A |  |  |  |  |
| CSNK2A1 |  |  |  |  |
| TAGAP |  |  |  |  |
| AMER1 |  |  |  |  |
| CLUAP1 |  |  |  |  |
| EYA1 |  |  |  |  |
| CXCL1 |  |  |  |  |
| CYP2C19 |  |  |  |  |
| P4HA2 |  |  |  |  |
| CNR1 |  |  |  |  |
| IRF4 |  |  |  |  |
| MEG3 |  |  |  |  |
| TNXB |  |  |  |  |
| STAT5A |  |  |  |  |
| KMT2C |  |  |  |  |
| XBP1 |  |  |  |  |
| RAC2 |  |  |  |  |
| ITPA |  |  |  |  |
| PLCG2 |  |  |  |  |
| IBD19 |  |  |  |  |
| CEACAM5 |  |  |  |  |
| TNPO3 |  |  |  |  |
| RIT1 |  |  |  |  |
| KDM4C |  |  |  |  |
| PAX5 |  |  |  |  |
| MIR100 |  |  |  |  |
| PGM1 |  |  |  |  |
| PTHLH |  |  |  |  |
| TRAF3IP2 |  |  |  |  |
| FOXO3 |  |  |  |  |
| THBS1 |  |  |  |  |
| TTC7A |  |  |  |  |
| LMNB2 |  |  |  |  |
| SDC1 |  |  |  |  |
| MUC2 |  |  |  |  |
| DICER1 |  |  |  |  |
| DCN |  |  |  |  |
| MIR146B |  |  |  |  |
| SOCS3 |  |  |  |  |
| IL17RA |  |  |  |  |
| CTNNA1 |  |  |  |  |
| C11orf65 |  |  |  |  |
| TNNI3K |  |  |  |  |
| ATRIP |  |  |  |  |
| MAP2K2 |  |  |  |  |
| CA2 |  |  |  |  |
| NMNAT1 |  |  |  |  |
| ZNF423 |  |  |  |  |
| QRICH1 |  |  |  |  |
| IGF2R |  |  |  |  |
| POLD1 |  |  |  |  |
| TAP2 |  |  |  |  |
| CTSF |  |  |  |  |
| ORMDL3 |  |  |  |  |
| KRT19 |  |  |  |  |
| AKT3 |  |  |  |  |
| MIR424 |  |  |  |  |
| TRAF6 |  |  |  |  |
| RORC |  |  |  |  |
| FGFR4 |  |  |  |  |
| PGF |  |  |  |  |
| CYP3A5 |  |  |  |  |
| BBIP1 |  |  |  |  |
| PDE4A |  |  |  |  |
| DDX58 |  |  |  |  |
| AUTS2 |  |  |  |  |
| P2RX7 |  |  |  |  |
| CFP |  |  |  |  |
| HLA-DRB5 |  |  |  |  |
| HPS4 |  |  |  |  |
| MICB |  |  |  |  |
| PRKD1 |  |  |  |  |
| STUB1 |  |  |  |  |
| DST |  |  |  |  |
| MIRLET7I |  |  |  |  |
| ETS1 |  |  |  |  |
| SIGLEC5 |  |  |  |  |
| MASP2 |  |  |  |  |
| CYLD |  |  |  |  |
| TWIST1 |  |  |  |  |
| MLH3 |  |  |  |  |
| NT5E |  |  |  |  |
| UBD |  |  |  |  |
| GFRA1 |  |  |  |  |
| COX5A |  |  |  |  |
| SHOC2 |  |  |  |  |
| PVALB |  |  |  |  |
| TSFM |  |  |  |  |
| C1R |  |  |  |  |
| WNT2B |  |  |  |  |
| AP3B1 |  |  |  |  |
| ENO1 |  |  |  |  |
| TPH1 |  |  |  |  |
| NFKBIL1 |  |  |  |  |
| FYN |  |  |  |  |
| MIR23B |  |  |  |  |
| CALB2 |  |  |  |  |
| HSPA1B |  |  |  |  |
| TCOF1 |  |  |  |  |
| ALPP |  |  |  |  |
| EIF2B4 |  |  |  |  |
| EFEMP1 |  |  |  |  |
| KLHDC8B |  |  |  |  |
| CD81 |  |  |  |  |
| MIR15B |  |  |  |  |
| VKORC1 |  |  |  |  |
| PLAUR |  |  |  |  |
| FADD |  |  |  |  |
| HNMT |  |  |  |  |
| TSLP |  |  |  |  |
| FTO |  |  |  |  |
| CEP63 |  |  |  |  |
| TRB |  |  |  |  |
| TJP1 |  |  |  |  |
| MIR26A1 |  |  |  |  |
| POLE |  |  |  |  |
| UGT1A6 |  |  |  |  |
| MSX1 |  |  |  |  |
| LCT |  |  |  |  |
| SLCO1B1 |  |  |  |  |
| MIR149 |  |  |  |  |
| AMT |  |  |  |  |
| RBFOX1 |  |  |  |  |
| AIF1 |  |  |  |  |
| NAMPT |  |  |  |  |
| PTX3 |  |  |  |  |
| SP1 |  |  |  |  |
| CDKAL1 |  |  |  |  |
| HPS1 |  |  |  |  |
| SCT |  |  |  |  |
| HINT1 |  |  |  |  |
| CCR7 |  |  |  |  |
| FANCC |  |  |  |  |
| ENTPD1 |  |  |  |  |
| BPI |  |  |  |  |
| LAMC2 |  |  |  |  |
| MIR451A |  |  |  |  |
| NOTCH4 |  |  |  |  |
| TNFRSF4 |  |  |  |  |
| CAMTA1 |  |  |  |  |
| APAF1 |  |  |  |  |
| MIR335 |  |  |  |  |
| RNASEH2C |  |  |  |  |
| ICOS |  |  |  |  |
| CYP24A1 |  |  |  |  |
| IL27 |  |  |  |  |
| FHIT |  |  |  |  |
| IGH |  |  |  |  |
| NLRP12 |  |  |  |  |
| WRN |  |  |  |  |
| RTEL1-TNFRSF6B |  |  |  |  |
| ADCY10 |  |  |  |  |
| ARMS2 |  |  |  |  |
| UGT1A8 |  |  |  |  |
| CYFIP2 |  |  |  |  |
| HPS3 |  |  |  |  |
| REL |  |  |  |  |
| FURIN |  |  |  |  |
| HDAC2 |  |  |  |  |
| LAMA3 |  |  |  |  |
| PLA2G4A |  |  |  |  |
| GYPA |  |  |  |  |
| ALDH5A1 |  |  |  |  |
| SEPTIN9 |  |  |  |  |
| SUCLA2 |  |  |  |  |
| OGG1 |  |  |  |  |
| MIRLET7G |  |  |  |  |
| RARRES2 |  |  |  |  |
| VPS11 |  |  |  |  |
| MIR16-1 |  |  |  |  |
| CACNA1G |  |  |  |  |
| FGF7 |  |  |  |  |
| MIR124-1 |  |  |  |  |
| CDK6 |  |  |  |  |
| TRPM3 |  |  |  |  |
| TIRAP |  |  |  |  |
| MUC16 |  |  |  |  |
| CD69 |  |  |  |  |
| HDAC9 |  |  |  |  |
| EPHX2 |  |  |  |  |
| GLS |  |  |  |  |
| ATP1A2 |  |  |  |  |
| FOXO1 |  |  |  |  |
| SEPSECS |  |  |  |  |
| GCKR |  |  |  |  |
| GRP |  |  |  |  |
| FOLH1 |  |  |  |  |
| OCLN |  |  |  |  |
| BUB1 |  |  |  |  |
| ACADM |  |  |  |  |
| CCL7 |  |  |  |  |
| PTPRJ |  |  |  |  |
| IBSP |  |  |  |  |
| SLC7A7 |  |  |  |  |
| DSCAM |  |  |  |  |
| GAL |  |  |  |  |
| AXIN1 |  |  |  |  |
| FLG-AS1 |  |  |  |  |
| MIR423 |  |  |  |  |
| TNNI2 |  |  |  |  |
| SOD3 |  |  |  |  |
| TNIP1 |  |  |  |  |
| FBXW7 |  |  |  |  |
| CAST |  |  |  |  |
| MIRLET7B |  |  |  |  |
| AURKA |  |  |  |  |
| TNFRSF10A |  |  |  |  |
| ARHGEF2 |  |  |  |  |
| MALAT1 |  |  |  |  |
| WNT10A |  |  |  |  |
| TREM1 |  |  |  |  |
| PVT1 |  |  |  |  |
| CCR4 |  |  |  |  |
| TLR8 |  |  |  |  |
| DHFR |  |  |  |  |
| CAVIN1 |  |  |  |  |
| PPP1CB |  |  |  |  |
| CHRNA7 |  |  |  |  |
| PROC |  |  |  |  |
| NLRP7 |  |  |  |  |
| SLC9A3R1 |  |  |  |  |
| GC |  |  |  |  |
| MIR139 |  |  |  |  |
| MGAM |  |  |  |  |
| TUBB2B |  |  |  |  |
| SEMA4A |  |  |  |  |
| UGT1A7 |  |  |  |  |
| TDGF1 |  |  |  |  |
| CXCL2 |  |  |  |  |
| ABCG2 |  |  |  |  |
| CD59 |  |  |  |  |
| LAT |  |  |  |  |
| GLI1 |  |  |  |  |
| UNC13D |  |  |  |  |
| HDAC4 |  |  |  |  |
| MIR125B1 |  |  |  |  |
| CLEC16A |  |  |  |  |
| MIR130A |  |  |  |  |
| NR1H2 |  |  |  |  |
| MIR99A |  |  |  |  |
| MIR373 |  |  |  |  |
| MIR92A1 |  |  |  |  |
| WNT4 |  |  |  |  |
| XRCC2 |  |  |  |  |
| NCR1 |  |  |  |  |
| BUB1B |  |  |  |  |
| WHRN |  |  |  |  |
| BIRC5 |  |  |  |  |
| ATG5 |  |  |  |  |
| MIR193A |  |  |  |  |
| FGF10 |  |  |  |  |
| CAPN10 |  |  |  |  |
| ALDOB |  |  |  |  |
| KMT5B |  |  |  |  |
| ZMIZ1 |  |  |  |  |
| ITGB4 |  |  |  |  |
| PYY |  |  |  |  |
| SERPING1 |  |  |  |  |
| PLCH2 |  |  |  |  |
| CYP7A1 |  |  |  |  |
| CLDN2 |  |  |  |  |
| IGHE |  |  |  |  |
| BMP7 |  |  |  |  |
| HTR1A |  |  |  |  |
| ATF4 |  |  |  |  |
| ANTXR2 |  |  |  |  |
| PPBP |  |  |  |  |
| PHB |  |  |  |  |
| LCK |  |  |  |  |
| CD68 |  |  |  |  |
| ODC1 |  |  |  |  |
| CAMP |  |  |  |  |
| INS-IGF2 |  |  |  |  |
| TNFSF12 |  |  |  |  |
| TCERG1 |  |  |  |  |
| CEBPA |  |  |  |  |
| PTPN12 |  |  |  |  |
| PPOX |  |  |  |  |
| MIR185 |  |  |  |  |
| MLN |  |  |  |  |
| KRT20 |  |  |  |  |
| HRH2 |  |  |  |  |
| CCL26 |  |  |  |  |
| TRIM21 |  |  |  |  |
| COX15 |  |  |  |  |
| IL6-AS1 |  |  |  |  |
| CHRNB2 |  |  |  |  |
| NTS |  |  |  |  |
| TMPRSS6 |  |  |  |  |
| CD38 |  |  |  |  |
| MIR338 |  |  |  |  |
| DMBT1 |  |  |  |  |
| KDM6A |  |  |  |  |
| FCGR1A |  |  |  |  |
| CARD11 |  |  |  |  |
| SCARB1 |  |  |  |  |
| PER3 |  |  |  |  |
| SCN2B |  |  |  |  |
| PPP2R1A |  |  |  |  |
| GABRB3 |  |  |  |  |
| TRPA1 |  |  |  |  |
| FDPS |  |  |  |  |
| DYNC2I1 |  |  |  |  |
| HAVCR2 |  |  |  |  |
| PRKCA |  |  |  |  |
| RASGRP1 |  |  |  |  |
| ANXA1 |  |  |  |  |
| JAZF1 |  |  |  |  |
| MIRLET7E |  |  |  |  |
| DOCK7 |  |  |  |  |
| SLCO2A1 |  |  |  |  |
| MTHFD1 |  |  |  |  |
| F2R |  |  |  |  |
| ZNF627 |  |  |  |  |
| CALM3 |  |  |  |  |
| ETFDH |  |  |  |  |
| TGFA |  |  |  |  |
| HLA-DQA2 |  |  |  |  |
| NLRC4 |  |  |  |  |
| KIF3A |  |  |  |  |
| NEAT1 |  |  |  |  |
| PSMB10 |  |  |  |  |
| BAD |  |  |  |  |
| ICAM3 |  |  |  |  |
| HOTAIR |  |  |  |  |
| PCNA |  |  |  |  |
| WNT5A |  |  |  |  |
| FOXP2 |  |  |  |  |
| RBPJ |  |  |  |  |
| PMS1 |  |  |  |  |
| PRKCB |  |  |  |  |
| TCN2 |  |  |  |  |
| BANK1 |  |  |  |  |
| ITGB1 |  |  |  |  |
| EZR |  |  |  |  |
| MANBA |  |  |  |  |
| MAP3K1 |  |  |  |  |
| SLIT2 |  |  |  |  |
| IFNAR2 |  |  |  |  |
| EDA |  |  |  |  |
| EIF2S1 |  |  |  |  |
| PUS10 |  |  |  |  |
| SKIV2L |  |  |  |  |
| ERN1 |  |  |  |  |
| LIF |  |  |  |  |
| BAK1 |  |  |  |  |
| TNNT3 |  |  |  |  |
| MIR324 |  |  |  |  |
| PTGIS |  |  |  |  |
| MIP |  |  |  |  |
| MGMT |  |  |  |  |
| DSPP |  |  |  |  |
| ANTXR1 |  |  |  |  |
| MIR193B |  |  |  |  |
| FERMT1 |  |  |  |  |
| MIR28 |  |  |  |  |
| CSNK2B |  |  |  |  |
| MIR183 |  |  |  |  |
| GHSR |  |  |  |  |
| IL1RL1 |  |  |  |  |
| CASP7 |  |  |  |  |
| PADI4 |  |  |  |  |
| TRPV1 |  |  |  |  |
| CX3CL1 |  |  |  |  |
| TACR1 |  |  |  |  |
| HPS5 |  |  |  |  |
| NDUFAF1 |  |  |  |  |
| HAP1 |  |  |  |  |
| NRP1 |  |  |  |  |
| TXN |  |  |  |  |
| FABP2 |  |  |  |  |
| MIR181A2 |  |  |  |  |
| MIR106A |  |  |  |  |
| MIR96 |  |  |  |  |
| CDX2 |  |  |  |  |
| NPSR1 |  |  |  |  |
| WNT3 |  |  |  |  |
| SOX11 |  |  |  |  |
| PPM1D |  |  |  |  |
| KAT5 |  |  |  |  |
| EMC1 |  |  |  |  |
| FCN3 |  |  |  |  |
| NR0B2 |  |  |  |  |
| KEAP1 |  |  |  |  |
| ERG |  |  |  |  |
| AKAP9 |  |  |  |  |
| SLAMF1 |  |  |  |  |
| APOA4 |  |  |  |  |
| SPINK5 |  |  |  |  |
| MUC4 |  |  |  |  |
| CXCL5 |  |  |  |  |
| TFF3 |  |  |  |  |
| UCP2 |  |  |  |  |
| CHRM3 |  |  |  |  |
| PRPF6 |  |  |  |  |
| MIR224 |  |  |  |  |
| GPX3 |  |  |  |  |
| IGFBP2 |  |  |  |  |
| HSD11B2 |  |  |  |  |
| MYRF |  |  |  |  |
| KRT17 |  |  |  |  |
| ARHGDIA |  |  |  |  |
| IRF6 |  |  |  |  |
| KLF4 |  |  |  |  |
| SLC9A9 |  |  |  |  |
| CDK1 |  |  |  |  |
| RPS6KB1 |  |  |  |  |
| MMP19 |  |  |  |  |
| MIR151A |  |  |  |  |
| AMBP |  |  |  |  |
| DPEP1 |  |  |  |  |
| IFNAR1 |  |  |  |  |
| TUBB2A |  |  |  |  |
| FERMT3 |  |  |  |  |
| EFEMP2 |  |  |  |  |
| ADH1B |  |  |  |  |
| FLNC-AS1 |  |  |  |  |
| MIR212 |  |  |  |  |
| MIR26B |  |  |  |  |
| MIR331 |  |  |  |  |
| MIR215 |  |  |  |  |
| TNFRSF25 |  |  |  |  |
| TNC |  |  |  |  |
| CDKN3 |  |  |  |  |
| CACNA2D1 |  |  |  |  |
| TSPO |  |  |  |  |
| F13B |  |  |  |  |
| LGALS1 |  |  |  |  |
| CCND2 |  |  |  |  |
| MIR455 |  |  |  |  |
| CYP1A2 |  |  |  |  |
| RASA2 |  |  |  |  |
| CD5 |  |  |  |  |
| BSN |  |  |  |  |
| MLX |  |  |  |  |
| WNT3A |  |  |  |  |
| F2RL1 |  |  |  |  |
| PKLR |  |  |  |  |
| VTN |  |  |  |  |
| SLC11A2 |  |  |  |  |
| ACSL4 |  |  |  |  |
| SLC25A20 |  |  |  |  |
| PI3 |  |  |  |  |
| LYRM4 |  |  |  |  |
| EGR1 |  |  |  |  |
| SLC39A8 |  |  |  |  |
| HDAC1 |  |  |  |  |
| MCL1 |  |  |  |  |
| UTS2 |  |  |  |  |
| PROCR |  |  |  |  |
| B3GALT6 |  |  |  |  |
| MX1 |  |  |  |  |
| ACTN1 |  |  |  |  |
| BDKRB2 |  |  |  |  |
| TNFRSF6B |  |  |  |  |
| NR2F2 |  |  |  |  |
| HPGD |  |  |  |  |
| OPRM1 |  |  |  |  |
| APEX1 |  |  |  |  |
| ZNF148 |  |  |  |  |
| ANGPT1 |  |  |  |  |
| XRCC3 |  |  |  |  |
| SLC25A15 |  |  |  |  |
| CCL21 |  |  |  |  |
| MIR196A2 |  |  |  |  |
| SLC9A1 |  |  |  |  |
| PDGFRL |  |  |  |  |
| MIR590 |  |  |  |  |
| MYB |  |  |  |  |
| MAFB |  |  |  |  |
| TKT |  |  |  |  |
| PIK3CG |  |  |  |  |
| MIR191 |  |  |  |  |
| CD247 |  |  |  |  |
| FAH |  |  |  |  |
| GPBAR1 |  |  |  |  |
| AKR1B1 |  |  |  |  |
| TP73 |  |  |  |  |
| AP4B1-AS1 |  |  |  |  |
| DPP6 |  |  |  |  |
| KCNK4 |  |  |  |  |
| ACKR2 |  |  |  |  |
| OSM |  |  |  |  |
| RPS26 |  |  |  |  |
| TALDO1 |  |  |  |  |
| RFX5 |  |  |  |  |
| REG4 |  |  |  |  |
| SETDB1 |  |  |  |  |
| LPP |  |  |  |  |
| NFATC1 |  |  |  |  |
| DACT1 |  |  |  |  |
| NUDT15 |  |  |  |  |
| SOX5 |  |  |  |  |
| DGAT1 |  |  |  |  |
| FBXO38 |  |  |  |  |
| MAPK9 |  |  |  |  |
| FEN1 |  |  |  |  |
| ULK1 |  |  |  |  |
| DAPK1 |  |  |  |  |
| TLR10 |  |  |  |  |
| MIR574 |  |  |  |  |
| GPX4 |  |  |  |  |
| FZD3 |  |  |  |  |
| CXCL11 |  |  |  |  |
| RARA |  |  |  |  |
| WNT10B |  |  |  |  |
| RNASET2 |  |  |  |  |
| MSH3 |  |  |  |  |
| RAD50 |  |  |  |  |
| IGSF6 |  |  |  |  |
| WNT7A |  |  |  |  |
| PLTP |  |  |  |  |
| PTPA |  |  |  |  |
| ABO |  |  |  |  |
| HLA-E |  |  |  |  |
| PPIF |  |  |  |  |
| LHX3 |  |  |  |  |
| KRTCAP3 |  |  |  |  |
| ACO2 |  |  |  |  |
| LBP |  |  |  |  |
| QDPR |  |  |  |  |
| SEC24C |  |  |  |  |
| PSMA6 |  |  |  |  |
| CYP2A6 |  |  |  |  |
| NTF4 |  |  |  |  |
| SPRY4 |  |  |  |  |
| PIK3R4 |  |  |  |  |
| HHIP |  |  |  |  |
| HLA-DMA |  |  |  |  |
| PIGR |  |  |  |  |
| RPL7 |  |  |  |  |
| OXA1L |  |  |  |  |
| TTC37 |  |  |  |  |
| LYZ |  |  |  |  |
| CYP11B1 |  |  |  |  |
| MUC3A |  |  |  |  |
| INPP5B |  |  |  |  |
| TNFRSF10B |  |  |  |  |
| CD27-AS1 |  |  |  |  |
| PTPN1 |  |  |  |  |
| CSF2RA |  |  |  |  |
| MIR32 |  |  |  |  |
| UQCRC1 |  |  |  |  |
| COG6 |  |  |  |  |
| SLC22A2 |  |  |  |  |
| KLF6 |  |  |  |  |
| MIR135A1 |  |  |  |  |
| G6PC3 |  |  |  |  |
| CDK2 |  |  |  |  |
| DBN1 |  |  |  |  |
| CXCR5 |  |  |  |  |
| DLC1 |  |  |  |  |
| ELAVL1 |  |  |  |  |
| ERAP2 |  |  |  |  |
| ITGAE |  |  |  |  |
| SNAI1 |  |  |  |  |
| ACD |  |  |  |  |
| ACADS |  |  |  |  |
| DAB2 |  |  |  |  |
| ZNF142 |  |  |  |  |
| FSCN1 |  |  |  |  |
| EPHB4 |  |  |  |  |
| CAMK2G |  |  |  |  |
| CD24 |  |  |  |  |
| CFLAR |  |  |  |  |
| TOP1 |  |  |  |  |
| IL21R |  |  |  |  |
| MIR675 |  |  |  |  |
| ABCG1 |  |  |  |  |
| EPHB2 |  |  |  |  |
| TRAF1 |  |  |  |  |
| SLC2A4RG |  |  |  |  |
| AOC1 |  |  |  |  |
| CCL22 |  |  |  |  |
| EBI3 |  |  |  |  |
| AREG |  |  |  |  |
| FOXF1 |  |  |  |  |
| PNKD |  |  |  |  |
| SLC7A9 |  |  |  |  |
| NEUROG3 |  |  |  |  |
| LGALS9 |  |  |  |  |
| FKBP5 |  |  |  |  |
| ABCC4 |  |  |  |  |
| IKZF3 |  |  |  |  |
| BANF1 |  |  |  |  |
| TRAP1 |  |  |  |  |
| MIR103A1 |  |  |  |  |
| SLC22A23 |  |  |  |  |
| SYT11 |  |  |  |  |
| UGT1A9 |  |  |  |  |
| MLANA |  |  |  |  |
| BSG |  |  |  |  |
| APLN |  |  |  |  |
| GSDMB |  |  |  |  |
| PPIG |  |  |  |  |
| CHRNA2 |  |  |  |  |
| CACNA1E |  |  |  |  |
| FREM1 |  |  |  |  |
| PSORS1C1 |  |  |  |  |
| SOX2-OT |  |  |  |  |
| EHBP1 |  |  |  |  |
| SERPINA6 |  |  |  |  |
| ALOX12 |  |  |  |  |
| CLDN4 |  |  |  |  |
| HSD11B1 |  |  |  |  |
| SLC29A1 |  |  |  |  |
| LGALS4 |  |  |  |  |
| SH2B1 |  |  |  |  |
| SLC35D1 |  |  |  |  |
| OLIG2 |  |  |  |  |
| MIR27B |  |  |  |  |
| ANXA2 |  |  |  |  |
| RUBCN |  |  |  |  |
| ACO1 |  |  |  |  |
| HIBCH |  |  |  |  |
| THY1 |  |  |  |  |
| RSPH3 |  |  |  |  |
| MIR375 |  |  |  |  |
| EPAS1 |  |  |  |  |
| PITX1 |  |  |  |  |
| RUNX3 |  |  |  |  |
| FANCE |  |  |  |  |
| HDC |  |  |  |  |
| ITGAV |  |  |  |  |
| MEGF10 |  |  |  |  |
| HMGA2 |  |  |  |  |
| HHEX |  |  |  |  |
| SHC1 |  |  |  |  |
| TMEFF2 |  |  |  |  |
| PNP |  |  |  |  |
| MARS2 |  |  |  |  |
| DDB2 |  |  |  |  |
| CTNND1 |  |  |  |  |
| RPSA |  |  |  |  |
| AFF4 |  |  |  |  |
| SIX5 |  |  |  |  |
| IVD |  |  |  |  |
| FADS2 |  |  |  |  |
| GAS5 |  |  |  |  |
| TUG1 |  |  |  |  |
| AP4B1 |  |  |  |  |
| MST1R |  |  |  |  |
| CSF2RB |  |  |  |  |
| KIR2DL1 |  |  |  |  |
| ICAM2 |  |  |  |  |
| PPM1L |  |  |  |  |
| PRLR |  |  |  |  |
| FABP4 |  |  |  |  |
| LEPQTL1 |  |  |  |  |
| MUC6 |  |  |  |  |
| LACTB |  |  |  |  |
| MAPKBP1 |  |  |  |  |
| KIF21B |  |  |  |  |
| PSMD4 |  |  |  |  |
| WNT2 |  |  |  |  |
| SPRED1 |  |  |  |  |
| EFNB1 |  |  |  |  |
| SAA2 |  |  |  |  |
| MMP10 |  |  |  |  |
| TYMS |  |  |  |  |
| REG3A |  |  |  |  |
| RXRA |  |  |  |  |
| CCL13 |  |  |  |  |
| ZNF335 |  |  |  |  |
| SAA4 |  |  |  |  |
| ZNF513 |  |  |  |  |
| DLG1 |  |  |  |  |
| SKI |  |  |  |  |
| SERPINB1 |  |  |  |  |
| C10orf55 |  |  |  |  |
| XRCC6 |  |  |  |  |
| CEP250 |  |  |  |  |
| SMIM35 |  |  |  |  |
| PKM |  |  |  |  |
| ACP1 |  |  |  |  |
| ANGPTL3 |  |  |  |  |
| TNFRSF9 |  |  |  |  |
| S100A4 |  |  |  |  |
| HRH1 |  |  |  |  |
| CARD6 |  |  |  |  |
| TBXT |  |  |  |  |
| UGT1A10 |  |  |  |  |
| GRK2 |  |  |  |  |
| IKBKE |  |  |  |  |
| ITIH4 |  |  |  |  |
| RAD54B |  |  |  |  |
| SLC12A5 |  |  |  |  |
| PRG2 |  |  |  |  |
| PDXK |  |  |  |  |
| BCAR1 |  |  |  |  |
| PDGFA |  |  |  |  |
| RORA |  |  |  |  |
| MIR133A1 |  |  |  |  |
| PDSS2 |  |  |  |  |
| ADORA1 |  |  |  |  |
| RARS1 |  |  |  |  |
| APEH |  |  |  |  |
| EIF4E |  |  |  |  |
| USF1 |  |  |  |  |
| MSTO1 |  |  |  |  |
| FANCG |  |  |  |  |
| DEFB103B |  |  |  |  |
| TNFRSF18 |  |  |  |  |
| PRDM1 |  |  |  |  |
| MSN |  |  |  |  |
| DMP1 |  |  |  |  |
| AMPD2 |  |  |  |  |
| RAP1A |  |  |  |  |
| ETHE1 |  |  |  |  |
| CTBP1 |  |  |  |  |
| SCYL1 |  |  |  |  |
| AMBRA1 |  |  |  |  |
| PRKAA1 |  |  |  |  |
| MCC |  |  |  |  |
| ATG14 |  |  |  |  |
| PHGDH |  |  |  |  |
| PDPN |  |  |  |  |
| MIR19B1 |  |  |  |  |
| UGT1A4 |  |  |  |  |
| SMNDC1 |  |  |  |  |
| KDM1A |  |  |  |  |
| MIR532 |  |  |  |  |
| GP2 |  |  |  |  |
| BIRC3 |  |  |  |  |
| PTK2 |  |  |  |  |
| FADS1 |  |  |  |  |
| MYO1B |  |  |  |  |
| MIR130B |  |  |  |  |
| CDH13 |  |  |  |  |
| HSP90B1 |  |  |  |  |
| GPX2 |  |  |  |  |
| UGT1A |  |  |  |  |
| TFF1 |  |  |  |  |
| CRHR1 |  |  |  |  |
| GRB2 |  |  |  |  |
| ATXN2L |  |  |  |  |
| ADAM9 |  |  |  |  |
| CH25H |  |  |  |  |
| WNT9B |  |  |  |  |
| CCNY |  |  |  |  |
| NRG3 |  |  |  |  |
| CASP2 |  |  |  |  |
| FASN |  |  |  |  |
| SPEF2 |  |  |  |  |
| MIR361 |  |  |  |  |
| ACACA |  |  |  |  |
| SLC39A4 |  |  |  |  |
| C7 |  |  |  |  |
| NELL1 |  |  |  |  |
| HTR3A |  |  |  |  |
| PNMT |  |  |  |  |
| IL31 |  |  |  |  |
| MTHFD1L |  |  |  |  |
| XPC |  |  |  |  |
| SETD1A |  |  |  |  |
| AICDA |  |  |  |  |
| PRDX5 |  |  |  |  |
| FIS1 |  |  |  |  |
| MAVS |  |  |  |  |
| NME1 |  |  |  |  |
| MIR196B |  |  |  |  |
| MIR206 |  |  |  |  |
| NANOG |  |  |  |  |
| CUX2 |  |  |  |  |
| SPINT2 |  |  |  |  |
| TNFSF8 |  |  |  |  |
| ARHGAP45 |  |  |  |  |
| FAF1 |  |  |  |  |
| CLDN1 |  |  |  |  |
| CFL1 |  |  |  |  |
| CORO1A |  |  |  |  |
| HPSE |  |  |  |  |
| PARD3 |  |  |  |  |
| C5AR1 |  |  |  |  |
| CD2 |  |  |  |  |
| UGT1A3 |  |  |  |  |
| MIR30C1 |  |  |  |  |
| GPC5 |  |  |  |  |
| E2F1 |  |  |  |  |
| VAMP1 |  |  |  |  |
| CCR8 |  |  |  |  |
| NRIP1 |  |  |  |  |
| WNT7B |  |  |  |  |
| ANKRD55 |  |  |  |  |
| ABCC1 |  |  |  |  |
| UMPS |  |  |  |  |
| UCN |  |  |  |  |
| ATG7 |  |  |  |  |
| PPARD |  |  |  |  |
| YDJC |  |  |  |  |
| CMA1 |  |  |  |  |
| BDKRB1 |  |  |  |  |
| CD58 |  |  |  |  |
| AVIL |  |  |  |  |
| LOC110806262 |  |  |  |  |
| PMEL |  |  |  |  |
| OR5V1 |  |  |  |  |
| EMSY |  |  |  |  |
| NPEPPS |  |  |  |  |
| COL13A1 |  |  |  |  |
| UBE2D1 |  |  |  |  |
| SATB1 |  |  |  |  |
| BACE1-AS |  |  |  |  |
| B3GNT2 |  |  |  |  |
| FGF19 |  |  |  |  |
| TWIST2 |  |  |  |  |
| KANK2 |  |  |  |  |
| CTTN |  |  |  |  |
| MIR296 |  |  |  |  |
| TRIM28 |  |  |  |  |
| SOX4 |  |  |  |  |
| KLC2 |  |  |  |  |
| DPF2 |  |  |  |  |
| AMPD3 |  |  |  |  |
| BLZF1 |  |  |  |  |
| TRAF3 |  |  |  |  |
| UGT1A5 |  |  |  |  |
| CFDP1 |  |  |  |  |
| ALDH1A1 |  |  |  |  |
| GSAP |  |  |  |  |
| TOP2A |  |  |  |  |
| TRIB1 |  |  |  |  |
| TRAF2 |  |  |  |  |
| EPHA3 |  |  |  |  |
| STK39 |  |  |  |  |
| GHRH |  |  |  |  |
| CD1D |  |  |  |  |
| FMN2 |  |  |  |  |
| WNT8B |  |  |  |  |
| MUC19 |  |  |  |  |
| CRYGC |  |  |  |  |
| CCKBR |  |  |  |  |
| XPO1 |  |  |  |  |
| SNX27 |  |  |  |  |
| CDH11 |  |  |  |  |
| ADAM33 |  |  |  |  |
| CSNK1A1 |  |  |  |  |
| MTMR3 |  |  |  |  |
| CUL2 |  |  |  |  |
| FBLN1 |  |  |  |  |
| PIK3CB |  |  |  |  |
| MASP1 |  |  |  |  |
| SLC5A1 |  |  |  |  |
| ADAD1 |  |  |  |  |
| PLD1 |  |  |  |  |
| SCARNA5 |  |  |  |  |
| OPCML |  |  |  |  |
| BCL2L11 |  |  |  |  |
| CCNA2 |  |  |  |  |
| KIR2DL3 |  |  |  |  |
| CXCL6 |  |  |  |  |
| SUMO4 |  |  |  |  |
| APOBEC3G |  |  |  |  |
| IRAK3 |  |  |  |  |
| SYNPO |  |  |  |  |
| PTK2B |  |  |  |  |
| RECK |  |  |  |  |
| HLA-DOA |  |  |  |  |
| POLR1D |  |  |  |  |
| ITGB7 |  |  |  |  |
| RBM10 |  |  |  |  |
| TAPBP |  |  |  |  |
| CNTN5 |  |  |  |  |
| ALPI |  |  |  |  |
| GOT1 |  |  |  |  |
| H2AX |  |  |  |  |
| UBASH3A |  |  |  |  |
| REEP6 |  |  |  |  |
| CCNB1 |  |  |  |  |
| LSM2 |  |  |  |  |
| ROBO2 |  |  |  |  |
| XRCC5 |  |  |  |  |
| ASH1L |  |  |  |  |
| IL31RA |  |  |  |  |
| FDX1 |  |  |  |  |
| MAP3K8 |  |  |  |  |
| MPDZ |  |  |  |  |
| PLVAP |  |  |  |  |
| MEPE |  |  |  |  |
| NTN1 |  |  |  |  |
| LAD1 |  |  |  |  |
| SULT1A1 |  |  |  |  |
| KRT15 |  |  |  |  |
| OSMR |  |  |  |  |
| CADM1 |  |  |  |  |
| CHD4 |  |  |  |  |
| DDX39B |  |  |  |  |
| CUL1 |  |  |  |  |
| RNASE2 |  |  |  |  |
| PRKACB |  |  |  |  |
| TERF1 |  |  |  |  |
| SLCO1B3 |  |  |  |  |
| CD226 |  |  |  |  |
| PLA2G10 |  |  |  |  |
| CAV2 |  |  |  |  |
| MUC12 |  |  |  |  |
| IL24 |  |  |  |  |
| BLVRB |  |  |  |  |
| NUDT1 |  |  |  |  |
| TUFM |  |  |  |  |
| PTPN3 |  |  |  |  |
| DMAP1 |  |  |  |  |
| GPI |  |  |  |  |
| IGHG3 |  |  |  |  |
| GABBR1 |  |  |  |  |
| LTB |  |  |  |  |
| SLAMF7 |  |  |  |  |
| ADGRE3 |  |  |  |  |
| AQP3 |  |  |  |  |
| CLEC12A |  |  |  |  |
| MDC1 |  |  |  |  |
| MIR598 |  |  |  |  |
| SRP54 |  |  |  |  |
| RBX1 |  |  |  |  |
| ZGPAT |  |  |  |  |
| NR5A2 |  |  |  |  |
| CLMP |  |  |  |  |
| TOLLIP |  |  |  |  |
| SGK1 |  |  |  |  |
| MXI1 |  |  |  |  |
| CLDN11 |  |  |  |  |
| TRIM8 |  |  |  |  |
| LMAN1 |  |  |  |  |
| METTL9 |  |  |  |  |
| TRAF3IP2-AS1 |  |  |  |  |
| IGFBP5 |  |  |  |  |
| CCND3 |  |  |  |  |
| ROS1 |  |  |  |  |
| POU2F1 |  |  |  |  |
| ACYP1 |  |  |  |  |
| HVCN1 |  |  |  |  |
| IGHG1 |  |  |  |  |
| TCF3 |  |  |  |  |
| MID1 |  |  |  |  |
| UBE2N |  |  |  |  |
| TRPV6 |  |  |  |  |
| IFI27 |  |  |  |  |
| SCD |  |  |  |  |
| PTGDR2 |  |  |  |  |
| FOSL1 |  |  |  |  |
| PEBP1 |  |  |  |  |
| SLC36A2 |  |  |  |  |
| MIR137 |  |  |  |  |
| LEF1 |  |  |  |  |
| CCS |  |  |  |  |
| PSMD3 |  |  |  |  |
| EPHA2 |  |  |  |  |
| ST14 |  |  |  |  |
| TRAIP |  |  |  |  |
| IL3RA |  |  |  |  |
| NDST1 |  |  |  |  |
| PLA2G1B |  |  |  |  |
| MIR3936 |  |  |  |  |
| HLA-DOB |  |  |  |  |
| NRBF2 |  |  |  |  |
| PRDM5 |  |  |  |  |
| PLA2R1 |  |  |  |  |
| MEI1 |  |  |  |  |
| MAZ |  |  |  |  |
| NNMT |  |  |  |  |
| ABCC3 |  |  |  |  |
| CDK5RAP1 |  |  |  |  |
| HTR7 |  |  |  |  |
| LTA4H |  |  |  |  |
| RHEB |  |  |  |  |
| CD207 |  |  |  |  |
| FLOT1 |  |  |  |  |
| FDX2 |  |  |  |  |
| MIR371A |  |  |  |  |
| NAT1 |  |  |  |  |
| PIK3R5 |  |  |  |  |
| GPD2 |  |  |  |  |
| FLAD1 |  |  |  |  |
| RDH10 |  |  |  |  |
| EPC1 |  |  |  |  |
| DM1-AS |  |  |  |  |
| LPIN2 |  |  |  |  |
| TAGLN |  |  |  |  |
| PSMA4 |  |  |  |  |
| HTR4 |  |  |  |  |
| H6PD |  |  |  |  |
| ALOX15 |  |  |  |  |
| CCN1 |  |  |  |  |
| LEMD2 |  |  |  |  |
| PPID |  |  |  |  |
| THADA |  |  |  |  |
| USP40 |  |  |  |  |
| IRF1-AS1 |  |  |  |  |
| CHRNA5 |  |  |  |  |
| TM2D1 |  |  |  |  |
| PDLIM1 |  |  |  |  |
| SSTR2 |  |  |  |  |
| TMEM201 |  |  |  |  |
| CHKA |  |  |  |  |
| HDAC5 |  |  |  |  |
| FCAR |  |  |  |  |
| PYCARD |  |  |  |  |
| TST |  |  |  |  |
| SEPTIN2 |  |  |  |  |
| IL13RA2 |  |  |  |  |
| IQGAP1 |  |  |  |  |
| BMI1 |  |  |  |  |
| MUS81 |  |  |  |  |
| LTBR |  |  |  |  |
| CSK |  |  |  |  |
| LRIT3 |  |  |  |  |
| AGR2 |  |  |  |  |
| ITGB6 |  |  |  |  |
| CEBPB |  |  |  |  |
| HOTTIP |  |  |  |  |
| IL1RL2 |  |  |  |  |
| LPO |  |  |  |  |
| IGFBP7 |  |  |  |  |
| APOM |  |  |  |  |
| RDX |  |  |  |  |
| CDH17 |  |  |  |  |
| CCRL2 |  |  |  |  |
| PLAA |  |  |  |  |
| PDLIM4 |  |  |  |  |
| CSMD1 |  |  |  |  |
| CD83 |  |  |  |  |
| S100A7 |  |  |  |  |
| DNAL4 |  |  |  |  |
| PRKAB1 |  |  |  |  |
| EFNB2 |  |  |  |  |
| HLA-DMB |  |  |  |  |
| LCN1 |  |  |  |  |
| CXCR6 |  |  |  |  |
| FCGRT |  |  |  |  |
| TRA |  |  |  |  |
| PRKCZ |  |  |  |  |
| TAB1 |  |  |  |  |
| TNFRSF17 |  |  |  |  |
| SSTR5 |  |  |  |  |
| RASSF1 |  |  |  |  |
| PSMD2 |  |  |  |  |
| RPS14 |  |  |  |  |
| AAGAB |  |  |  |  |
| CHEK1 |  |  |  |  |
| RO60 |  |  |  |  |
| PLA2G4F |  |  |  |  |
| CLCF1 |  |  |  |  |
| DDIT4 |  |  |  |  |
| HDAC7 |  |  |  |  |
| CES1 |  |  |  |  |
| CPAMD8 |  |  |  |  |
| ANXA7 |  |  |  |  |
| GATD3 |  |  |  |  |
| GRHL3 |  |  |  |  |
| LRIG2 |  |  |  |  |
| CD96 |  |  |  |  |
| SLC15A1 |  |  |  |  |
| EMG1 |  |  |  |  |
| HCK |  |  |  |  |
| FUT4 |  |  |  |  |
| LRP1B |  |  |  |  |
| CNTN1 |  |  |  |  |
| SYVN1 |  |  |  |  |
| C1orf141 |  |  |  |  |
| GPR65 |  |  |  |  |
| LAG3 |  |  |  |  |
| EIF4H |  |  |  |  |
| STK24 |  |  |  |  |
| FLVCR2 |  |  |  |  |
| TAPBPL |  |  |  |  |
| CCL8 |  |  |  |  |
| IRS4 |  |  |  |  |
| RALGAPA1 |  |  |  |  |
| DNMBP |  |  |  |  |
| CYP2J2 |  |  |  |  |
| THSD7A |  |  |  |  |
| NICN1 |  |  |  |  |
| CREM |  |  |  |  |
| MIR490 |  |  |  |  |
| UCA1 |  |  |  |  |
| BCYRN1 |  |  |  |  |
| WNT11 |  |  |  |  |
| LYRM7 |  |  |  |  |
| PSMD14 |  |  |  |  |
| CCL24 |  |  |  |  |
| TCN1 |  |  |  |  |
| UBA7 |  |  |  |  |
| LYN |  |  |  |  |
| HSD17B1 |  |  |  |  |
| AKR1C3 |  |  |  |  |
| POLB |  |  |  |  |
| TRIM5 |  |  |  |  |
| MCM2 |  |  |  |  |
| MMP11 |  |  |  |  |
| CNR2 |  |  |  |  |
| GABRA5 |  |  |  |  |
| H4-16 |  |  |  |  |
| MIR542 |  |  |  |  |
| MAML2 |  |  |  |  |
| SKAP2 |  |  |  |  |
| TNFRSF14 |  |  |  |  |
| EDN2 |  |  |  |  |
| PAK1 |  |  |  |  |
| DGCR5 |  |  |  |  |
| MIR3936HG |  |  |  |  |
| H3-2 |  |  |  |  |
| ST8SIA4 |  |  |  |  |
| PSMA7 |  |  |  |  |
| LYVE1 |  |  |  |  |
| ANGPTL4 |  |  |  |  |
| ETS2 |  |  |  |  |
| MROS |  |  |  |  |
| EPHA1 |  |  |  |  |
| STIP1 |  |  |  |  |
| SLC51B |  |  |  |  |
| TNK2 |  |  |  |  |
| REV3L |  |  |  |  |
| TOR1B |  |  |  |  |
| LIN28B |  |  |  |  |
| IQCE |  |  |  |  |
| DUSP1 |  |  |  |  |
| PNPLA8 |  |  |  |  |
| VIPR1 |  |  |  |  |
| IL1R2 |  |  |  |  |
| FCHSD2 |  |  |  |  |
| TENM3 |  |  |  |  |
| PTBP1 |  |  |  |  |
| TFF2 |  |  |  |  |
| FAP |  |  |  |  |
| ARCN1 |  |  |  |  |
| NSMCE2 |  |  |  |  |
| ADRA1A |  |  |  |  |
| PLCB3 |  |  |  |  |
| PSMG1 |  |  |  |  |
| MAP2K4 |  |  |  |  |
| FUT8 |  |  |  |  |
| FAAH |  |  |  |  |
| DDX21 |  |  |  |  |
| TEC |  |  |  |  |
| MIR129-1 |  |  |  |  |
| MLKL |  |  |  |  |
| STX8 |  |  |  |  |
| IL15RA |  |  |  |  |
| KIR2DL2 |  |  |  |  |
| PRICKLE2 |  |  |  |  |
| MAPKAPK2 |  |  |  |  |
| HORMAD2 |  |  |  |  |
| TNS1 |  |  |  |  |
| MIR128-2 |  |  |  |  |
| GNAI3 |  |  |  |  |
| ENTR1 |  |  |  |  |
| BCL3 |  |  |  |  |
| CRCS5 |  |  |  |  |
| CRCS6 |  |  |  |  |
| PPT2 |  |  |  |  |
| CRCS2 |  |  |  |  |
| VPS4B |  |  |  |  |
| GADD45A |  |  |  |  |
| FNIP1 |  |  |  |  |
| IGSF6-DREV1 |  |  |  |  |
| CCL19 |  |  |  |  |
| ARAF |  |  |  |  |
| MARVELD2 |  |  |  |  |
| XPNPEP1 |  |  |  |  |
| P2RY2 |  |  |  |  |
| RAB5B |  |  |  |  |
| CRCS11 |  |  |  |  |
| CRCS9 |  |  |  |  |
| PTGER2 |  |  |  |  |
| MIR219A1 |  |  |  |  |
| RRM2 |  |  |  |  |
| TERF2 |  |  |  |  |
| NRON |  |  |  |  |
| SERPINB2 |  |  |  |  |
| KLF5 |  |  |  |  |
| LNPEP |  |  |  |  |
| RAPGEF3 |  |  |  |  |
| GRIN3B |  |  |  |  |
| HSPE1 |  |  |  |  |
| TTF2 |  |  |  |  |
| DENND1B |  |  |  |  |
| CCDC8 |  |  |  |  |
| PSMG2 |  |  |  |  |
| ENPP2 |  |  |  |  |
| RMI2 |  |  |  |  |
| LAMA5 |  |  |  |  |
| MIR642A |  |  |  |  |
| SPHK2 |  |  |  |  |
| LY75 |  |  |  |  |
| DPM3 |  |  |  |  |
| LAMP3 |  |  |  |  |
| CRCS7 |  |  |  |  |
| CRCS8 |  |  |  |  |
| RNF186 |  |  |  |  |
| IL25 |  |  |  |  |
| RBMX |  |  |  |  |
| USP25 |  |  |  |  |
| ARHGEF6 |  |  |  |  |
| WNT16 |  |  |  |  |
| PSMA2 |  |  |  |  |
| RAB1B |  |  |  |  |
| MTX1 |  |  |  |  |
| CCHCR1 |  |  |  |  |
| CLCA1 |  |  |  |  |
| HCP5 |  |  |  |  |
| POLR3H |  |  |  |  |
| GART |  |  |  |  |
| ESRRA |  |  |  |  |
| ADORA3 |  |  |  |  |
| PLA2G5 |  |  |  |  |
| BIRC2 |  |  |  |  |
| CLDN5 |  |  |  |  |
| CCNT1 |  |  |  |  |
| ATP2B2 |  |  |  |  |
| MRPL23 |  |  |  |  |
| POU6F2 |  |  |  |  |
| ZBTB38 |  |  |  |  |
| DEFB103A |  |  |  |  |
| PLB1 |  |  |  |  |
| TNFSF18 |  |  |  |  |
| GRM8 |  |  |  |  |
| PPP2R1B |  |  |  |  |
| SRSF6 |  |  |  |  |
| BID |  |  |  |  |
| PLK1 |  |  |  |  |
| FAM167A |  |  |  |  |
| CCL25 |  |  |  |  |
| LY96 |  |  |  |  |
| PSORS1C3 |  |  |  |  |
| ZBTB16 |  |  |  |  |
| PSMD9 |  |  |  |  |
| TNFSF14 |  |  |  |  |
| PROX1 |  |  |  |  |
| NLRP5 |  |  |  |  |
| SERPINA4 |  |  |  |  |
| FUT3 |  |  |  |  |
| SERPINB5 |  |  |  |  |
| TNFRSF12A |  |  |  |  |
| ILK |  |  |  |  |
| IGF2-AS |  |  |  |  |
| SLC25A28 |  |  |  |  |
| LIME1 |  |  |  |  |
| MGLL |  |  |  |  |
| CCDC122 |  |  |  |  |
| USP34 |  |  |  |  |
| TNIK |  |  |  |  |
| CALCOCO2 |  |  |  |  |
| NCR2 |  |  |  |  |
| PXN |  |  |  |  |
| SHMT1 |  |  |  |  |
| F11R |  |  |  |  |
| CLCA2 |  |  |  |  |
| CHGB |  |  |  |  |
| CYP2R1 |  |  |  |  |
| FCGR2C |  |  |  |  |
| E2F4 |  |  |  |  |
| SBNO2 |  |  |  |  |
| AGO2 |  |  |  |  |
| TSBP1-AS1 |  |  |  |  |
| TERF2IP |  |  |  |  |
| MEIS1 |  |  |  |  |
| VEGFB |  |  |  |  |
| TXK |  |  |  |  |
| SYMPK |  |  |  |  |
| PASK |  |  |  |  |
| MAP3K11 |  |  |  |  |
| CREB5 |  |  |  |  |
| CCL1 |  |  |  |  |
| CARS1 |  |  |  |  |
| ST13 |  |  |  |  |
| PGLYRP1 |  |  |  |  |
| MCM7 |  |  |  |  |
| TRAPPC10 |  |  |  |  |
| MTDH |  |  |  |  |
| PHETA1 |  |  |  |  |
| DNAH12 |  |  |  |  |
| NLRP6 |  |  |  |  |
| CAPZB |  |  |  |  |
| AZU1 |  |  |  |  |
| DIABLO |  |  |  |  |
| HLA-F |  |  |  |  |
| SLC7A5 |  |  |  |  |
| PIK3R3 |  |  |  |  |
| TMSB4X |  |  |  |  |
| USP4 |  |  |  |  |
| SENP7 |  |  |  |  |
| NDUFAB1 |  |  |  |  |
| IL17D |  |  |  |  |
| MATN1 |  |  |  |  |
| SLC37A1 |  |  |  |  |
| RASIP1 |  |  |  |  |
| TPM4 |  |  |  |  |
| VASP |  |  |  |  |
| FABP5 |  |  |  |  |
| HSPA6 |  |  |  |  |
| SNAPC4 |  |  |  |  |
| SSRP1 |  |  |  |  |
| ZFP36L1 |  |  |  |  |
| SELENBP1 |  |  |  |  |
| CD248 |  |  |  |  |
| LSP1 |  |  |  |  |
| ANXA6 |  |  |  |  |
| ATF3 |  |  |  |  |
| CPEB4 |  |  |  |  |
| RNF43 |  |  |  |  |
| TRIM31 |  |  |  |  |
| CCDC22 |  |  |  |  |
| TMEM17 |  |  |  |  |
| TRIM33 |  |  |  |  |
| ABCB5 |  |  |  |  |
| SLC3A2 |  |  |  |  |
| LY9 |  |  |  |  |
| CEP89 |  |  |  |  |
| MMP26 |  |  |  |  |
| DNAH10 |  |  |  |  |
| SNHG1 |  |  |  |  |
| CYP26B1 |  |  |  |  |
| MIR103A2 |  |  |  |  |
| FLOT2 |  |  |  |  |
| SUCO |  |  |  |  |
| PCAT1 |  |  |  |  |
| MUCL3 |  |  |  |  |
| THBS3 |  |  |  |  |
| CEACAM1 |  |  |  |  |
| MIR135B |  |  |  |  |
| ZPBP2 |  |  |  |  |
| HACE1 |  |  |  |  |
| HK2 |  |  |  |  |
| RNF146 |  |  |  |  |
| D2HGDH |  |  |  |  |
| PTPRD |  |  |  |  |
| TMPRSS15 |  |  |  |  |
| GSDME |  |  |  |  |
| PLCL1 |  |  |  |  |
| C1GALT1 |  |  |  |  |
| MIR497 |  |  |  |  |
| PZP |  |  |  |  |
| PTPN6 |  |  |  |  |
| PSRC1 |  |  |  |  |
| PTGES |  |  |  |  |
| GSDMA |  |  |  |  |
| GUK1 |  |  |  |  |
| IZUMO1 |  |  |  |  |
| CXCL16 |  |  |  |  |
| PSMD11 |  |  |  |  |
| IFNG-AS1 |  |  |  |  |
| GBGT1 |  |  |  |  |
| CCR5AS |  |  |  |  |
| KCNH6 |  |  |  |  |
| SOAT1 |  |  |  |  |
| NR4A1 |  |  |  |  |
| CD82 |  |  |  |  |
| CCAT1 |  |  |  |  |
| CASP5 |  |  |  |  |
| B4GALT1 |  |  |  |  |
| WNT5B |  |  |  |  |
| SELENOP |  |  |  |  |
| MAP2K5 |  |  |  |  |
| FZD8 |  |  |  |  |
| DDX6 |  |  |  |  |
| IL17C |  |  |  |  |
| XIST |  |  |  |  |
| CSN1S1 |  |  |  |  |
| SLC45A1 |  |  |  |  |
| PFKFB3 |  |  |  |  |
| MCAM |  |  |  |  |
| P4HTM |  |  |  |  |
| FGL2 |  |  |  |  |
| CD47 |  |  |  |  |
| CD99 |  |  |  |  |
| CLSTN2 |  |  |  |  |
| CA9 |  |  |  |  |
| IL22RA1 |  |  |  |  |
| LPXN |  |  |  |  |
| FLRT1 |  |  |  |  |
| CHMP4B |  |  |  |  |
| BCL2L2 |  |  |  |  |
| IL20 |  |  |  |  |
| CRHR2 |  |  |  |  |
| ANKRD49 |  |  |  |  |
| FZD1 |  |  |  |  |
| PSTPIP2 |  |  |  |  |
| LGR5 |  |  |  |  |
| LRRC32 |  |  |  |  |
| MIR31HG |  |  |  |  |
| ARRB1 |  |  |  |  |
| TBC1D1 |  |  |  |  |
| PCDH12 |  |  |  |  |
| PPP1R12C |  |  |  |  |
| ATP8B3 |  |  |  |  |
| FCGBP |  |  |  |  |
| PNOC |  |  |  |  |
| NLRP2 |  |  |  |  |
| P2RX3 |  |  |  |  |
| VEGFD |  |  |  |  |
| GALNT2 |  |  |  |  |
| MIR339 |  |  |  |  |
| GOLGB1 |  |  |  |  |
| FIBP |  |  |  |  |
| WNT9A |  |  |  |  |
| HULC |  |  |  |  |
| DOCK2 |  |  |  |  |
| KLRB1 |  |  |  |  |
| CST6 |  |  |  |  |
| LTB4R |  |  |  |  |
| LINC01475 |  |  |  |  |
| CDC25C |  |  |  |  |
| MNAT1 |  |  |  |  |
| PAPOLG |  |  |  |  |
| ACSL6 |  |  |  |  |
| ITGA1 |  |  |  |  |
| ACVR2A |  |  |  |  |
| ATP8B2 |  |  |  |  |
| POLR2E |  |  |  |  |
| SNRPD2 |  |  |  |  |
| PLK4 |  |  |  |  |
| HERPUD1 |  |  |  |  |
| P3H3 |  |  |  |  |
| SNHG3 |  |  |  |  |
| PSMF1 |  |  |  |  |
| CLEC5A |  |  |  |  |
| VWA7 |  |  |  |  |
| GZMM |  |  |  |  |
| MYOZ3 |  |  |  |  |
| SOCS2 |  |  |  |  |
| MLNR |  |  |  |  |
| WNT6 |  |  |  |  |
| SIRT2 |  |  |  |  |
| PTRHD1 |  |  |  |  |
| PPP5C |  |  |  |  |
| RPS25 |  |  |  |  |
| CCN4 |  |  |  |  |
| CRTC3 |  |  |  |  |
| RXRB |  |  |  |  |
| MAP2K3 |  |  |  |  |
| TBC1D5 |  |  |  |  |
| PMM1 |  |  |  |  |
| CTSZ |  |  |  |  |
| TRIM39 |  |  |  |  |
| SORCS2 |  |  |  |  |
| CENPT |  |  |  |  |
| ALCAM |  |  |  |  |
| RIPK3 |  |  |  |  |
| SFRP2 |  |  |  |  |
| TIMM10 |  |  |  |  |
| MIR29B2 |  |  |  |  |
| UBAC1 |  |  |  |  |
| CDC25B |  |  |  |  |
| PPP2R3C |  |  |  |  |
| SLC66A1 |  |  |  |  |
| LST1 |  |  |  |  |
| SERBP1 |  |  |  |  |
| ANAPC13 |  |  |  |  |
| FOXM1 |  |  |  |  |
| ARRB2 |  |  |  |  |
| ANO2 |  |  |  |  |
| PSORS1C2 |  |  |  |  |
| IL1RAP |  |  |  |  |
| VIL1 |  |  |  |  |
| ARIH2 |  |  |  |  |
| DPP10 |  |  |  |  |
| CBR3-AS1 |  |  |  |  |
| RALA |  |  |  |  |
| CLIC1 |  |  |  |  |
| SNRPC |  |  |  |  |
| SPRY4-IT1 |  |  |  |  |
| ADAM19 |  |  |  |  |
| CDR1-AS |  |  |  |  |
| MIR24-2 |  |  |  |  |
| YBX1 |  |  |  |  |
| FAU |  |  |  |  |
| FER |  |  |  |  |
| TINCR |  |  |  |  |
| UCN2 |  |  |  |  |
| OPLAH |  |  |  |  |
| SRRT |  |  |  |  |
| PRRC2A |  |  |  |  |
| CADPS |  |  |  |  |
| RUNX1T1 |  |  |  |  |
| RPL3 |  |  |  |  |
| SPAG17 |  |  |  |  |
| ENTPD7 |  |  |  |  |
| CES2 |  |  |  |  |
| ADRA1B |  |  |  |  |
| HMGN1 |  |  |  |  |
| YIF1A |  |  |  |  |
| GPR68 |  |  |  |  |
| TNFRSF10D |  |  |  |  |
| SLC7A10 |  |  |  |  |
| MIR372 |  |  |  |  |
| MBD4 |  |  |  |  |
| SLIT1 |  |  |  |  |
| ARHGAP24 |  |  |  |  |
| LGALS3BP |  |  |  |  |
| UQCR10 |  |  |  |  |
| FAM53B |  |  |  |  |
| C2orf74 |  |  |  |  |
| DCLRE1B |  |  |  |  |
| INHBA |  |  |  |  |
| NCKIPSD |  |  |  |  |
| NCR3 |  |  |  |  |
| BRD2 |  |  |  |  |
| PSMB7 |  |  |  |  |
| SAT1 |  |  |  |  |
| SBSPON |  |  |  |  |
| ICAM5 |  |  |  |  |
| ADRA1D |  |  |  |  |
| EIF3C |  |  |  |  |
| INTS11 |  |  |  |  |
| SDF4 |  |  |  |  |
| MIRLET7F1 |  |  |  |  |
| WNT8A |  |  |  |  |
| RFT1 |  |  |  |  |
| PROSER1 |  |  |  |  |
| MAGI1 |  |  |  |  |
| PRSS8 |  |  |  |  |
| TRPV5 |  |  |  |  |
| ADCY3 |  |  |  |  |
| RARG |  |  |  |  |
| TLE1 |  |  |  |  |
| RPL6 |  |  |  |  |
| ADO |  |  |  |  |
| PBX2 |  |  |  |  |
| PDCD4 |  |  |  |  |
| ADAMTS6 |  |  |  |  |
| RPS6KA2 |  |  |  |  |
| CYP4F3 |  |  |  |  |
| PDCD1LG2 |  |  |  |  |
| ATG16L2 |  |  |  |  |
| FABP6 |  |  |  |  |
| OTOP2 |  |  |  |  |
| GPR55 |  |  |  |  |
| JUNB |  |  |  |  |
| AKAP1 |  |  |  |  |
| ANKRD30A |  |  |  |  |
| SLC22A1 |  |  |  |  |
| KCNB2 |  |  |  |  |
| SCAMP3 |  |  |  |  |
| SLC28A3 |  |  |  |  |
| CLRN1-AS1 |  |  |  |  |
| ATG4B |  |  |  |  |
| RIN1 |  |  |  |  |
| DNMT3L |  |  |  |  |
| SANBR |  |  |  |  |
| CNNM1 |  |  |  |  |
| GTF2B |  |  |  |  |
| HLA-DQB2 |  |  |  |  |
| KLKB1 |  |  |  |  |
| KRTCAP2 |  |  |  |  |
| RSPO3 |  |  |  |  |
| BTRC |  |  |  |  |
| CCDC184 |  |  |  |  |
| CD6 |  |  |  |  |
| SYT14 |  |  |  |  |
| SERPINB6 |  |  |  |  |
| UBE2V1 |  |  |  |  |
| CUTC |  |  |  |  |
| PSORS8 |  |  |  |  |
| AK3 |  |  |  |  |
| TMEM258 |  |  |  |  |
| ELMO1 |  |  |  |  |
| ARFGAP1 |  |  |  |  |
| GPAA1 |  |  |  |  |
| RASSF5 |  |  |  |  |
| EREG |  |  |  |  |
| DALRD3 |  |  |  |  |
| FES |  |  |  |  |
| RBM17 |  |  |  |  |
| MUC20 |  |  |  |  |
| GLP2R |  |  |  |  |
| RAD51B |  |  |  |  |
| EXO1 |  |  |  |  |
| CDC37 |  |  |  |  |
| HMGCS2 |  |  |  |  |
| PTGIR |  |  |  |  |
| KAT2B |  |  |  |  |
| NBR1 |  |  |  |  |
| TCF19 |  |  |  |  |
| SLC12A9 |  |  |  |  |
| CYTOR |  |  |  |  |
| PTTG1 |  |  |  |  |
| PRNT |  |  |  |  |
| PDLIM5 |  |  |  |  |
| SLCO6A1 |  |  |  |  |
| TPX2 |  |  |  |  |
| SYNDIG1L |  |  |  |  |
| IL34 |  |  |  |  |
| BAG6 |  |  |  |  |
| ASIC2 |  |  |  |  |
| ACADL |  |  |  |  |
| LDHD |  |  |  |  |
| CD84 |  |  |  |  |
| ELOVL6 |  |  |  |  |
| TPPP |  |  |  |  |
| SPRED2 |  |  |  |  |
| ARID5B |  |  |  |  |
| PANX1 |  |  |  |  |
| UBXN10 |  |  |  |  |
| IL32 |  |  |  |  |
| FNDC4 |  |  |  |  |
| MRPS27 |  |  |  |  |
| PHB2 |  |  |  |  |
| ADCY7 |  |  |  |  |
| NDFIP1 |  |  |  |  |
| LRG1 |  |  |  |  |
| TUFT1 |  |  |  |  |
| SULT1A2 |  |  |  |  |
| RAB37 |  |  |  |  |
| ARMH3 |  |  |  |  |
| PPIL2 |  |  |  |  |
| TIAM1 |  |  |  |  |
| VAMP3 |  |  |  |  |
| ZFP90 |  |  |  |  |
| TRAPPC4 |  |  |  |  |
| ZNF341 |  |  |  |  |
| GRM3 |  |  |  |  |
| MAPRE1 |  |  |  |  |
| FPGS |  |  |  |  |
| RNF123 |  |  |  |  |
| INKA1 |  |  |  |  |
| PLA2G2D |  |  |  |  |
| ZNF300 |  |  |  |  |
| SCG2 |  |  |  |  |
| TDRD9 |  |  |  |  |
| TEX41 |  |  |  |  |
| UNC93B1 |  |  |  |  |
| DUSP22 |  |  |  |  |
| DTYMK |  |  |  |  |
| MROH3P |  |  |  |  |
| LOC400867 |  |  |  |  |
| TSC22D3 |  |  |  |  |
| TCERG1L |  |  |  |  |
| SELENOS |  |  |  |  |
| MUC21 |  |  |  |  |
| NKAP |  |  |  |  |
| RPL13A |  |  |  |  |
| KPNA1 |  |  |  |  |
| ZPBP |  |  |  |  |
| DMRTA1 |  |  |  |  |
| PTPN13 |  |  |  |  |
| SEC16A |  |  |  |  |
| MYL9 |  |  |  |  |
| CCR10 |  |  |  |  |
| CIBAR2 |  |  |  |  |
| RPL24 |  |  |  |  |
| BNIP3 |  |  |  |  |
| CYP26A1 |  |  |  |  |
| MUC3B |  |  |  |  |
| DUS2 |  |  |  |  |
| TMED10 |  |  |  |  |
| DUSP5 |  |  |  |  |
| SNHG5 |  |  |  |  |
| CYTL1 |  |  |  |  |
| GUCA2A |  |  |  |  |
| BBC3 |  |  |  |  |
| NAB1 |  |  |  |  |
| TSPAN8 |  |  |  |  |
| GALNT12 |  |  |  |  |
| ATF6B |  |  |  |  |
| SERPINA12 |  |  |  |  |
| SPHK1 |  |  |  |  |
| ENTPD5 |  |  |  |  |
| RIC8B |  |  |  |  |
| MMD2 |  |  |  |  |
| TCF7 |  |  |  |  |
| STMN1 |  |  |  |  |
| CCDC88B |  |  |  |  |
| SCARNA6 |  |  |  |  |
| MIR95 |  |  |  |  |
| TIMP4 |  |  |  |  |
| RNF114 |  |  |  |  |
| NCAPD2 |  |  |  |  |
| LINC-ROR |  |  |  |  |
| ELF1 |  |  |  |  |
| FZD7 |  |  |  |  |
| DANCR |  |  |  |  |
| LY86 |  |  |  |  |
| PRICKLE4 |  |  |  |  |
| EDC4 |  |  |  |  |
| XCL1 |  |  |  |  |
| ITGA5 |  |  |  |  |
| TRAF3IP3 |  |  |  |  |
| BRAP |  |  |  |  |
| UHRF1 |  |  |  |  |
| SF3B2 |  |  |  |  |
| CLDN7 |  |  |  |  |
| BOD1 |  |  |  |  |
| CD48 |  |  |  |  |
| LIN54 |  |  |  |  |
| CFAP126 |  |  |  |  |
| P2RY11 |  |  |  |  |
| RETNLB |  |  |  |  |
| RNPEPL1 |  |  |  |  |
| C10orf67 |  |  |  |  |
| NMB |  |  |  |  |
| ERRFI1 |  |  |  |  |
| PTGER3 |  |  |  |  |
| ST3GAL4 |  |  |  |  |
| DOP1B |  |  |  |  |
| ITGB1BP1 |  |  |  |  |
| CSRP1 |  |  |  |  |
| NAA25 |  |  |  |  |
| MUC17 |  |  |  |  |
| SNX20 |  |  |  |  |
| VWA2 |  |  |  |  |
| EFCAB6 |  |  |  |  |
| ENPEP |  |  |  |  |
| SLC27A1 |  |  |  |  |
| SEPTIN8 |  |  |  |  |
| DDX20 |  |  |  |  |
| NUPR1 |  |  |  |  |
| MAPKAPK5 |  |  |  |  |
| CEBPE |  |  |  |  |
| CDO1 |  |  |  |  |
| MRPL9 |  |  |  |  |
| OLIG3 |  |  |  |  |
| AQP8 |  |  |  |  |
| MUC13 |  |  |  |  |
| DOT1L |  |  |  |  |
| CHP1 |  |  |  |  |
| TACR2 |  |  |  |  |
| ACIN1 |  |  |  |  |
| TRIM22 |  |  |  |  |
| CDK12 |  |  |  |  |
| CDX1 |  |  |  |  |
| GRB10 |  |  |  |  |
| IDDM15 |  |  |  |  |
| GNAZ |  |  |  |  |
| MAP3K4 |  |  |  |  |
| MIR625 |  |  |  |  |
| B3GAT1 |  |  |  |  |
| GXYLT2 |  |  |  |  |
| GNA12 |  |  |  |  |
| HNRNPM |  |  |  |  |
| HSD17B8 |  |  |  |  |
| ZFAND6 |  |  |  |  |
| GIMAP5 |  |  |  |  |
| SF3A1 |  |  |  |  |
| PTPRK |  |  |  |  |
| TDRKH |  |  |  |  |
| TNFRSF10C |  |  |  |  |
| LINC00460 |  |  |  |  |
| CDHR3 |  |  |  |  |
| IL11RA |  |  |  |  |
| LOC110973015 |  |  |  |  |
| PSG2 |  |  |  |  |
| IP6K1 |  |  |  |  |
| SLC43A3 |  |  |  |  |
| CLDN8 |  |  |  |  |
| SDK1 |  |  |  |  |
| PTENP1 |  |  |  |  |
| RABEP2 |  |  |  |  |
| UBE2L6 |  |  |  |  |
| ITGB5 |  |  |  |  |
| CD74 |  |  |  |  |
| PDGFD |  |  |  |  |
| KCP |  |  |  |  |
| USP20 |  |  |  |  |
| TMEM59 |  |  |  |  |
| OLIG1 |  |  |  |  |
| LUZP2 |  |  |  |  |
| ZFAS1 |  |  |  |  |
| HEATR3 |  |  |  |  |
| SEPTIN1 |  |  |  |  |
| CD300LF |  |  |  |  |
| VPS28 |  |  |  |  |
| QKI |  |  |  |  |
| CISD1 |  |  |  |  |
| SFRP1 |  |  |  |  |
| NLRP13 |  |  |  |  |
| ZNRD2 |  |  |  |  |
| ATP6V1G2 |  |  |  |  |
| MED1 |  |  |  |  |
| CNN1 |  |  |  |  |
| BYSL |  |  |  |  |
| GGH |  |  |  |  |
| TRPT1 |  |  |  |  |
| ADAM15 |  |  |  |  |
| ASCL2 |  |  |  |  |
| BAG1 |  |  |  |  |
| FAM189B |  |  |  |  |
| MRGPRG-AS1 |  |  |  |  |
| SSR2 |  |  |  |  |
| PRM2 |  |  |  |  |
| IP6K3 |  |  |  |  |
| IGSF3 |  |  |  |  |
| CELF3 |  |  |  |  |
| RGS14 |  |  |  |  |
| ATP6V0A1 |  |  |  |  |
| ZFP36L2 |  |  |  |  |
| GPR183 |  |  |  |  |
| MIR340 |  |  |  |  |
| DBP |  |  |  |  |
| MTA1 |  |  |  |  |
| ZNRF1 |  |  |  |  |
| AKAP12 |  |  |  |  |
| SEC31B |  |  |  |  |
| MAD2L1 |  |  |  |  |
| MPPED2 |  |  |  |  |
| NRBP1 |  |  |  |  |
| FAM168A |  |  |  |  |
| SULF1 |  |  |  |  |
| KSR1 |  |  |  |  |
| YES1 |  |  |  |  |
| NTSR1 |  |  |  |  |
| SEPHS2 |  |  |  |  |
| PRKAR2A |  |  |  |  |
| AQP10 |  |  |  |  |
| CSE1L |  |  |  |  |
| JCHAIN |  |  |  |  |
| CARD16 |  |  |  |  |
| STK25 |  |  |  |  |
| PRMT1 |  |  |  |  |
| ABI3 |  |  |  |  |
| C17orf67 |  |  |  |  |
| WRAP73 |  |  |  |  |
| FCRL6 |  |  |  |  |
| ZNF354B |  |  |  |  |
| TMEM132D |  |  |  |  |
| DCLK1 |  |  |  |  |
| WASHC5-AS1 |  |  |  |  |
| ST6GAL1 |  |  |  |  |
| WBP11 |  |  |  |  |
| PSMB11 |  |  |  |  |
| SLC9A2 |  |  |  |  |
| PEA15 |  |  |  |  |
| IMPDH2 |  |  |  |  |
| CLDND1 |  |  |  |  |
| MYOZ1 |  |  |  |  |
| RPL37 |  |  |  |  |
| PHTF1 |  |  |  |  |
| TRAF4 |  |  |  |  |
| TMEM50B |  |  |  |  |
| LURAP1L-AS1 |  |  |  |  |
| DAP3 |  |  |  |  |
| HAPLN3 |  |  |  |  |
| NCAPD3 |  |  |  |  |
| AGPAT1 |  |  |  |  |
| S100A11 |  |  |  |  |
| MIER1 |  |  |  |  |
| UBASH3B |  |  |  |  |
| TRPM2 |  |  |  |  |
| INKA2 |  |  |  |  |
| CLK2 |  |  |  |  |
| BCAP29 |  |  |  |  |
| HLA-H |  |  |  |  |
| MIR129-2 |  |  |  |  |
| C20orf203 |  |  |  |  |
| AP5B1 |  |  |  |  |
| CRNDE |  |  |  |  |
| MELK |  |  |  |  |
| ABCF2 |  |  |  |  |
| CXCL3 |  |  |  |  |
| ZNF354A |  |  |  |  |
| BOK |  |  |  |  |
| RUSC1-AS1 |  |  |  |  |
| SLC35C2 |  |  |  |  |
| AKR1C1 |  |  |  |  |
| HIF1A-AS1 |  |  |  |  |
| KLF3 |  |  |  |  |
| PLA2G4B |  |  |  |  |
| LOC285626 |  |  |  |  |
| PPP2R5E |  |  |  |  |
| SMURF1 |  |  |  |  |
| APOBEC3A |  |  |  |  |
| DLGAP4-AS1 |  |  |  |  |
| RBM4 |  |  |  |  |
| CACNA1I |  |  |  |  |
| TMCO4 |  |  |  |  |
| ZSCAN9 |  |  |  |  |
| CYP4A11 |  |  |  |  |
| CEP76 |  |  |  |  |
| TRAC |  |  |  |  |
| MAGEA1 |  |  |  |  |
| FOSL2 |  |  |  |  |
| GPSM3 |  |  |  |  |
| NAT9 |  |  |  |  |
| PHACTR2 |  |  |  |  |
| TRD |  |  |  |  |
| PMAIP1 |  |  |  |  |
| OLFML3 |  |  |  |  |
| NOP2 |  |  |  |  |
| SYTL1 |  |  |  |  |
| CYP21A1P |  |  |  |  |
| INTS8 |  |  |  |  |
| HNRNPD |  |  |  |  |
| EAPP |  |  |  |  |
| SLC26A6 |  |  |  |  |
| GRK6 |  |  |  |  |
| LOC111365141 |  |  |  |  |
| C4BPB |  |  |  |  |
| UNC119B |  |  |  |  |
| WBP4 |  |  |  |  |
| TSSC4 |  |  |  |  |
| POFUT2 |  |  |  |  |
| TNPO1 |  |  |  |  |
| HCG22 |  |  |  |  |
| KLRG1 |  |  |  |  |
| PRM1 |  |  |  |  |
| NFYB |  |  |  |  |
| TAF3 |  |  |  |  |
| CCDC85B |  |  |  |  |
| FNBP1 |  |  |  |  |
| MGAT5 |  |  |  |  |
| ATG2B |  |  |  |  |
| BBOX1 |  |  |  |  |
| LINC01554 |  |  |  |  |
| SLC48A1 |  |  |  |  |
| LINC00598 |  |  |  |  |
| IPMK |  |  |  |  |
| GRB7 |  |  |  |  |
| SNHG28 |  |  |  |  |
| FAM177A1 |  |  |  |  |
| IKZF4 |  |  |  |  |
| TACC3 |  |  |  |  |
| VMP1 |  |  |  |  |
| FYB1 |  |  |  |  |
| RASGRF1 |  |  |  |  |
| PRMT5 |  |  |  |  |
| NFATC2 |  |  |  |  |
| BLOC1S2 |  |  |  |  |
| ATG2A |  |  |  |  |
| MDFIC |  |  |  |  |
| TUBD1 |  |  |  |  |
| WIF1 |  |  |  |  |
| NLRP9 |  |  |  |  |
| SYNGR1 |  |  |  |  |
| JRKL |  |  |  |  |
| FCER1G |  |  |  |  |
| ARPC2 |  |  |  |  |
| CLDN18 |  |  |  |  |
| NLRP11 |  |  |  |  |
| AFAP1-AS1 |  |  |  |  |
| BMX |  |  |  |  |
| LOC102723878 |  |  |  |  |
| LINGO2 |  |  |  |  |
| SEMA6D |  |  |  |  |
| FIGNL1 |  |  |  |  |
| NKD1 |  |  |  |  |
| SLC2A13 |  |  |  |  |
| MDM4 |  |  |  |  |
| FARP2 |  |  |  |  |
| MTERF4 |  |  |  |  |
| CCAT2 |  |  |  |  |
| LINC00472 |  |  |  |  |
| CD101 |  |  |  |  |
| TP53COR1 |  |  |  |  |
| STK19 |  |  |  |  |
| COX11 |  |  |  |  |
| AGXT2 |  |  |  |  |
| HLA-DRB9 |  |  |  |  |
| TANK |  |  |  |  |
| EPDR1 |  |  |  |  |
| MAN2A2 |  |  |  |  |
| SLC23A1 |  |  |  |  |
| FOXA1 |  |  |  |  |
| KANSL2 |  |  |  |  |
| SNU13 |  |  |  |  |
| WDR6 |  |  |  |  |
| TRAF5 |  |  |  |  |
| PTPRT |  |  |  |  |
| CEACAM7 |  |  |  |  |
| CD276 |  |  |  |  |
| HCST |  |  |  |  |
| RPAP3 |  |  |  |  |
| IGF2BP3 |  |  |  |  |
| TAGLN2 |  |  |  |  |
| AQP12A |  |  |  |  |
| PROX2 |  |  |  |  |
| LSR |  |  |  |  |
| LIX1 |  |  |  |  |
| TOP3B |  |  |  |  |
| TNP2 |  |  |  |  |
| PRNCR1 |  |  |  |  |
| HNF1A-AS1 |  |  |  |  |
| PRELID1 |  |  |  |  |
| SLC12A5-AS1 |  |  |  |  |
| TSBP1 |  |  |  |  |
| KCTD15 |  |  |  |  |
| IGF2BP1 |  |  |  |  |
| SIRPA |  |  |  |  |
| TUSC7 |  |  |  |  |
| FCRLA |  |  |  |  |
| TMBIM1 |  |  |  |  |
| ING5 |  |  |  |  |
| GLS2 |  |  |  |  |
| STARD10 |  |  |  |  |
| IL13RA1 |  |  |  |  |
| TSHZ2 |  |  |  |  |
| PTP4A1 |  |  |  |  |
| CTDSP1 |  |  |  |  |
| HMGB2 |  |  |  |  |
| RNF138 |  |  |  |  |
| VSIG8 |  |  |  |  |
| BRWD1 |  |  |  |  |
| NBPF3 |  |  |  |  |
| DNAJC27 |  |  |  |  |
| CBFA2T2 |  |  |  |  |
| SF3B3 |  |  |  |  |
| EFNA1 |  |  |  |  |
| DGKD |  |  |  |  |
| ACAD10 |  |  |  |  |
| ATP5MG |  |  |  |  |
| AHSA2P |  |  |  |  |
| IDO2 |  |  |  |  |
| DYDC1 |  |  |  |  |
| LURAP1L |  |  |  |  |
| SENP1 |  |  |  |  |
| KANK4 |  |  |  |  |
| SEPHS1 |  |  |  |  |
| KPNA4 |  |  |  |  |
| BRD7 |  |  |  |  |
| DEFB104A |  |  |  |  |
| TIAL1 |  |  |  |  |
| CFAP70 |  |  |  |  |
| USP1 |  |  |  |  |
| MIR3939 |  |  |  |  |
| CD200R1 |  |  |  |  |
| AAMP |  |  |  |  |
| STARD3 |  |  |  |  |
| RBM14 |  |  |  |  |
| CTSW |  |  |  |  |
| LASP1 |  |  |  |  |
| ITGA11 |  |  |  |  |
| SH2D4B |  |  |  |  |
| ZP4 |  |  |  |  |
| GPANK1 |  |  |  |  |
| DYDC2 |  |  |  |  |
| EGFL8 |  |  |  |  |
| UPK2 |  |  |  |  |
| BRMS1 |  |  |  |  |
| CBLL1 |  |  |  |  |
| RPS3AP51 |  |  |  |  |
| TRIM27 |  |  |  |  |
| PCSK7 |  |  |  |  |
| GJA10 |  |  |  |  |
| ARFRP1 |  |  |  |  |
| MDK |  |  |  |  |
| SMOX |  |  |  |  |
| IP6K2 |  |  |  |  |
| AQP7 |  |  |  |  |
| LOC645266 |  |  |  |  |
| PRG3 |  |  |  |  |
| LCE3B |  |  |  |  |
| ZDHHC23 |  |  |  |  |
| PIK3AP1 |  |  |  |  |
| ICAM4 |  |  |  |  |
| PTPRU |  |  |  |  |
| MUL1 |  |  |  |  |
| HOXB8 |  |  |  |  |
| SNX32 |  |  |  |  |
| PACSIN2 |  |  |  |  |
| LLGL1 |  |  |  |  |
| ATF1 |  |  |  |  |
| DEFA1 |  |  |  |  |
| HGFAC |  |  |  |  |
| DAP |  |  |  |  |
| LSAMP |  |  |  |  |
| RCE1 |  |  |  |  |
| SLC16A9 |  |  |  |  |
| ERBIN |  |  |  |  |
| JAKMIP1 |  |  |  |  |
| HECTD4 |  |  |  |  |
| CELSR3 |  |  |  |  |
| BANCR |  |  |  |  |
| KRTAP9-2 |  |  |  |  |
| MIR425 |  |  |  |  |
| ZNF300P1 |  |  |  |  |
| ZNF678 |  |  |  |  |
| TRIM4 |  |  |  |  |
| FBH1 |  |  |  |  |
| ALPK1 |  |  |  |  |
| ZNF831 |  |  |  |  |
| IL1F10 |  |  |  |  |
| CCDC116 |  |  |  |  |
| ERP29 |  |  |  |  |
| FZD10 |  |  |  |  |
| DCTN5 |  |  |  |  |
| EXOC2 |  |  |  |  |
| AKR7A2 |  |  |  |  |
| USP50 |  |  |  |  |
| PPP3R2 |  |  |  |  |
| ADAM30 |  |  |  |  |
| SLCO3A1 |  |  |  |  |
| HOXA11-AS |  |  |  |  |
| TPBG |  |  |  |  |
| NUMB |  |  |  |  |
| SART1 |  |  |  |  |
| SLC9A4 |  |  |  |  |
| NNT-AS1 |  |  |  |  |
| OTUD3 |  |  |  |  |
| RHOU |  |  |  |  |
| RPS6KA4 |  |  |  |  |
| PANDAR |  |  |  |  |
| CCDC91 |  |  |  |  |
| JPH4 |  |  |  |  |
| GRPR |  |  |  |  |
| PPP2R3B |  |  |  |  |
| FCHO2 |  |  |  |  |
| H1-3 |  |  |  |  |
| ECD |  |  |  |  |
| RAVER2 |  |  |  |  |
| TATDN1 |  |  |  |  |
| EGLN3 |  |  |  |  |
| SLAMF8 |  |  |  |  |
| TSPAN33 |  |  |  |  |
| EXOSC1 |  |  |  |  |
| NLRP14 |  |  |  |  |
| TIPIN |  |  |  |  |
| GPR18 |  |  |  |  |
| SUPT7L |  |  |  |  |
| MOB4 |  |  |  |  |
| DOK3 |  |  |  |  |
| ZFP36 |  |  |  |  |
| GPN1 |  |  |  |  |
| LRRC3C |  |  |  |  |
| MTSS1 |  |  |  |  |
| MXD3 |  |  |  |  |
| RFTN2 |  |  |  |  |
| SNORA54 |  |  |  |  |
| NUTM2B-AS1 |  |  |  |  |
| BOLL |  |  |  |  |
| TM9SF4 |  |  |  |  |
| SNX17 |  |  |  |  |
| STX2 |  |  |  |  |
| SLC39A11 |  |  |  |  |
| RNF126 |  |  |  |  |
| OS9 |  |  |  |  |
| ARPC1A |  |  |  |  |
| RGCC |  |  |  |  |
| PWP2 |  |  |  |  |
| FRG2C |  |  |  |  |
| RCL1 |  |  |  |  |
| SEZ6L |  |  |  |  |
| SKOR1 |  |  |  |  |
| JDP2 |  |  |  |  |
| MIR454 |  |  |  |  |
| SFMBT1 |  |  |  |  |
| ACSM3 |  |  |  |  |
| NUAK1 |  |  |  |  |
| HLTF |  |  |  |  |
| SNHG6 |  |  |  |  |
| ZDHHC1 |  |  |  |  |
| EFNA3 |  |  |  |  |
| UBE2U |  |  |  |  |
| LOC105447645 |  |  |  |  |
| ST6GALNAC2 |  |  |  |  |
| SLAIN2 |  |  |  |  |
| SULF2 |  |  |  |  |
| MIR611 |  |  |  |  |
| TRMT112 |  |  |  |  |
| BAZ1A |  |  |  |  |
| TNS4 |  |  |  |  |
| FSTL4 |  |  |  |  |
| EFR3B |  |  |  |  |
| WTAP |  |  |  |  |
| CNOT11 |  |  |  |  |
| HMGN4 |  |  |  |  |
| LY6G5B |  |  |  |  |
| DAD1 |  |  |  |  |
| PFKFB4 |  |  |  |  |
| ENTPD2 |  |  |  |  |
| LCA5L |  |  |  |  |
| ZEB1-AS1 |  |  |  |  |
| IFNA10 |  |  |  |  |
| ADGRL2 |  |  |  |  |
| TMTC2 |  |  |  |  |
| NLRP10 |  |  |  |  |
| ATP6V1G3 |  |  |  |  |
| ENDOG |  |  |  |  |
| GLYAT |  |  |  |  |
| TSPAN14 |  |  |  |  |
| PCOLCE |  |  |  |  |
| KIAA1217 |  |  |  |  |
| VSTM2B |  |  |  |  |
| LIMS1 |  |  |  |  |
| NFIL3 |  |  |  |  |
| PTGFR |  |  |  |  |
| FFAR4 |  |  |  |  |
| NOX3 |  |  |  |  |
| MIR3614 |  |  |  |  |
| NPAS4 |  |  |  |  |
| TPRG1 |  |  |  |  |
| SNX7 |  |  |  |  |
| SPATA2 |  |  |  |  |
| OLFM4 |  |  |  |  |
| CASC2 |  |  |  |  |
| GBAP1 |  |  |  |  |
| LGR6 |  |  |  |  |
| C6orf47 |  |  |  |  |
| OAZ3 |  |  |  |  |
| VPS52 |  |  |  |  |
| ZNF385D |  |  |  |  |
| TPD52L2 |  |  |  |  |
| QSOX2 |  |  |  |  |
| FOXD2-AS1 |  |  |  |  |
| NSG2 |  |  |  |  |
| CYTH4 |  |  |  |  |
| MAGI3 |  |  |  |  |
| NXPE1 |  |  |  |  |
| LIMD1 |  |  |  |  |
| CENPO |  |  |  |  |
| MPPE1 |  |  |  |  |
| NLRP8 |  |  |  |  |
| GID8 |  |  |  |  |
| EPS8L2 |  |  |  |  |
| THAP7 |  |  |  |  |
| MRPL11 |  |  |  |  |
| IFNA4 |  |  |  |  |
| MRGPRE |  |  |  |  |
| PSMG4 |  |  |  |  |
| SAPCD1 |  |  |  |  |
| SLC7A11 |  |  |  |  |
| IGLL5 |  |  |  |  |
| PRRT1 |  |  |  |  |
| GPR158 |  |  |  |  |
| LINC00492 |  |  |  |  |
| CDK11B |  |  |  |  |
| PTPRR |  |  |  |  |
| ACTR3B |  |  |  |  |
| PARVB |  |  |  |  |
| TEF |  |  |  |  |
| CD160 |  |  |  |  |
| UCKL1 |  |  |  |  |
| SLC27A6 |  |  |  |  |
| YTHDF1 |  |  |  |  |
| NFATC3 |  |  |  |  |
| PPP1R35 |  |  |  |  |
| THEM4 |  |  |  |  |
| CTIF |  |  |  |  |
| BRINP3 |  |  |  |  |
| ZBTB40 |  |  |  |  |
| SYS1 |  |  |  |  |
| CNOT9 |  |  |  |  |
| AMIGO3 |  |  |  |  |
| NCOA5 |  |  |  |  |
| PHLPP1 |  |  |  |  |
| SLC10A4 |  |  |  |  |
| ARHGAP30 |  |  |  |  |
| SNHG16 |  |  |  |  |
| TM9SF2 |  |  |  |  |
| SLC2A14 |  |  |  |  |
| UNC45A |  |  |  |  |
| SLC39A12 |  |  |  |  |
| RPL3P2 |  |  |  |  |
| MMP28 |  |  |  |  |
| ACSL5 |  |  |  |  |
| SLC30A5 |  |  |  |  |
| NDST2 |  |  |  |  |
| RNY1 |  |  |  |  |
| CASS4 |  |  |  |  |
| DDX27 |  |  |  |  |
| RSBN1 |  |  |  |  |
| RANBP10 |  |  |  |  |
| RSL1D1 |  |  |  |  |
| FAM135A |  |  |  |  |
| BORCS5 |  |  |  |  |
| GDI2 |  |  |  |  |
| PIP5K1A |  |  |  |  |
| MRPL28 |  |  |  |  |
| ZBTB46 |  |  |  |  |
| ZNF74 |  |  |  |  |
| FOXR1 |  |  |  |  |
| THEMIS |  |  |  |  |
| SORBS1 |  |  |  |  |
| PSME1 |  |  |  |  |
| PPM1G |  |  |  |  |
| CASC3 |  |  |  |  |
| PKP4 |  |  |  |  |
| MYDGF |  |  |  |  |
| ICA1 |  |  |  |  |
| SLC39A7 |  |  |  |  |
| UTP25 |  |  |  |  |
| RAB3C |  |  |  |  |
| FBXL8 |  |  |  |  |
| SLC7A6 |  |  |  |  |
| KRTDAP |  |  |  |  |
| GPSM1 |  |  |  |  |
| CGN |  |  |  |  |
| ILRUN |  |  |  |  |
| MIR345 |  |  |  |  |
| CBLC |  |  |  |  |
| UBE2Q1 |  |  |  |  |
| ZNF133 |  |  |  |  |
| SLC13A2 |  |  |  |  |
| NRAP |  |  |  |  |
| MED20 |  |  |  |  |
| CDH22 |  |  |  |  |
| DYRK2 |  |  |  |  |
| TRIM35 |  |  |  |  |
| SLC8A3 |  |  |  |  |
| MAP1S |  |  |  |  |
| QPCTL |  |  |  |  |
| AMZ1 |  |  |  |  |
| SGF29 |  |  |  |  |
| ABHD4 |  |  |  |  |
| HLA-DRB6 |  |  |  |  |
| POLE2 |  |  |  |  |
| PRXL2B |  |  |  |  |
| CCDC68 |  |  |  |  |
| RAB32 |  |  |  |  |
| ZNF266 |  |  |  |  |
| SPSB2 |  |  |  |  |
| CLEC2D |  |  |  |  |
| RC3H1 |  |  |  |  |
| GMPS |  |  |  |  |
| LINGO4 |  |  |  |  |
| HCG18 |  |  |  |  |
| NUBP1 |  |  |  |  |
| DIDO1 |  |  |  |  |
| ABRAXAS2 |  |  |  |  |
| SGCZ |  |  |  |  |
| ASGR1 |  |  |  |  |
| AURKAIP1 |  |  |  |  |
| CASC8 |  |  |  |  |
| SOCS5 |  |  |  |  |
| ENSG00000261832 |  |  |  |  |
| CSMD2 |  |  |  |  |
| TUBG2 |  |  |  |  |
| CENPW |  |  |  |  |
| MIR422A |  |  |  |  |
| COLCA2 |  |  |  |  |
| CDC42SE2 |  |  |  |  |
| TCF7L1 |  |  |  |  |
| MRPL4 |  |  |  |  |
| PTGES2 |  |  |  |  |
| UBAP2L |  |  |  |  |
| NORAD |  |  |  |  |
| SMDT1 |  |  |  |  |
| ST3GAL2 |  |  |  |  |
| MDGA2 |  |  |  |  |
| TRAFD1 |  |  |  |  |
| ZC3H7A |  |  |  |  |
| AKR1B10 |  |  |  |  |
| FCMR |  |  |  |  |
| CHMP5 |  |  |  |  |
| BPIFB3 |  |  |  |  |
| GSPT1 |  |  |  |  |
| SNHG20 |  |  |  |  |
| GMEB2 |  |  |  |  |
| HNF4G |  |  |  |  |
| CDCP1 |  |  |  |  |
| PTP4A3 |  |  |  |  |
| ATAD3B |  |  |  |  |
| CNGA2 |  |  |  |  |
| USP12 |  |  |  |  |
| LSM14A |  |  |  |  |
| ZSWIM8 |  |  |  |  |
| ATP6V1F |  |  |  |  |
| TAX1BP1 |  |  |  |  |
| SLC29A2 |  |  |  |  |
| HSF2 |  |  |  |  |
| CEP72 |  |  |  |  |
| KCNIP4 |  |  |  |  |
| MNX1-AS1 |  |  |  |  |
| NLRP4 |  |  |  |  |
| HLA-DQB1-AS1 |  |  |  |  |
| USO1 |  |  |  |  |
| IFNLR1 |  |  |  |  |
| C3orf62 |  |  |  |  |
| GPR137 |  |  |  |  |
| GUCA2B |  |  |  |  |
| NUDT13 |  |  |  |  |
| PNRC2 |  |  |  |  |
| RNF14 |  |  |  |  |
| HOXB6 |  |  |  |  |
| DPEP2 |  |  |  |  |
| GIGYF1 |  |  |  |  |
| PFN3 |  |  |  |  |
| PMF1 |  |  |  |  |
| GNPDA1 |  |  |  |  |
| STMN3 |  |  |  |  |
| ST3GAL1 |  |  |  |  |
| WDR43 |  |  |  |  |
| ENTPD3 |  |  |  |  |
| PHF5A |  |  |  |  |
| NBL1 |  |  |  |  |
| C9orf78 |  |  |  |  |
| SGIP1 |  |  |  |  |
| MED24 |  |  |  |  |
| FBXO24 |  |  |  |  |
| CNTNAP5 |  |  |  |  |
| TXNDC11 |  |  |  |  |
| IRGC |  |  |  |  |
| SLC5A8 |  |  |  |  |
| RAPGEF6 |  |  |  |  |
| FOXK1 |  |  |  |  |
| M1AP |  |  |  |  |
| HCN3 |  |  |  |  |
| DNAJC9 |  |  |  |  |
| ZNF512 |  |  |  |  |
| NIPAL4-DT |  |  |  |  |
| CNTNAP3 |  |  |  |  |
| PF4V1 |  |  |  |  |
| SDF2L1 |  |  |  |  |
| DRAP1 |  |  |  |  |
| LPCAT3 |  |  |  |  |
| TBCC |  |  |  |  |
| BCAR4 |  |  |  |  |
| RPP25L |  |  |  |  |
| RALB |  |  |  |  |
| NSMAF |  |  |  |  |
| CCDC61 |  |  |  |  |
| MRGPRG |  |  |  |  |
| MIR7-3 |  |  |  |  |
| IHO1 |  |  |  |  |
| LOC101929710 |  |  |  |  |
| WDCP |  |  |  |  |
| LRCH4 |  |  |  |  |
| LNCRNA-ATB |  |  |  |  |
| PTPRS |  |  |  |  |
| FBXL19-AS1 |  |  |  |  |
| TMEM174 |  |  |  |  |
| RAVER1 |  |  |  |  |
| SNHG12 |  |  |  |  |
| REEP3 |  |  |  |  |
| TDRD10 |  |  |  |  |
| ZNF366 |  |  |  |  |
| EIPR1 |  |  |  |  |
| FLJ31356 |  |  |  |  |
| DPP10-AS1 |  |  |  |  |
| MACC1 |  |  |  |  |
| EIF1AD |  |  |  |  |
| CLEC4A |  |  |  |  |
| ZFPL1 |  |  |  |  |
| DNAJC28 |  |  |  |  |
| SHISA5 |  |  |  |  |
| GPA33 |  |  |  |  |
| BATF |  |  |  |  |
| FRS3 |  |  |  |  |
| RAB13 |  |  |  |  |
| OR2B11 |  |  |  |  |
| LINC00824 |  |  |  |  |
| PHLDB2 |  |  |  |  |
| MPRIP |  |  |  |  |
| PXYLP1 |  |  |  |  |
| ASCC2 |  |  |  |  |
| LRRC2 |  |  |  |  |
| DYNLT5 |  |  |  |  |
| GPM6A |  |  |  |  |
| TCTA |  |  |  |  |
| KHDC4 |  |  |  |  |
| PANX2 |  |  |  |  |
| ANKMY1 |  |  |  |  |
| SELENOM |  |  |  |  |
| ASAP2 |  |  |  |  |
| KPNA7 |  |  |  |  |
| SNTG1 |  |  |  |  |
| PRXL2A |  |  |  |  |
| EHBP1L1 |  |  |  |  |
| SBK1 |  |  |  |  |
| ELOVL2 |  |  |  |  |
| SLC9A8 |  |  |  |  |
| MGAT3 |  |  |  |  |
| CEP131 |  |  |  |  |
| LINC00993 |  |  |  |  |
| ZCCHC10 |  |  |  |  |
| C11orf68 |  |  |  |  |
| MIR802 |  |  |  |  |
| PLXNB1 |  |  |  |  |
| CCDC51 |  |  |  |  |
| LRP11 |  |  |  |  |
| EIF3CL |  |  |  |  |
| TBC1D10A |  |  |  |  |
| IFITM4P |  |  |  |  |
| HSBP1 |  |  |  |  |
| RPL35P3 |  |  |  |  |
| ATAT1 |  |  |  |  |
| CADM3 |  |  |  |  |
| INSL5 |  |  |  |  |
| THAP11 |  |  |  |  |
| DEXI |  |  |  |  |
| POLA2 |  |  |  |  |
| CBARP |  |  |  |  |
| ABHD10 |  |  |  |  |
| LEF1-AS1 |  |  |  |  |
| BOLA2 |  |  |  |  |
| ZNF217 |  |  |  |  |
| FBXL20 |  |  |  |  |
| GPR31 |  |  |  |  |
| TFDP1 |  |  |  |  |
| MED4 |  |  |  |  |
| PCNX3 |  |  |  |  |
| MRPL52 |  |  |  |  |
| PKIG |  |  |  |  |
| ZNF507 |  |  |  |  |
| KCNN1 |  |  |  |  |
| VIM-AS1 |  |  |  |  |
| MYLK3 |  |  |  |  |
| PLA2G2E |  |  |  |  |
| ZNF384 |  |  |  |  |
| ING4 |  |  |  |  |
| KRT18P39 |  |  |  |  |
| USP19 |  |  |  |  |
| SLC43A1 |  |  |  |  |
| ZBTB9 |  |  |  |  |
| NGRN |  |  |  |  |
| CCDC71 |  |  |  |  |
| MTARC2 |  |  |  |  |
| PRPF4B |  |  |  |  |
| FER1L4 |  |  |  |  |
| SERINC3 |  |  |  |  |
| RPS14P1 |  |  |  |  |
| ABLIM1 |  |  |  |  |
| PUSL1 |  |  |  |  |
| BABAM2 |  |  |  |  |
| GPATCH1 |  |  |  |  |
| PYGO2 |  |  |  |  |
| RBM4B |  |  |  |  |
| IL17REL |  |  |  |  |
| SYPL1 |  |  |  |  |
| HIF3A |  |  |  |  |
| LINC02694 |  |  |  |  |
| MSTO2P |  |  |  |  |
| CEP192 |  |  |  |  |
| FKBP2 |  |  |  |  |
| OVGP1 |  |  |  |  |
| PRM3 |  |  |  |  |
| MIER2 |  |  |  |  |
| GPR12 |  |  |  |  |
| AGMAT |  |  |  |  |
| RPL13P2 |  |  |  |  |
| ANAPC4 |  |  |  |  |
| DCAF5 |  |  |  |  |
| DUSP18 |  |  |  |  |
| ASB6 |  |  |  |  |
| MRPS35P3 |  |  |  |  |
| CCDC69 |  |  |  |  |
| ZWILCH |  |  |  |  |
| B4GALT3 |  |  |  |  |
| ATG4C |  |  |  |  |
| CACNB1 |  |  |  |  |
| FKBP15 |  |  |  |  |
| ENDOU |  |  |  |  |
| WFDC3 |  |  |  |  |
| SYT4 |  |  |  |  |
| CLEC1B |  |  |  |  |
| GON4L |  |  |  |  |
| ZC3H4 |  |  |  |  |
| SNORD117 |  |  |  |  |
| CALU |  |  |  |  |
| LILRB4 |  |  |  |  |
| ATG4A |  |  |  |  |
| MARCHF7 |  |  |  |  |
| ZNF3 |  |  |  |  |
| WIPF2 |  |  |  |  |
| MSL1 |  |  |  |  |
| LINC01567 |  |  |  |  |
| HOXA-AS2 |  |  |  |  |
| LYZL2 |  |  |  |  |
| SLC38A10 |  |  |  |  |
| DELEC1 |  |  |  |  |
| DNAJC4 |  |  |  |  |
| DAZAP1 |  |  |  |  |
| ZC3H12C |  |  |  |  |
| AKAP11 |  |  |  |  |
| LINC00261 |  |  |  |  |
| RASSF2 |  |  |  |  |
| EIF3K |  |  |  |  |
| RBM22 |  |  |  |  |
| CPSF3 |  |  |  |  |
| OCTN3 |  |  |  |  |
| DUSP8 |  |  |  |  |
| ASB8 |  |  |  |  |
| GPATCH2L |  |  |  |  |
| RUSC1 |  |  |  |  |
| MEP1B |  |  |  |  |
| SIKE1 |  |  |  |  |
| GPR107 |  |  |  |  |
| MIR101-2 |  |  |  |  |
| CDK18 |  |  |  |  |
| RAB24 |  |  |  |  |
| MCCD1 |  |  |  |  |
| SNHG32 |  |  |  |  |
| C1QTNF12 |  |  |  |  |
| IQCH |  |  |  |  |
| RALGDS |  |  |  |  |
| MED16 |  |  |  |  |
| PCP4L1 |  |  |  |  |
| LOC100287329 |  |  |  |  |
| DUSP16 |  |  |  |  |
| CCDC146 |  |  |  |  |
| KCNK10 |  |  |  |  |
| DUSP28 |  |  |  |  |
| KLHDC1 |  |  |  |  |
| PHETA2 |  |  |  |  |
| LCE3A |  |  |  |  |
| LINC01185 |  |  |  |  |
| LINC-PINT |  |  |  |  |
| BCL2L15 |  |  |  |  |
| ABCB8 |  |  |  |  |
| UBLCP1 |  |  |  |  |
| ITLN2 |  |  |  |  |
| PILRB |  |  |  |  |
| FTX |  |  |  |  |
| PLEKHG6 |  |  |  |  |
| TAC4 |  |  |  |  |
| MK280269-056 |  |  |  |  |
| MN298114-196 |  |  |  |  |
| KLF3-AS1 |  |  |  |  |
| MIR22HG |  |  |  |  |
| INTS9 |  |  |  |  |
| STXBP4 |  |  |  |  |
| CSMD3 |  |  |  |  |
| HUNK |  |  |  |  |
| BLACAT1 |  |  |  |  |
| MEIKIN |  |  |  |  |
| SEC11C |  |  |  |  |
| CASC19 |  |  |  |  |
| RBM23 |  |  |  |  |
| SLC28A2 |  |  |  |  |
| SCUBE1 |  |  |  |  |
| PLD5 |  |  |  |  |
| SMYD2 |  |  |  |  |
| GIPC2 |  |  |  |  |
| CASC11 |  |  |  |  |
| FUT6 |  |  |  |  |
| PPAN-P2RY11 |  |  |  |  |
| FAS-AS1 |  |  |  |  |
| FUT11 |  |  |  |  |
| ETS1-AS1 |  |  |  |  |
| PRRC1 |  |  |  |  |
| USP21 |  |  |  |  |
| HOXB-AS3 |  |  |  |  |
| NACC2 |  |  |  |  |
| ZFP91 |  |  |  |  |
| ENPP7 |  |  |  |  |
| AKR7A3 |  |  |  |  |
| PHLPP2 |  |  |  |  |
| THEM5 |  |  |  |  |
| PITX1-AS1 |  |  |  |  |
| RTP5 |  |  |  |  |
| NGDN |  |  |  |  |
| ZNF532 |  |  |  |  |
| HOTAIRM1 |  |  |  |  |
| CENATAC |  |  |  |  |
| TCHHL1 |  |  |  |  |
| SLC50A1 |  |  |  |  |
| FAM98B |  |  |  |  |
| PCDHGC3 |  |  |  |  |
| VRTN |  |  |  |  |
| MAMSTR |  |  |  |  |
| IFFO1 |  |  |  |  |
| UBE3D |  |  |  |  |
| HTR3E |  |  |  |  |
| HIPK1 |  |  |  |  |
| MIR1181 |  |  |  |  |
| EFS |  |  |  |  |
| AOC4P |  |  |  |  |
| TRIM46 |  |  |  |  |
| IL22RA2 |  |  |  |  |
| EXOC3 |  |  |  |  |
| COMMD8 |  |  |  |  |
| C1orf74 |  |  |  |  |
| EIF5A2 |  |  |  |  |
| USP37 |  |  |  |  |
| GHET1 |  |  |  |  |
| BCL9L |  |  |  |  |
| ZMAT5 |  |  |  |  |
| COMMD7 |  |  |  |  |
| MORC4 |  |  |  |  |
| ITPKA |  |  |  |  |
| NAT8 |  |  |  |  |
| CFAP45 |  |  |  |  |
| L3MBTL2 |  |  |  |  |
| UMODL1 |  |  |  |  |
| CHCHD1 |  |  |  |  |
| LINC00243 |  |  |  |  |
| ADAM1A |  |  |  |  |
| ENSG00000272501 |  |  |  |  |
| ENSG00000272221 |  |  |  |  |
| RF00017-1164 |  |  |  |  |
| EEF1AKMT2 |  |  |  |  |
| TOB2 |  |  |  |  |
| SLC36A3 |  |  |  |  |
| FRMD8 |  |  |  |  |
| GCNT3 |  |  |  |  |
| CPD |  |  |  |  |
| TMEM259 |  |  |  |  |
| PDPR |  |  |  |  |
| DDX53 |  |  |  |  |
| NPTN-IT1 |  |  |  |  |
| CREBRF |  |  |  |  |
| SNHG17 |  |  |  |  |
| ENSG00000283782 |  |  |  |  |
| SLC35F4 |  |  |  |  |
| PELATON |  |  |  |  |
| MTRNR2L13 |  |  |  |  |
| MIR6090 |  |  |  |  |
| TMEM106C |  |  |  |  |
| RASL12 |  |  |  |  |
| DNAJC17 |  |  |  |  |
| TMEM116 |  |  |  |  |
| OR10AD1 |  |  |  |  |
| CATIP-AS1 |  |  |  |  |
| RASL11A |  |  |  |  |
| MICOS10 |  |  |  |  |
| UBA52P6 |  |  |  |  |
| WSPAR |  |  |  |  |
| LINC00581 |  |  |  |  |
| NXPE4 |  |  |  |  |
| GDE1 |  |  |  |  |
| KRTAP5-6 |  |  |  |  |
| DUXAP9 |  |  |  |  |
| PNMA8A |  |  |  |  |
| RXFP4 |  |  |  |  |
| LINC02863 |  |  |  |  |
| INKA2-AS1 |  |  |  |  |
| IFITM3P5 |  |  |  |  |
| IL27RA |  |  |  |  |
| DUSP2 |  |  |  |  |
| MBL1P |  |  |  |  |
| COL16A1 |  |  |  |  |
| EPB41L2 |  |  |  |  |
| CUZD1 |  |  |  |  |
| PCIF1 |  |  |  |  |
| DEDD |  |  |  |  |
| SNN |  |  |  |  |
| TSGA10IP |  |  |  |  |
| RIMBP3 |  |  |  |  |
| USP28 |  |  |  |  |
| PLA2G4E |  |  |  |  |
| DEFA1A3 |  |  |  |  |
| PCDHB7 |  |  |  |  |
| MEF2B |  |  |  |  |
| INTS6 |  |  |  |  |
| DUSP12 |  |  |  |  |
| FEZF1-AS1 |  |  |  |  |
| JAZF1-AS1 |  |  |  |  |
| POP4 |  |  |  |  |
| ZBTB17 |  |  |  |  |
| HORMAD2-AS1 |  |  |  |  |
| LINC01433 |  |  |  |  |
| TMIGD1 |  |  |  |  |
| BTF3L4P3 |  |  |  |  |
| NAA16 |  |  |  |  |
| LINC01133 |  |  |  |  |
| CRISP3 |  |  |  |  |
| TNFRSF19 |  |  |  |  |
| ENSG00000202533 |  |  |  |  |
| ENSG00000224431 |  |  |  |  |
| MG828730-053 |  |  |  |  |
| RF00017-5002 |  |  |  |  |
| NLRC3 |  |  |  |  |
| TPI1P2 |  |  |  |  |
| GPR25 |  |  |  |  |
| RNF145 |  |  |  |  |
| RNF128 |  |  |  |  |
| PSKH1 |  |  |  |  |
| FOXP4-AS1 |  |  |  |  |
| RPL17P22 |  |  |  |  |
| FABP3P2 |  |  |  |  |
| CEBPG |  |  |  |  |
| SYDE1 |  |  |  |  |
| ABCB9 |  |  |  |  |
| DNLZ |  |  |  |  |
| TTYH3 |  |  |  |  |
| FNDC3A |  |  |  |  |
| FAM171B |  |  |  |  |
| NUDT22 |  |  |  |  |
| NPSR1-AS1 |  |  |  |  |
| COLCA1 |  |  |  |  |
| VAV2 |  |  |  |  |
| CPXM2 |  |  |  |  |
| FAM118A |  |  |  |  |
| MYRF-AS1 |  |  |  |  |
| ADGB |  |  |  |  |
| PDXDC1 |  |  |  |  |
| ELL2 |  |  |  |  |
| MON2 |  |  |  |  |
| SNED1 |  |  |  |  |
| EPHB6 |  |  |  |  |
| GGTLC2 |  |  |  |  |
| CCDC157 |  |  |  |  |
| RIMBP3C |  |  |  |  |
| WAKMAR2 |  |  |  |  |
| B4GALT5 |  |  |  |  |
| REC114 |  |  |  |  |
| HCG23 |  |  |  |  |
| LOC100287049 |  |  |  |  |
| LINC02471 |  |  |  |  |
| RBM43 |  |  |  |  |
| ENSG00000249141 |  |  |  |  |
| SNORD16 |  |  |  |  |
| PCNPP1 |  |  |  |  |
| MTCO3P1 |  |  |  |  |
| ENSG00000260773 |  |  |  |  |
| ENSG00000271581 |  |  |  |  |
| lnc-MAP3K7-3 |  |  |  |  |
| lnc-IRF1-1 |  |  |  |  |
| lnc-FAM109A-1 |  |  |  |  |
| HSALNG0094037 |  |  |  |  |
| MN298214 |  |  |  |  |
| HSALNG0094038 |  |  |  |  |
| HSALNG0044846 |  |  |  |  |
| HSALNG0044847 |  |  |  |  |
| piR-48007 |  |  |  |  |
| piR-51327 |  |  |  |  |
| piR-36455 |  |  |  |  |
| piR-38259 |  |  |  |  |
| piR-50346 |  |  |  |  |
| piR-51449 |  |  |  |  |
| piR-56480-015 |  |  |  |  |
| LINC02042 |  |  |  |  |
| PLCXD2 |  |  |  |  |
| PYROXD2 |  |  |  |  |
| FAM99B |  |  |  |  |
| TTPAL |  |  |  |  |
| SPATA25 |  |  |  |  |
| DNAI4 |  |  |  |  |
| POU5F1P4 |  |  |  |  |
| RPS12P16 |  |  |  |  |
| RPL30P13 |  |  |  |  |
| TMEM89 |  |  |  |  |
| CYLD-AS1 |  |  |  |  |
| SMIM3 |  |  |  |  |
| C13orf42 |  |  |  |  |
| REM2 |  |  |  |  |
| SLCO2B1 |  |  |  |  |
| SLC16A7 |  |  |  |  |
| RPL12P7 |  |  |  |  |
| MRPL11P2 |  |  |  |  |
| TP53TG1 |  |  |  |  |
| KRTAP5-5 |  |  |  |  |
| FCAMR |  |  |  |  |
| BEND7 |  |  |  |  |
| RNF183 |  |  |  |  |
| FFAR2 |  |  |  |  |
| LRRTM4 |  |  |  |  |
| HNF4A-AS1 |  |  |  |  |
| DNER |  |  |  |  |
| ENHO |  |  |  |  |
| LDOC1 |  |  |  |  |
| RNFT1 |  |  |  |  |
| RTCB |  |  |  |  |
| TOMM6 |  |  |  |  |
| GNG8 |  |  |  |  |
| DPEP3 |  |  |  |  |
| OSGIN2 |  |  |  |  |
| APOBR |  |  |  |  |
| CDHR4 |  |  |  |  |
| PTTG2 |  |  |  |  |
| RTN4RL2 |  |  |  |  |
| SECTM1 |  |  |  |  |
| RPL34-DT |  |  |  |  |
| AQP12B |  |  |  |  |
| MIR1185-1 |  |  |  |  |
| MIR1208 |  |  |  |  |
| TMA7 |  |  |  |  |
| MIR647 |  |  |  |  |
| RALGAPA2 |  |  |  |  |
| ENSG00000235888 |  |  |  |  |
| ENSG00000259605 |  |  |  |  |
| HNRNPA1P41 |  |  |  |  |
| lnc-HLA-C-2 |  |  |  |  |
| lnc-HLA-DQA1-9 |  |  |  |  |
| lnc-HLA-DRB1-7 |  |  |  |  |
| lnc-BRAP-1 |  |  |  |  |
| NONHSAG043472.2 |  |  |  |  |
| HSALNG0049423 |  |  |  |  |
| HSALNG0049424 |  |  |  |  |
| HSALNG0133104 |  |  |  |  |
| RF00994-258 |  |  |  |  |
| TENT2 |  |  |  |  |
| DEDD2 |  |  |  |  |
| NDST3 |  |  |  |  |
| NPIPB7 |  |  |  |  |
| GAPLINC |  |  |  |  |
| TMEM238L |  |  |  |  |
| ENSG00000250264 |  |  |  |  |
| ENSG00000124593 |  |  |  |  |
| CSDC2 |  |  |  |  |
| RNY3 |  |  |  |  |
| C1orf53 |  |  |  |  |
| SPATA48 |  |  |  |  |
| PPP1R14B |  |  |  |  |
| PIM3 |  |  |  |  |
| MFSD1 |  |  |  |  |
| SLC35E4 |  |  |  |  |
| CDC37P1 |  |  |  |  |
| SAP25 |  |  |  |  |
| C14orf93 |  |  |  |  |
| UQCRHP1 |  |  |  |  |
| LINC02098 |  |  |  |  |
| CCND3P1 |  |  |  |  |
| BOK-AS1 |  |  |  |  |
| LINC01537 |  |  |  |  |
| KCNH8 |  |  |  |  |
| CLECL1 |  |  |  |  |
| PLAC9 |  |  |  |  |
| ENSG00000228778 |  |  |  |  |
| LOC102724748 |  |  |  |  |
| ERN2 |  |  |  |  |
| NAALADL1 |  |  |  |  |
| LINC01250 |  |  |  |  |
| MIR623 |  |  |  |  |
| IRS3P |  |  |  |  |
| TMEM255B |  |  |  |  |
| MFSD9 |  |  |  |  |
| LINC00858 |  |  |  |  |
| CFAP92 |  |  |  |  |
| MIR1908 |  |  |  |  |
| OLFML2B |  |  |  |  |
| OR7E116P |  |  |  |  |
| FLJ31104 |  |  |  |  |
| LINC02202 |  |  |  |  |
| LOC101929574 |  |  |  |  |
| LINC01882 |  |  |  |  |
| LINC01845 |  |  |  |  |
| LINC02708 |  |  |  |  |
| EBAG9P1 |  |  |  |  |
| ENSG00000199550 |  |  |  |  |
| ENSG00000247121 |  |  |  |  |
| ENSG00000224228 |  |  |  |  |
| ENSG00000266469 |  |  |  |  |
| RNU6-474P |  |  |  |  |
| RPSAP64 |  |  |  |  |
| ENSG00000271380 |  |  |  |  |
| ENSG00000268810 |  |  |  |  |
| HNRNPA1P49 |  |  |  |  |
| RPL23AP12 |  |  |  |  |
| ENSG00000242798 |  |  |  |  |
| ENSG00000219159 |  |  |  |  |
| lnc-RNASET2-2 |  |  |  |  |
| ENSG00000285616 |  |  |  |  |
| lnc-IL19-2 |  |  |  |  |
| lnc-IL31RA-5 |  |  |  |  |
| lnc-IL6ST-2 |  |  |  |  |
| lnc-RAB5B-2 |  |  |  |  |
| lnc-ZCCHC24-7 |  |  |  |  |
| MN298364 |  |  |  |  |
| HSALNG0055121 |  |  |  |  |
| piR-39701-054 |  |  |  |  |
| HSALNG0079213 |  |  |  |  |
| MN309174-057 |  |  |  |  |
| RF00017-4241 |  |  |  |  |
| LOC105378119 |  |  |  |  |
| HSALNG0023605 |  |  |  |  |
| HSALNG0023609 |  |  |  |  |
| ENSG00000285552 |  |  |  |  |
| lnc-IL12B-2 |  |  |  |  |
| lnc-CEP76-2 |  |  |  |  |
| HSALNG0106744 |  |  |  |  |
| HSALNG0133110 |  |  |  |  |
| HSALNG0020027 |  |  |  |  |
| HSALNG0069758 |  |  |  |  |
| piR-43099-059 |  |  |  |  |
| piR-61240-151 |  |  |  |  |
| RF00001-253 |  |  |  |  |
| piR-48325-111 |  |  |  |  |
| NONHSAG017238.2 |  |  |  |  |
| MN309188 |  |  |  |  |
| HSALNG0079128 |  |  |  |  |
| RF00017-5251 |  |  |  |  |
| LOC105372877 |  |  |  |  |
| HSALNG0046199 |  |  |  |  |
| HSALNG0091478 |  |  |  |  |
| HSALNG0041369 |  |  |  |  |
| piR-40398 |  |  |  |  |
| piR-58297-114 |  |  |  |  |
| NONHSAG045795.2 |  |  |  |  |
| HSALNG0013825 |  |  |  |  |
| CPTP |  |  |  |  |
| PI4KAP2 |  |  |  |  |
| TRIM41 |  |  |  |  |
| DLEU7-AS1 |  |  |  |  |
| SCARNA12 |  |  |  |  |
| HLA-DQB3 |  |  |  |  |
| HIGD1C |  |  |  |  |
| USP49 |  |  |  |  |
| DOK6 |  |  |  |  |
| CEACAM4 |  |  |  |  |
| IQANK1 |  |  |  |  |
| FAM205A |  |  |  |  |
| TMEM250 |  |  |  |  |
| FAM205BP |  |  |  |  |
| SMG1P5 |  |  |  |  |
| SOX21-AS1 |  |  |  |  |
| SLC16A10 |  |  |  |  |
| HEIH |  |  |  |  |
| SAPCD1-AS1 |  |  |  |  |
| ENSG00000284779 |  |  |  |  |
| COL19A1 |  |  |  |  |
| C11orf87 |  |  |  |  |
| NUSAP1 |  |  |  |  |
| C1orf189 |  |  |  |  |
| TRIM50 |  |  |  |  |
| RBM26 |  |  |  |  |
| BTBD8 |  |  |  |  |
| TMEM78 |  |  |  |  |
| MIR4686 |  |  |  |  |
| RHPN2 |  |  |  |  |
| NTN5 |  |  |  |  |
| ACRBP |  |  |  |  |
| SHISA6 |  |  |  |  |
| ANKHD1 |  |  |  |  |
| GALNTL6 |  |  |  |  |
| ALDH1L2 |  |  |  |  |
| HOXD3 |  |  |  |  |
| KRT38 |  |  |  |  |
| C5orf47 |  |  |  |  |
| NCRUPAR |  |  |  |  |
| GGNBP1 |  |  |  |  |
| MIR4425 |  |  |  |  |
| IGLV5-52 |  |  |  |  |
| LINC00892 |  |  |  |  |
| BSN-DT |  |  |  |  |
| LINC02341 |  |  |  |  |
| MHENCR |  |  |  |  |
| TDRKH-AS1 |  |  |  |  |
| STAG3L5P |  |  |  |  |
| ABHD17AP4 |  |  |  |  |
| RUNX3-AS1 |  |  |  |  |
| RPS19P3 |  |  |  |  |
| LOC105371083 |  |  |  |  |
| ENSG00000232937 |  |  |  |  |
| ENSG00000234630 |  |  |  |  |
| ENSG00000205414 |  |  |  |  |
| ENSG00000227598 |  |  |  |  |
| ENSG00000234389 |  |  |  |  |
| ENSG00000258559 |  |  |  |  |
| RN7SL688P |  |  |  |  |
| RNA5SP184 |  |  |  |  |
| ENSG00000252840 |  |  |  |  |
| ENSG00000184441 |  |  |  |  |
| ENSG00000230684 |  |  |  |  |
| ENSG00000253111 |  |  |  |  |
| ENSG00000259202 |  |  |  |  |
| RPL35P9 |  |  |  |  |
| RNU6-299P |  |  |  |  |
| RNU6-320P |  |  |  |  |
| LOC105372988 |  |  |  |  |
| ENSG00000234789 |  |  |  |  |
| ENSG00000201207 |  |  |  |  |
| ENSG00000238326 |  |  |  |  |
| RNU6-144P |  |  |  |  |
| RN7SKP113 |  |  |  |  |
| RN7SKP226 |  |  |  |  |
| ENSG00000271267 |  |  |  |  |
| ENSG00000236710 |  |  |  |  |
| ENSG00000252461 |  |  |  |  |
| ENSG00000237371 |  |  |  |  |
| ZYXP1 |  |  |  |  |
| ENSG00000288064 |  |  |  |  |
| ENSG00000288630 |  |  |  |  |
| ENSG00000272779 |  |  |  |  |
| lnc-IGF2-4 |  |  |  |  |
| lnc-TMEM258-1 |  |  |  |  |
| HSALNG0082198 |  |  |  |  |
| HSALNG0082195 |  |  |  |  |
| HSALNG0088101 |  |  |  |  |
| HSALNG0088102 |  |  |  |  |
| lnc-C9orf78-2 |  |  |  |  |
| lnc-MRPL23-2 |  |  |  |  |
| HSALNG0064168-001 |  |  |  |  |
| ENSG00000224988 |  |  |  |  |
| HSALNG0087578 |  |  |  |  |
| HSALNG0088100 |  |  |  |  |
| lnc-EPHX2-4 |  |  |  |  |
| lnc-FNBP1-2 |  |  |  |  |
| lnc-RAD51B-6 |  |  |  |  |
| lnc-USP20-5 |  |  |  |  |
| MK280466 |  |  |  |  |
| piR-59241 |  |  |  |  |
| NONHSAG043568.2 |  |  |  |  |
| piR-33458 |  |  |  |  |
| RF00483 |  |  |  |  |
| RF00017-5005 |  |  |  |  |
| RF00017-6441 |  |  |  |  |
| LOC105371082 |  |  |  |  |
| HSALNG0068535 |  |  |  |  |
| lnc-GOT1-1 |  |  |  |  |
| lnc-IQCH-5 |  |  |  |  |
| lnc-TTC33-6 |  |  |  |  |
| HSALNG0134866 |  |  |  |  |
| HSALNG0074774 |  |  |  |  |
| HSALNG0074773 |  |  |  |  |
| HSALNG0068536-001 |  |  |  |  |
| HSALNG0068536-002 |  |  |  |  |
| HSALNG0074775 |  |  |  |  |
| ENSG00000227836 |  |  |  |  |
| piR-47234 |  |  |  |  |
| piR-48852 |  |  |  |  |
| RF00017-3458 |  |  |  |  |
| RF00017-3670 |  |  |  |  |
| HSALNG0084556 |  |  |  |  |
| HSALNG0084557 |  |  |  |  |
| HSALNG0098906 |  |  |  |  |
| HSALNG0098908 |  |  |  |  |
| lnc-TMEM258-2-001 |  |  |  |  |
| piR-30396 |  |  |  |  |
| RF00017-4245 |  |  |  |  |
| RF00017-7481 |  |  |  |  |
| HSALNG0068897-001 |  |  |  |  |
| HSALNG0068898 |  |  |  |  |
| HSALNG0080046 |  |  |  |  |
| HSALNG0032571-002 |  |  |  |  |
| HSALNG0032579 |  |  |  |  |
| piR-39098-183 |  |  |  |  |
| lnc-CYTL1-3 |  |  |  |  |
| piR-43107-300 |  |  |  |  |
| PPIAP9 |  |  |  |  |
| SBDSP1 |  |  |  |  |
| PELI3 |  |  |  |  |
| OVOS2 |  |  |  |  |
| PLEKHN1 |  |  |  |  |
