# Supplementary material for: Network Pharmacology-Based Strategy to Identify the Pharmacological Mechanisms of Pulsatilla Decoction against Crohn’s Disease
Source: Front Pharmacol. 2022 Apr 5;13:844685. doi: 10.3389/fphar.2022.844685 (PMC9016333; doi:10.3389/fphar.2022.844685)
Supplement: Supplementary file 1 [file DataSheet1.zip › Table (4).DOCX]

| **Supplemental Table 4. Drug-disease intersection genes** |
| --- |
| NOS2 |
| PTGS1 |
| ESR1 |
| AR |
| PPARG |
| PTGS2 |
| CA2 |
| ESR2 |
| MAPK14 |
| GSK3B |
| HSP90AA1 |
| CDK2 |
| PRSS1 |
| CCNA2 |
| NR3C2 |
| KDR |
| PTPN1 |
| TOP2A |
| DPP4 |
| PYGM |
| PPARD |
| CHEK1 |
| AKR1B1 |
| F2 |
| ACHE |
| RELA |
| NCF1 |
| CHRM3 |
| ADRA1A |
| ADRA1B |
| CHRNA2 |
| SLC6A4 |
| OPRM1 |
| CHRNA7 |
| BCL2 |
| BAX |
| CASP9 |
| JUN |
| CASP3 |
| CASP8 |
| PRKCA |
| PON1 |
| IGHG1 |
| RXRA |
| PLAU |
| LTA4H |
| MMP2 |
| MMP9 |
| CYP3A4 |
| IL4 |
| CYP2B6 |
| ADRA1D |
| HTR3A |
| FOS |
| CDKN1A |
| MAPK1 |
| IL10RA |
| EGF |
| RB1 |
| IL6R |
| TP53 |
| NFKBIA |
| ODC1 |
| TOP1 |
| RAF1 |
| SOD1 |
| MMP1 |
| HIF1A |
| STAT1 |
| RUNX1T1 |
| CDK1 |
| HSPA5 |
| ERBB2 |
| ACACA |
| HMOX1 |
| CYP1A2 |
| CAV1 |
| MYC |
| F3 |
| CYP1A1 |
| ICAM1 |
| IL1B |
| CCL2 |
| SELE |
| VCAM1 |
| PTGER3 |
| CXCL8 |
| PRKCB |
| BIRC5 |
| DUOX2 |
| NOS3 |
| HSPB1 |
| IL2RA |
| NR1I2 |
| CYP1B1 |
| CCNB1 |
| PLAT |
| THBD |
| SERPINE1 |
| COL1A1 |
| IFNG |
| ALOX5 |
| IL1A |
| MPO |
| ABCG2 |
| GSTP1 |
| NFE2L2 |
| NQO1 |
| PARP1 |
| AHR |
| PSMD3 |
| COL3A1 |
| CXCL11 |
| CXCL2 |
| DCAF5 |
| CHEK2 |
| CLDN4 |
| PPARA |
| CXCL10 |
| CHUK |
| SPP1 |
| RUNX2 |
| RASSF1 |
| E2F1 |
| CTSD |
| IGFBP3 |
| IGF2 |
| CD40LG |
| IRF1 |
| ERBB3 |
| PCOLCE |
| NPEPPS |
| HK2 |
| GSTM1 |
