# Supplementary material for: Network Pharmacology-Based Strategy to Identify the Pharmacological Mechanisms of Pulsatilla Decoction against Crohn’s Disease
Source: Front Pharmacol. 2022 Apr 5;13:844685. doi: 10.3389/fphar.2022.844685 (PMC9016333; doi:10.3389/fphar.2022.844685)
Supplement: Supplementary file 1 [file DataSheet1.zip › Table (5).DOCX]

| **Supplemental Table 5. Prepared form for the drug-compound-target-disease network** | | | |
| --- | --- | --- | --- |
| Node1 | Node2 | Net | MolName |
| MOL001978 | NOS2 | target | Aureusidin |
| MOL001978 | PTGS1 | target | Aureusidin |
| MOL001978 | ESR1 | target | Aureusidin |
| MOL001978 | AR | target | Aureusidin |
| MOL001978 | PPARG | target | Aureusidin |
| MOL001978 | PTGS2 | target | Aureusidin |
| MOL001978 | CA2 | target | Aureusidin |
| MOL001978 | ESR2 | target | Aureusidin |
| MOL001978 | MAPK14 | target | Aureusidin |
| MOL001978 | GSK3B | target | Aureusidin |
| MOL001978 | HSP90AA1 | target | Aureusidin |
| MOL001978 | CDK2 | target | Aureusidin |
| MOL001978 | PRSS1 | target | Aureusidin |
| MOL001978 | CCNA2 | target | Aureusidin |
| MOL001979 | NR3C2 | target | LAN |
| MOL001985 | PTGS1 | target | ZINC01615307 |
| MOL001985 | PTGS2 | target | ZINC01615307 |
| MOL001985 | KDR | target | ZINC01615307 |
| MOL001985 | PTPN1 | target | ZINC01615307 |
| MOL001985 | TOP2A | target | ZINC01615307 |
| MOL001985 | HSP90AA1 | target | ZINC01615307 |
| MOL001987 | TOP2A | target | β-sitosterol |
| MOL000354 | NOS2 | target | isorhamnetin |
| MOL000354 | PTGS1 | target | isorhamnetin |
| MOL000354 | ESR1 | target | isorhamnetin |
| MOL000354 | AR | target | isorhamnetin |
| MOL000354 | PPARG | target | isorhamnetin |
| MOL000354 | PTGS2 | target | isorhamnetin |
| MOL000354 | PTPN1 | target | isorhamnetin |
| MOL000354 | ESR2 | target | isorhamnetin |
| MOL000354 | DPP4 | target | isorhamnetin |
| MOL000354 | MAPK14 | target | isorhamnetin |
| MOL000354 | GSK3B | target | isorhamnetin |
| MOL000354 | HSP90AA1 | target | isorhamnetin |
| MOL000354 | CDK2 | target | isorhamnetin |
| MOL000354 | PRSS1 | target | isorhamnetin |
| MOL000354 | CCNA2 | target | isorhamnetin |
| MOL000354 | PYGM | target | isorhamnetin |
| MOL000354 | PPARD | target | isorhamnetin |
| MOL000354 | CHEK1 | target | isorhamnetin |
| MOL000354 | AKR1B1 | target | isorhamnetin |
| MOL000354 | F2 | target | isorhamnetin |
| MOL000354 | ACHE | target | isorhamnetin |
| MOL000354 | RELA | target | isorhamnetin |
| MOL000354 | NCF1 | target | isorhamnetin |
| MOL000358 | PTGS1 | target | beta-sitosterol |
| MOL000358 | PTGS2 | target | beta-sitosterol |
| MOL000358 | HSP90AA1 | target | beta-sitosterol |
| MOL000358 | CHRM3 | target | beta-sitosterol |
| MOL000358 | ADRA1A | target | beta-sitosterol |
| MOL000358 | ADRA1B | target | beta-sitosterol |
| MOL000358 | CHRNA2 | target | beta-sitosterol |
| MOL000358 | SLC6A4 | target | beta-sitosterol |
| MOL000358 | OPRM1 | target | beta-sitosterol |
| MOL000358 | CHRNA7 | target | beta-sitosterol |
| MOL000358 | BCL2 | target | beta-sitosterol |
| MOL000358 | BAX | target | beta-sitosterol |
| MOL000358 | CASP9 | target | beta-sitosterol |
| MOL000358 | JUN | target | beta-sitosterol |
| MOL000358 | CASP3 | target | beta-sitosterol |
| MOL000358 | CASP8 | target | beta-sitosterol |
| MOL000358 | PRKCA | target | beta-sitosterol |
| MOL000358 | PON1 | target | beta-sitosterol |
| MOL000449 | NR3C2 | target | Stigmasterol |
| MOL000449 | IGHG1 | target | Stigmasterol |
| MOL000449 | RXRA | target | Stigmasterol |
| MOL000449 | PTGS1 | target | Stigmasterol |
| MOL000449 | PTGS2 | target | Stigmasterol |
| MOL000449 | AKR1B1 | target | Stigmasterol |
| MOL000449 | PLAU | target | Stigmasterol |
| MOL000449 | LTA4H | target | Stigmasterol |
| MOL000449 | CHRM3 | target | Stigmasterol |
| MOL000449 | ADRA1A | target | Stigmasterol |
| MOL000449 | ADRA1B | target | Stigmasterol |
| MOL000449 | CHRNA7 | target | Stigmasterol |
| MOL001454 | NOS2 | target | berberine |
| MOL001454 | PTGS1 | target | berberine |
| MOL001454 | ESR1 | target | berberine |
| MOL001454 | AR | target | berberine |
| MOL001454 | PTGS2 | target | berberine |
| MOL001454 | RXRA | target | berberine |
| MOL001454 | HSP90AA1 | target | berberine |
| MOL001454 | PRSS1 | target | berberine |
| MOL001458 | NOS2 | target | coptisine |
| MOL001458 | PTGS1 | target | coptisine |
| MOL001458 | ESR1 | target | coptisine |
| MOL001458 | AR | target | coptisine |
| MOL001458 | PTGS2 | target | coptisine |
| MOL001458 | PRSS1 | target | coptisine |
| MOL002641 | PTGS2 | target | Phellavin_qt |
| MOL002641 | HSP90AA1 | target | Phellavin_qt |
| MOL002644 | F2 | target | Phellopterin |
| MOL002644 | PTGS2 | target | Phellopterin |
| MOL002644 | RXRA | target | Phellopterin |
| MOL002644 | ADRA1B | target | Phellopterin |
| MOL002644 | PTPN1 | target | Phellopterin |
| MOL002644 | DPP4 | target | Phellopterin |
| MOL002644 | HSP90AA1 | target | Phellopterin |
| MOL002644 | CHRNA7 | target | Phellopterin |
| MOL002651 | CHRM3 | target | Dehydrotanshinone II A |
| MOL002651 | F2 | target | Dehydrotanshinone II A |
| MOL002651 | ESR1 | target | Dehydrotanshinone II A |
| MOL002651 | AR | target | Dehydrotanshinone II A |
| MOL002651 | PPARG | target | Dehydrotanshinone II A |
| MOL002651 | PTGS2 | target | Dehydrotanshinone II A |
| MOL002651 | ACHE | target | Dehydrotanshinone II A |
| MOL002651 | ADRA1A | target | Dehydrotanshinone II A |
| MOL002651 | OPRM1 | target | Dehydrotanshinone II A |
| MOL002651 | DPP4 | target | Dehydrotanshinone II A |
| MOL002651 | CHRNA7 | target | Dehydrotanshinone II A |
| MOL002662 | PTGS1 | target | rutaecarpine |
| MOL002662 | AR | target | rutaecarpine |
| MOL002662 | PTGS2 | target | rutaecarpine |
| MOL002662 | RXRA | target | rutaecarpine |
| MOL002662 | CHEK1 | target | rutaecarpine |
| MOL002662 | MMP2 | target | rutaecarpine |
| MOL002662 | MMP9 | target | rutaecarpine |
| MOL002662 | CYP3A4 | target | rutaecarpine |
| MOL002662 | IL4 | target | rutaecarpine |
| MOL002662 | CYP2B6 | target | rutaecarpine |
| MOL002663 | RXRA | target | Skimmianin |
| MOL002663 | HSP90AA1 | target | Skimmianin |
| MOL002666 | PTGS1 | target | Chelerythrine |
| MOL002666 | PTGS2 | target | Chelerythrine |
| MOL002666 | RXRA | target | Chelerythrine |
| MOL002668 | NOS2 | target | Worenine |
| MOL002668 | PTGS1 | target | Worenine |
| MOL002668 | ESR1 | target | Worenine |
| MOL002668 | AR | target | Worenine |
| MOL002668 | PTGS2 | target | Worenine |
| MOL002668 | CHEK1 | target | Worenine |
| MOL002670 | PTGS1 | target | Cavidine |
| MOL002670 | CHRM3 | target | Cavidine |
| MOL002670 | PTGS2 | target | Cavidine |
| MOL002670 | RXRA | target | Cavidine |
| MOL002670 | ADRA1B | target | Cavidine |
| MOL002670 | ADRA1D | target | Cavidine |
| MOL002670 | TOP2A | target | Cavidine |
| MOL002670 | OPRM1 | target | Cavidine |
| MOL002670 | HSP90AA1 | target | Cavidine |
| MOL002670 | SLC6A4 | target | Cavidine |
| MOL002672 | F2 | target | Hericenone H |
| MOL000785 | NOS2 | target | palmatine |
| MOL000785 | PTGS1 | target | palmatine |
| MOL000785 | ESR1 | target | palmatine |
| MOL000785 | AR | target | palmatine |
| MOL000785 | PTGS2 | target | palmatine |
| MOL000785 | RXRA | target | palmatine |
| MOL000785 | ESR2 | target | palmatine |
| MOL000785 | HSP90AA1 | target | palmatine |
| MOL000785 | PRSS1 | target | palmatine |
| MOL000785 | CDK2 | target | palmatine |
| MOL002894 | NOS2 | target | berberrubine |
| MOL002894 | PTGS1 | target | berberrubine |
| MOL002894 | ESR1 | target | berberrubine |
| MOL002894 | AR | target | berberrubine |
| MOL002894 | PTGS2 | target | berberrubine |
| MOL002894 | RXRA | target | berberrubine |
| MOL002894 | PRSS1 | target | berberrubine |
| MOL002897 | NOS2 | target | epiberberine |
| MOL002897 | ESR1 | target | epiberberine |
| MOL002897 | AR | target | epiberberine |
| MOL002897 | PTGS2 | target | epiberberine |
| MOL002897 | RXRA | target | epiberberine |
| MOL002897 | PRSS1 | target | epiberberine |
| MOL002903 | PTGS1 | target | (R)-Canadine |
| MOL002903 | CHRM3 | target | (R)-Canadine |
| MOL002903 | PTGS2 | target | (R)-Canadine |
| MOL002903 | HTR3A | target | (R)-Canadine |
| MOL002903 | ADRA1B | target | (R)-Canadine |
| MOL002903 | ADRA1D | target | (R)-Canadine |
| MOL002903 | SLC6A4 | target | (R)-Canadine |
| MOL002903 | OPRM1 | target | (R)-Canadine |
| MOL002903 | RXRA | target | (R)-Canadine |
| MOL002903 | ADRA1A | target | (R)-Canadine |
| MOL002904 | NOS2 | target | Berlambine |
| MOL002904 | PTGS1 | target | Berlambine |
| MOL002904 | CHRM3 | target | Berlambine |
| MOL002904 | AR | target | Berlambine |
| MOL002904 | PTGS2 | target | Berlambine |
| MOL002904 | RXRA | target | Berlambine |
| MOL002904 | ADRA1B | target | Berlambine |
| MOL002904 | ADRA1D | target | Berlambine |
| MOL002904 | PRSS1 | target | Berlambine |
| MOL002907 | NR3C2 | target | Corchoroside A_qt |
| MOL000098 | PTGS1 | target | quercetin |
| MOL000098 | AR | target | quercetin |
| MOL000098 | PPARG | target | quercetin |
| MOL000098 | PTGS2 | target | quercetin |
| MOL000098 | DPP4 | target | quercetin |
| MOL000098 | AKR1B1 | target | quercetin |
| MOL000098 | PRSS1 | target | quercetin |
| MOL000098 | FOS | target | quercetin |
| MOL000098 | CDKN1A | target | quercetin |
| MOL000098 | BAX | target | quercetin |
| MOL000098 | CASP9 | target | quercetin |
| MOL000098 | PLAU | target | quercetin |
| MOL000098 | MMP2 | target | quercetin |
| MOL000098 | MMP9 | target | quercetin |
| MOL000098 | MAPK1 | target | quercetin |
| MOL000098 | IL10RA | target | quercetin |
| MOL000098 | EGF | target | quercetin |
| MOL000098 | RB1 | target | quercetin |
| MOL000098 | JUN | target | quercetin |
| MOL000098 | IL6R | target | quercetin |
| MOL000098 | CASP3 | target | quercetin |
| MOL000098 | TP53 | target | quercetin |
| MOL000098 | NFKBIA | target | quercetin |
| MOL000098 | ODC1 | target | quercetin |
| MOL000098 | CASP8 | target | quercetin |
| MOL000098 | TOP1 | target | quercetin |
| MOL000098 | RAF1 | target | quercetin |
| MOL000098 | SOD1 | target | quercetin |
| MOL000098 | PRKCA | target | quercetin |
| MOL000098 | MMP1 | target | quercetin |
| MOL000098 | HIF1A | target | quercetin |
| MOL000098 | STAT1 | target | quercetin |
| MOL000098 | RUNX1T1 | target | quercetin |
| MOL000098 | CDK1 | target | quercetin |
| MOL000098 | HSPA5 | target | quercetin |
| MOL000098 | ERBB2 | target | quercetin |
| MOL000098 | ACACA | target | quercetin |
| MOL000098 | HMOX1 | target | quercetin |
| MOL000098 | CYP3A4 | target | quercetin |
| MOL000098 | CYP1A2 | target | quercetin |
| MOL000098 | CAV1 | target | quercetin |
| MOL000098 | MYC | target | quercetin |
| MOL000098 | F3 | target | quercetin |
| MOL000098 | CYP1A1 | target | quercetin |
| MOL000098 | ICAM1 | target | quercetin |
| MOL000098 | IL1B | target | quercetin |
| MOL000098 | CCL2 | target | quercetin |
| MOL000098 | SELE | target | quercetin |
| MOL000098 | VCAM1 | target | quercetin |
| MOL000098 | PTGER3 | target | quercetin |
| MOL000098 | CXCL8 | target | quercetin |
| MOL000098 | PRKCB | target | quercetin |
| MOL000098 | BIRC5 | target | quercetin |
| MOL000098 | DUOX2 | target | quercetin |
| MOL000098 | NOS3 | target | quercetin |
| MOL000098 | HSPB1 | target | quercetin |
| MOL000098 | IL2RA | target | quercetin |
| MOL000098 | NR1I2 | target | quercetin |
| MOL000098 | CYP1B1 | target | quercetin |
| MOL000098 | CCNB1 | target | quercetin |
| MOL000098 | PLAT | target | quercetin |
| MOL000098 | THBD | target | quercetin |
| MOL000098 | SERPINE1 | target | quercetin |
| MOL000098 | COL1A1 | target | quercetin |
| MOL000098 | IFNG | target | quercetin |
| MOL000098 | ALOX5 | target | quercetin |
| MOL000098 | IL1A | target | quercetin |
| MOL000098 | MPO | target | quercetin |
| MOL000098 | TOP2A | target | quercetin |
| MOL000098 | NCF1 | target | quercetin |
| MOL000098 | ABCG2 | target | quercetin |
| MOL000098 | GSTP1 | target | quercetin |
| MOL000098 | NFE2L2 | target | quercetin |
| MOL000098 | NQO1 | target | quercetin |
| MOL000098 | PARP1 | target | quercetin |
| MOL000098 | AHR | target | quercetin |
| MOL000098 | PSMD3 | target | quercetin |
| MOL000098 | COL3A1 | target | quercetin |
| MOL000098 | CXCL11 | target | quercetin |
| MOL000098 | CXCL2 | target | quercetin |
| MOL000098 | DCAF5 | target | quercetin |
| MOL000098 | CHEK2 | target | quercetin |
| MOL000098 | CLDN4 | target | quercetin |
| MOL000098 | PPARA | target | quercetin |
| MOL000098 | PPARD | target | quercetin |
| MOL000098 | CXCL10 | target | quercetin |
| MOL000098 | CHUK | target | quercetin |
| MOL000098 | SPP1 | target | quercetin |
| MOL000098 | RUNX2 | target | quercetin |
| MOL000098 | RASSF1 | target | quercetin |
| MOL000098 | E2F1 | target | quercetin |
| MOL000098 | CTSD | target | quercetin |
| MOL000098 | IGFBP3 | target | quercetin |
| MOL000098 | IGF2 | target | quercetin |
| MOL000098 | CD40LG | target | quercetin |
| MOL000098 | IRF1 | target | quercetin |
| MOL000098 | ERBB3 | target | quercetin |
| MOL000098 | PON1 | target | quercetin |
| MOL000098 | PCOLCE | target | quercetin |
| MOL000098 | NPEPPS | target | quercetin |
| MOL000098 | HK2 | target | quercetin |
| MOL000098 | GSTM1 | target | quercetin |
| MOL006709 | PTGS2 | target | AIDS214634 |
| MOL006710 | F2 | target | 8-(beta-D-Glucopyranosyloxy)-7-hydroxy-6-methoxy-2H-1-benzopyran-2-one |
| MOL006710 | PTGS2 | target | 8-(beta-D-Glucopyranosyloxy)-7-hydroxy-6-methoxy-2H-1-benzopyran-2-one |
| MOL006710 | PTPN1 | target | 8-(beta-D-Glucopyranosyloxy)-7-hydroxy-6-methoxy-2H-1-benzopyran-2-one |
| MOL006710 | TOP2A | target | 8-(beta-D-Glucopyranosyloxy)-7-hydroxy-6-methoxy-2H-1-benzopyran-2-one |
| MOL006710 | PRSS1 | target | 8-(beta-D-Glucopyranosyloxy)-7-hydroxy-6-methoxy-2H-1-benzopyran-2-one |
