# Supplementary material for: Network Pharmacology-Based Strategy to Identify the Pharmacological Mechanisms of Pulsatilla Decoction against Crohn’s Disease
Source: Front Pharmacol. 2022 Apr 5;13:844685. doi: 10.3389/fphar.2022.844685 (PMC9016333; doi:10.3389/fphar.2022.844685)
Supplement: Supplementary file 1 [file DataSheet1.zip › Table (6).DOCX]

| **Supplemental Table 6. The original score of CytoNCA** | | | | | | |
| --- | --- | --- | --- | --- | --- | --- |
| Name | Betweenness | Closeness | Degree | Eigenvector | LAC | Network |
| ADRA1A | 0 | 0.152496626 | 2 | 2.20E-04 | 0 | 0 |
| ADRA1B | 220 | 0.179080824 | 4 | 0.002979196 | 0 | 0 |
| F2 | 1444.840986 | 0.216061185 | 22 | 0.040403921 | 1.818181818 | 6.03982684 |
| AHR | 388.3952699 | 0.222879684 | 14 | 0.071544558 | 2.857142857 | 3.569230769 |
| AR | 87.18273618 | 0.222003929 | 18 | 0.097686887 | 3.555555556 | 4.173238526 |
| CYP1B1 | 30.72203606 | 0.193493151 | 6 | 0.007601903 | 2.666666667 | 3.2 |
| RELA | 967.4023403 | 0.241452991 | 54 | 0.258933932 | 12.88888889 | 30.46023233 |
| STAT1 | 787.5890028 | 0.237394958 | 32 | 0.150402933 | 6 | 9.256200557 |
| CYP1A1 | 905.6249614 | 0.209647495 | 16 | 0.02593407 | 2 | 3.627838828 |
| ESR1 | 540.4933253 | 0.236401674 | 38 | 0.203690886 | 10.73684211 | 17.5375476 |
| HSP90AA1 | 1840.000972 | 0.243534483 | 60 | 0.236316577 | 8.4 | 25.54634419 |
| ALOX5 | 584.3539838 | 0.193825043 | 10 | 0.010856478 | 1.6 | 2.133333333 |
| LTA4H | 0 | 0.163059163 | 2 | 7.87E-04 | 0 | 0 |
| IL4 | 442.5054621 | 0.216475096 | 22 | 0.0987783 | 9.454545455 | 11.62880051 |
| CYP2B6 | 565.5288642 | 0.193162393 | 14 | 0.006646616 | 2.857142857 | 5.944100344 |
| PTGS1 | 436 | 0.168908819 | 6 | 0.001278224 | 1.333333333 | 1.6 |
| PTGS2 | 710.1642686 | 0.218568665 | 12 | 0.041796379 | 1.333333333 | 1.535353535 |
| MAPK14 | 573.9353809 | 0.235908142 | 42 | 0.22195676 | 10.66666667 | 17.9738345 |
| RB1 | 366.4367488 | 0.234927235 | 34 | 0.183550656 | 10.82352941 | 15.87104746 |
| GSK3B | 2.172072275 | 0.211214953 | 8 | 0.064771049 | 4 | 4.571428571 |
| CAV1 | 445.388786 | 0.226 | 26 | 0.080797859 | 4 | 7.112360911 |
| CASP8 | 325.9485595 | 0.224206349 | 18 | 0.089561798 | 3.555555556 | 4.530152201 |
| JUN | 866.7767912 | 0.240938166 | 56 | 0.277243853 | 13.14285714 | 30.90345653 |
| RUNX2 | 307.409703 | 0.222440945 | 24 | 0.113310531 | 4.666666667 | 5.193896156 |
| BAX | 3.702852955 | 0.20582878 | 6 | 0.033618465 | 1.333333333 | 1.6 |
| TP53 | 2071.632568 | 0.248351648 | 64 | 0.282124817 | 11.25 | 32.08244467 |
| BCL2 | 38.05172544 | 0.217726397 | 10 | 0.065312281 | 4 | 4.8 |
| MAPK1 | 1811.025225 | 0.248351648 | 54 | 0.273300618 | 12.88888889 | 27.36473401 |
| RXRA | 795.1830417 | 0.227364185 | 26 | 0.102235317 | 4 | 4.990326797 |
| BIRC5 | 86.97961629 | 0.211214953 | 18 | 0.088144995 | 5.777777778 | 6.502673797 |
| CCNB1 | 36.08332331 | 0.20071048 | 20 | 0.066532135 | 8.4 | 10.70512893 |
| CCNA2 | 34.31921151 | 0.210037175 | 20 | 0.088450424 | 11.6 | 13.6079153 |
| CASP9 | 13.91040148 | 0.204339964 | 8 | 0.036988255 | 2 | 2.285714286 |
| HIF1A | 47.63655144 | 0.229208925 | 22 | 0.166695386 | 12.36363636 | 13.43848309 |
| FOS | 281.2825733 | 0.228282828 | 34 | 0.184032202 | 9.647058824 | 13.48601444 |
| MYC | 223.0451151 | 0.235416667 | 36 | 0.231093317 | 14.44444444 | 19.71537775 |
| TOP2A | 8.233061528 | 0.188333333 | 12 | 0.03343254 | 6 | 6.545454545 |
| CDK1 | 204.676776 | 0.222879684 | 26 | 0.119206481 | 11.07692308 | 15.17839937 |
| CASP3 | 72.19500093 | 0.218992248 | 18 | 0.097745925 | 6.666666667 | 9.030812325 |
| TOP1 | 18.11114809 | 0.202146691 | 8 | 0.03363049 | 3 | 3.428571429 |
| CDKN1A | 139.1705881 | 0.227364185 | 30 | 0.171083704 | 12 | 15.69026325 |
| PARP1 | 19.68103851 | 0.206959707 | 10 | 0.047724795 | 4 | 4.825396825 |
| COL1A1 | 269.2243366 | 0.202508961 | 12 | 0.030798385 | 2 | 4.024242424 |
| IL1B | 162.8882336 | 0.216475096 | 24 | 0.104262248 | 10.33333333 | 14.12805002 |
| COL3A1 | 32.69755326 | 0.187396352 | 6 | 0.008992689 | 2.666666667 | 3.733333333 |
| CHUK | 50.0781959 | 0.215238095 | 14 | 0.074331433 | 4.571428571 | 4.923076923 |
| HMOX1 | 48.52929968 | 0.212007505 | 14 | 0.06895712 | 4.571428571 | 5.583216783 |
| KDR | 5.136819537 | 0.210037175 | 8 | 0.045129761 | 3 | 3.428571429 |
| EGF | 252.0885201 | 0.219844358 | 20 | 0.067270443 | 3.6 | 6.025730994 |
| NOS3 | 11.79229794 | 0.213207547 | 14 | 0.07932958 | 7.428571429 | 8.751248751 |
| PRKCA | 219.3225048 | 0.228282828 | 22 | 0.104218036 | 5.454545455 | 6.706322917 |
| IGFBP3 | 708.4352373 | 0.223762376 | 16 | 0.041155022 | 2 | 3.597113997 |
| PTPN1 | 3.622551425 | 0.195164076 | 6 | 0.01760474 | 1.333333333 | 1.6 |
| DPP4 | 2.950672047 | 0.204339964 | 4 | 0.026481764 | 0 | 0 |
| ESR2 | 7.740714229 | 0.212806026 | 12 | 0.077432044 | 6.666666667 | 7.272727273 |
| CCL2 | 36.11186943 | 0.208872458 | 18 | 0.086434126 | 11.11111111 | 12.89089995 |
| IL1A | 56.45985733 | 0.211610487 | 20 | 0.091487646 | 10.4 | 12.71562012 |
| CXCL2 | 5.152136752 | 0.199646643 | 10 | 0.042369623 | 7.2 | 8 |
| CXCL10 | 241.2806134 | 0.202146691 | 14 | 0.041234206 | 5.142857143 | 5.948717949 |
| CXCL8 | 60.27246281 | 0.210820896 | 18 | 0.088979609 | 10.22222222 | 11.16906623 |
| CDK2 | 37.60896397 | 0.215648855 | 18 | 0.110242516 | 11.11111111 | 11.76470588 |
| CHEK1 | 73.36094809 | 0.214828897 | 14 | 0.06246027 | 6.857142857 | 8.537062937 |
| E2F1 | 26.76831302 | 0.210037175 | 18 | 0.095793389 | 9.777777778 | 10.35294118 |
| CHEK2 | 4.458410312 | 0.201785714 | 6 | 0.029985812 | 2.666666667 | 3.2 |
| CD40LG | 2.396405229 | 0.204339964 | 8 | 0.054261141 | 5 | 5.714285714 |
| NFKBIA | 229.8663479 | 0.229208925 | 28 | 0.164806187 | 10.85714286 | 14.28155809 |
| NOS2 | 47.3774703 | 0.226907631 | 18 | 0.126384422 | 8.888888889 | 9.411764706 |
| CHRNA2 | 0 | 0.008849558 | 2 | 0 | 0 | 0 |
| CHRNA7 | 0 | 0.008849558 | 2 | 0 | 0 | 0 |
| PRKCB | 66.02952689 | 0.215238095 | 16 | 0.073582053 | 3.5 | 4.09013209 |
| MMP2 | 393.7966678 | 0.207720588 | 12 | 0.023695303 | 2.666666667 | 4.003463203 |
| PCOLCE | 0 | 0.169415292 | 4 | 0.002904675 | 2 | 2.666666667 |
| CTSD | 0 | 0.192176871 | 2 | 0.014859403 | 0 | 0 |
| CXCL11 | 0 | 0.168908819 | 2 | 0.002991491 | 0 | 0 |
| GSTM1 | 220 | 0.174652241 | 4 | 0.001907494 | 0 | 0 |
| CYP3A4 | 70.9760344 | 0.179936306 | 8 | 0.00299666 | 2 | 3.047619048 |
| GSTP1 | 30.88511396 | 0.202508961 | 4 | 0.022105021 | 0 | 0 |
| PPARA | 683.3465873 | 0.236401674 | 22 | 0.140123904 | 9.454545455 | 10.24985994 |
| CYP1A2 | 0 | 0.163059163 | 4 | 7.04E-04 | 2 | 2.666666667 |
| NR1I2 | 40.78246356 | 0.189597315 | 4 | 0.007693226 | 0 | 0 |
| IL2RA | 19.85773105 | 0.212806026 | 10 | 0.060874376 | 3.2 | 3.555555556 |
| F3 | 27.5 | 0.190878378 | 4 | 0.007877628 | 0 | 0 |
| ERBB2 | 45.08227373 | 0.206959707 | 10 | 0.041373484 | 3.2 | 4.266666667 |
| ERBB3 | 0 | 0.20035461 | 6 | 0.025199341 | 4 | 4.8 |
| IL6R | 313.6112967 | 0.20733945 | 8 | 0.03414958 | 1 | 1.142857143 |
| HSPB1 | 2.077713773 | 0.216061185 | 8 | 0.068879202 | 5 | 5.714285714 |
| PLAT | 0 | 0.178797468 | 4 | 0.003217388 | 2 | 2.666666667 |
| SERPINE1 | 1 | 0.179080824 | 6 | 0.003438629 | 2.666666667 | 4.266666667 |
| RAF1 | 86.58560577 | 0.218146718 | 12 | 0.066553101 | 4.666666667 | 5.090909091 |
| IGF2 | 41.9192028 | 0.195164076 | 8 | 0.008122181 | 4 | 4.571428571 |
| PLAU | 0 | 0.178797468 | 4 | 0.003217388 | 2 | 2.666666667 |
| THBD | 0 | 0.178515008 | 2 | 0.002962558 | 0 | 0 |
| PPARG | 24.5412042 | 0.218992248 | 12 | 0.093942083 | 4.666666667 | 5.090909091 |
| NFE2L2 | 25.39128269 | 0.20620438 | 12 | 0.05378465 | 2.666666667 | 3.345454545 |
| SPP1 | 0 | 0.149273448 | 2 | 1.40E-04 | 0 | 0 |
| NQO1 | 13.6355264 | 0.203603604 | 6 | 0.029521341 | 1.333333333 | 1.6 |
| IFNG | 163.7910921 | 0.219844358 | 18 | 0.093201153 | 5.333333333 | 6.692810458 |
| IRF1 | 7.331605827 | 0.214421252 | 10 | 0.074103139 | 4.8 | 5.333333333 |
| HSPA5 | 222.6214286 | 0.202872531 | 6 | 0.020383222 | 0 | 0 |
| NR3C2 | 0 | 0.196864111 | 2 | 0.017260803 | 0 | 0 |
| SOD1 | 0 | 0.169415292 | 2 | 0.00149104 | 0 | 0 |
| ICAM1 | 102.2733097 | 0.173846154 | 6 | 0.002813364 | 1.333333333 | 2.133333333 |
| SELE | 0 | 0.152909337 | 4 | 4.88E-04 | 2 | 2.666666667 |
| VCAM1 | 132.2251752 | 0.175738725 | 6 | 0.003850711 | 1.333333333 | 2.133333333 |
| MMP1 | 213.9634399 | 0.189279732 | 10 | 0.005554305 | 4 | 6.298412698 |
| IL10RA | 0 | 0.192832765 | 2 | 0.010973226 | 0 | 0 |
| NCF1 | 338.5916476 | 0.209647495 | 10 | 0.049397368 | 2.4 | 2.666666667 |
| PRSS1 | 0 | 0.160056657 | 4 | 5.74E-04 | 2 | 2.666666667 |
| MMP9 | 35.24771111 | 0.173579109 | 6 | 0.002190289 | 2.666666667 | 3.733333333 |
| MPO | 220 | 0.145431145 | 4 | 9.30E-05 | 0 | 0 |
| PON1 | 0 | 0.127395716 | 2 | 6.68E-06 | 0 | 0 |
| PSMD3 | 223.5235554 | 0.20323741 | 8 | 0.039379422 | 1 | 1.142857143 |
| ODC1 | 0 | 0.16966967 | 2 | 0.002871182 | 0 | 0 |
| PPARD | 0 | 0.18616145 | 2 | 0.007473172 | 0 | 0 |
