# Supplementary material for: Network Pharmacology-Based Strategy to Identify the Pharmacological Mechanisms of Pulsatilla Decoction against Crohn’s Disease
Source: Front Pharmacol. 2022 Apr 5;13:844685. doi: 10.3389/fphar.2022.844685 (PMC9016333; doi:10.3389/fphar.2022.844685)
Supplement: Supplementary file 1 [file DataSheet1.zip › Table (7).DOCX]

| **Supplemental Table 7. The first score of CytoNCA filtration** | | | | | | |
| --- | --- | --- | --- | --- | --- | --- |
| Name | Betweenness | Closeness | Degree | Eigenvector | LAC | Network |
| CHUK | 10.07131665 | 0.524590164 | 12 | 0.090919577 | 4 | 4.363636364 |
| RB1 | 25.33262201 | 0.603773585 | 22 | 0.185236111 | 9.454545455 | 12.3645898 |
| MAPK14 | 60.862258 | 0.653061224 | 30 | 0.256602764 | 12.26666667 | 17.75427317 |
| IL1B | 7.285612536 | 0.5 | 16 | 0.120196819 | 9 | 10.63682984 |
| PPARA | 5.86035816 | 0.571428571 | 16 | 0.162485838 | 9 | 9.890909091 |
| IL4 | 6.939450364 | 0.507936508 | 16 | 0.118391357 | 9.5 | 11.42874903 |
| CDKN1A | 13.3776677 | 0.592592593 | 20 | 0.170591235 | 8.8 | 10.15974078 |
| CHEK1 | 0 | 0.47761194 | 6 | 0.054075848 | 4 | 4.8 |
| HSP90AA1 | 112.9411781 | 0.666666667 | 34 | 0.230462238 | 8.941176471 | 18.40956759 |
| ESR1 | 20.36997055 | 0.603773585 | 22 | 0.220599189 | 12 | 13.20017994 |
| CXCL8 | 0.458333333 | 0.47761194 | 12 | 0.103323929 | 9.333333333 | 10.18181818 |
| CASP3 | 2.776068376 | 0.516129032 | 12 | 0.114400245 | 6.666666667 | 7.272727273 |
| CDK1 | 16.2339582 | 0.524590164 | 16 | 0.107695833 | 5.5 | 7.193162393 |
| STAT1 | 17.43016961 | 0.581818182 | 18 | 0.153372034 | 6.666666667 | 7.822660999 |
| RELA | 87.51880472 | 0.695652174 | 36 | 0.285973787 | 13.55555556 | 24.84373661 |
| MYC | 29.2141277 | 0.64 | 28 | 0.263523459 | 14.28571429 | 18.73043012 |
| IL1A | 8.323924224 | 0.507936508 | 14 | 0.107278578 | 7.428571429 | 8.27972028 |
| FOS | 54.32481097 | 0.615384615 | 26 | 0.214931622 | 9.846153846 | 13.21835146 |
| HIF1A | 8.702561708 | 0.592592593 | 20 | 0.206588477 | 12.4 | 13.75438596 |
| BIRC5 | 2.893230626 | 0.47761194 | 10 | 0.093695931 | 4.8 | 5.333333333 |
| RXRA | 4.501960784 | 0.5 | 12 | 0.101061754 | 5.333333333 | 5.818181818 |
| PRKCA | 28.70944426 | 0.571428571 | 18 | 0.123521917 | 6.222222222 | 8.738816739 |
| MAPK1 | 89.67021247 | 0.711111111 | 38 | 0.312476456 | 14.52631579 | 26.79504728 |
| EGF | 3.968627451 | 0.5 | 10 | 0.068462625 | 4 | 4.444444444 |
| TP53 | 79.6102032 | 0.680851064 | 34 | 0.282832861 | 13.41176471 | 22.53357474 |
| RAF1 | 0.666666667 | 0.484848485 | 8 | 0.074219547 | 5 | 5.714285714 |
| RUNX2 | 5.675583567 | 0.524590164 | 14 | 0.118881084 | 5.714285714 | 6.153846154 |
| HMOX1 | 8.3748779 | 0.492307692 | 10 | 0.082736999 | 4 | 4.444444444 |
| IFNG | 10.84550265 | 0.524590164 | 12 | 0.105002642 | 4.666666667 | 5.090909091 |
| JUN | 89.95232627 | 0.695652174 | 36 | 0.295445621 | 14 | 24.69596756 |
| CASP8 | 8.294871795 | 0.524590164 | 12 | 0.105236694 | 4.666666667 | 5.090909091 |
| NFKBIA | 13.89490118 | 0.592592593 | 20 | 0.189643711 | 10.8 | 12.6668543 |
| CAV1 | 8.918398268 | 0.492307692 | 10 | 0.063266255 | 3.2 | 3.555555556 |
