# Supplementary material for: Network Pharmacology-Based Strategy to Identify the Pharmacological Mechanisms of Pulsatilla Decoction against Crohn’s Disease
Source: Front Pharmacol. 2022 Apr 5;13:844685. doi: 10.3389/fphar.2022.844685 (PMC9016333; doi:10.3389/fphar.2022.844685)
Supplement: Supplementary file 1 [file DataSheet1.zip › Table (8).DOCX]

| **Supplemental Table 8. The second score of CytoNCA filtration** | | | | | | |
| --- | --- | --- | --- | --- | --- | --- |
| Name | Betweenness | Closeness | Degree | Eigenvector | LAC | Network |
| FOS | 0 | 0.714285714 | 12 | 0.253852546 | 10 | 10.90909091 |
| MAPK1 | 6.166666667 | 1 | 20 | 0.363025635 | 12.8 | 18.45466429 |
| RB1 | 0.8 | 0.666666667 | 10 | 0.197268948 | 6.4 | 7.111111111 |
| HSP90AA1 | 0.8 | 0.666666667 | 10 | 0.194026232 | 6.4 | 7.111111111 |
| ESR1 | 2.3 | 0.833333333 | 16 | 0.312584072 | 11.5 | 13.28484848 |
| TP53 | 4.1 | 0.909090909 | 18 | 0.334579974 | 12 | 15.39726679 |
| MAPK14 | 3.533333333 | 0.909090909 | 18 | 0.341519296 | 12.44444444 | 15.49518717 |
| JUN | 3.533333333 | 0.909090909 | 18 | 0.341519296 | 12.44444444 | 15.49518717 |
| RELA | 1.066666667 | 0.833333333 | 16 | 0.319667578 | 12.5 | 14.3030303 |
| NFKBIA | 0 | 0.714285714 | 12 | 0.256320506 | 10 | 10.90909091 |
| MYC | 3.7 | 0.909090909 | 18 | 0.341173828 | 12.44444444 | 15.52655971 |
