# Supplementary material for: Network Pharmacology-Based Strategy to Identify the Pharmacological Mechanisms of Pulsatilla Decoction against Crohn’s Disease
Source: Front Pharmacol. 2022 Apr 5;13:844685. doi: 10.3389/fphar.2022.844685 (PMC9016333; doi:10.3389/fphar.2022.844685)
Supplement: Supplementary file 1 [file DataSheet1.zip › Table (9).DOCX]

| **Supplemental Table 9. Pathways of KEGG enrichment analysis** | | | | | | | | |
| --- | --- | --- | --- | --- | --- | --- | --- | --- |
| ID | Description | GeneRatio | BgRatio | pvalue | p.adjust | qvalue | geneID | Count |
| hsa05417 | Lipid and atherosclerosis | 34/127 | 215/8093 | 2.04E-25 | 5.03E-23 | 1.81E-23 | PPARG/MAPK14/GSK3B/HSP90AA1/RELA/NCF1/BCL2/BAX/CASP9/JUN/CASP3/CASP8/PRKCA/RXRA/MMP9/CYP2B6/FOS/MAPK1/TP53/NFKBIA/MMP1/HSPA5/CYP1A1/ICAM1/IL1B/CCL2/SELE/VCAM1/CXCL8/NOS3/NFE2L2/CXCL2/CHUK/CD40LG | 34 |
| hsa05418 | Fluid shear stress and atherosclerosis | 28/127 | 139/8093 | 5.98E-24 | 7.35E-22 | 2.64E-22 | MAPK14/HSP90AA1/KDR/RELA/NCF1/BCL2/JUN/MMP2/MMP9/FOS/TP53/HMOX1/CAV1/ICAM1/IL1B/CCL2/SELE/VCAM1/NOS3/PLAT/THBD/IFNG/IL1A/GSTP1/NFE2L2/NQO1/CHUK/GSTM1 | 28 |
| hsa04933 | AGE-RAGE signaling pathway in diabetic complications | 24/127 | 100/8093 | 1.57E-22 | 1.29E-20 | 4.64E-21 | MAPK14/RELA/BCL2/BAX/JUN/CASP3/PRKCA/MMP2/MAPK1/STAT1/F3/ICAM1/IL1B/CCL2/SELE/VCAM1/CXCL8/PRKCB/NOS3/THBD/SERPINE1/COL1A1/IL1A/COL3A1 | 24 |
| hsa05161 | Hepatitis B | 26/127 | 162/8093 | 1.29E-19 | 7.93E-18 | 2.85E-18 | MAPK14/CDK2/CCNA2/RELA/BCL2/BAX/CASP9/JUN/CASP3/CASP8/PRKCA/MMP9/FOS/CDKN1A/MAPK1/RB1/TP53/NFKBIA/RAF1/STAT1/MYC/CXCL8/PRKCB/BIRC5/CHUK/E2F1 | 26 |
| hsa04657 | IL-17 signaling pathway | 21/127 | 94/8093 | 4.34E-19 | 2.13E-17 | 7.67E-18 | PTGS2/MAPK14/GSK3B/HSP90AA1/RELA/JUN/CASP3/CASP8/MMP9/IL4/FOS/MAPK1/NFKBIA/MMP1/IL1B/CCL2/CXCL8/IFNG/CXCL2/CXCL10/CHUK | 21 |
| hsa05215 | Prostate cancer | 21/127 | 97/8093 | 8.75E-19 | 3.59E-17 | 1.29E-17 | AR/GSK3B/HSP90AA1/CDK2/RELA/BCL2/CASP9/PLAU/MMP9/CDKN1A/MAPK1/EGF/RB1/TP53/NFKBIA/RAF1/ERBB2/PLAT/GSTP1/CHUK/E2F1 | 21 |
| hsa05207 | Chemical carcinogenesis - receptor activation | 27/127 | 212/8093 | 1.14E-17 | 4.00E-16 | 1.44E-16 | ESR1/AR/ESR2/HSP90AA1/RELA/CHRNA7/BCL2/JUN/PRKCA/RXRA/CYP3A4/CYP2B6/FOS/MAPK1/EGF/RB1/RAF1/CYP1A2/MYC/CYP1A1/PRKCB/BIRC5/CYP1B1/AHR/PPARA/E2F1/GSTM1 | 27 |
| hsa05160 | Hepatitis C | 23/127 | 157/8093 | 1.67E-16 | 5.13E-15 | 1.85E-15 | GSK3B/CDK2/RELA/BAX/CASP9/CASP3/CASP8/RXRA/CDKN1A/MAPK1/EGF/RB1/TP53/NFKBIA/RAF1/STAT1/MYC/IFNG/CLDN4/PPARA/CXCL10/CHUK/E2F1 | 23 |
| hsa05219 | Bladder cancer | 14/127 | 41/8093 | 6.53E-16 | 1.78E-14 | 6.41E-15 | MMP2/MMP9/CDKN1A/MAPK1/EGF/RB1/TP53/RAF1/MMP1/ERBB2/MYC/CXCL8/RASSF1/E2F1 | 14 |
| hsa05167 | Kaposi sarcoma-associated herpesvirus infection | 24/127 | 194/8093 | 1.80E-15 | 4.43E-14 | 1.59E-14 | PTGS2/MAPK14/GSK3B/RELA/BAX/CASP9/JUN/CASP3/CASP8/FOS/CDKN1A/MAPK1/RB1/TP53/NFKBIA/RAF1/HIF1A/STAT1/MYC/ICAM1/CXCL8/CXCL2/CHUK/E2F1 | 24 |
| hsa04668 | TNF signaling pathway | 19/127 | 112/8093 | 5.50E-15 | 1.15E-13 | 4.13E-14 | PTGS2/MAPK14/RELA/JUN/CASP3/CASP8/MMP9/FOS/MAPK1/NFKBIA/ICAM1/IL1B/CCL2/SELE/VCAM1/CXCL2/CXCL10/CHUK/IRF1 | 19 |
| hsa05163 | Human cytomegalovirus infection | 25/127 | 225/8093 | 5.61E-15 | 1.15E-13 | 4.13E-14 | PTGS2/MAPK14/GSK3B/RELA/BAX/CASP9/CASP3/CASP8/PRKCA/CDKN1A/MAPK1/IL10RA/RB1/IL6R/TP53/NFKBIA/RAF1/MYC/IL1B/CCL2/PTGER3/CXCL8/PRKCB/CHUK/E2F1 | 25 |
| hsa05140 | Leishmaniasis | 15/127 | 77/8093 | 5.78E-13 | 9.82E-12 | 3.53E-12 | NOS2/PTGS2/MAPK14/RELA/NCF1/JUN/IL4/FOS/MAPK1/NFKBIA/STAT1/IL1B/PRKCB/IFNG/IL1A | 15 |
| hsa04659 | Th17 cell differentiation | 17/127 | 108/8093 | 5.94E-13 | 9.82E-12 | 3.53E-12 | MAPK14/HSP90AA1/RELA/JUN/RXRA/IL4/FOS/MAPK1/IL6R/NFKBIA/HIF1A/STAT1/IL1B/IL2RA/IFNG/AHR/CHUK | 17 |
| hsa05222 | Small cell lung cancer | 16/127 | 92/8093 | 5.99E-13 | 9.82E-12 | 3.53E-12 | NOS2/PTGS2/CDK2/RELA/BCL2/BAX/CASP9/CASP3/RXRA/CDKN1A/RB1/TP53/NFKBIA/MYC/CHUK/E2F1 | 16 |
| hsa05164 | Influenza A | 20/127 | 172/8093 | 1.66E-12 | 2.41E-11 | 8.66E-12 | PRSS1/RELA/BAX/CASP9/CASP3/CASP8/PRKCA/MAPK1/NFKBIA/RAF1/STAT1/ICAM1/IL1B/CCL2/CXCL8/PRKCB/IFNG/IL1A/CXCL10/CHUK | 20 |
| hsa01522 | Endocrine resistance | 16/127 | 98/8093 | 1.66E-12 | 2.41E-11 | 8.66E-12 | ESR1/ESR2/MAPK14/BCL2/BAX/JUN/MMP2/MMP9/FOS/CDKN1A/MAPK1/RB1/TP53/RAF1/ERBB2/E2F1 | 16 |
| hsa04218 | Cellular senescence | 19/127 | 156/8093 | 2.72E-12 | 3.72E-11 | 1.34E-11 | MAPK14/CDK2/CCNA2/CHEK1/RELA/CDKN1A/MAPK1/RB1/TP53/RAF1/CDK1/MYC/CXCL8/CCNB1/SERPINE1/IL1A/CHEK2/E2F1/IGFBP3 | 19 |
| hsa05223 | Non-small cell lung cancer | 14/127 | 72/8093 | 3.68E-12 | 4.76E-11 | 1.71E-11 | BAX/CASP9/PRKCA/RXRA/CDKN1A/MAPK1/EGF/RB1/TP53/RAF1/ERBB2/PRKCB/RASSF1/E2F1 | 14 |
| hsa05169 | Epstein-Barr virus infection | 21/127 | 202/8093 | 3.96E-12 | 4.87E-11 | 1.75E-11 | MAPK14/CDK2/CCNA2/RELA/BCL2/BAX/CASP9/JUN/CASP3/CASP8/CDKN1A/RB1/TP53/NFKBIA/STAT1/MYC/ICAM1/PSMD3/CXCL10/CHUK/E2F1 | 21 |
| hsa04115 | p53 signaling pathway | 14/127 | 73/8093 | 4.49E-12 | 5.26E-11 | 1.89E-11 | CDK2/CHEK1/BCL2/BAX/CASP9/CASP3/CASP8/CDKN1A/TP53/CDK1/CCNB1/SERPINE1/CHEK2/IGFBP3 | 14 |
| hsa04066 | HIF-1 signaling pathway | 16/127 | 109/8093 | 9.07E-12 | 1.01E-10 | 3.64E-11 | NOS2/RELA/BCL2/PRKCA/CDKN1A/MAPK1/EGF/IL6R/HIF1A/ERBB2/HMOX1/PRKCB/NOS3/SERPINE1/IFNG/HK2 | 16 |
| hsa05208 | Chemical carcinogenesis - reactive oxygen species | 21/127 | 223/8093 | 2.68E-11 | 2.86E-10 | 1.03E-10 | MAPK14/PTPN1/RELA/NCF1/JUN/FOS/MAPK1/EGF/NFKBIA/RAF1/SOD1/HIF1A/HMOX1/CYP1A2/CYP1A1/CYP1B1/NFE2L2/NQO1/AHR/CHUK/GSTM1 | 21 |
| hsa05162 | Measles | 17/127 | 139/8093 | 3.94E-11 | 4.04E-10 | 1.45E-10 | GSK3B/CDK2/RELA/BCL2/BAX/CASP9/JUN/CASP3/CASP8/FOS/TP53/NFKBIA/STAT1/IL1B/IL2RA/IL1A/CHUK | 17 |
| hsa05205 | Proteoglycans in cancer | 20/127 | 205/8093 | 4.39E-11 | 4.32E-10 | 1.55E-10 | ESR1/MAPK14/KDR/CASP3/PRKCA/PLAU/MMP2/MMP9/CDKN1A/MAPK1/TP53/RAF1/HIF1A/ERBB2/CAV1/MYC/PRKCB/COL1A1/IGF2/ERBB3 | 20 |
| hsa05210 | Colorectal cancer | 14/127 | 86/8093 | 4.64E-11 | 4.39E-10 | 1.58E-10 | GSK3B/BCL2/BAX/CASP9/JUN/CASP3/FOS/CDKN1A/MAPK1/EGF/TP53/RAF1/MYC/BIRC5 | 14 |
| hsa01524 | Platinum drug resistance | 13/127 | 73/8093 | 7.31E-11 | 6.66E-10 | 2.39E-10 | TOP2A/BCL2/BAX/CASP9/CASP3/CASP8/CDKN1A/MAPK1/TP53/ERBB2/BIRC5/GSTP1/GSTM1 | 13 |
| hsa05212 | Pancreatic cancer | 13/127 | 76/8093 | 1.24E-10 | 1.09E-09 | 3.93E-10 | RELA/BAX/CASP9/CDKN1A/MAPK1/EGF/RB1/TP53/RAF1/STAT1/ERBB2/CHUK/E2F1 | 13 |
| hsa04151 | PI3K-Akt signaling pathway | 25/127 | 354/8093 | 1.55E-10 | 1.31E-09 | 4.73E-10 | GSK3B/HSP90AA1/CDK2/KDR/RELA/BCL2/CASP9/PRKCA/RXRA/IL4/CDKN1A/MAPK1/EGF/IL6R/TP53/RAF1/ERBB2/MYC/NOS3/IL2RA/COL1A1/CHUK/SPP1/IGF2/ERBB3 | 25 |
| hsa05145 | Toxoplasmosis | 15/127 | 112/8093 | 1.62E-10 | 1.33E-09 | 4.79E-10 | NOS2/MAPK14/RELA/BCL2/CASP9/CASP3/CASP8/MAPK1/IL10RA/NFKBIA/STAT1/IFNG/ALOX5/CHUK/CD40LG | 15 |
| hsa04210 | Apoptosis | 16/127 | 136/8093 | 2.79E-10 | 2.21E-09 | 7.96E-10 | RELA/BCL2/BAX/CASP9/JUN/CASP3/CASP8/FOS/MAPK1/TP53/NFKBIA/RAF1/BIRC5/PARP1/CHUK/CTSD | 16 |
| hsa05142 | Chagas disease | 14/127 | 102/8093 | 4.93E-10 | 3.79E-09 | 1.36E-09 | NOS2/MAPK14/RELA/JUN/CASP8/FOS/MAPK1/NFKBIA/IL1B/CCL2/CXCL8/SERPINE1/IFNG/CHUK | 14 |
| hsa04064 | NF-kappa B signaling pathway | 14/127 | 104/8093 | 6.42E-10 | 4.64E-09 | 1.67E-09 | PTGS2/RELA/BCL2/PLAU/NFKBIA/ICAM1/IL1B/VCAM1/CXCL8/PRKCB/PARP1/CXCL2/CHUK/CD40LG | 14 |
| hsa04620 | Toll-like receptor signaling pathway | 14/127 | 104/8093 | 6.42E-10 | 4.64E-09 | 1.67E-09 | MAPK14/RELA/JUN/CASP8/FOS/MAPK1/NFKBIA/STAT1/IL1B/CXCL8/CXCL11/CXCL10/CHUK/SPP1 | 14 |
| hsa05225 | Hepatocellular carcinoma | 17/127 | 168/8093 | 8.11E-10 | 5.70E-09 | 2.05E-09 | GSK3B/BAX/PRKCA/CDKN1A/MAPK1/RB1/TP53/RAF1/HMOX1/MYC/PRKCB/GSTP1/NFE2L2/NQO1/E2F1/IGF2/GSTM1 | 17 |
| hsa04926 | Relaxin signaling pathway | 15/127 | 129/8093 | 1.24E-09 | 8.45E-09 | 3.04E-09 | NOS2/MAPK14/RELA/JUN/PRKCA/MMP2/MMP9/FOS/MAPK1/NFKBIA/RAF1/MMP1/NOS3/COL1A1/COL3A1 | 15 |
| hsa01521 | EGFR tyrosine kinase inhibitor resistance | 12/127 | 79/8093 | 2.77E-09 | 1.84E-08 | 6.63E-09 | GSK3B/KDR/BCL2/BAX/PRKCA/MAPK1/EGF/IL6R/RAF1/ERBB2/PRKCB/ERBB3 | 12 |
| hsa05170 | Human immunodeficiency virus 1 infection | 18/127 | 212/8093 | 4.31E-09 | 2.79E-08 | 1.00E-08 | MAPK14/CHEK1/RELA/BCL2/BAX/CASP9/JUN/CASP3/CASP8/PRKCA/FOS/MAPK1/NFKBIA/RAF1/CDK1/PRKCB/CCNB1/CHUK | 18 |
| hsa05224 | Breast cancer | 15/127 | 147/8093 | 7.70E-09 | 4.86E-08 | 1.75E-08 | ESR1/ESR2/GSK3B/BAX/JUN/FOS/CDKN1A/MAPK1/EGF/RB1/TP53/RAF1/ERBB2/MYC/E2F1 | 15 |
| hsa05166 | Human T-cell leukemia virus 1 infection | 18/127 | 222/8093 | 8.97E-09 | 5.52E-08 | 1.98E-08 | CDK2/CCNA2/CHEK1/RELA/BAX/JUN/FOS/CDKN1A/MAPK1/RB1/TP53/NFKBIA/MYC/ICAM1/IL2RA/CHEK2/CHUK/E2F1 | 18 |
| hsa05235 | PD-L1 expression and PD-1 checkpoint pathway in cancer | 12/127 | 89/8093 | 1.13E-08 | 6.76E-08 | 2.43E-08 | MAPK14/RELA/JUN/FOS/MAPK1/EGF/NFKBIA/RAF1/HIF1A/STAT1/IFNG/CHUK | 12 |
| hsa04932 | Non-alcoholic fatty liver disease | 15/127 | 155/8093 | 1.60E-08 | 9.35E-08 | 3.36E-08 | PPARG/MAPK14/GSK3B/RELA/BAX/JUN/CASP3/CASP8/RXRA/FOS/IL6R/IL1B/CXCL8/IL1A/PPARA | 15 |
| hsa05152 | Tuberculosis | 16/127 | 180/8093 | 1.75E-08 | 9.78E-08 | 3.52E-08 | NOS2/MAPK14/RELA/BCL2/BAX/CASP9/CASP3/CASP8/MAPK1/IL10RA/RAF1/STAT1/IL1B/IFNG/IL1A/CTSD | 16 |
| hsa05213 | Endometrial cancer | 10/127 | 58/8093 | 1.75E-08 | 9.78E-08 | 3.52E-08 | GSK3B/BAX/CASP9/CDKN1A/MAPK1/EGF/TP53/RAF1/ERBB2/MYC | 10 |
| hsa04370 | VEGF signaling pathway | 10/127 | 59/8093 | 2.08E-08 | 1.14E-07 | 4.08E-08 | PTGS2/MAPK14/KDR/CASP9/PRKCA/MAPK1/RAF1/PRKCB/NOS3/HSPB1 | 10 |
| hsa05133 | Pertussis | 11/127 | 76/8093 | 2.22E-08 | 1.16E-07 | 4.17E-08 | NOS2/MAPK14/RELA/JUN/CASP3/FOS/MAPK1/IL1B/CXCL8/IL1A/IRF1 | 11 |
| hsa05220 | Chronic myeloid leukemia | 11/127 | 76/8093 | 2.22E-08 | 1.16E-07 | 4.17E-08 | RELA/BAX/CDKN1A/MAPK1/RB1/TP53/NFKBIA/RAF1/MYC/CHUK/E2F1 | 11 |
| hsa04010 | MAPK signaling pathway | 20/127 | 294/8093 | 2.53E-08 | 1.29E-07 | 4.65E-08 | MAPK14/KDR/RELA/JUN/CASP3/PRKCA/FOS/MAPK1/EGF/TP53/RAF1/ERBB2/MYC/IL1B/PRKCB/HSPB1/IL1A/CHUK/IGF2/ERBB3 | 20 |
| hsa05202 | Transcriptional misregulation in cancer | 16/127 | 192/8093 | 4.38E-08 | 2.20E-07 | 7.91E-08 | PPARG/CCNA2/RELA/BAX/RXRA/PLAU/MMP9/CDKN1A/TP53/RUNX1T1/MYC/CXCL8/PLAT/MPO/RUNX2/IGFBP3 | 16 |
| hsa05146 | Amoebiasis | 12/127 | 102/8093 | 5.40E-08 | 2.66E-07 | 9.54E-08 | NOS2/RELA/CASP3/PRKCA/IL1B/CXCL8/PRKCB/HSPB1/COL1A1/IFNG/COL3A1/CXCL2 | 12 |
| hsa04625 | C-type lectin receptor signaling pathway | 12/127 | 104/8093 | 6.72E-08 | 3.18E-07 | 1.14E-07 | PTGS2/MAPK14/RELA/JUN/CASP8/MAPK1/NFKBIA/RAF1/STAT1/IL1B/CHUK/IRF1 | 12 |
| hsa04660 | T cell receptor signaling pathway | 12/127 | 104/8093 | 6.72E-08 | 3.18E-07 | 1.14E-07 | MAPK14/GSK3B/RELA/JUN/IL4/FOS/MAPK1/NFKBIA/RAF1/IFNG/CHUK/CD40LG | 12 |
| hsa05226 | Gastric cancer | 14/127 | 149/8093 | 7.23E-08 | 3.34E-07 | 1.20E-07 | GSK3B/CDK2/BCL2/BAX/RXRA/CDKN1A/MAPK1/EGF/RB1/TP53/RAF1/ERBB2/MYC/E2F1 | 14 |
| hsa04012 | ErbB signaling pathway | 11/127 | 85/8093 | 7.33E-08 | 3.34E-07 | 1.20E-07 | GSK3B/JUN/PRKCA/CDKN1A/MAPK1/EGF/RAF1/ERBB2/MYC/PRKCB/ERBB3 | 11 |
| hsa04380 | Osteoclast differentiation | 13/127 | 128/8093 | 8.66E-08 | 3.88E-07 | 1.39E-07 | PPARG/MAPK14/RELA/NCF1/JUN/FOS/MAPK1/NFKBIA/STAT1/IL1B/IFNG/IL1A/CHUK | 13 |
| hsa05171 | Coronavirus disease - COVID-19 | 17/127 | 232/8093 | 1.07E-07 | 4.71E-07 | 1.69E-07 | MAPK14/F2/RELA/JUN/PRKCA/FOS/MAPK1/IL6R/NFKBIA/MMP1/STAT1/IL1B/CCL2/CXCL8/PRKCB/CXCL10/CHUK | 17 |
| hsa04917 | Prolactin signaling pathway | 10/127 | 70/8093 | 1.13E-07 | 4.90E-07 | 1.76E-07 | ESR1/ESR2/MAPK14/GSK3B/RELA/FOS/MAPK1/RAF1/STAT1/IRF1 | 10 |
| hsa04658 | Th1 and Th2 cell differentiation | 11/127 | 92/8093 | 1.68E-07 | 7.14E-07 | 2.57E-07 | MAPK14/RELA/JUN/IL4/FOS/MAPK1/NFKBIA/STAT1/IL2RA/IFNG/CHUK | 11 |
| hsa05165 | Human papillomavirus infection | 20/127 | 331/8093 | 1.80E-07 | 7.51E-07 | 2.70E-07 | PTGS2/GSK3B/CDK2/CCNA2/RELA/BAX/CASP3/CASP8/CDKN1A/MAPK1/EGF/RB1/TP53/RAF1/STAT1/COL1A1/CHUK/SPP1/E2F1/IRF1 | 20 |
| hsa04915 | Estrogen signaling pathway | 13/127 | 138/8093 | 2.12E-07 | 8.67E-07 | 3.12E-07 | ESR1/ESR2/HSP90AA1/OPRM1/BCL2/JUN/MMP2/MMP9/FOS/MAPK1/RAF1/NOS3/CTSD | 13 |
| hsa05214 | Glioma | 10/127 | 75/8093 | 2.22E-07 | 8.95E-07 | 3.22E-07 | BAX/PRKCA/CDKN1A/MAPK1/EGF/RB1/TP53/RAF1/PRKCB/E2F1 | 10 |
| hsa04919 | Thyroid hormone signaling pathway | 12/127 | 121/8093 | 3.63E-07 | 1.44E-06 | 5.18E-07 | ESR1/GSK3B/CASP9/PRKCA/RXRA/MAPK1/TP53/RAF1/HIF1A/STAT1/MYC/PRKCB | 12 |
| hsa05130 | Pathogenic Escherichia coli infection | 15/127 | 197/8093 | 3.90E-07 | 1.52E-06 | 5.47E-07 | MAPK14/F2/RELA/BAX/CASP9/JUN/CASP3/CASP8/FOS/MAPK1/NFKBIA/IL1B/CXCL8/CLDN4/CHUK | 15 |
| hsa04110 | Cell cycle | 12/127 | 126/8093 | 5.66E-07 | 2.17E-06 | 7.82E-07 | GSK3B/CDK2/CCNA2/CHEK1/CDKN1A/RB1/TP53/CDK1/MYC/CCNB1/CHEK2/E2F1 | 12 |
| hsa05221 | Acute myeloid leukemia | 9/127 | 67/8093 | 8.62E-07 | 3.26E-06 | 1.17E-06 | CCNA2/PPARD/RELA/MAPK1/RAF1/RUNX1T1/MYC/MPO/CHUK | 9 |
| hsa05144 | Malaria | 8/127 | 50/8093 | 9.11E-07 | 3.39E-06 | 1.22E-06 | ICAM1/IL1B/CCL2/SELE/VCAM1/CXCL8/IFNG/CD40LG | 8 |
| hsa04621 | NOD-like receptor signaling pathway | 14/127 | 184/8093 | 9.87E-07 | 3.62E-06 | 1.30E-06 | MAPK14/HSP90AA1/RELA/BCL2/JUN/CASP8/MAPK1/NFKBIA/STAT1/IL1B/CCL2/CXCL8/CXCL2/CHUK | 14 |
| hsa05143 | African trypanosomiasis | 7/127 | 37/8093 | 1.38E-06 | 4.93E-06 | 1.77E-06 | PRKCA/ICAM1/IL1B/SELE/VCAM1/PRKCB/IFNG | 7 |
| hsa05216 | Thyroid cancer | 7/127 | 37/8093 | 1.38E-06 | 4.93E-06 | 1.77E-06 | PPARG/BAX/RXRA/CDKN1A/MAPK1/TP53/MYC | 7 |
| hsa05206 | MicroRNAs in cancer | 18/127 | 310/8093 | 1.42E-06 | 4.97E-06 | 1.79E-06 | PTGS2/BCL2/CASP3/PRKCA/PLAU/MMP9/CDKN1A/MAPK1/TP53/RAF1/ERBB2/HMOX1/MYC/PRKCB/CYP1B1/RASSF1/E2F1/ERBB3 | 18 |
| hsa05132 | Salmonella infection | 16/127 | 249/8093 | 1.54E-06 | 5.33E-06 | 1.91E-06 | MAPK14/HSP90AA1/RELA/BCL2/BAX/JUN/CASP3/CASP8/FOS/MAPK1/NFKBIA/RAF1/MYC/IL1B/CXCL8/CHUK | 16 |
| hsa04062 | Chemokine signaling pathway | 14/127 | 192/8093 | 1.65E-06 | 5.62E-06 | 2.02E-06 | GSK3B/RELA/NCF1/MAPK1/NFKBIA/RAF1/STAT1/CCL2/CXCL8/PRKCB/CXCL11/CXCL2/CXCL10/CHUK | 14 |
| hsa05323 | Rheumatoid arthritis | 10/127 | 93/8093 | 1.70E-06 | 5.74E-06 | 2.06E-06 | JUN/FOS/MMP1/ICAM1/IL1B/CCL2/CXCL8/IFNG/IL1A/CXCL2 | 10 |
| hsa04071 | Sphingolipid signaling pathway | 11/127 | 119/8093 | 2.31E-06 | 7.68E-06 | 2.76E-06 | MAPK14/RELA/BCL2/BAX/PRKCA/MAPK1/TP53/RAF1/PRKCB/NOS3/CTSD | 11 |
| hsa05134 | Legionellosis | 8/127 | 57/8093 | 2.56E-06 | 8.39E-06 | 3.01E-06 | RELA/CASP9/CASP3/CASP8/NFKBIA/IL1B/CXCL8/CXCL2 | 8 |
| hsa05415 | Diabetic cardiomyopathy | 14/127 | 203/8093 | 3.19E-06 | 1.03E-05 | 3.71E-06 | MAPK14/GSK3B/RELA/NCF1/PRKCA/MMP2/MMP9/PRKCB/NOS3/COL1A1/PARP1/COL3A1/PPARA/CTSD | 14 |
| hsa05203 | Viral carcinogenesis | 14/127 | 204/8093 | 3.38E-06 | 1.08E-05 | 3.88E-06 | CDK2/CCNA2/CHEK1/RELA/BAX/JUN/CASP3/CASP8/CDKN1A/MAPK1/RB1/TP53/NFKBIA/CDK1 | 14 |
| hsa04662 | B cell receptor signaling pathway | 9/127 | 82/8093 | 4.85E-06 | 1.53E-05 | 5.49E-06 | GSK3B/RELA/JUN/FOS/MAPK1/NFKBIA/RAF1/PRKCB/CHUK | 9 |
| hsa04215 | Apoptosis - multiple species | 6/127 | 32/8093 | 8.60E-06 | 2.68E-05 | 9.63E-06 | BCL2/BAX/CASP9/CASP3/CASP8/BIRC5 | 6 |
| hsa05135 | Yersinia infection | 11/127 | 137/8093 | 9.13E-06 | 2.81E-05 | 1.01E-05 | MAPK14/GSK3B/RELA/JUN/FOS/MAPK1/NFKBIA/IL1B/CCL2/CXCL8/CHUK | 11 |
| hsa04726 | Serotonergic synapse | 10/127 | 115/8093 | 1.18E-05 | 3.57E-05 | 1.28E-05 | PTGS1/PTGS2/SLC6A4/CASP3/PRKCA/HTR3A/MAPK1/RAF1/PRKCB/ALOX5 | 10 |
| hsa05120 | Epithelial cell signaling in Helicobacter pylori infection | 8/127 | 70/8093 | 1.23E-05 | 3.69E-05 | 1.33E-05 | MAPK14/RELA/JUN/CASP3/NFKBIA/CXCL8/CXCL2/CHUK | 8 |
| hsa05022 | Pathways of neurodegeneration - multiple diseases | 21/127 | 476/8093 | 1.40E-05 | 4.14E-05 | 1.49E-05 | NOS2/PTGS2/MAPK14/GSK3B/RELA/CHRM3/CHRNA7/BCL2/BAX/CASP9/CASP3/CASP8/PRKCA/MAPK1/RAF1/SOD1/HSPA5/IL1B/PRKCB/IL1A/PSMD3 | 21 |
| hsa04510 | Focal adhesion | 13/127 | 201/8093 | 1.47E-05 | 4.30E-05 | 1.55E-05 | GSK3B/KDR/BCL2/JUN/PRKCA/MAPK1/EGF/RAF1/ERBB2/CAV1/PRKCB/COL1A1/SPP1 | 13 |
| hsa05218 | Melanoma | 8/127 | 72/8093 | 1.52E-05 | 4.40E-05 | 1.58E-05 | BAX/CDKN1A/MAPK1/EGF/RB1/TP53/RAF1/E2F1 | 8 |
| hsa04722 | Neurotrophin signaling pathway | 10/127 | 119/8093 | 1.59E-05 | 4.55E-05 | 1.64E-05 | MAPK14/GSK3B/RELA/BCL2/BAX/JUN/MAPK1/TP53/NFKBIA/RAF1 | 10 |
| hsa04020 | Calcium signaling pathway | 14/127 | 240/8093 | 2.19E-05 | 6.18E-05 | 2.22E-05 | NOS2/KDR/CHRM3/ADRA1A/ADRA1B/CHRNA7/PRKCA/ADRA1D/EGF/ERBB2/PTGER3/PRKCB/NOS3/ERBB3 | 14 |
| hsa04928 | Parathyroid hormone synthesis, secretion and action | 9/127 | 106/8093 | 3.96E-05 | 0.00011063 | 3.98E-05 | BCL2/PRKCA/RXRA/FOS/CDKN1A/MAPK1/RAF1/PRKCB/RUNX2 | 9 |
| hsa04630 | JAK-STAT signaling pathway | 11/127 | 162/8093 | 4.42E-05 | 0.000122244 | 4.39E-05 | BCL2/IL4/CDKN1A/IL10RA/EGF/IL6R/RAF1/STAT1/MYC/IL2RA/IFNG | 11 |
| hsa05321 | Inflammatory bowel disease | 7/127 | 65/8093 | 6.49E-05 | 0.000177478 | 6.38E-05 | RELA/JUN/IL4/STAT1/IL1B/IFNG/IL1A | 7 |
| hsa04670 | Leukocyte transendothelial migration | 9/127 | 114/8093 | 7.03E-05 | 0.000190049 | 6.83E-05 | MAPK14/NCF1/PRKCA/MMP2/MMP9/ICAM1/VCAM1/PRKCB/CLDN4 | 9 |
| hsa05204 | Chemical carcinogenesis - DNA adducts | 7/127 | 69/8093 | 9.55E-05 | 0.00025545 | 9.18E-05 | PTGS2/CYP3A4/CYP1A2/CYP1A1/CYP1B1/GSTP1/GSTM1 | 7 |
| hsa04935 | Growth hormone synthesis, secretion and action | 9/127 | 119/8093 | 9.82E-05 | 0.00025982 | 9.34E-05 | MAPK14/GSK3B/PRKCA/FOS/MAPK1/RAF1/STAT1/PRKCB/IGFBP3 | 9 |
| hsa04622 | RIG-I-like receptor signaling pathway | 7/127 | 70/8093 | 0.000104781 | 0.000271326 | 9.75E-05 | MAPK14/RELA/CASP8/NFKBIA/CXCL8/CXCL10/CHUK | 7 |
| hsa05230 | Central carbon metabolism in cancer | 7/127 | 70/8093 | 0.000104781 | 0.000271326 | 9.75E-05 | MAPK1/TP53/RAF1/HIF1A/ERBB2/MYC/HK2 | 7 |
| hsa05131 | Shigellosis | 13/127 | 247/8093 | 0.000125215 | 0.000320864 | 0.00011533 | MAPK14/GSK3B/RELA/BCL2/BAX/JUN/MAPK1/TP53/NFKBIA/IL1B/CXCL8/CHUK/HK2 | 13 |
| hsa05231 | Choline metabolism in cancer | 8/127 | 98/8093 | 0.000142262 | 0.000360789 | 0.00012968 | JUN/PRKCA/FOS/MAPK1/EGF/RAF1/HIF1A/PRKCB | 8 |
| hsa04061 | Viral protein interaction with cytokine and cytokine receptor | 8/127 | 100/8093 | 0.000163884 | 0.000411381 | 0.000147865 | IL10RA/IL6R/CCL2/CXCL8/IL2RA/CXCL11/CXCL2/CXCL10 | 8 |
| hsa04217 | Necroptosis | 10/127 | 159/8093 | 0.000187857 | 0.000462832 | 0.000166358 | HSP90AA1/PYGM/BCL2/BAX/CASP8/STAT1/IL1B/IFNG/IL1A/PARP1 | 10 |
| hsa04914 | Progesterone-mediated oocyte maturation | 8/127 | 102/8093 | 0.000188143 | 0.000462832 | 0.000166358 | MAPK14/HSP90AA1/CDK2/CCNA2/MAPK1/RAF1/CDK1/CCNB1 | 8 |
| hsa00980 | Metabolism of xenobiotics by cytochrome P450 | 7/127 | 78/8093 | 0.000208166 | 0.000507018 | 0.00018224 | CYP3A4/CYP2B6/CYP1A2/CYP1A1/CYP1B1/GSTP1/GSTM1 | 7 |
| hsa04931 | Insulin resistance | 8/127 | 108/8093 | 0.000279237 | 0.000673454 | 0.000242063 | GSK3B/PTPN1/PYGM/RELA/NFKBIA/PRKCB/NOS3/PPARA | 8 |
| hsa05416 | Viral myocarditis | 6/127 | 60/8093 | 0.000332016 | 0.000792969 | 0.000285021 | CASP9/CASP3/CASP8/CAV1/ICAM1/CD40LG | 6 |
| hsa05020 | Prion disease | 13/127 | 273/8093 | 0.000335937 | 0.000794619 | 0.000285614 | MAPK14/GSK3B/NCF1/BAX/CASP9/CASP3/MAPK1/SOD1/HSPA5/CAV1/IL1B/IL1A/PSMD3 | 13 |
| hsa04725 | Cholinergic synapse | 8/127 | 113/8093 | 0.000380244 | 0.000890858 | 0.000320206 | ACHE/CHRM3/CHRNA7/BCL2/PRKCA/FOS/MAPK1/PRKCB | 8 |
| hsa04929 | GnRH secretion | 6/127 | 64/8093 | 0.00047245 | 0.001096441 | 0.000394099 | ESR2/PRKCA/MAPK1/RAF1/PRKCB/SPP1 | 6 |
| hsa04912 | GnRH signaling pathway | 7/127 | 93/8093 | 0.00061473 | 0.001413304 | 0.000507991 | MAPK14/JUN/PRKCA/MMP2/MAPK1/RAF1/PRKCB | 7 |
| hsa04664 | Fc epsilon RI signaling pathway | 6/127 | 68/8093 | 0.00065539 | 0.001492833 | 0.000536577 | MAPK14/PRKCA/IL4/MAPK1/RAF1/ALOX5 | 6 |
| hsa04921 | Oxytocin signaling pathway | 9/127 | 154/8093 | 0.000678148 | 0.001530499 | 0.000550115 | PTGS2/JUN/PRKCA/FOS/CDKN1A/MAPK1/RAF1/PRKCB/NOS3 | 9 |
| hsa04060 | Cytokine-cytokine receptor interaction | 13/127 | 295/8093 | 0.000702229 | 0.00157044 | 0.000564471 | IL4/IL10RA/IL6R/IL1B/CCL2/CXCL8/IL2RA/IFNG/IL1A/CXCL11/CXCL2/CXCL10/CD40LG | 13 |
| hsa05010 | Alzheimer disease | 15/127 | 384/8093 | 0.000952219 | 0.002110324 | 0.000758525 | NOS2/PTGS2/GSK3B/RELA/CHRM3/CHRNA7/CASP9/CASP3/CASP8/MAPK1/RAF1/IL1B/IL1A/PSMD3/CHUK | 15 |
| hsa04068 | FoxO signaling pathway | 8/127 | 131/8093 | 0.001016337 | 0.002232312 | 0.000802371 | MAPK14/CDK2/CDKN1A/MAPK1/EGF/RAF1/CCNB1/CHUK | 8 |
| hsa01523 | Antifolate resistance | 4/127 | 31/8093 | 0.001310679 | 0.002853336 | 0.001025589 | RELA/IL1B/ABCG2/CHUK | 4 |
| hsa04610 | Complement and coagulation cascades | 6/127 | 85/8093 | 0.002109076 | 0.004551164 | 0.001635848 | F2/PLAU/F3/PLAT/THBD/SERPINE1 | 6 |
| hsa04540 | Gap junction | 6/127 | 88/8093 | 0.002515659 | 0.005381324 | 0.001934237 | PRKCA/MAPK1/EGF/RAF1/CDK1/PRKCB | 6 |
| hsa04960 | Aldosterone-regulated sodium reabsorption | 4/127 | 37/8093 | 0.002558741 | 0.005426295 | 0.001950401 | NR3C2/PRKCA/MAPK1/PRKCB | 4 |
| hsa00590 | Arachidonic acid metabolism | 5/127 | 61/8093 | 0.002591664 | 0.005449139 | 0.001958612 | PTGS1/PTGS2/LTA4H/CYP2B6/ALOX5 | 5 |
| hsa04623 | Cytosolic DNA-sensing pathway | 5/127 | 63/8093 | 0.002986771 | 0.006226658 | 0.00223808 | RELA/NFKBIA/IL1B/CXCL10/CHUK | 5 |
| hsa04611 | Platelet activation | 7/127 | 124/8093 | 0.00327485 | 0.006769857 | 0.002433325 | PTGS1/MAPK14/F2/MAPK1/NOS3/COL1A1/COL3A1 | 7 |
| hsa04970 | Salivary secretion | 6/127 | 93/8093 | 0.003320798 | 0.006807635 | 0.002446903 | CHRM3/ADRA1A/ADRA1B/PRKCA/ADRA1D/PRKCB | 6 |
| hsa04014 | Ras signaling pathway | 10/127 | 232/8093 | 0.003442196 | 0.006998183 | 0.002515393 | KDR/RELA/PRKCA/MAPK1/EGF/RAF1/PRKCB/CHUK/RASSF1/IGF2 | 10 |
| hsa04920 | Adipocytokine signaling pathway | 5/127 | 69/8093 | 0.004432528 | 0.008803287 | 0.003164211 | RELA/RXRA/NFKBIA/PPARA/CHUK | 5 |
| hsa05211 | Renal cell carcinoma | 5/127 | 69/8093 | 0.004432528 | 0.008803287 | 0.003164211 | JUN/CDKN1A/MAPK1/RAF1/HIF1A | 5 |
| hsa04650 | Natural killer cell mediated cytotoxicity | 7/127 | 131/8093 | 0.004437429 | 0.008803287 | 0.003164211 | CASP3/PRKCA/MAPK1/RAF1/ICAM1/PRKCB/IFNG | 7 |
| hsa04270 | Vascular smooth muscle contraction | 7/127 | 133/8093 | 0.004820822 | 0.009487378 | 0.003410098 | ADRA1A/ADRA1B/PRKCA/ADRA1D/MAPK1/RAF1/PRKCB | 7 |
| hsa00982 | Drug metabolism - cytochrome P450 | 5/127 | 72/8093 | 0.005316741 | 0.010298569 | 0.003701668 | CYP3A4/CYP2B6/CYP1A2/GSTP1/GSTM1 | 5 |
| hsa04137 | Mitophagy - animal | 5/127 | 72/8093 | 0.005316741 | 0.010298569 | 0.003701668 | RELA/JUN/TP53/HIF1A/E2F1 | 5 |
| hsa04910 | Insulin signaling pathway | 7/127 | 137/8093 | 0.00566206 | 0.010881772 | 0.003911291 | GSK3B/PTPN1/PYGM/MAPK1/RAF1/ACACA/HK2 | 7 |
| hsa04371 | Apelin signaling pathway | 7/127 | 138/8093 | 0.005888578 | 0.011229381 | 0.004036235 | NOS2/MAPK1/RAF1/NOS3/PLAT/SERPINE1/SPP1 | 7 |
| hsa03320 | PPAR signaling pathway | 5/127 | 76/8093 | 0.00668092 | 0.012642356 | 0.004544108 | PPARG/PPARD/RXRA/MMP1/PPARA | 5 |
| hsa04913 | Ovarian steroidogenesis | 4/127 | 51/8093 | 0.008181718 | 0.015364142 | 0.005522413 | PTGS2/CYP1A1/CYP1B1/ALOX5 | 4 |
| hsa04261 | Adrenergic signaling in cardiomyocytes | 7/127 | 150/8093 | 0.009165819 | 0.017081753 | 0.006139783 | MAPK14/ADRA1A/ADRA1B/BCL2/PRKCA/ADRA1D/MAPK1 | 7 |
| hsa04613 | Neutrophil extracellular trap formation | 8/127 | 190/8093 | 0.009908632 | 0.018327245 | 0.006587456 | MAPK14/RELA/NCF1/PRKCA/MAPK1/RAF1/PRKCB/MPO | 8 |
| hsa04934 | Cushing syndrome | 7/127 | 155/8093 | 0.010866695 | 0.019949306 | 0.007170482 | GSK3B/CDK2/CDKN1A/MAPK1/RB1/AHR/E2F1 | 7 |
| hsa04730 | Long-term depression | 4/127 | 60/8093 | 0.0143365 | 0.026124289 | 0.009389988 | PRKCA/MAPK1/RAF1/PRKCB | 4 |
| hsa00140 | Steroid hormone biosynthesis | 4/127 | 61/8093 | 0.015160699 | 0.02742303 | 0.009856801 | CYP3A4/CYP1A2/CYP1A1/CYP1B1 | 4 |
| hsa04310 | Wnt signaling pathway | 7/127 | 166/8093 | 0.015400544 | 0.027653532 | 0.009939652 | GSK3B/PPARD/JUN/PRKCA/TP53/MYC/PRKCB | 7 |
| hsa04114 | Oocyte meiosis | 6/127 | 131/8093 | 0.016853788 | 0.029904882 | 0.010748867 | AR/MAPK14/CDK2/MAPK1/CDK1/CCNB1 | 6 |
| hsa05217 | Basal cell carcinoma | 4/127 | 63/8093 | 0.016897474 | 0.029904882 | 0.010748867 | GSK3B/BAX/CDKN1A/TP53 | 4 |
| hsa04666 | Fc gamma R-mediated phagocytosis | 5/127 | 97/8093 | 0.017986925 | 0.031605597 | 0.011360163 | NCF1/PRKCA/MAPK1/RAF1/PRKCB | 5 |
| hsa04640 | Hematopoietic cell lineage | 5/127 | 99/8093 | 0.019477787 | 0.033982522 | 0.012214514 | IL4/IL6R/IL1B/IL2RA/IL1A | 5 |
| hsa04720 | Long-term potentiation | 4/127 | 67/8093 | 0.020732605 | 0.035917049 | 0.012909851 | PRKCA/MAPK1/RAF1/PRKCB | 4 |
| hsa04916 | Melanogenesis | 5/127 | 101/8093 | 0.021047795 | 0.036208095 | 0.013014463 | GSK3B/PRKCA/MAPK1/RAF1/PRKCB | 5 |
| hsa05330 | Allograft rejection | 3/127 | 38/8093 | 0.021350853 | 0.036474375 | 0.013110173 | IL4/IFNG/CD40LG | 3 |
| hsa04024 | cAMP signaling pathway | 8/127 | 219/8093 | 0.021667115 | 0.036586946 | 0.013150635 | RELA/JUN/FOS/MAPK1/NFKBIA/RAF1/PTGER3/PPARA | 8 |
| hsa00830 | Retinol metabolism | 4/127 | 68/8093 | 0.021768225 | 0.036586946 | 0.013150635 | CYP3A4/CYP2B6/CYP1A2/CYP1A1 | 4 |
| hsa04972 | Pancreatic secretion | 5/127 | 102/8093 | 0.021862931 | 0.036586946 | 0.013150635 | CA2/PRSS1/CHRM3/PRKCA/PRKCB | 5 |
| hsa05031 | Amphetamine addiction | 4/127 | 69/8093 | 0.022835038 | 0.037955535 | 0.013642554 | JUN/PRKCA/FOS/PRKCB | 4 |
| hsa00380 | Tryptophan metabolism | 3/127 | 42/8093 | 0.027775694 | 0.045552139 | 0.016373041 | CYP1A2/CYP1A1/CYP1B1 | 3 |
| hsa05332 | Graft-versus-host disease | 3/127 | 42/8093 | 0.027775694 | 0.045552139 | 0.016373041 | IL1B/IFNG/IL1A | 3 |
| hsa04072 | Phospholipase D signaling pathway | 6/127 | 148/8093 | 0.028709859 | 0.046772353 | 0.016811629 | F2/PRKCA/MAPK1/EGF/RAF1/CXCL8 | 6 |
| hsa04940 | Type I diabetes mellitus | 3/127 | 43/8093 | 0.029525288 | 0.047784347 | 0.017175375 | IL1B/IFNG/IL1A | 3 |
| hsa04918 | Thyroid hormone synthesis | 4/127 | 75/8093 | 0.029901279 | 0.048076567 | 0.017280409 | PRKCA/HSPA5/PRKCB/DUOX2 | 4 |
| hsa04971 | Gastric acid secretion | 4/127 | 76/8093 | 0.031191299 | 0.049825062 | 0.01790888 | CA2/CHRM3/PRKCA/PRKCB | 4 |
| hsa04150 | mTOR signaling pathway | 6/127 | 155/8093 | 0.034877752 | 0.055354368 | 0.019896307 | GSK3B/PRKCA/MAPK1/RAF1/PRKCB/CHUK | 6 |
| hsa00983 | Drug metabolism - other enzymes | 4/127 | 80/8093 | 0.036676287 | 0.057835683 | 0.020788179 | CYP3A4/MPO/GSTP1/GSTM1 | 4 |
| hsa04080 | Neuroactive ligand-receptor interaction | 10/127 | 341/8093 | 0.041466356 | 0.064972762 | 0.023353496 | PRSS1/F2/CHRM3/ADRA1A/ADRA1B/CHRNA2/OPRM1/CHRNA7/ADRA1D/PTGER3 | 10 |
| hsa00330 | Arginine and proline metabolism | 3/127 | 51/8093 | 0.045554962 | 0.070927346 | 0.025493783 | NOS2/ODC1/NOS3 | 3 |
| hsa00220 | Arginine biosynthesis | 2/127 | 22/8093 | 0.045999251 | 0.071168653 | 0.025580517 | NOS2/NOS3 | 2 |
| hsa04015 | Rap1 signaling pathway | 7/127 | 210/8093 | 0.046948577 | 0.072183437 | 0.025945266 | MAPK14/KDR/PRKCA/MAPK1/EGF/RAF1/PRKCB | 7 |
| hsa04022 | cGMP-PKG signaling pathway | 6/127 | 167/8093 | 0.047326576 | 0.072213445 | 0.025956052 | ADRA1A/ADRA1B/ADRA1D/MAPK1/RAF1/NOS3 | 6 |
| hsa05168 | Herpes simplex virus 1 infection | 13/127 | 497/8093 | 0.047555196 | 0.072213445 | 0.025956052 | RELA/BCL2/BAX/CASP9/CASP3/CASP8/TP53/NFKBIA/STAT1/IL1B/CCL2/IFNG/CHUK | 13 |
